# Supplementary material for: An RNA sponge controls quorum sensing dynamics and biofilm formation in Vibrio cholerae
Source: Nat Commun. 2022 Dec 8;13:7585. doi: 10.1038/s41467-022-35261-x (PMC9732341; doi:10.1038/s41467-022-35261-x)
Supplement: Supplementary file 1 — Supplementary Information [file 41467_2022_35261_MOESM1_ESM.pdf]

Supplementary Information

An RNA sponge controls quorum sensing dynamics and biofilm formation in *Vibrio cholerae*

Michaela Huber[1*], Anne Lippegaus[1*], Sahar Melamed[2,3], Malte Siemers[1], Benjamin R. Wucher[4], Mona Hoyos[1], Carey Nadell[4], Gisela Storz[2], and Kai Papenfort[1,5 #]

[1] Friedrich Schiller University Jena, Institute of Microbiology, 07745 Jena, Germany

[2] Division of Molecular and Cellular Biology, Eunice Kennedy Shriver National Institute of Child Health and Human Development, Bethesda, MD 20892-5430, USA

[3] Department of Microbiology and Molecular Genetics, Faculty of Medicine, The Hebrew University of Jerusalem, Jerusalem 9112102, Israel.

[4] Department of Biological Sciences, Dartmouth College, Hanover, NH 03755, USA

[5] Microverse Cluster, Friedrich Schiller University Jena, 07743 Jena, Germany.

* these authors contributed equally to this manuscript.

This supplement contains:

Figures S1 to S8

Tables S1 to S4

Supplemental References

TABLE OF CONTENTS

**Figure S1**	RNA duplex distribution and base-pairing predictions
**Figure S2**	Interaction partners of selected sRNAs discovered by RIL-seq
**Figure S3**	sRNA-sRNA interactions
**Figure S4**	Expression pattern of QrrX and the Qrr1-4 sRNAs
**Figure S5**	Stability of QrrX upon induction of Qrr1-4
**Figure S6**	Influence of RNase E and RNase G on QrrX - Qrr4 interaction
**Figure S7**	Transcriptional regulation of QrrX
**Figure S8**	Influence of QrrX on biofilm accumulation
**Table S1**	Previously published sRNA targets captured in chimeras in RIL-seq
**Table S2**	Plasmids used in this study
**Table S3**	Strains used in this study
**Table S4**	Oligonucleotides used in this study

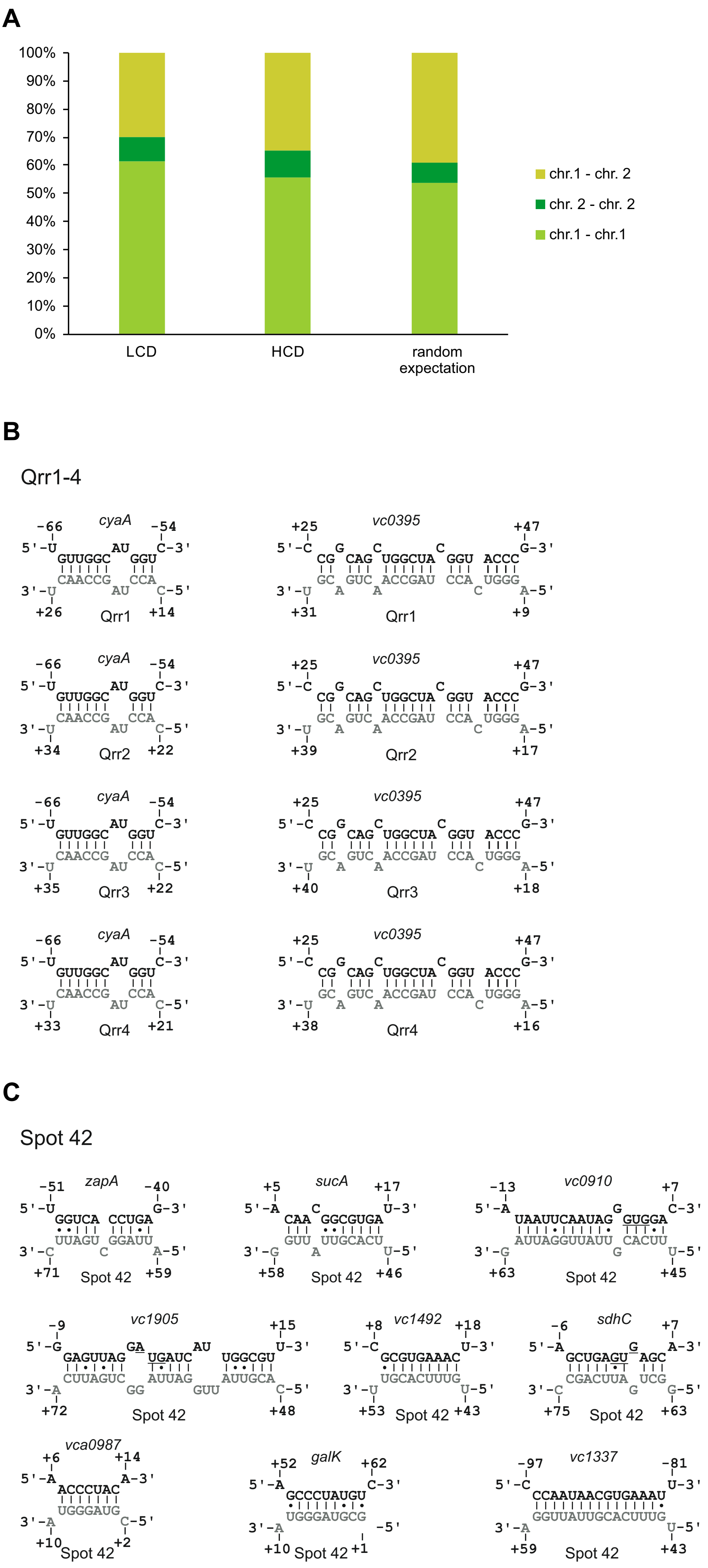
A
100%
90%
80%
70%
60%
50%
40%
30%
20%
10%
0%
LCD
HCD
random expectation
chr.1 - chr. 2
chr. 2 - chr. 2
chr.1 - chr.1
B
Qrr1-4
cyaA
-66
-54
5'-U AU C-3'
GUUGGC GGU
CAACCG CCA
3'-U AU C-5'
+26 Qrr1 +14
vc0395
+25
+47
5'-C G C C G-3'
CG CAG UGGCUA GGU ACCC
GC GUC ACCGAU CCA UGGG
3'-U A A C A-5'
+31 Qrr1 +9
+34 Qrr2 +22
+39 Qrr2 +17
+35 Qrr3 +22
+40 Qrr3 +18
+33 Qrr4 +21
+38 Qrr4 +16
C
Spot 42
zapA
-51 -40
5'-U G-3'
GGUCA CCUGA
UUAGU GGAUU
3'-C C A-5'
+71 Spot 42 +59
sucA
+5 +17
5'-A C U-3'
CAA GGCGUGA
GUU UUGCACU
3'-G A U-5'
+58 Spot 42 +46
vc0910
-13 +7
5'-A G C-3'
UAAUUCAAUAG GUGGA
AUUAGGUUAUU CACUU
3'-G G U-5'
+63 Spot 42 +45
vc1905
-9 +15
5'-G GA AU U-3'
GAGUUAG UGAUC UGGCGU
CUUAGUC AUUAG AUUGCA
3'-A GG GUU C-5'
+72 Spot 42 +48
vc1492
+8 +18
5'-C U-3'
GCGUGAAAC
UGCACUUUG
3'-U U-5'
+53 Spot 42 +43
sdhC
-6 +7
5'-A G A-3'
GCUGAGU AGC
CGACUUA UCG
3'-C G G-5'
+75 Spot 42 +63
vca0987
+6 +14
5'-A A-3'
ACCCUAC
UGGGAUG
3'-A C-5'
+10 Spot 42 +2
galK
+52 +62
5'-A C-3'
GCCCUAUGU
UGGGAUGCG
3'-A -5'
+10 Spot 42 +1
vc1337
-97 -81
5'-C U-3'
CCAAUAACGUGAAAU
GGUUAUUGCACUUUG
3'-A U-5'
+59 Spot 42 +43

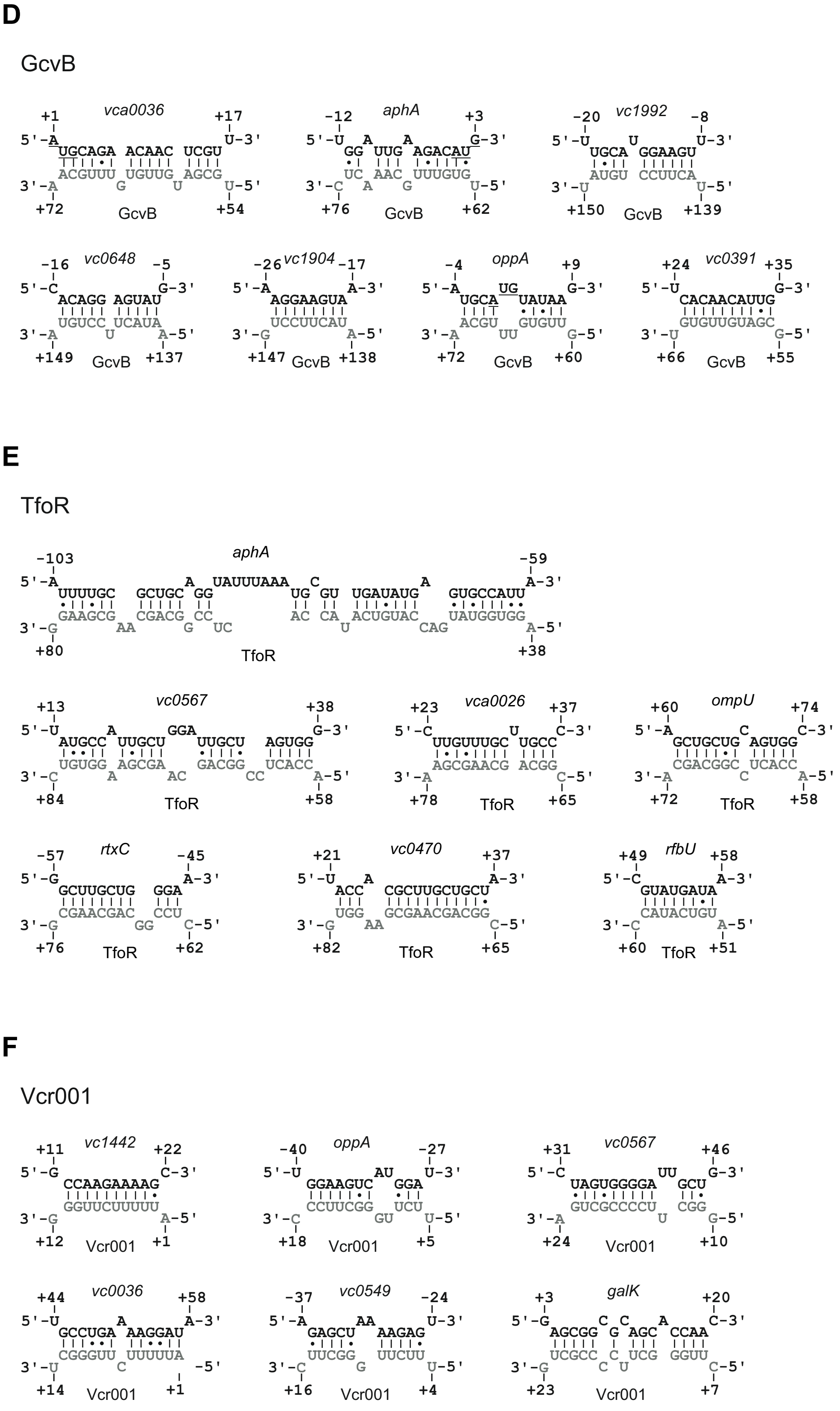

D
GcvB
vca0036
aphA
vc1992
vc0648
vc1904
oppA
vc0391
E
TfoR
aphA
vc0567
vca0026
ompU
rtxC
vc0470
rfbU
F
Vcr001
vc1442
oppA
vc0567
vc0036
vc0549
galK

G

Vcr017

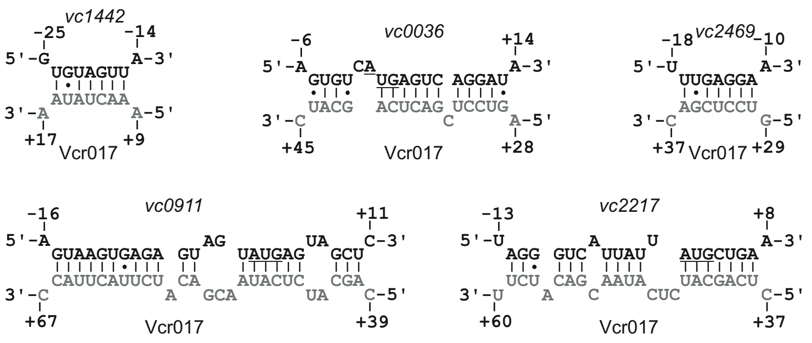

H

Vcr043

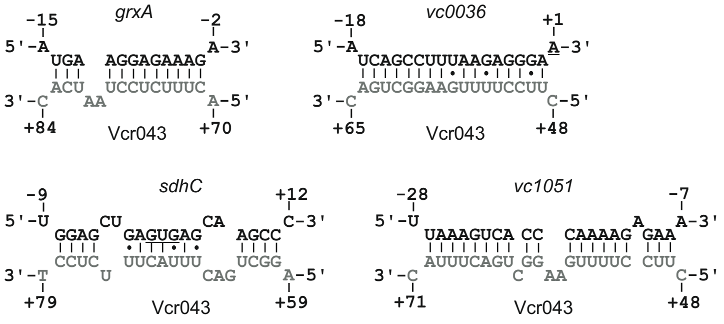

I

Vcr227

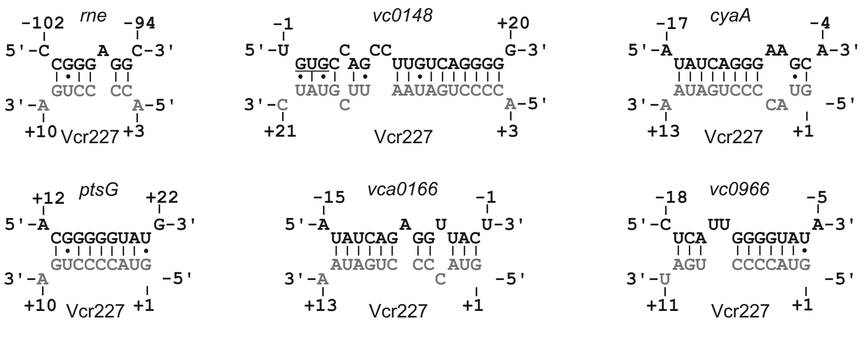

Figure S1: RNA duplex distribution and base-pairing predictions

(A) Chromosomal localization of RNA-RNA chimeras discovered by RIL-seq at low (LCD) and high cell density (HCD), respectively. Percentage of inter- and intrachromosomal interactions together with the expected values are presented in the indicated colors.

(B-I) Predicted base-pairing interactions between selected sRNAs and targets tested in Fig. 1C. The start codon is underlined. Numbers indicate position relative to the start codon (mRNAs) or to the transcriptional start site (sRNAs). *RNAhybrid* (Bielefeld BioInformatics Service) was used for predictions (1). Source data underlying panel **(A)** is provided as a Source Data file.

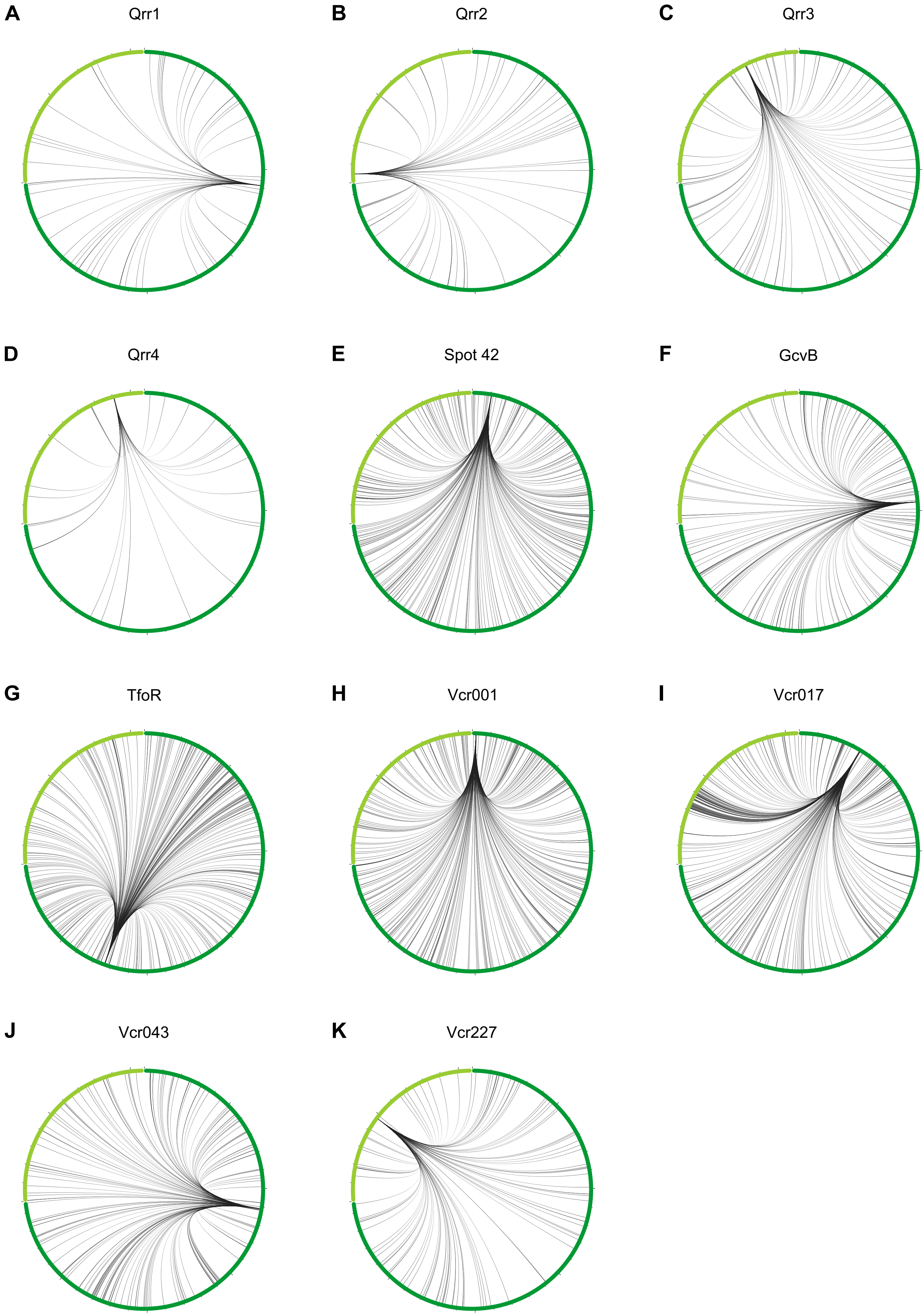
A
Qrr1
B
Qrr2
C
Qrr3
D
Qrr4
E
Spot 42
F
GcvB
G
TfoR
H
Vcr001
I
Vcr017
J
Vcr043
K
Vcr227

Figure S2: Interaction partners of selected sRNAs discovered by RIL-seq

(A-K) Interaction partners of Qrr1-4 (A-D), Spot 42 (E), GcvB (F), TfoR (G), Vcr001 (H), Vcr017 (I), Vcr043 (J) and Vcr227 (K). Circos plots visualizing interaction partners identified by RIL-seq. The first and the second chromosome are marked in dark and light green, respectively. Circos plots were generated using the circos component of the Dash Bio package.

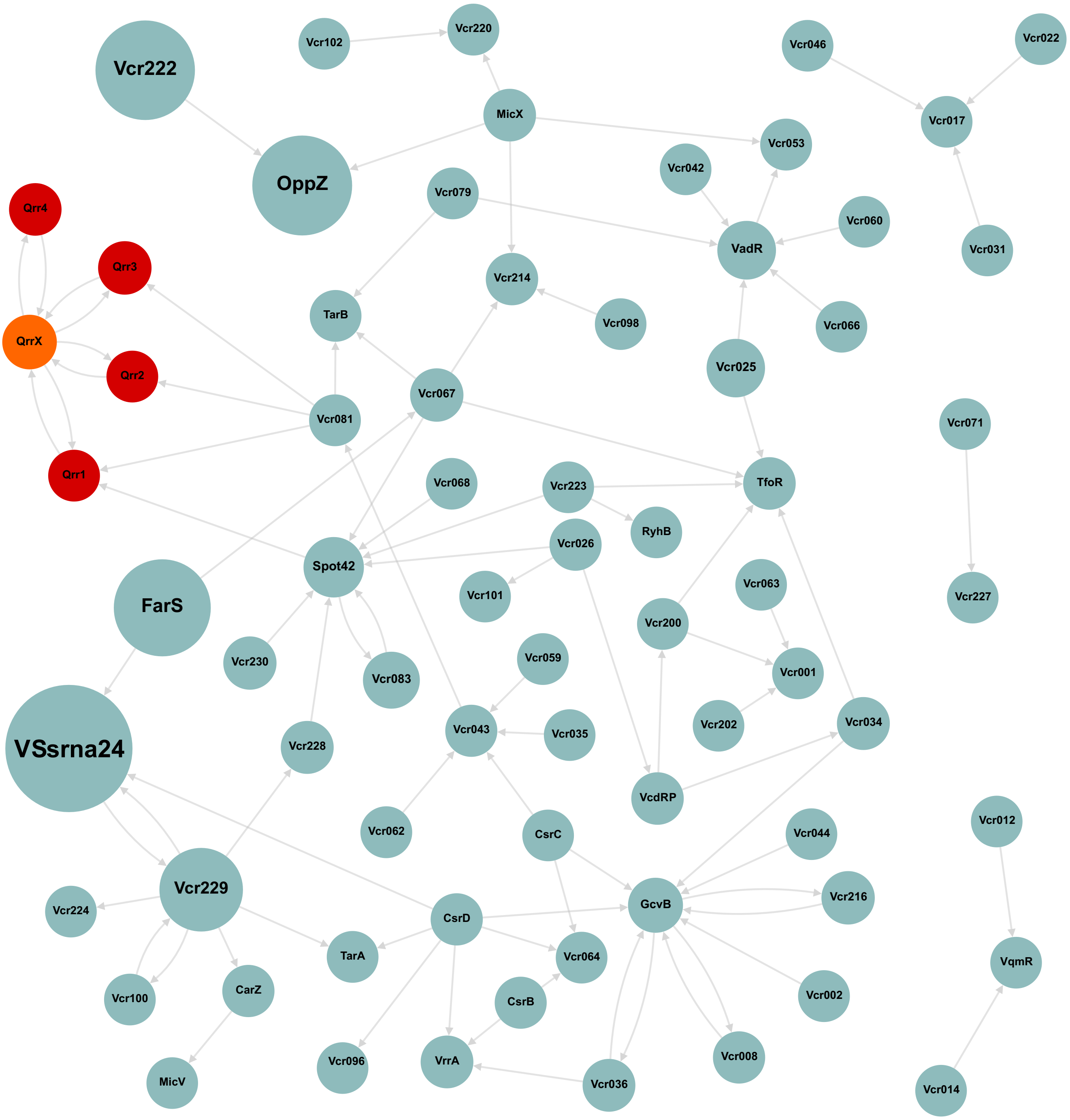

Figure S3: sRNA-sRNA interactions

Graph-based visualization of all sRNA-sRNA interactions. Qrr1-4 and QrrX are highlighted in red and orange, respectively. The arrows between sRNA circles indicate the order in which the sRNAs appear in the chimeric transcript, pointing from ones located at the 5' end towards ones at the 3' end. The size of the circles correlates with the number of chimeric reads attributed to the sRNA.

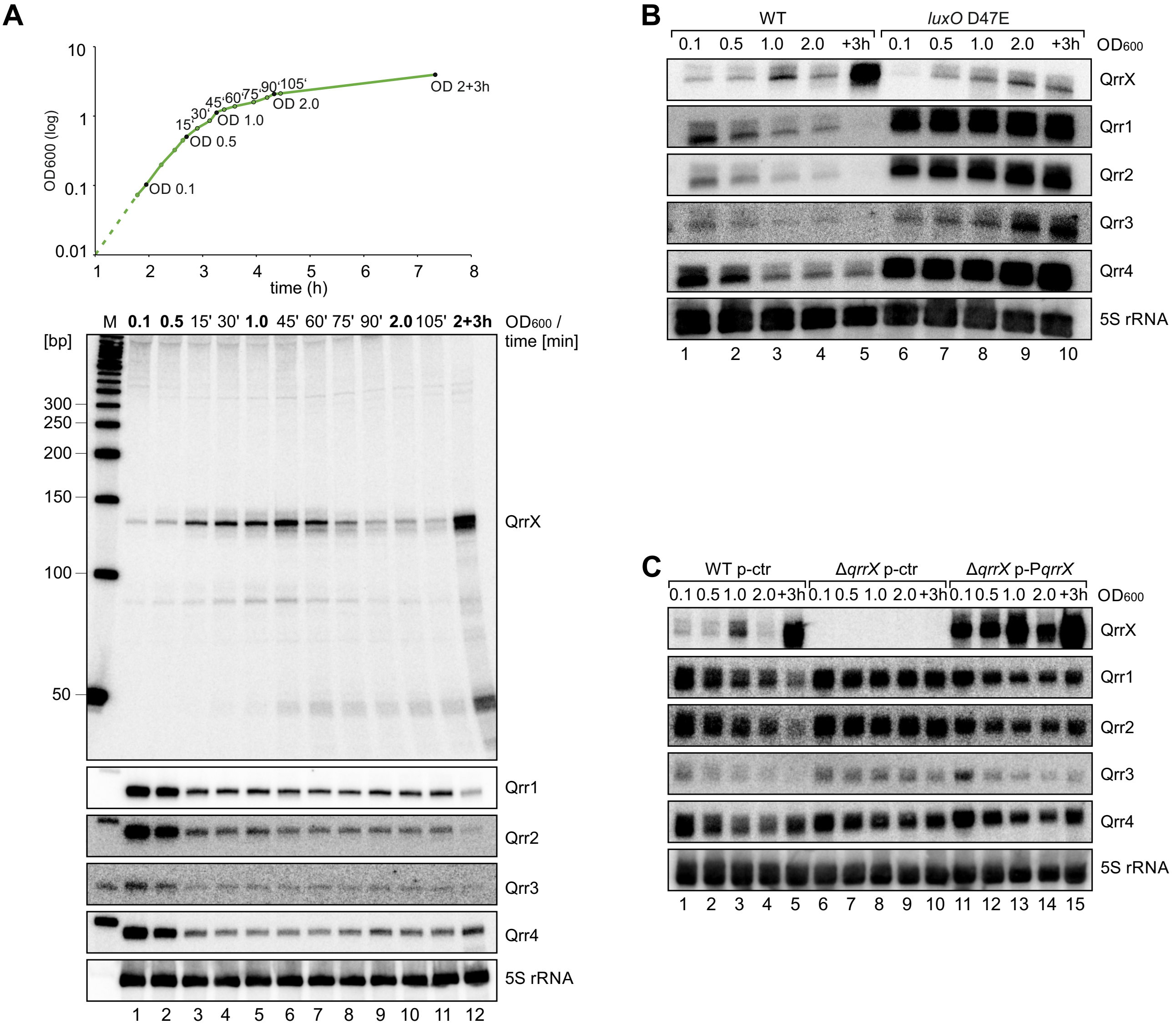

A
10
1
0.1
0.01
OD600 (log)
1 2 3 4 5 6 7 8
time (h)
OD 0.1
OD 0.5
15' 30'
OD 1.0
45' 60' 75' 90' 105'
OD 2.0
OD 2+3h
M 0.1 0.5 15' 30' 1.0 45' 60' 75' 90' 2.0 105' 2+3h
OD600 / time [min]
[bp]
300
250
200
150
100
50
QrrX
Qrr1
Qrr2
Qrr3
Qrr4
5S rRNA
1 2 3 4 5 6 7 8 9 10 11 12
B
WT
luxO D47E
0.1 0.5 1.0 2.0 +3h 0.1 0.5 1.0 2.0 +3h
OD600
QrrX
Qrr1
Qrr2
Qrr3
Qrr4
5S rRNA
1 2 3 4 5 6 7 8 9 10
C
WT p-ctr
ΔqrrX p-ctr
ΔqrrX p-PqrrX
0.1 0.5 1.0 2.0 +3h 0.1 0.5 1.0 2.0 +3h 0.1 0.5 1.0 2.0 +3h
OD600
QrrX
Qrr1
Qrr2
Qrr3
Qrr4
5S rRNA
1 2 3 4 5 6 7 8 9 10 11 12 13 14 15

Figure S4: Expression patterns of QrrX and the Qrr1-4 sRNAs

(A) *V. cholerae* wild-type cells were cultivated in LB medium and RNA samples were collected at various stages of growth and time points as indicated. Northern blot analysis with specific oligonucleotide probes served to monitor QrrX, Qrr1, Qrr2, Qrr3 and Qrr4 RNA levels. Probing for 5S ribosomal RNA served as loading control. The experiment was performed with three independent biological replicates (*n* = 3).

(B) *V. cholerae* wild-type and *luxO* D47E cells were cultivated in LB medium and RNA samples were collected at the indicated OD_{600} readings. Northern blot analysis with specific oligonucleotide probes served to monitor QrrX, Qrr1, Qrr2, Qrr3 and Qrr4 RNA levels. Probing for 5S ribosomal RNA served as loading control. The experiment was performed with two independent biological replicates (*n* = 2).

(C) *V. cholerae* wild-type and Δ*qrrX* cells carrying either a control plasmid (p-ctr) or a complementation plasmid where *qrrX* is expressed from its native promotor (p-P*qrrX*) were cultivated in LB medium, and RNA samples were collected at the indicated OD_{600} readings. Northern blot analysis with specific oligonucleotide probes served to monitor QrrX, Qrr1, Qrr2, Qrr3 and Qrr4 RNA levels. Probing for 5S ribosomal RNA served as loading control. The experiment was performed with three independent biological replicates (*n* = 3). Source data underlying panels **(A)-(C)** are provided as a Source Data file.

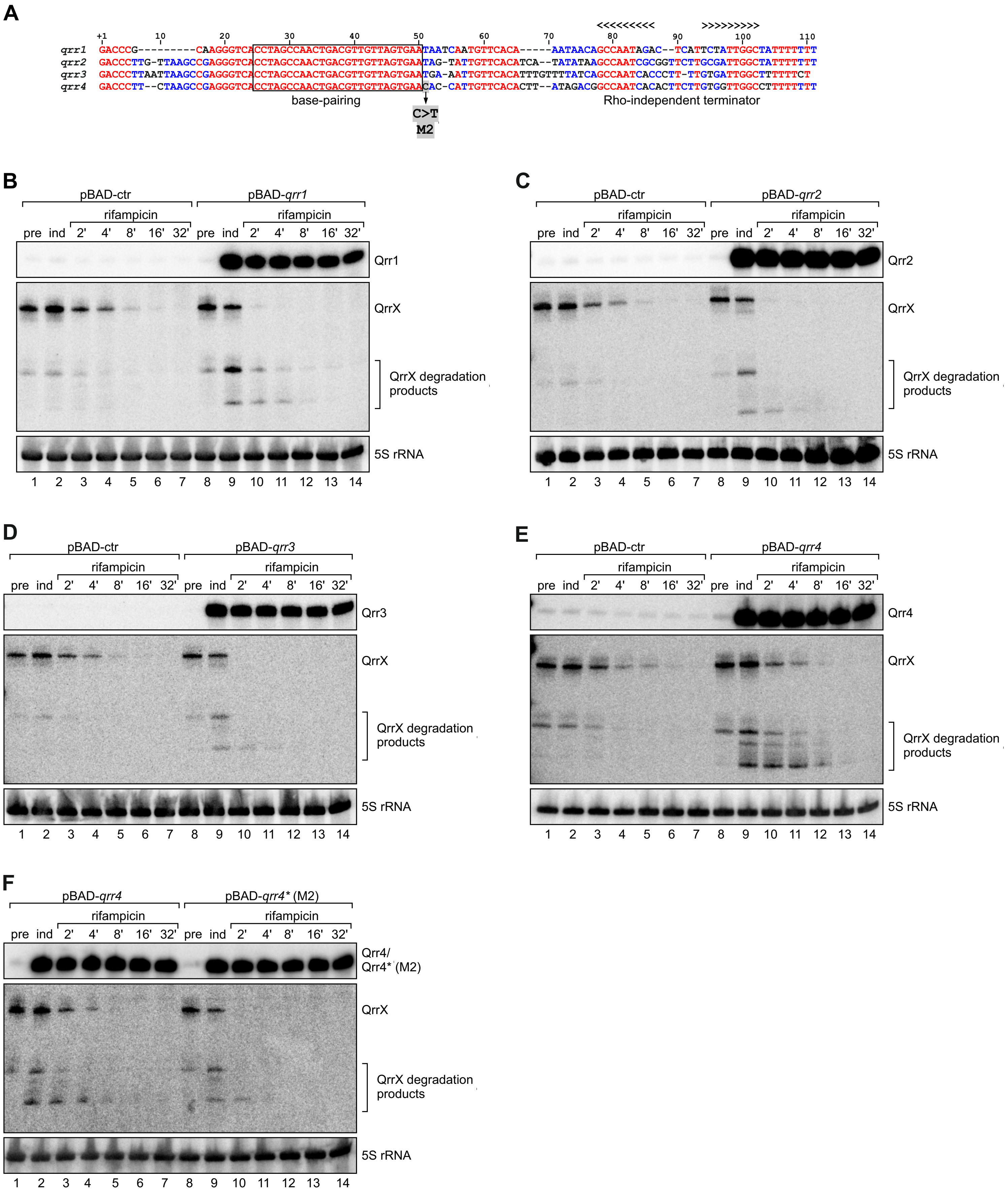
A
+1 10 20 30 40 50 60 70 80 90 100 110
qrr1 GACCCG---------CAAGGGTCACCTAGCCAACTGACGTTGTTAGTGAATAATCAATGTTCACA-----AATAACAGCCAATAGAC--TCATTCTATTGGCTATTTTTTT
qrr2 GACCCTTG-TTAAGCCGAGGGTCACCTAGCCAACTGACGTTGTTAGTGAATAG-TATTGTTCACATCA--TATATAAGCCAATCGCGGTTCTTGCGATTGGCTATTTTTTT
qrr3 GACCCTTAATTAAGCCGAGGGTCACCTAGCCAACTGACGTTGTTAGTGAATGA-AATTGTTCACATTTGTTTTATCAGCCAATCACCCTT-TTGTGATTGGCTTTTTTCT
qrr4 GACCCTT--CTAAGCCGAGGGTCACCTAGCCAACTGACGTTGTTAGTGAACAC-CATTGTTCACACTT--ATAGACGGCCAATCACACTTCTTGTGGTTGGCCTTTTTTT
base-pairing
Rho-independent terminator
C>T
M2
B
pBAD-ctr
pBAD-qrr1
rifampicin
pre ind 2' 4' 8' 16' 32' pre ind 2' 4' 8' 16' 32'
Qrr1
QrrX
QrrX degradation products
5S rRNA
1 2 3 4 5 6 7 8 9 10 11 12 13 14
C
pBAD-ctr
pBAD-qrr2
rifampicin
pre ind 2' 4' 8' 16' 32' pre ind 2' 4' 8' 16' 32'
Qrr2
QrrX
QrrX degradation products
5S rRNA
1 2 3 4 5 6 7 8 9 10 11 12 13 14
D
pBAD-ctr
pBAD-qrr3
rifampicin
pre ind 2' 4' 8' 16' 32' pre ind 2' 4' 8' 16' 32'
Qrr3
QrrX
QrrX degradation products
5S rRNA
1 2 3 4 5 6 7 8 9 10 11 12 13 14
E
pBAD-ctr
pBAD-qrr4
rifampicin
pre ind 2' 4' 8' 16' 32' pre ind 2' 4' 8' 16' 32'
Qrr4
QrrX
QrrX degradation products
5S rRNA
1 2 3 4 5 6 7 8 9 10 11 12 13 14
F
pBAD-qrr4
pBAD-qrr4* (M2)
rifampicin
pre ind 2' 4' 8' 16' 32' pre ind 2' 4' 8' 16' 32'
Qrr4/
Qrr4* (M2)
QrrX
QrrX degradation products
5S rRNA
1 2 3 4 5 6 7 8 9 10 11 12 13 14

Figure S5: Stability of QrrX upon induction of Qrr1-4

(A) Alignment of *qrr1-4*. The sequences of *qrr1*, *qrr2*, *qrr3* and *qrr4* were aligned using the Multalin tool (2). The predicted region of base-pairing identical in all four homologs is marked with a black box, the M2 mutation in *qrr4* is marked in gray. The Rho-independent terminator is indicated.

(B-E) *V. cholerae* wild-type cells harboring either pBAD-*qrr1* (B), pBAD-*qrr2* (C), pBAD-*qrr3* (D), pBAD-*qrr4* (E) or an empty control plasmid (pBAD-ctr) were cultivated in LB medium to OD_{600} of 1.0. Expression of the Qrr1-4 sRNAs was induced with L-arabinose and rifampicin was added to monitor RNA stability. Northern blot analysis shows QrrX and Qrr1-4 levels at the indicated time points. 5S ribosomal RNA was used as loading control. The experiment was performed with two independent biological replicates (*n* = 2).

(F) *V. cholerae* wild-type cells harboring either pBAD-*qrr4* or pBAD-*qrr4** (M2) (indicated in (A)) were cultivated in LB medium to OD_{600} of 1.0. Expression of Qrr4 or Qrr4* was induced with L-arabinose and rifampicin was added to monitor RNA stability. Northern blot analysis shows QrrX and Qrr4/Qrr4* levels at the indicated time points. 5S ribosomal RNA was used as loading control. The experiment was performed with three independent biological replicates (*n*=3). Source data underlying panels **(B)-(F)** are provided as a Source Data file.

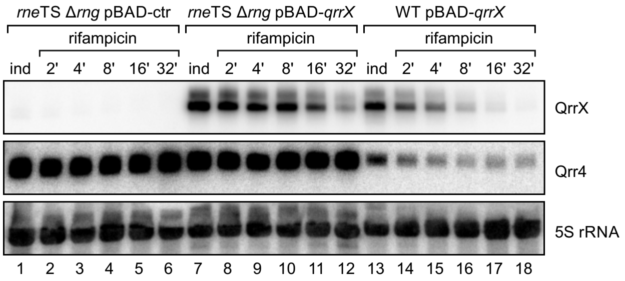

Figure S6: Influence of RNase E and RNase G on QrrX - Qrr4 interaction

V. cholerae wild-type and *rne*^{TS} Δ*rng* cells carrying either pBAD-ctr or pBAD-*qrrX* were cultivated at 30°C to OD_{600} of 0.2 and then shifted to 44°C. After 15 min, expression of QrrX was induced with L-arabinose and rifampicin was added to monitor RNA stability. Northern blot analysis shows QrrX and Qrr4 levels at the indicated time points. 5S ribosomal RNA was used as loading control. The experiment was performed with three independent biological replicates (*n* = 3). Source data underlying Figure S6 is provided as a Source Data file.

Figure S7

Huber *et al.*, 2022

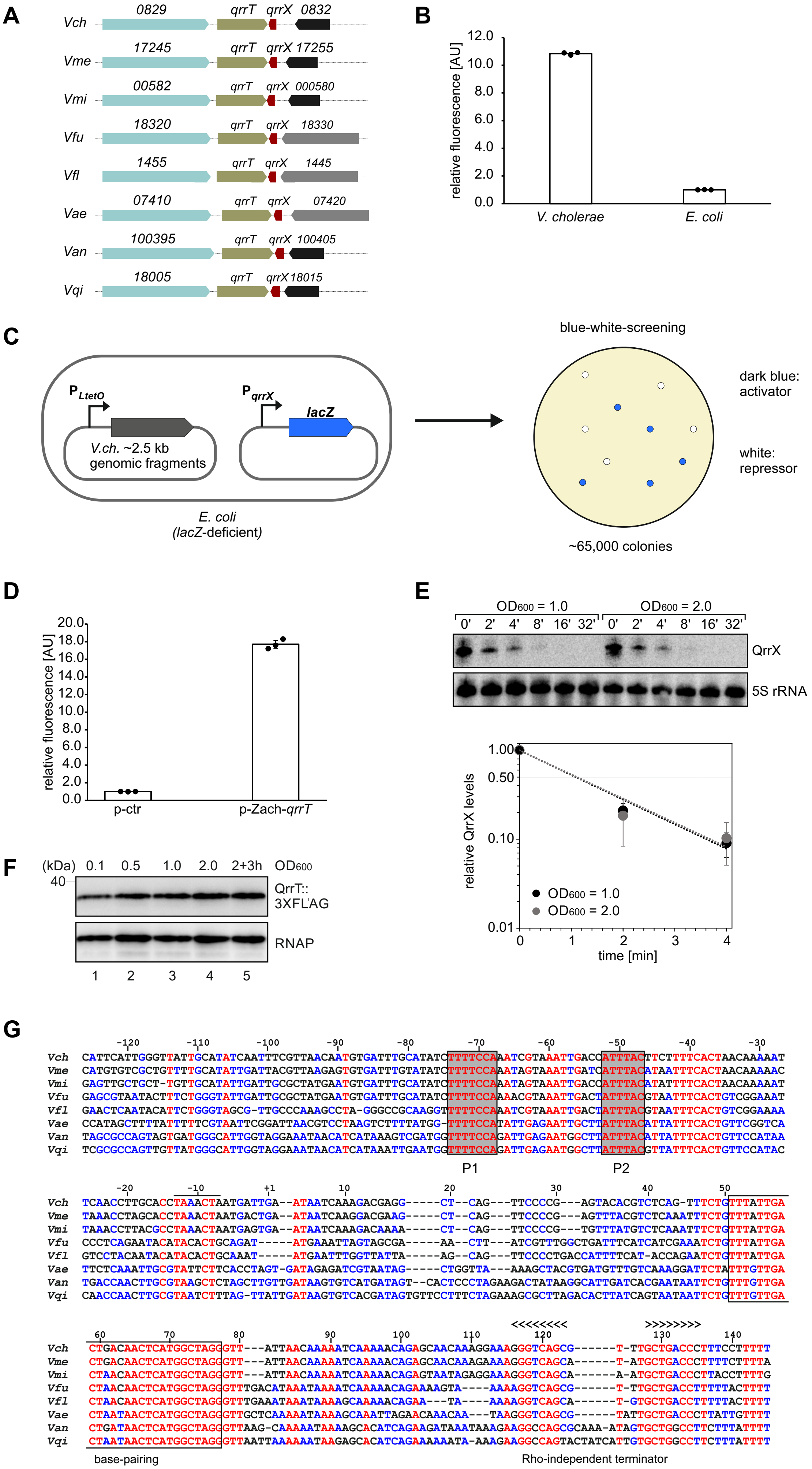

Figure S7: Transcriptional regulation of QrrX

(A) Gene synteny analysis of the genomic loci encoding the *qrrX* sRNA in various *Vibrio* species. Homologous genes are shown in the same colors. *Vch*, *Vibrio cholerae; Vme, Vibrio metoecus, Vmi, Vibrio mimicus; Vfu, Vibrio furnissii; Vfl, Vibrio fluvialis; Vae, Vibrio aestuarianus; Van, Vibrio anguillarum, Vqi, Vibrio qinghaiensis.*

(B) Relative P_{qrrX} promotor activity. *V. cholerae* and *E. coli* Top10 cells carrying an mKate2-based transcriptional reporter for *qrrX* (P_{qrrX}*::mKate2*) were cultivated in LB medium and relative fluorescence was determined. Data are presented as mean values of biological independent replicates ±SD, *n*=3.

(C) Schematic scheme visualizing the experimental procedure to identify factors regulating the transcription of *qrrX*.

(D) Relative P_{qrrX} promotor activity. *E. coli* Top10 cells carrying an mKate2-based transcriptional reporter for *qrrX* (P_{qrrX}*::mKate2*) and an empty control plasmid (p-ctr) or a pZach library plasmid containing the full *qrrT* gene (pZach-*qrrT*) were cultivated in LB medium and relative fluorescence was determined. Data are presented as mean values of biological independent replicates ±SD, *n*=3.

(E) Stability of QrrX. *V. cholerae* wild-type cells were cultivated in LB medium to OD_{600} = 1.0 and OD_{600} = 2.0, and rifampicin was added to determine RNA stability. Northern blot analysis shows QrrX levels at the indicated time points. 5S ribosomal RNA was used as loading control. Graph shows quantification of three independent biological replicates. QrrX levels at 0 min were set to 1.

(F) Expression of QrrT. *V. cholerae qrrT::3XFLAG* cells were cultivated in LB medium and protein samples were collected at various stages of growth. Western blot analysis was performed to determine QrrT protein levels. RNAP served as loading control. The experiment was performed with three independent biological replicates (*n* = 3).

(G) Alignment of *qrrX* sequences including promotor region. Conserved *qrrX* promoter elements P1 and P2 are marked with gray boxes. Numbers above the sequences indicate the distance to the transcriptional start site. *Vch*, *Vibrio cholerae; Vme, Vibrio metoecus, Vmi, Vibrio mimicus; Vfu, Vibrio furnissii; Vfl, Vibrio fluvialis; Vae, Vibrio aestuarianus; Van, Vibrio anguillarum, Vqi, Vibrio qinghaiensis.* Source data underlying panels **(B)**, **(D)**, **(E)** and **(F)** are provided as a Source Data file.

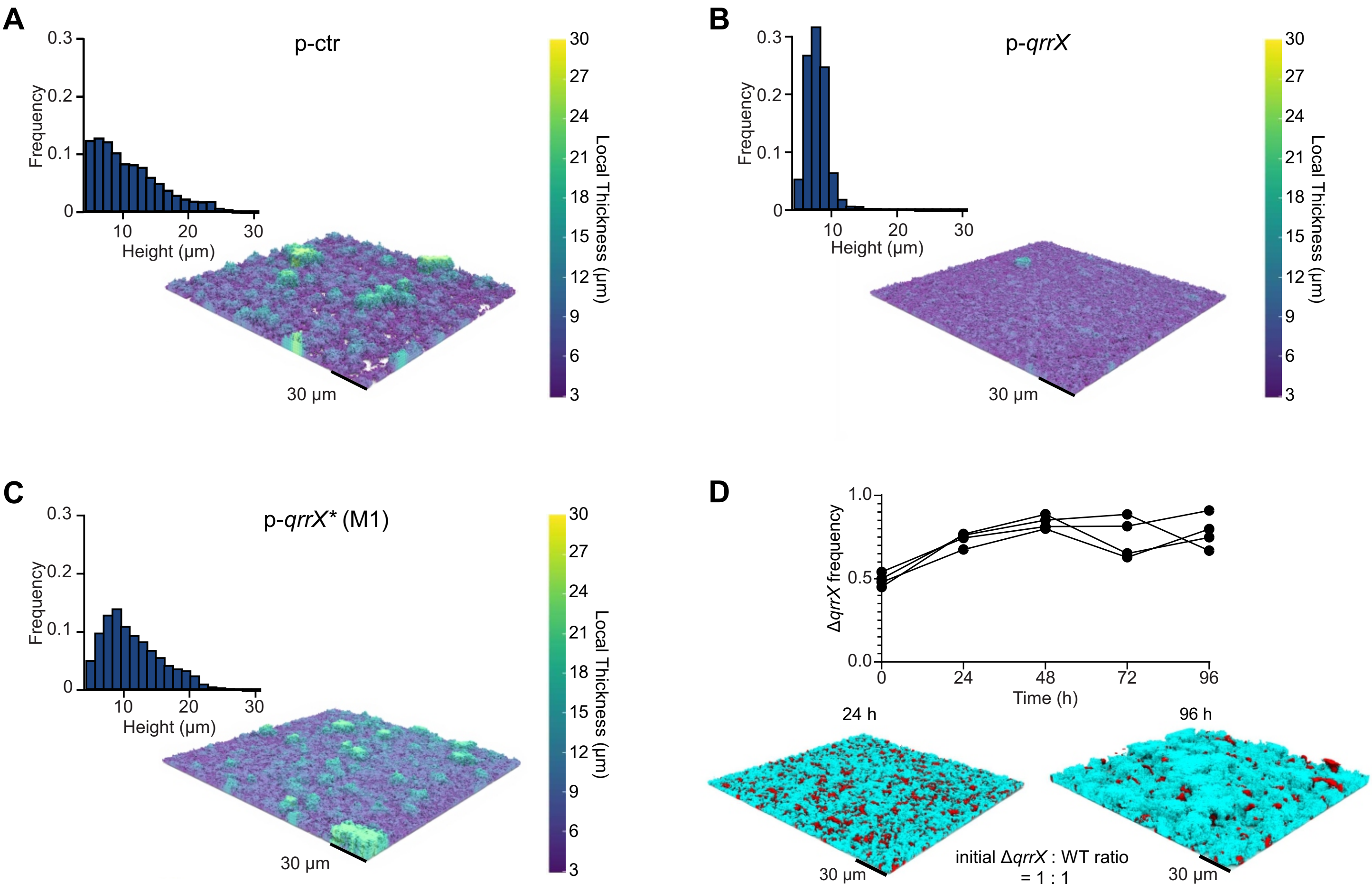

Figure S8: Influence of QrrX on biofilm accumulation

(A-C) Biofilms of *V. cholerae* wild-type cells harboring either p-ctr (A), p-*qrrX* (B) or p-*qrrX** (M1) (C) were grown for 48 h and imaged through their whole depth by confocal microscopy. 3D renderings of representative images are color-coded by local thickness. Bar graphs show frequency distributions of local height for each biofilm.

(D) Mixed biofilm of *V. cholerae* wild-type and Δ*qrrX* cells seeded at an initial ratio of 1:1 was grown for 96 h. Line graph shows the trajectory of each replicate (n = 4). Representative images are shown for 24 h and 96 h of growth (WT = red, Δ*qrrX* = cyan). Source data underlying panels **(A)-(D)** are provided as a Source Data file.

Table S1: Previously published sRNA targets captured in chimeras in RIL-seq**.** Numbers shown in parentheses are below our threshold of 20 chimeras. Altogether, this results in 40 interactions found in our data set, of which 35 are found in at least one condition with a number of chimeras above our threshold.

**sRNA**	**target**	**# chimeras LCD**	**# chimeras HCD**	**reference**
CarZ	*vc2390* (*carA*)	[6]	[7]	(13)
FarS	*vc1740* (*fadE*)	149	126	(14)
MicV	*vc1854* (*ompT*)	[8]	25	(6)
MicV	*vc0156* (*btuB*)	47	---	(6)
MicV	*vc1091 (oppA)*	78	---	(6)
MicX	*vc0972*	104380	20332	(15)
MicX	*vc0620*	1925	[5]	(15)
OppZ	*vc1092* (*oppB*)	22	126	(13)
Qrr1	*vc0583* (*hapR*)	280	355	(16)
Qrr1	*vca0939*	[8]	[4]	(17)
Qrr2	*vc2647* (*aphA*)	14500	582	(18, 19)
Qrr2	*vc0583* (*hapR*)	65	72	(16)
Qrr2	*vca0939*	[13]	[8]	(17)
Qrr3	*vc2647* (*aphA*)	5242	915	(18, 19)
Qrr3	*vc0583* (*hapR*)	1400	2082	(16)
Qrr3	*vca0939*	25	25	(17)
Qrr4	*vc2647* (*aphA*)	6133	189	(18, 19)
Qrr4	*vc0583* (*hapR*)	66	32	(16)
TfoR	*vc1153* (*tfoX*)	895	86	(20)
TfoR	*vc0534* (*rpoS*)	143	25	(21)
VadR	*vc1264* (*irpA*)	[14]	---	(22)
VadR	*vc0916* (*vpsU*)	42	---	(22)
VadR	*vc2352*	557	70	(22)
VcdRP	*vc2013* (*ptsG*)	706	150	(23)
VcdRP	*vc0995* (*nagE*)	24	[9]	(23)
VcdRP	*vc0966* (*ptsH*)	361	31	(23)
Vcr043	*vc0534* (*rpoS*)	---	74	(21)
VqmR	*vca0068*	96	19	(4)
VqmR	*vc0200*	---	[6]	(4)
VqmR	*vca0591*	95	93	(4)
VqmR	*vc1865*	6567	181	(4)
VqmR	*vc1063*	31	[13]	(4)
VqmR	*vca0917*	71	141	(4)
VqmR	*vc2647* (*aphA*)	---	336	(8)
VrrA	*vc2213* (*ompA*)	782	94	(24)
VrrA	*vc1854* (*ompT*)	[10]	194	(25)
VrrA	*vc2467 (rpoE)*	83	---	(6)
VrrA	*vca0059* (*lpp*)	3300	4396	(6)
VrrA	*vca1835 (pal)*	94	[15]	(6)
VrrA	*vc0429*	26	31	(6)

Table S2: Plasmids used in this study

Plasmid trivial name	Plasmid Stock name	Relevant fragment	Comment	Origin, marker	Reference
**Plasmids for RIL-seq target validation (GFP reporter plasmids)**					
pXG10-sfGFP	pXG10-sfGFP	lacZ'::sfGFP	Template plasmid for translational reporter	pSC101*, Cm^R	(3)
pXG30-sfGFP	pXG30-sfGFP	FLAG::lacZ'::sfGFP	Template plasmid for translational reporter (for operons)	pSC101*, Cm^R	(3)
pXG10-*vc0395*	pMH063	5' UTR + 20 aa of *vc0395*	Translational GFP reporter	pSC101*, Cm^R	This study
pXG10-*vc0122* (*cyaA*)	pJR026	5' UTR + 20 aa of *vc0122*	Translational GFP reporter	pSC101*, Cm^R	This study
pXG10-*vc2478* (*zapA*)	pJR039	5' UTR + 20 aa of *vc2478*	Translational GFP reporter	pSC101*, Cm^R	This study
pXG30-*vc2088-vc2087* (*sucA*)	pKT003	3' part of *vc2088* + IGR + 20 aa of *vc2087*	Translational GFP reporter	pSC101*, Cm^R	This study
pXG10-1C-*vc0910*	pNP058	5' UTR + 15 aa of *vc0910*	Translational GFP reporter	pSC101*, Cm^R	This study
pXG10-*vc1905*	pKT006	5' UTR + 20 aa of *vc1905*	Translational GFP reporter	pSC101*, Cm^R	This study
pXG10-*vc1492*	pKT001	5' UTR + 20 aa of *vc1492*	Translational GFP reporter	pSC101*, Cm^R	This study
pXG10-*vc2091* (*sdhC*)	pMH073	5' UTR + 20 aa of *vc2091*	Translational GFP reporter	pSC101*, Cm^R	This study
pXG10-*vca0987*	pJR040	5' UTR + 20 aa of *vca0987*	Translational GFP reporter	pSC101*, Cm^R	This study
pXG30-*vc1596-vc1595* (*galK*)	pMH066	3' part of *vc1596* + IGR +20 aa of *vc1595*	Translational GFP reporter	pSC101*, Cm^R	This study
pXG30- *vc1336-vc1337*	pMH072	3' part of *vc1336* + IGR + 20 aa of *vc1337*	Translational GFP reporter	pSC101*, Cm^R	This study
pXG10-*vca0036*	pMH093	5' UTR + 20 aa of *vca0036*	Translational GFP reporter	pSC101*, Cm^R	This study
pXG10-*vc2647* (*aphA*)	pKP462	5' UTR + 20 aa of *vc2647*	Translational GFP reporter	pSC101*, Cm^R	(8)
pXG10-*vc1992*	pSM001	5' UTR + 20 aa of *vc1992*	Translational GFP reporter	pSC101*, Cm^R	This study
pXG10-*vc0648*	pKT008	5' UTR + 20 aa of *vc0648*	Translational GFP reporter	pSC101*, Cm^R	This study
pXG10-*vc1904*	pKT005	5' UTR + 20 aa of *vc1904*	Translational GFP reporter	pSC101*, Cm^R	This study
pXG10-*vc1091* (*oppA*)	pMD092	5' UTR + 20 aa of *vc1091*	Translational GFP reporter	pSC101*, Cm^R	This study
pXG10-*vc0391*	pKT007	5' UTR + 20 aa of *vc0391*	Translational GFP reporter	pSC101*, Cm^R	This study
pXG10-*vc0567*	pYH034	5' UTR + 15 aa of *vc0567*	Translational GFP reporter	pSC101*, Cm^R	This study
pXG10-1C-*vca0026*	pYH038	5' UTR + 15 aa of *vca0026*	Translational GFP reporter	pSC101*, Cm^R	This study

pXG10-1C-*vc0633* (*ompU*)	pNP085	5' UTR + 30 aa of *vc0633*	Translational GFP reporter	pSC101*, Cm^R	(6)
pXG10-*vc1449* (*rtxC*)	pKP353	5' UTR + 24 aa of *vc1449*	Translational GFP reporter	pSC101*, Cm^R	(4)
pXG10-*vc0470*	pMH067	5' UTR + 20 aa of *vc0470*	Translational GFP reporter	pSC101*, Cm^R	This study
pXG10-*vc0260* (*rfbU*)	pYH033	5' UTR + 15 aa of *vc0260*	Translational GFP reporter	pSC101*, Cm^R	This study
pXG10-*vc1442*	pMH060	5' UTR + 20 aa of *vc1442*	Translational GFP reporter	pSC101*, Cm^R	This study
pXG10-*vc0036*	pJR029	5' UTR + 20 aa of *vc0036*	Translational GFP reporter	pSC101*, Cm^R	This study
pXG10-*vc0549*	pMH071	5' UTR + 20 aa of *vc0549*	Translational GFP reporter	pSC101*, Cm^R	This study
pXG10-*vc2469*	pJR044	5' UTR + 20 aa of *vc2469*	Translational GFP reporter	pSC101*, Cm^R	This study
pXG10-*vc0911*	pJR043	5' UTR + 20 aa of *vc0911*	Translational GFP reporter	pSC101*, Cm^R	This study
pXG10-*vc2217*	pJR042	5' UTR + 20 aa of *vc2217*	Translational GFP reporter	pSC101*, Cm^R	This study
pXG10-*vc1146* (*grxA*)	pMH059	5' UTR + 20 aa of *vc1146*	Translational GFP reporter	pSC101*, Cm^R	This study
pXG10-*vc1051*	pMH056	5' UTR + 20 aa of *vc1051*	Translational GFP reporter	pSC101*, Cm^R	This study
pXG10-*vc2030* (*rne*)	pJR045	5' UTR + 20 aa of *vc2030*	Translational GFP reporter	pSC101*, Cm^R	This study
pXG30-*vc0147-vc0148*	pKT004	3' part of *vc0147* + IGR + 20 aa of *vc0148*	Translational GFP reporter	pSC101*, Cm^R	This study
pXG10-*vc2013* (*ptsG*)	pMD161	5' UTR + 20 aa of *vc2013*	Translational GFP reporter	pSC101*, Cm^R	This study
pXG10-*vca0166*	pJR036	5' UTR + 20 aa of *vca0166*	Translational GFP reporter	pSC101*, Cm^R	This study
pXG10-*vc0966*	pMH062	5' UTR + 20 aa of *vc0966*	Translational GFP reporter	pSC101*, Cm^R	This study
**Plasmids for RIL-seq target validation (sRNA expression plasmids)**					
pCMW-1	pCMW-1		Control plasmid	p15A, Kan^R	(10)
pEVS143	pEVS143	P*tac* promotor	Constitutive overexpression plasmid (template)	p15A, Kan^R	(7)
p-*qrr1*	pAL030	*qrr1*	*qrr1* expression plasmid	p15A, Kan^R	This study
p-*qrr2*	pAL032	*qrr2*	*qrr2* expression plasmid	p15A, Kan^R	This study
p-*qrr3*	pAL031	*qrr3*	*qrr3* expression plasmid	p15A, Kan^R	This study
p-*qrr4*	pRH003	*qrr4*	*qrr4* expression plasmid	p15A, Kan^R	(23)
p-*spot 42*	pAS001	*spot 42*	*spot 42* expression plasmid	p15A, Kan^R	(23)
p-*gcvB*	pRH006	*gcvB*	*gcvB* expression plasmid	p15A, Kan^R	(23)
p-*tfoR*	pMD104	*tfoR*	*tfoR* expression plasmid	p15A, Kan^R	(23)

p-*vcr001*	pMH057	*vcr001*	*vcr001* expression plasmid	p15A, Kan^R	This study
p-*vcr017*	pSG001	*vcr017*	*vcr017* expression plasmid	p15A, Kan^R	(22)
p-*vcr043*	pNP004	*vcr043*	*vcr043* expression plasmid	p15A, Kan^R	(23)
p-*vcr227*	pJR035	*vcr227*	*vcr227* expression plasmid	p15A, Kan^R	This study
**Other plasmids**					
pBAD1K-ctr	pMD004		Control plasmid	p15A, Kan^R	(13)
pBAD1K-*qrrX*	pMD099	*qrrX*	Inducible *qrrX* expression plasmid	p15A, Kan^R	This study
pBAD1K-*qrrX** (M1)	pMD103	*qrrX** (G72C)	Inducible *qrrX** (M1) expression plasmid	p15A, Kan^R	This study
pBAD1K-*qrr4*	pMD176	*qrr4*	Inducible *qrr4* expression plasmid	p15A, Kan^R	This study
pBAD1K-*qrr4** (M1)	pMH086	*qrr4** (C28G)	Inducible *qrr4** (M1) expression plasmid	p15A, Kan^R	This study
pBAD1K-*qrr1*	pMH088	*qrr1*	Inducible *qrr1* expression plasmid	p15A, Kan^R	This study
pBAD1K-*qrr1** (M1)	pAL026	*qrr1** (C21G)	Inducible *qrr1** (M1) expression plasmid	p15A, Kan^R	This study
pBAD1K-*qrr2*	pMH092	*qrr2*	Inducible *qrr2* expression plasmid	p15A, Kan^R	This study
pBAD1K-*qrr3*	pMH090	*qrr3*	Inducible *qrr3* expression plasmid	p15A, Kan^R	This study
pBAD1K-*qrr4** (M2)	pAL027	*qrr4** (C49T)	Inducible *qrr4** (M2) expression plasmid	p15A, Kan^R	This study
pCMW-1C-*mKate2*	pYH010	*mKate2*	Promoterless plasmid for transcriptional reporters	p15A, Cm^R	(8)
pCMW-1C-P_{qrrX}::*mKate2*	pAF012	P_{qrrX}::*mKate2*	Transcriptional reporter for QrrX	p15A, Cm^R	This study
pCMW-1C-P_{qrrX}::*mKate2* ΔP1	pMH083	P_{qrrX}::*mKate2* ΔP1	Transcriptional reporter for QrrX ΔP1	p15A, Cm^R	This study
pCMW-1C-P_{qrrX}::*mKate2* ΔP1	pMH085	P_{qrrX}::*mKate2* ΔP1	Transcriptional reporter for QrrX ΔP1	p15A, Cm^R	This study
pKAS32	pKAS32		Suicide plasmid for allelic exchange	R6K, Amp^R	(9)
pKAS32-Δ*rng*	pJR024	up/downstream flanks of *rng*	Suicide plasmid for *rng* knock-out	R6K, Amp^R	This study
pKAS32-Δ*qrrX*	pAS005	up/downstream flanks of *qrrX*	Suicide plasmid for *qrrX* knock-out	R6K, Amp^R	This study

pKAS32-Δ*qrrT*	pAF013	up/downstream flanks of *qrrT*	Suicide plasmid for *qrrT* knock-out	R6K, Amp^R	This study
pKAS32-*qrrT::3XFLAG*	pMH075	*qrrT::3XFLAG*	Suicide plasmid *qrrT::3XFLAG* allelic replacement	R6K, Amp^R	This study
pKAS32-Δ*qrrT* Δ*qrrX*	pAL033	up/downstream flanks of *qrrT-qrrX*	Suicide plasmid for *qrrT-qrrX* knock-out	R6K, Amp^R	This study
pKAS32-*hapR::3XFLAG*	pASp017	*hapR::3XFLAG*	Suicide plasmid *hapR::3XFLAG* allelic replacement	R6K, Amp^R	This study
pKAS32-*qrrX** M1	pMH091	up/downstream flanks of *qrrX** M1	Suicide plasmid *qrrX** (G72C) allelic replacement	R6K, Amp^R	This study
pKAS32-*qrr1** M1	pAL035	up/downstream flanks of *qrr1** M1	Suicide plasmid *qrr1** (C21G) allelic replacement	R6K, Amp^R	This study
pKAS32-*qrr4** M1	pAL036	up/downstream flanks of *qrr4** M1	Suicide plasmid *qrr4** (C28G) allelic replacement	R6K, Amp^R	This study
pBB1	---	*luxCDABE* (*V. harveyi*)	Bioluminescence assay	Tet^R	(26)
pEVS-protein	pMD080	P*tac* promotor, 5'UTR, MCS and T1 terminator	Protein expression plasmid	p15A, Kan^R	(23)
p-*qrrT*	pAL001	*qrrT*	*qrrT* expression plasmid	p15A, Kan^R	This study
pCMW-2	pLH001		Control plasmid	p15A, Kan^R	(13)
p-P*qrrX*	pLH002	*qrrX*	*qrrX* expression plasmid with native promotor	p15A, Kan^R	This study
pBBR1-MCS5-*lacZ*	pBBR1MCS	*lacZ*	Promotorless plasmid for transcriptional reporters	pBBR1, $Gent^R$	(11)
pBBR1-P_{qrrX}-*lacZ*	pMD285	P_{qrrX}*::lacZ*	Transcriptional reporter for QrrX	pBBR1, $Gent^R$	This study
pZach	pZND132	*V.ch.* genomic fragments	Genomic fragment expression plasmid	p15A, Cm^R	(12)
pZach-*qrrT*	---	*qrrT*	*qrrT* fragment expression plasmid	p15A, Cm^R	(12)
p-*qrrX*	pNP012	*qrrX*	*qrrX* expression plasmid	p15A, Kan^R	(23)
p-*qrrX** (M1)	pAF015	*qrrX** (G72C)	*qrrX* (G72C) expression plasmid	p15A, Kan^R	This study

Table S3: Strains used in this study

Strain	Relevant markers / Genotype	Reference / Source
V. cholerae		
KPS-0014	C6706 wild-type	(27)
KPS-0023	C6706 *luxO* D47E	(28)
KPS-0358	C6706 Δ*qrr1-4*	(28)
KPS-0995	C6706 *hfq::3XFLAG*	(6)
KPVC-10141	C6706 *rne*^{TS}	(13)
KPVC-11271	C6706 Δ*qrr1-4 aphA::3XFLAG*	This study
KPVC-11952	C6706 Δ*rng*	This study
KPVC-11971	C6706 *rne*^{TS} Δ*rng*	This study
KPVC-12032	C6706 Δ*qrrT*	This study
KPVC-12548	C6706 *qrrT::3XFLAG*	This study
KPVC-12735	C6706 Δ*qrrX*	This study
KPVC-12778	C6706 Δ*qrrX aphA::3XFLAG*	This study
KPVC-12901	C6706 *aphA::3XFLAG*	This study
KPVC-12904	C6706 Δ*qrrT hapR::3XFLAG*	This study
KPVC-12925	C6706 *hapR::3XFLAG*	This study
KPVC-13690	C6706 Δ*qrr1-4 hapR::3XFLAG*	This study
KPVC-13859	C6706 Δ*qrrX hapR::3XFLAG*	This study
KPVC-14437	C6706 Δ*qrrT* Δ*qrrX aphA::3XFLAG*	This study
KPVC-14438	C6706 Δ*qrrT* Δ*qrrX hapR::3XFLAG*	This study
KPVC-14462	C6706 *qrrX** (M1)	This study
KPVC-14473	C6706 *qrr1** (M1)	This study
KPVC-14472	C6706 *qrr4** (M1)	This study
CNV141	C6706 Ptac-sfGFP at *lacZ* site	Laboratory stock
CNV175	C6706 Ptac-Mruby3 at *lacZ* site	Laboratory stock
CNV259	C6706 Δ*qrrX* Ptac-sfGFP at *lacZ* site	This study
E. coli		
Top10	*F- mcrA Δ(mrr-hsdRMS-mcrBC) φ80lacZΔM15 ΔlacX74 nupG recA1 araD139 Δ(ara-leu)7697 galE15 galK16 rpsL(StrR) endA1 λ-*	Invitrogen
S17λpir	*ΔlacU169 (ΦlacZΔM15), recA1, endA1, hsdR17, thi-1, gyrA96, relA1, λpir*	New England Biolabs

Table S4: Oligonucleotides used in this study

Name	Sequence 5' to 3'	Description
**Oligonucleotides for linearization of plasmids**		
KPO-0092	CCACACATTATACGAGCCGA	Plasmid construction (pEVS143)
KPO-0196	GGAGAAACAGTAGAGAGTTGCG	Plasmid construction (pBAD1K)
KPO-0267	TAATAGGCCTAGGATGCATATG	Plasmid construction (pKAS32)
KPO-0268	CGTTAACAACCGGTACCTCTA	Plasmid construction (pKAS32)

KPO-1397	GATCCGGTGATTGATTGAGC	Plasmid construction (pEVS143 and pBAD1K)
KPO-1702	ATGCATGTGCTCAGTATCTCTATC	Plasmid construction (pXG10)
KPO-1703	GCTAGCGGATCCGCTGG	Plasmid construction (pXG10 and pXG30)
KPO-2591	GTCGACAGGCCTAGTTG	Plasmid construction (pCMW-1C-*mKate2*)
KPO-2592	GCATGCAAAAAGACCCTTC	Plasmid construction (pCMW-1C-*mKate2* and p-P$_{qrrX}$)
KPO-2757	TGAGGATCCGGTGATTGATTGAGCA	Plasmid construction (p-P$_{qrrX}$)
KPO-4646	CCATTCGCCATTCAGGCTG	Plasmid construction (pXG30)
pBAD-ATGrev	GGTTAATTCCTCCTGTTAGC	Plasmid construction (pEVS-prot.)
pZE-Stop-XbaI	TAATCTAGAGGCATCAAATAAAACGA	Plasmid construction (pEVS-prot.)
**Oligonucleotides for GFP reporter fusions (RIL-seq target validation)**		
KPO-1708	GAGATACTGAGCACATGCATAATTGATTTGGGACTGTTCCCAA	Plasmid construction (pNP058)
KPO-1709	GAGCCAGCGGATCCGCTAGCCAATTCGATAAGACGCGTCAC	Plasmid construction (pNP058)
KPO-2573	GAGATACTGAGCACATGCATAATCCCTGTCAGGTGTAAG	Plasmid construction (pMD092)
KPO-2574	GAGCCAGCGGATCCGCTAGCACCAGCACCTAACAGCAG	Plasmid construction (pMD092)
KPO-2779	GAGATACTGAGCACATGCAT CGGAAAATATAATGCAAAAAGTGG	Plasmid construction (pMD161)
KPO-2780	GAGCCAGCGGATCCGCTAGC GATTAAGTTATTAGAATTGCTGGG	Plasmid construction (pMD161)
KPO-3003	GTTTTTATGCATGATACTAATATAACGCATAACAATATC	Plasmid construction (pYH033)
KPO-3004	GTTTTTGCTAGCATGATGACGCTGTGCGCTTA	Plasmid construction (pYH033)
KPO-3005	GTTTTTATGCATGTACATGTGTAACCGATGGG	Plasmid construction (pYH034)
KPO-3006	GTTTTTGCTAGCAGCAATCCCCACTAGCAATC	Plasmid construction (pYH034)
KPO-3054	GTTTTTATGCATGCTCGTCACAGAACGAAATAC	Plasmid construction (pYH038)
KPO-3055	GTTTTTGCTAGCAAAACAGAGGGCAAGCAAACAAG	Plasmid construction (pYH038)
KPO-3795	GAGATACTGAGCACATGCATGTTTTGTTGGCATGGTCGC	Plasmid construction (pJR026)
KPO-3796	CCAGCGGATCCGCTAGCAATACGTTGCCGGTTTAGC	Plasmid construction (pJR026)
KPO-3813	GAGATACTGAGCACATGCATCTAAAAAACCATCAGTCCCC	Plasmid construction (pJR029)
KPO-3814	CCAGCGGATCCGCTAGCACTATCCTTTTCAGGCATTCG	Plasmid construction (pJR029)
KPO-4019	GAGATACTGAGCACATGCATGGCTCTTAGGTAAGAGTTGTT	Plasmid construction (pJR036)

KPO-4020	CCAGCGGATCCGCTAGCGAAACCAAAACCTTTAGTTTCG	Plasmid construction (pJR036)
KPO-4056	GAGATACTGAGCACATGCAT TTCTTCTGCGTTAAGCGCAA	Plasmid construction (pMH071)
KPO-4057	CCAGCGGATCCGCTAGCAGCCATTCCGGTGACCATC	Plasmid construction (pMH071)
KPO-4060	GAGATACTGAGCACATGCATACAAAATTAATGAGGCTACCTT	Plasmid construction (pMH056)
KPO-4061	CCAGCGGATCCGCTAGCTGCAACCACGCCTAGAAAG	Plasmid construction (pMH056)
KPO-4078	GAGATACTGAGCACATGCATCAAACAAAATGAAGGAGAAAGA	Plasmid construction (pMH059)
KPO-4079	CCAGCGGATCCGCTAGCATGCTCTTTGGCACGAACAC	Plasmid construction (pMH059)
KPO-4132	GAGATACTGAGCACATGCATATAATACGCATAATAAAACCCG	Plasmid construction (pJR040)
KPO-4133	CCAGCGGATCCGCTAGCCCAGAGGGTGTTCTTTTGC	Plasmid construction (pJR040)
KPO-4136	CCAGCGGATCCGCTAGCCATAGGGCTTCTCACTTTAAA	Plasmid construction (pMH066)
KPO-4137	GAGATACTGAGCACATGCATTCGATGGTCACCTGAGTTG	Plasmid construction (pJR039)
KPO-4138	CCAGCGGATCCGCTAGCTGGGCAGTTTACCCGGG	Plasmid construction (pJR039)
KPO-4184	GAGATACTGAGCACATGCATGAATTTTAGGTAAAGCCATTGG	Plasmid construction (pJR042)
KPO-4185	CCAGCGGATCCGCTAGCGAGTGCGCAGCCACTTAATG	Plasmid construction (pJR042)
KPO-4210	GAGATACTGAGCACATGCATAAAAAATTTCTCCCTAGCACAC	Plasmid construction (pMH063)
KPO-4211	CCAGCGGATCCGCTAGCCGCTGGTAGGAATCGGGTA	Plasmid construction (pMH063)
KPO-4212	GAGATACTGAGCACATGCAT GTTTTATTTTTAGCTTCAAAATAAAG	Plasmid construction (pMH062)
KPO-4213	CCAGCGGATCCGCTAGCTGCAGCAGGACGAGTGTG	Plasmid construction (pMH062)
KPO-4276	GAGATACTGAGCACATGCAT GAAAATGCCACTTTTTCATAAAGTC	Plasmid construction (pMH060)
KPO-4277	CCAGCGGATCCGCTAGCTTGGCGGACCACGGTATAG	Plasmid construction (pMH060)
KPO-4469	GAGATACTGAGCACATGCATATTCCCTATGCTGAGCGC	Plasmid construction (pJR043)
KPO-4470	CCAGCGGATCCGCTAGCATAAATTTGATAAATTGCAGCGG	Plasmid construction (pJR043)
KPO-4471	GAGATACTGAGCACATGCATAGGACTTACACCGTATCAAC	Plasmid construction (pJR044)
KPO-4472	CCAGCGGATCCGCTAGCTCGGTCTGCATTCATAAAATTG	Plasmid construction (pJR044)
KPO-4473	GAGATACTGAGCACATGCATCTGCAAGAGCTGAACCGG	Plasmid construction (pJR045)
KPO-4474	CCAGCGGATCCGCTAGCATCGACCAACGCGACACG	Plasmid construction (pJR045)
KPO-4642	GAGATACTGAGCACATGCATAAAAAACCTACTTATCTACGTC	Plasmid construction (pMH067)
KPO-4643	CCAGCGGATCCGCTAGCGGCAAAGGTGAGCAGAGG	Plasmid construction (pMH067)

KPO-4651	CAGCCTGAATGGCGAATGGCACTGGCAGTTGCACGC	Plasmid construction (pMH066)
KPO-4935	CAGCCTGAATGGCGAATGGCCACTCAGCGCTTTCCGT	Plasmid construction (pMH072)
KPO-4936	CCAGCGGATCCGCTAGCGGTGCTTCCCGCGCTTTG	Plasmid construction (pMH072)
KPO-4937	GAGATACTGAGCACATGCATGTCCGTATAGTGACACAGA	Plasmid construction (pMH073)
KPO-4938	CCAGCGGATCCGCTAGCAATGGTCTGCAAATCTAAATTAAC	Plasmid construction (pMH073)
KPO-5187	CAGCCTGAATGGCGAATGGCGTTGGTTGATTGATAGCCG	Plasmid construction (pKT003)
KPO-5188	CCAGCGGATCCGCTAGCTGCATTGGCGCCAGCCAA	Plasmid construction (pKT003)
KPO-5191	GAGATACTGAGCACATGCATGCATTACCATCAGTAAGCGC	Plasmid construction (pKT001)
KPO-5192	CCAGCGGATCCGCTAGCTTGAATCAGTTGATAAACTTTTTC	Plasmid construction (pKT001)
KPO-5208	CAGCCTGAATGGCGAATGGCCCATCACGGGCATTACC	Plasmid construction (pKT004)
KPO-5209	CCAGCGGATCCGCTAGCATACGCTTTACTGACTTGCTG	Plasmid construction (pKT004)
KPO-5367	CCAGCAGCGGAGCCAGCGGATCCGCTAGC CAATATATTGCGATCTATAC	Plasmid construction (pKT005)
KPO-5369	CCAGCAGCGGAGCCAGCGGATCCGCTAGC GGCACACCCTGACAGCAGAAC	Plasmid construction (pKT008)
KPO-5372	GAGATACTGAGCACATGCATCTGGCTCACCGTTCAGCCGC	Plasmid construction (pSM001)
KPO-5373	CCAGCAGCGGAGCCAGCGGATCCGCTAGC GATCTTGGAGATCAGTCCCG	Plasmid construction (pSM001)
KPO-5371	CCAGCAGCGGAGCCAGC GGATCCGCTAGCAAACGAGTTGTTGGTAACC	Plasmid construction (pKT007)
KPO-5408	GAGATACTGAGCACATGCAT AAGGAAGTAATAAGGTGGAATA	Plasmid construction (pKT005)
KPO-5409	GAGATACTGAGCACATGCAT GTTATTCGCTCCCTTTTTTATG	Plasmid construction (pKT008)
KPO-5410	GAGATACTGAGCACATGCATGCTATCTAGCAGAAGAGGAG	Plasmid construction (pKT007)
KPO-5411	GAGATACTGAGCACATGCATCAAATTCGATAGAACAACCCT	Plasmid construction (pKT006)
KPO-5412	CCAGCGGATCCGCTAGCTGGAATCATGCCCACACGAT	Plasmid construction (pKT006)
KPO-7574	GAGATACTGAGCACATGCATCTTCAGGATTAATGACTGAAG	Plasmid construction (pMH093)
KPO-7575	CCAGCGGATCCGCTAGCTAAAATTTGTAAGACCAGATTTC	Plasmid construction (pMH093)
**Oligonucleotides for sRNA expression plasmids (RIL-seq target validation)**		
KPO-3965	GGCTCGTATAATGTGTGGGTACCCCTGATAATTCGTATC	Plasmid construction (pJR035)
KPO-3966	GCTCAATCAATCACCGGATCCTACACAGGGATTAAATCTC	Plasmid construction (pJR035)
KPO-4062	GGCTCGTATAATGTGTGGATTTTTCTTGGGCTTCCCC	Plasmid construction (pMH057)
KPO-4063	GCTCAATCAATCACCGGATCGGCGGGTTTCTCATTGTG	Plasmid construction (pMH057)

KPO-7115	GCTCAATCAATCACCGGATC GAGCAAGTGTTTAGGAAGAG	Plasmid construction (pMH088, pAL030)
KPO-7117	GCTCAATCAATCACCGGATC GTGAAAATAGCGGGCGATC	Plasmid construction (pMH092, pAL032)
KPO-7119	GCTCAATCAATCACCGGATC GAATGTTGCAGGCTAAATGG	Plasmid construction (pMH090, pAL031)
KPO-7587	TCGGCTCGTATAATGTGTGGGACCCGCAAGGGTCACC	Plasmid construction (pAL030)
KPO-7588	TCGGCTCGTATAATGTGTGGGACCCTTAATTAAGCCGAGG	Plasmid construction (pAL031)
KPO-7603	TCGGCTCGTATAATGTGTGGGACCCTTGTTAAGCCGAGG	Plasmid construction (pAL032)
**Oligonucleotides for construction of other plasmids**		
KPO-1026	P-TGAATAATCAAAGACGAGGCTC	Plasmid construction (pNP012)
KPO-1027	gtttttTCTAGAGAACAGCCAGTTAACTTGAGA	Plasmid construction (pNP012)
KPO-1301	CCAGTTAACTTGAGATGAAAATG GTGCAAGGTTGAATTTTTGTTAGTG	Plasmid construction (pAS005)
KPO-1302	CATTTTCATCTCAAGTTAACTGG	Plasmid construction (pAS005)
KPO-1304	GTTTTTGGTACCGAATGCGTTGTAACTCTATGAAC	Plasmid construction (pAS005)
KPO-1305	GTTTTTCCTAGGTACGGCATAAGTCATGACTCG	Plasmid construction (pAS005)
KPO-1525	GCGGCCCTCTCACTTCC	Plasmid construction (pMH086)
KPO-1529	GGAAGTGAGAGGGCCGCGGCAAAGCCGTTTTTCCATAG	Plasmid construction (pMH086)
KPO-1734	TGAGGATCCGGTGATTGATT	Plasmid construction (pMH085)
KPO-1737	AATCAATCACCGGATCCTCAAAAAAAAAGCACCCCGGTTTG	Plasmid construction (pMH085)
KPO-1872	CATATGCATCCTAGGCCTATTAGCGCTCTGGATGTATTAG	Plasmid construction (pASp017)
KPO-1873	ATAAAGATCATGATATCGACTACAAAGATGACGATAAA TAGTAATTTCTTGGGCAGCACAAAG	Plasmid construction (pASp017)
KPO-1874	GTCGATATCATGATCTTTATAATCACCGTCATGGTCTTTG TAGTCGTTCTTATAGATACACAGCAT	Plasmid construction (pASp017)
KPO-1875	TAGAGGTACCGGTTGTTAACGCAATCCAACCAAAACTAAATC	Plasmid construction (pASp017)
KPO-2558	CGCAACTCTCTACTGTTTCTCCTGAATAATCAAAGACGAGGC	Plasmid construction (pMD099)
KPO-2559	GCTCAATCAATCACCGGATCGAACAGCCAGTTAACTTGAG	Plasmid construction (pMD099)
KPO-3451	TAGAGGTACCGGTTGTTAACGCTTGGCTGCTGCTGGTCT	Plasmid construction (pJR024)
KPO-3452	GAGCGCACCTCAATGATTA	Plasmid construction (pJR024)
KPO-3453	TAATCATTGAGGTGCGCTCTAAATAGGGCATCGGGTGAT	Plasmid construction (pJR024)
KPO-3454	TATGCATCCTAGGCCTATTACCAGAGGTAACAGGTTCTC	Plasmid construction (pJR024)
KPO-3676	GAAGGGTCTTTTTGCATGCCTACTTGTACAGCGGCTTTAT	Plasmid construction (pAF012)

KPO-3677	CAACTAGGCCTGTCGACTATTCAATCATTAGTTTAGGTGCAA	Plasmid construction (pAF012)
KPO-3741	TAGAGGTACCGGTTGTTAACGGTCTCTGCACTCGCCAG	Plasmid construction (pAF013, pAL033)
KPO-3742	ACCGATAAATGTGCCAAGATAAC	Plasmid construction (pAF013, pAL033)
KPO-3743	TCTTGGCACATTTATCGGT GATGAAAATGGGTGAAAAAGGAAAG	Plasmid construction (pAF013)
KPO-3744	CATATGCATCCTAGGCCTATTA GAACATCTGACAAAAATCCTCTC	Plasmid construction (pAF013)
KPO-3749	CAACTCATGCCTAGGGTTATTAACAAAAATCAAAAAC	Plasmid construction (pMD103, pMH092, pMH091)
KPO-3750	TAACCCTAGGCATGAGTTGTCAGTCAATAAACAG	Plasmid construction (pMD103, pMH092, pMH091)
KPO-3779	CGCAACTCTCTACTGTTTCTCCGACCCTTCTAAGCCGAGG	Plasmid construction (pMD176)
KPO-3780	GCTCAATCAATCACCGGATCCCACGAAAGCCAAGATGCTAT	Plasmid construction (pMD176)
KPO-3870	GCTAACAGGAGGAATTAACCATGGATTATATTCACCTCTCC	Plasmid construction (pAL001)
KPO-4660	GTCGACCTCGAGGGGG	Plasmid construction (pMD285)
KPO-4661	ACTAGTTCTAGAGCGGCCG	Plasmid construction (pMD285)
KPO-4662	GGCCGCTCTAGAACTAGT CTACTTGTACAGCGGCTTTATTT	Plasmid construction (pMD285)
KPO-4663	CCCCTCGAGGTCGAC TATTCAATCATTAGTTTAGGTGCAAG	Plasmid construction (pMD285)
KPO-5419	GAAGGGTCTTTTTGCATGCCTACTTGTACAGCGGCTTTATT	Plasmid construction (pLH002)
KPO-5420	TGCTCAATCAATCACCGGATCCTCA AAAAAGGAAAGGGTCAGCAAAC	Plasmid construction (pLH002)
KPO-6321	TCGTTTTATTTGATGCCTCTAGATTA TCAAGTTAACTGGCTGTTCAG	Plasmid construction (pAL001)
KPO-6752	CAATTTACGATTGATATGCAAATCACATTGTTAAC	Plasmid construction (pMH085)
KPO-6753	CGTAAATTGACCTTCTTTTCACTAACAAAAATTC	Plasmid construction (pMH083)
KPO-6754	GTTAGTGAAAAGAAGGTCAATTTACGATTTGGAAA	Plasmid construction (pMH083)
KPO-6884	GATTTGCATATCAATCGTAAATTGACCATTTACTTC	Plasmid construction (pMH085)
KPO-7032	CGAGGGTCACCTAGGCAACTGACGTTGTTAGTGAAC	Plasmid construction (pMH086, pAL035)
KPO-7033	CTAACAACGTCAGTTGCCTAGGTGACCCTCGGCTT	Plasmid construction (pMH086, pAL035)
KPO-7114	CGCAACTCTCTACTGTTTCTCC GACCCGCAAGGGTCACC	Plasmid construction (pMH088)
KPO-7118	CGCAACTCTCTACTGTTTCTCC GACCCTTAATTAAGCCGAGG	Plasmid construction (pMH090)
KPO-7120	TAGAGGTACCGGTTGTTAACG CAGCATTGCCCGAATACAAG	Plasmid construction (pMH091)
KPO-7121	CATATGCATCCTAGGCCTATTA CACCTCTCCCGATTTAGC	Plasmid construction (pMH091)

KPO-7122	TAGAGGTACCGGTTGTTAACG GATGGATTGGGATTCATTGC	Plasmid construction (pAL035)
KPO-7123	CATATGCATCCTAGGCCTATTA CGCATGATGTAACAGACGAAC	Plasmid construction (pAL035)
KPO-7338	CGCAACTCTCTACTGTTTCTCC GACCCTTGTTAAGCCGAGG	Plasmid construction (pMH092)
KPO-7431	AAGGGTCACCTAGgCAACTGACGTTGTTAGTGAAT	Plasmid construction (pAL026, pAL036)
KPO-7432	AACAACGTCAGTTGcCTAGGTGACCCTTGCGG	Plasmid construction (pAL026, pAL036)
KPO-7433	ACGTTGTTAGTGAAtACCATTGTTCACACTTATAG	Plasmid construction (pAL027)
KPO-7434	GTGTGAACAATGGTaTTCACTAACAACGTCAGTTG	Plasmid construction (pAL027)
KPO-7604	TCTTGGCACATTTATCGGTGTGCAAGGTTGAATTTTTGTTAGTG	Plasmid construction (pAL033)
KPO-7605	CATATGCATCCTAGGCCTATTA GAATGCGTTGTAACTCTATGAAC	Plasmid construction (pAL033)
KPO-7668	TAGAGGTACCGGTTGTTAACG GCGTCCTTCTTGCACTTCC	Plasmid construction (pAL036)
KPO-7669	CATATGCATCCTAGGCCTATTA GGTGGATGATCTGCTTTCTG	Plasmid construction (pAL036)
**Oligonucleotides for Northern blot probing**		
KPO-0060	GTCTATTGGCTGTTATTTGTGAAC	Qrr1 oligoprobe
KPO-0061	GGCTTATATATGATGTGAACAATAC	Qrr2 oligoprobe
KPO-0062	GGCTTATATATGATGTGAACAATAC	Qrr3 oligoprobe
KPO-0063	CGTCTATAAGTGTGAACAATGGTG	Qrr4 oligoprobe
KPO-0243	TTCGTTTCACTTCTGAGTTCGG	5S oligoprobe
KPO-3794	GACCCTTTCCTTTGTTGCTC	QrrX oligoprobe
**Oligonucleotides for qPCR**		
KPO-0496	GTTTGTACTTTACCGAACGC	P_{vqmR} qPCR
KPO-1539	CGACACTGTTTCATTCATTGG	P_{qrrX} qPCR
KPO-2378	GGTAACCCAGAAACTACCACTG	*recA* qPCR
KPO-2379	CACCACTTCTTCGCCTTCTT	*recA* qPCR
KPO-7711	AGTTTAGGTGCAAGGTTGA	P_{qrrX} qPCR
KPO-7712	CGCCAATTATGTCGGTTTC	P_{vqmR} qPCR

Supplemental References

1. M. Rehmsmeier, P. Steffen, M. Hochsmann, R. Giegerich, Fast and effective prediction of microRNA/target duplexes. *RNA* **10**, 1507-1517 (2004).
2. F. Corpet, Multiple sequence alignment with hierarchical clustering. *Nucleic acids research* **16**, 10881-10890 (1988).
3. C. P. Corcoran *et al.*, Superfolder GFP reporters validate diverse new mRNA targets of the classic porin regulator, MicF RNA. *Molecular microbiology* **84**, 428-445 (2012).
4. K. Papenfort, K. U. Forstner, J. P. Cong, C. M. Sharma, B. L. Bassler, Differential RNA-seq of Vibrio cholerae identifies the VqmR small RNA as a regulator of biofilm formation. *Proc Natl Acad Sci U S A* **112**, E766-775 (2015).
5. D. G. Gibson, Synthesis of DNA fragments in yeast by one-step assembly of overlapping oligonucleotides. *Nucleic Acids Res* **37**, 6984-6990 (2009).
6. N. Peschek, M. Hoyos, R. Herzog, K. U. Forstner, K. Papenfort, A conserved RNA seed-pairing domain directs small RNA-mediated stress resistance in enterobacteria. *EMBO J* **38**, e101650 (2019).
7. A. K. Dunn, D. S. Millikan, D. M. Adin, J. L. Bose, E. V. Stabb, New rfp- and pES213-derived tools for analyzing symbiotic Vibrio fischeri reveal patterns of infection and lux expression in situ. *Appl Environ Microbiol* **72**, 802-810 (2006).
8. R. Herzog, N. Peschek, K. S. Frohlich, K. Schumacher, K. Papenfort, Three autoinducer molecules act in concert to control virulence gene expression in Vibrio cholerae. *Nucleic Acids Res* **47**, 3171-3183 (2019).
9. K. Skorupski, R. K. Taylor, Positive selection vectors for allelic exchange. *Gene* **169**, 47-52 (1996).
10. C. M. Waters, B. L. Bassler, The Vibrio harveyi quorum-sensing system uses shared regulatory components to discriminate between multiple autoinducers. *Genes Dev* **20**, 2754-2767 (2006).
11. L. Fried, J. Lassak, K. Jung, A comprehensive toolbox for the rapid construction of lacZ fusion reporters. *J Microbiol Methods* **91**, 537-543 (2012).
12. Z. Donnell (2015) Regulation of cqsA and cqsS in Vibrio cholerae. (Princeton University, Princeton).
13. M. Hoyos, M. Huber, K. U. Forstner, K. Papenfort, Gene autoregulation by 3' UTR-derived bacterial small RNAs. *Elife* **9** (2020).
14. M. Huber, K. S. Frohlich, J. Radmer, K. Papenfort, Switching fatty acid metabolism by an RNA-controlled feed forward loop. *Proc Natl Acad Sci U S A* **117**, 8044-8054 (2020).
15. B. M. Davis, M. K. Waldor, RNase E-dependent processing stabilizes MicX, a Vibrio cholerae sRNA. *Mol Microbiol* **65**, 373-385 (2007).
16. J. P. Bardill, X. Zhao, B. K. Hammer, The Vibrio cholerae quorum sensing response is mediated by Hfq-dependent sRNA/mRNA base pairing interactions. *Mol Microbiol* **80**, 1381-1394 (2011).
17. B. K. Hammer, B. L. Bassler, Regulatory small RNAs circumvent the conventional quorum sensing pathway in pandemic Vibrio cholerae. *Proc Natl Acad Sci U S A* **104**, 11145-11149 (2007).
18. Y. Shao, B. L. Bassler, Quorum-sensing non-coding small RNAs use unique pairing regions to differentially control mRNA targets. *Mol Microbiol* **83**, 599-611 (2012).
19. S. T. Rutherford, J. C. van Kessel, Y. Shao, B. L. Bassler, AphA and LuxR/HapR reciprocally control quorum sensing in vibrios. *Genes Dev* **25**, 397-408 (2011).
20. S. Yamamoto *et al.*, Identification of a chitin-induced small RNA that regulates translation of the tfoX gene, encoding a positive regulator of natural competence in Vibrio cholerae. *J Bacteriol* **193**, 1953-1965 (2011).
21. K. Han, S. Lory, Toward a Comprehensive Analysis of Posttranscriptional Regulatory Networks: a New Tool for the Identification of Small RNA Regulators of Specific mRNAs. *mBio* **12** (2021).
22. N. Peschek *et al.*, RNA-mediated control of cell shape modulates antibiotic resistance in Vibrio cholerae. *Nat Commun* **11**, 6067 (2020).
23. K. Venkat *et al.*, A dual-function RNA balances carbon uptake and central metabolism in Vibrio cholerae. *EMBO J* 10.15252/embj.2021108542, e108542 (2021).
24. T. Song *et al.*, A new Vibrio cholerae sRNA modulates colonization and affects release of outer membrane vesicles. *Mol Microbiol* **70**, 100-111 (2008).

25. T. Song, D. Sabharwal, S. N. Wai, VrrA mediates Hfq-dependent regulation of OmpT synthesis in Vibrio cholerae. *J Mol Biol* **400**, 682-688 (2010).
26. M. B. Miller, K. Skorupski, D. H. Lenz, R. K. Taylor, B. L. Bassler, Parallel quorum sensing systems converge to regulate virulence in Vibrio cholerae. *Cell* **110**, 303-314 (2002).
27. K. H. Thelin, R. K. Taylor, Toxin-coregulated pilus, but not mannose-sensitive hemagglutinin, is required for colonization by Vibrio cholerae O1 El Tor biotype and O139 strains. *Infect Immun* **64**, 2853-2856 (1996).
28. S. L. Svenningsen, K. C. Tu, B. L. Bassler, Gene dosage compensation calibrates four regulatory RNAs to control Vibrio cholerae quorum sensing. *EMBO J* **28**, 429-439 (2009).
