# Supplementary material for: An RNA sponge controls quorum sensing dynamics and biofilm formation in Vibrio cholerae
Source: Nat Commun. 2022 Dec 8;13:7585. doi: 10.1038/s41467-022-35261-x (PMC9732341; doi:10.1038/s41467-022-35261-x)
Supplement: Supplementary file 3 — Description of Additional Supplementary Files [file 41467_2022_35261_MOESM3_ESM.pdf]

Description of Additional Supplementary Files:

Supplementary Data 1: List of candidate sRNA-target interactions in V. cholerae identified by RILSeq
