# Supplementary material for: An RNA sponge controls quorum sensing dynamics and biofilm formation in Vibrio cholerae
Source: Nat Commun. 2022 Dec 8;13:7585. doi: 10.1038/s41467-022-35261-x (PMC9732341; doi:10.1038/s41467-022-35261-x)
Supplement: Supplementary file 5 — Reporting Summary [file 41467_2022_35261_MOESM5_ESM.pdf]

nature portfolio

Corresponding author(s): Kai Papenfort

Last updated by author(s): 16/11/22

Reporting Summary

Nature Portfolio wishes to improve the reproducibility of the work that we publish. This form provides structure for consistency and transparency in reporting. For further information on Nature Portfolio policies, see our Editorial Policies and the Editorial Policy Checklist.

Statistics

For all statistical analyses, confirm that the following items are present in the figure legend, table legend, main text, or Methods section.

n/a	Confirmed	
☐	☒	The exact sample size (*n*) for each experimental group/condition, given as a discrete number and unit of measurement
☐	☒	A statement on whether measurements were taken from distinct samples or whether the same sample was measured repeatedly
☐	☒	The statistical test(s) used AND whether they are one- or two-sided<br>*Only common tests should be described solely by name; describe more complex techniques in the Methods section.*
☒	☐	A description of all covariates tested
☐	☒	A description of any assumptions or corrections, such as tests of normality and adjustment for multiple comparisons
☐	☒	A full description of the statistical parameters including central tendency (e.g. means) or other basic estimates (e.g. regression coefficient) AND variation (e.g. standard deviation) or associated estimates of uncertainty (e.g. confidence intervals)
☐	☒	For null hypothesis testing, the test statistic (e.g. *F*, *t*, *r*) with confidence intervals, effect sizes, degrees of freedom and *P* value noted<br>*Give P values as exact values whenever suitable.*
☒	☐	For Bayesian analysis, information on the choice of priors and Markov chain Monte Carlo settings
☒	☐	For hierarchical and complex designs, identification of the appropriate level for tests and full reporting of outcomes
☒	☐	Estimates of effect sizes (e.g. Cohen's *d*, Pearson's *r*), indicating how they were calculated

Our web collection on statistics for biologists contains articles on many of the points above.

Software and code

Policy information about availability of computer code

Data collection	Next-generation sequencing data was collected by Illumina HiSeq 2500 (RNA-seq: paired-read mode). Phase contrast imaging was performed on a Zeiss Axio Imager M1 microscope equipped with EC Plan Neofluar 100x/ 1.3 Oil Ph3 objective (Zeiss). Confocal Microscopy: Images were acquired using a Zeiss LSM 880 laser scanning confocal microscope fitted with a 40x/1.2 N.A. water immersion objective.
Data analysis	RIL-seq computational analysis<br>De-multiplexed raw sequencing reads were checked for quality using fastP (v0.23.2) and polyX tails, regions of low complexity, as well as low quality tails were removed. The remaining reads were mapped to the V. cholerae reference genome (NCBI accession numbers NC_002505.1 and NC_002506.1), using bwa-mem2 (v2.2.1) with default values for the affine gap model and samtools (v1.16.1). A minimum score of 20 was set to allow for ~4 errors in reads with lengths 36 (read1) and 45 (read2) and the reads were mapped in paired-end mode. Bwa-mem2 handles chimeric reads and produces one alignment per fragment in each read. Alignments, which were assigned to multiple positions were discarded. The alignments resulting from the paired-end mapping were assigned to the annotations of the V. cholerae reference genome, including annotations for Vcr001-Vcr10714 and Vcr200-Vcr23013 and the predicted 5'UTRs and 3'UTRs, and then grouped and sorted according to the position of the read they originated from. In every such set of collected alignments, each pair of alignments was then classified to be chimeric or not by checking for both parts to be at least 1,000 nt apart from each other on the V. cholerae genome, while not sharing the same annotation. Every pair of chimeric alignments was then counted as an interaction between the annotations they belong to. Depending on the number of alignments found in a pair of reads, this procedure can result in a 'single' transcript, or one or more interactions per read pair. Replicates were pooled together, adding each replicates interactions to the pooled sum. After processing all alignments, the resulting interactions were assigned a p-value by testing for the significance of the interaction between two annotations against the background of all other interactions using the right tailed Fisher's exact test. The p-values were corrected using the method of Benjamini-Hochberg. The interactions were then filtered by their number and their statistical significance using a cut-off of 20 reads per interaction and a false discovery rate of 0.05. This strategy was adapted from previous RIL-seq studies and allowed us to recover a high number of published sRNA-

target mRNA interaction from V. cholerae (Table S1).
Microscopy and image analysis
Biofilm imaging was done using a Zeiss LSM 880 laser scanning confocal microscope fitted with a 40x/1.2 N.A. water immersion objective. A 488nm laser line was used to excite sfGFP producing strains and a 594nm laser was used for the mRuby3 producing strains. Replicate images of each biofilm were taken from independent locations from microfluidic devices inoculated with separate identical cultures. Microscope hardware was run via Zeiss Zen Black software. The 3D confocal image data collected from these replicates was then analyzed using the image analysis framework BiofilmQ. Briefly, confocal image data were processed for segmentation and partitioned into a cubic grid with each cube side approximately 1 cell in length (2µm). Frequency diagrams were generated by using the local thickness parameter. 3D renderings of the biofilms were created using Paraview software utilizing OptiX pathtracer raycasting.
Band intensities of Western Blots were quantified using the Bio-1D software (version 15.08, Vilber).
Band intensities of Northern Blots were quantified using the GelQuant software (version 1.8.2, BiochemLabsolutions.com).
The MultAlin algorithm (version 5.4.1; http://multalin.toulouse.inra.fr/multalin/) was used to align sequences.
The RNAhybrid algorithm (version 2.1.2; http://bibserv.cebitec.uni-bielefeld.de/rnahybrid) was used to predict RNA duplex formation.
Statistics were calculated using Graphpad Prism (version 8.4.3; Graphpad software).

For manuscripts utilizing custom algorithms or software that are central to the research but not yet described in published literature, software must be made available to editors and reviewers. We strongly encourage code deposition in a community repository (e.g. GitHub). See the Nature Portfolio guidelines for submitting code & software for further information.

Data

Policy information about availability of data

All manuscripts must include a data availability statement. This statement should provide the following information, where applicable:
- Accession codes, unique identifiers, or web links for publicly available datasets
- A description of any restrictions on data availability
- For clinical datasets or third party data, please ensure that the statement adheres to our policy

The demultiplexed sequencing data of the RIL-seq experiments are available at the National Center for Biotechnology Information Gene Expression Omnibus (GEO) under the accession code GSE198671. Previously published and reanalyzed Term-seq and dRNA-seq sequencing data can be found under the GEO accession codes GSE144478 and GSE62084, respectively. Additional raw and analyzed data that support the findings of this study are available from the corresponding author upon request. Source data are provided with this paper.

Human research participants

Policy information about studies involving human research participants and Sex and Gender in Research.

Reporting on sex and gender	n/a
Population characteristics	n/a
Recruitment	n/a
Ethics oversight	n/a

Note that full information on the approval of the study protocol must also be provided in the manuscript.

Field-specific reporting

Please select the one below that is the best fit for your research. If you are not sure, read the appropriate sections before making your selection.

☒ Life sciences ☐ Behavioural & social sciences ☐ Ecological, evolutionary & environmental sciences

For a reference copy of the document with all sections, see nature.com/documents/nr-reporting-summary-flat.pdf

Life sciences study design

All studies must disclose on these points even when the disclosure is negative.

Sample size	All relevant information is provided in the mansucript and supplemental files
Data exclusions	No data were excluded.
Replication	The experiments were conducted in several biological replicates, specified in each case. Western and Northern blots were performed in at least 3 replicates (unless stated otherwise) and representative blots are shown.
Randomization	Not applicable. Randomization is not standard for the experiments performed.
Blinding	Not applicable. Blinding is not standard for the experiments performed.

Reporting for specific materials, systems and methods

We require information from authors about some types of materials, experimental systems and methods used in many studies. Here, indicate whether each material, system or method listed is relevant to your study. If you are not sure if a list item applies to your research, read the appropriate section before selecting a response.

Materials & experimental systems

n/a	Involved in the study
☐	☒ Antibodies
☒	☐ Eukaryotic cell lines
☒	☐ Palaeontology and archaeology
☒	☐ Animals and other organisms
☒	☐ Clinical data
☒	☐ Dual use research of concern

Methods

n/a	Involved in the study
☒	☐ ChIP-seq
☒	☐ Flow cytometry
☒	☐ MRI-based neuroimaging

Antibodies

Antibodies used	- anti-FLAG antibody (Sigma, #F1804), 1:1,000 dilution in TBS-T + 3 % BSA<br>- anti-RnaPα antibody (BioLegend, #WP003), 1:5,000 dilution in TBS-T + 3 % BSA
Validation	Information about the anti-FLAGantibody can be found on the manufactor's webpage: https://www.sigmaaldrich.com/catalog/product/sigma/f1804?lang=de&region=DE&gclid=CjwKCAiAv4n9BRA9EiwA30WND9c9YIGDUY2-0pos_HK_M1sLfjEkixsUB1Z40n2xTwq2CRH4luosKxoCzGYQAvD_BwE<br>anti-RnaPα antibody: https://www.biolegend.com/en-us/products/purified-anti-e-coli-rna-polymerase-alpha-antibody-14680
