# Supplementary material for: An RNA sponge controls quorum sensing dynamics and biofilm formation in Vibrio cholerae
Source: Nat Commun. 2022 Dec 8;13:7585. doi: 10.1038/s41467-022-35261-x (PMC9732341; doi:10.1038/s41467-022-35261-x)

Source data for Fig. 2C

Replicate I

QrrX

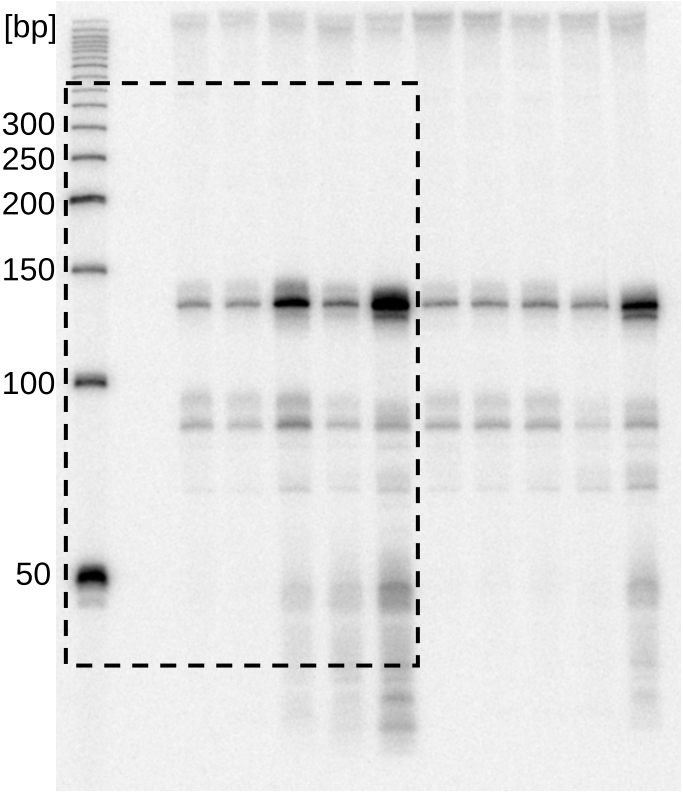

Qrr1

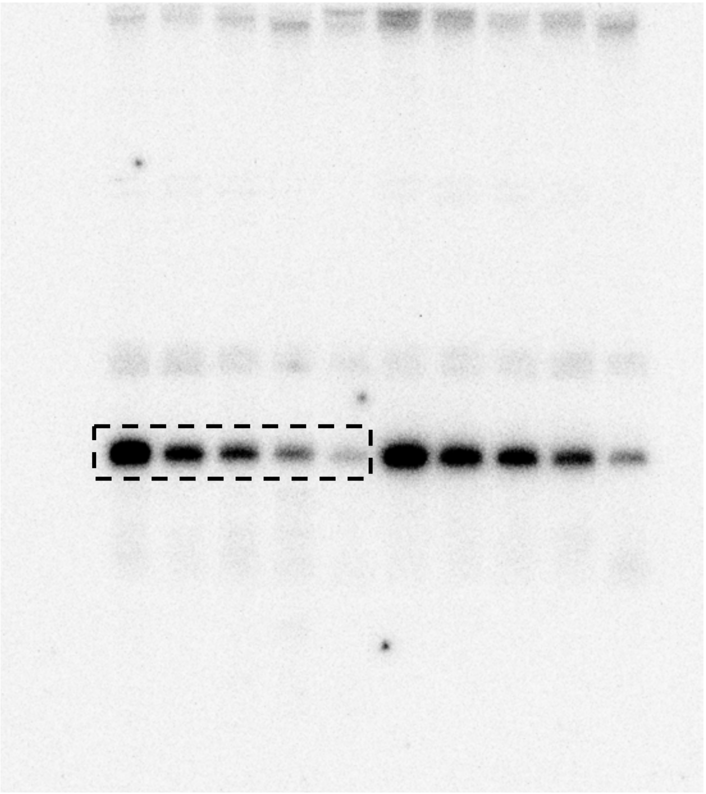

Qrr2

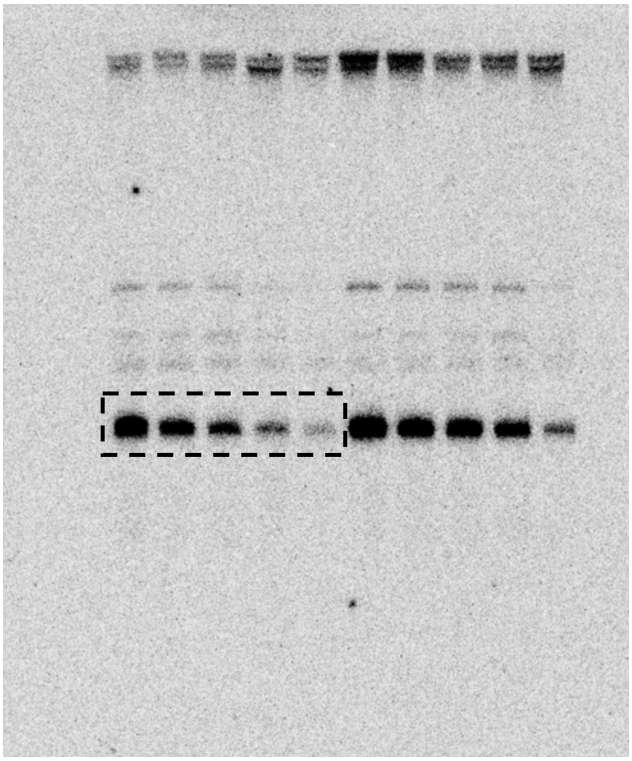

Qrr3

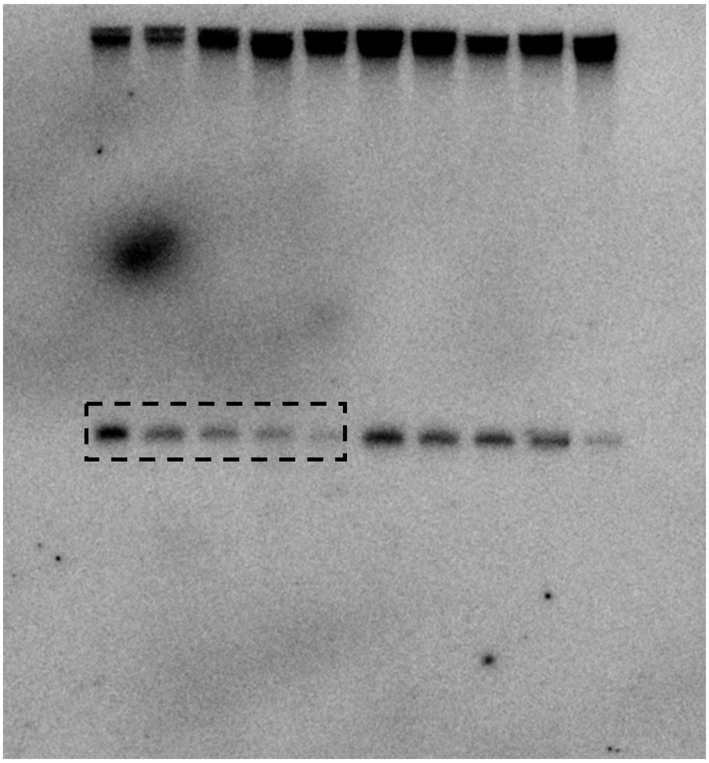

Source data for Fig. 2C

Replicate I

Qrr4

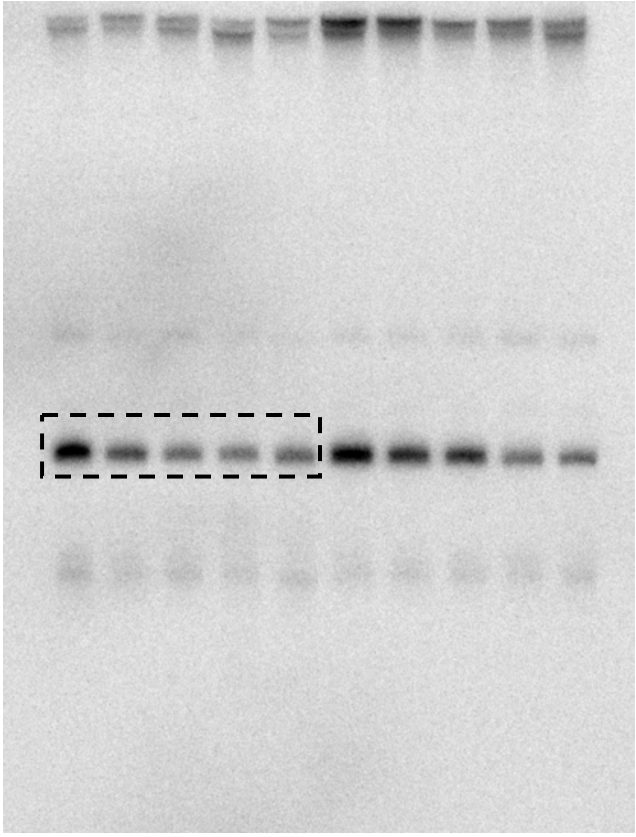

5S rRNA

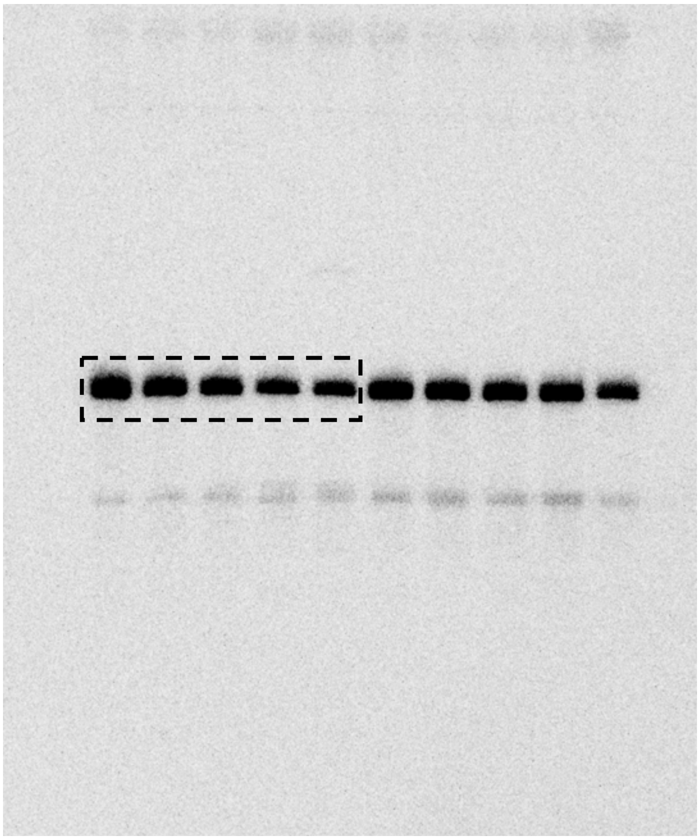

Source data for Fig. 2C

Replicate II

QrrX

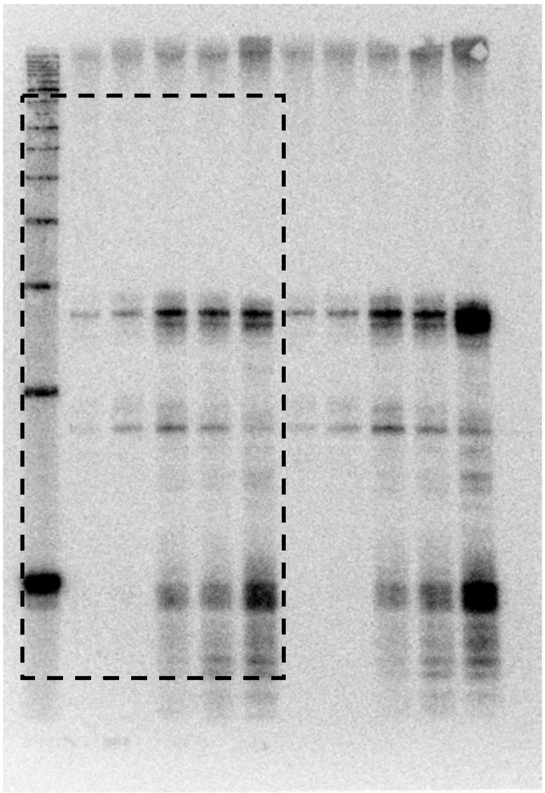

Qrr1

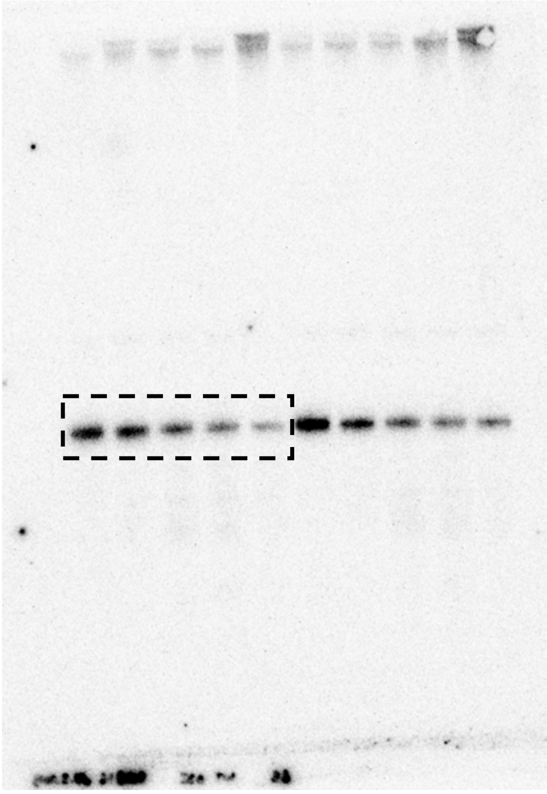

Qrr2

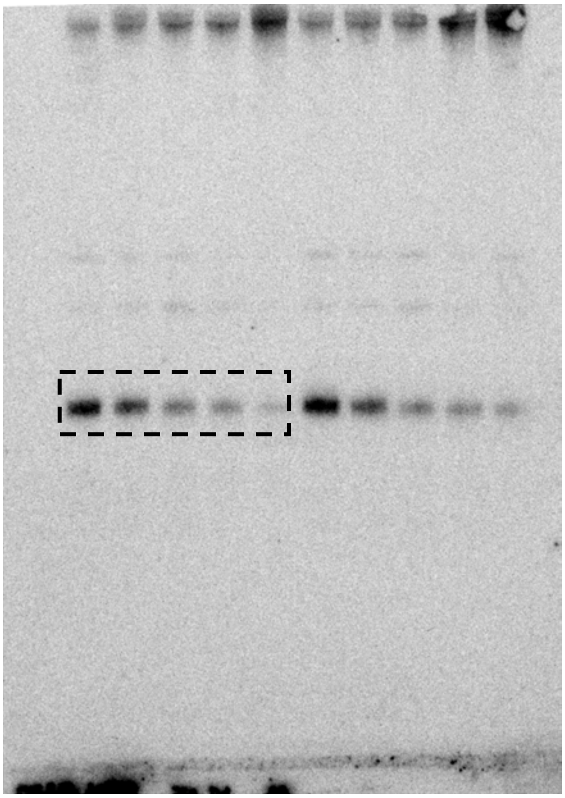

Qrr3

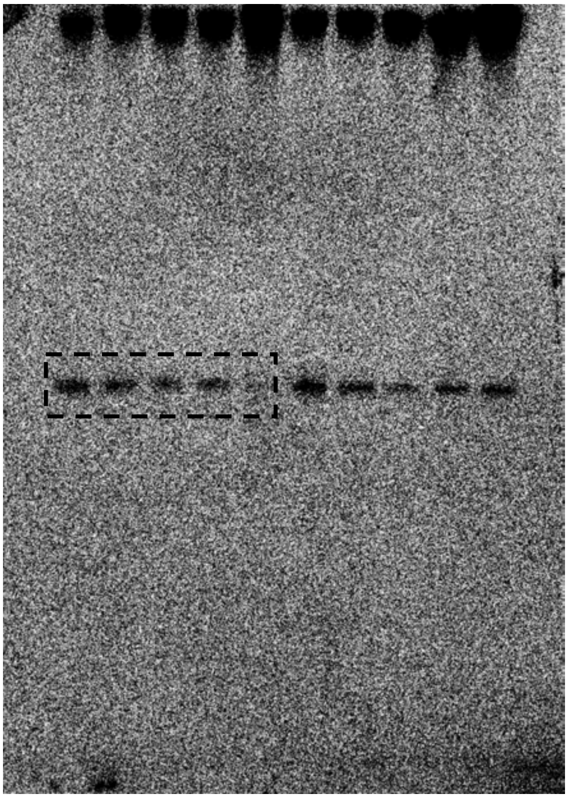

Source data for Fig. 2C

Replicate II

Qrr4

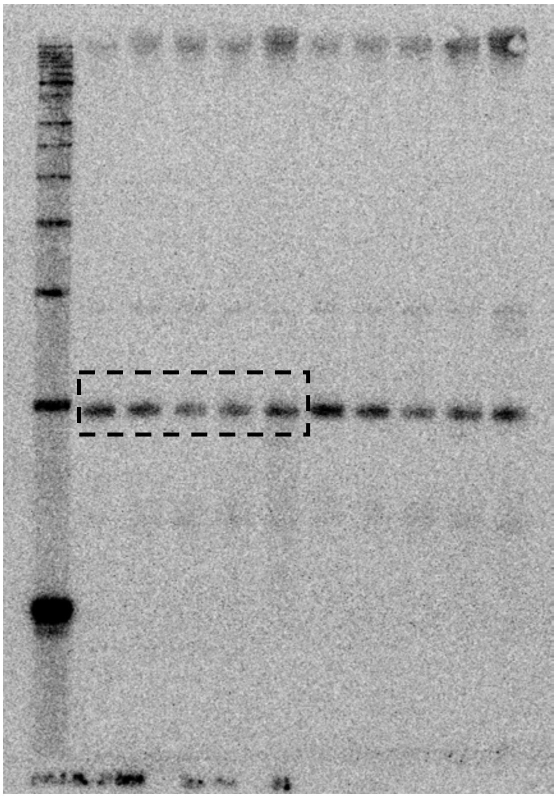

5S rRNA

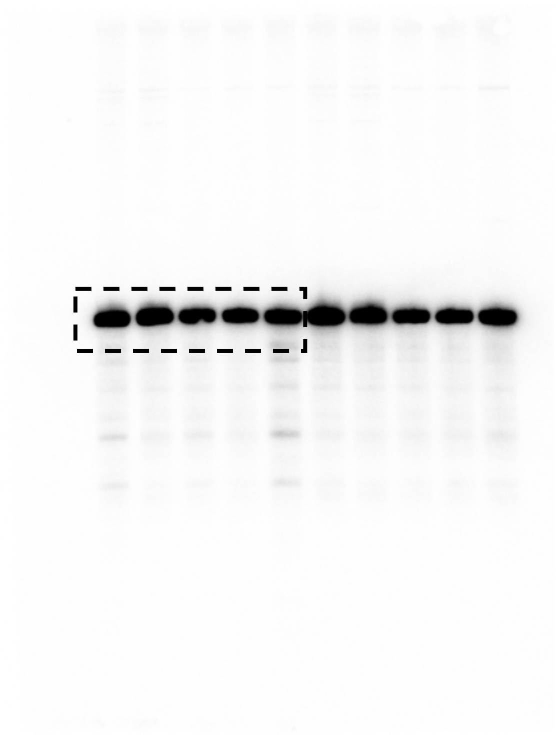

Source data for Fig. 2C

Replicate III

QrrX

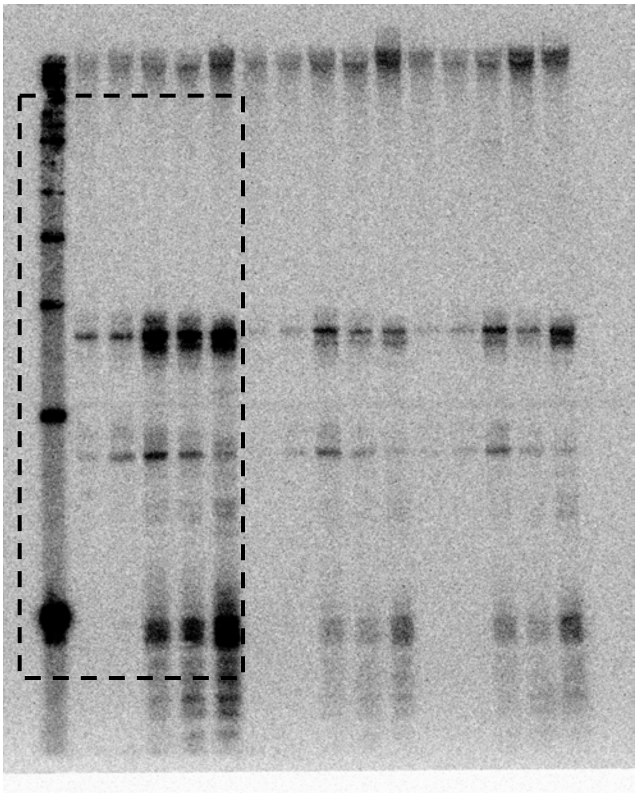

Qrr1

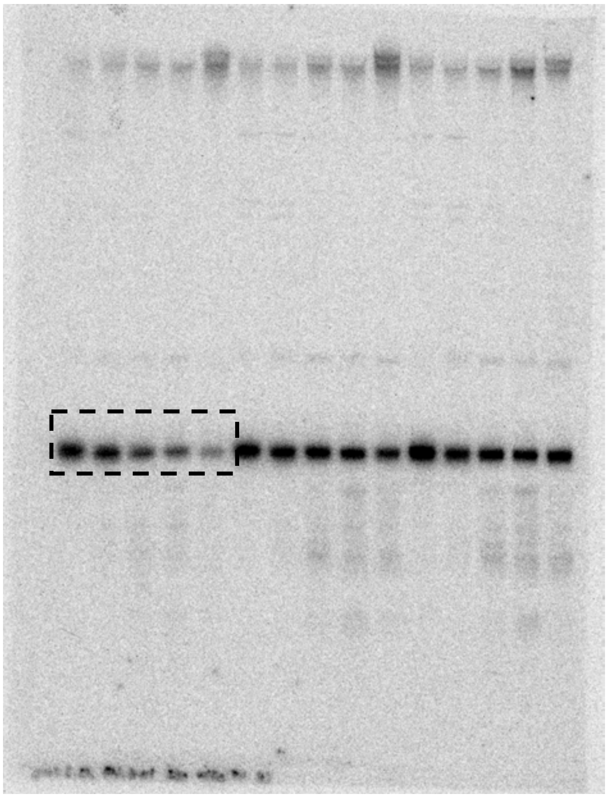

Qrr2

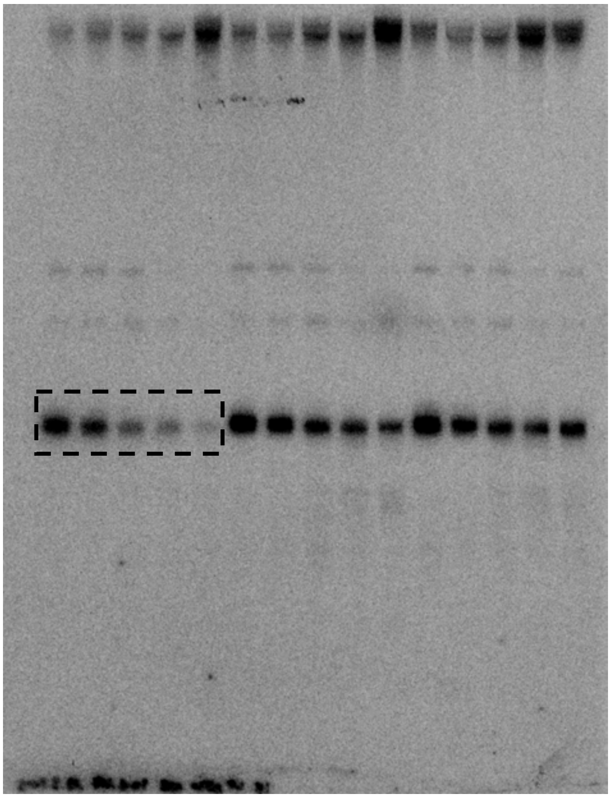

Qrr3

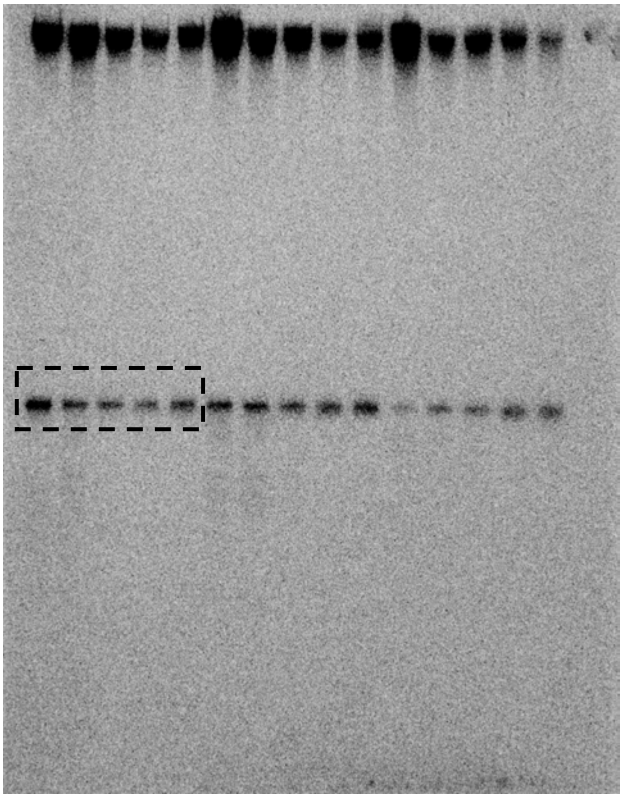

Source data for Fig. 2C

Replicate III

Qrr4

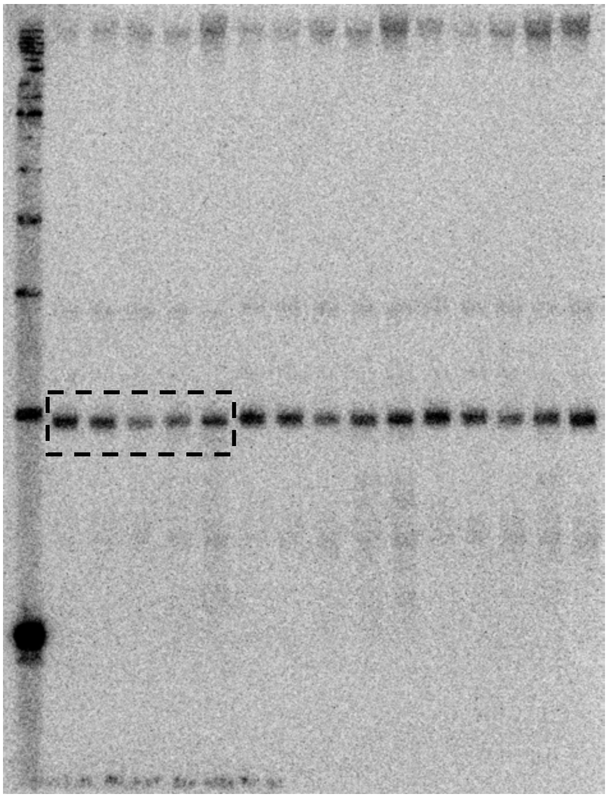

5S rRNA

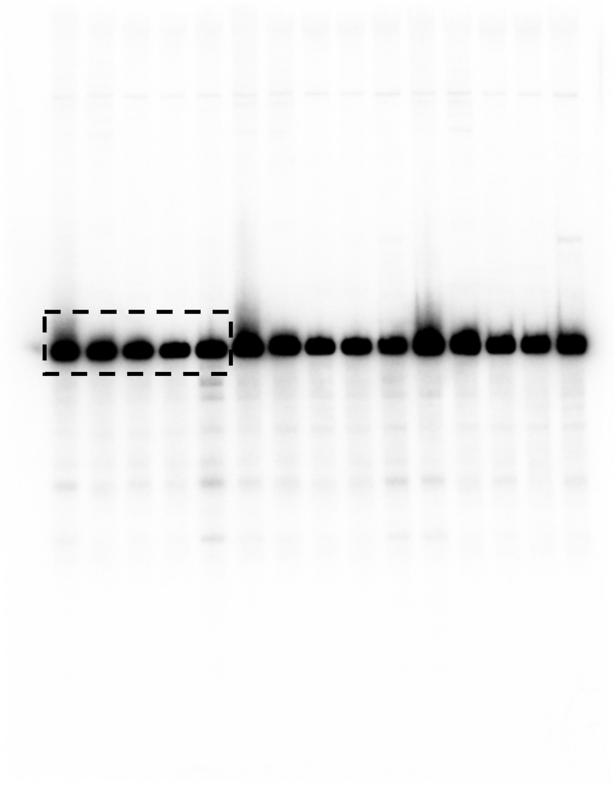

Source data for Fig. 3A

Replicate I

QrrX

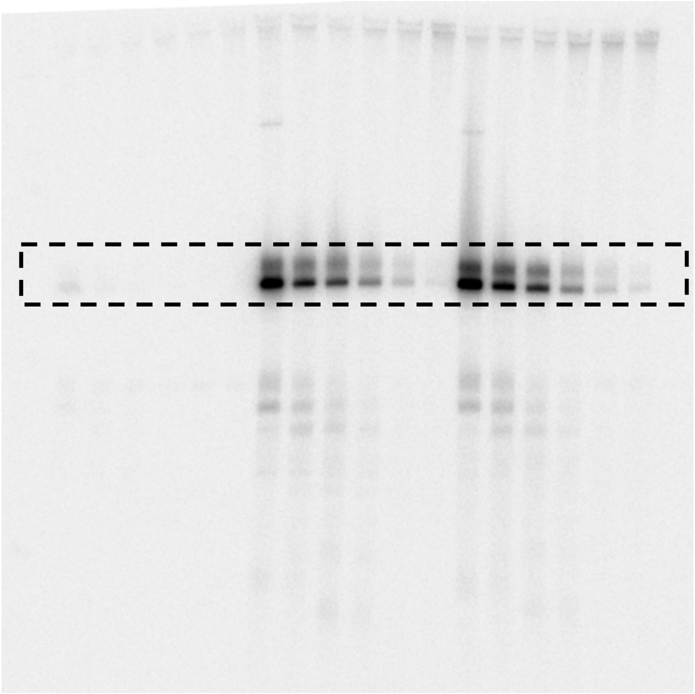

Qrr1

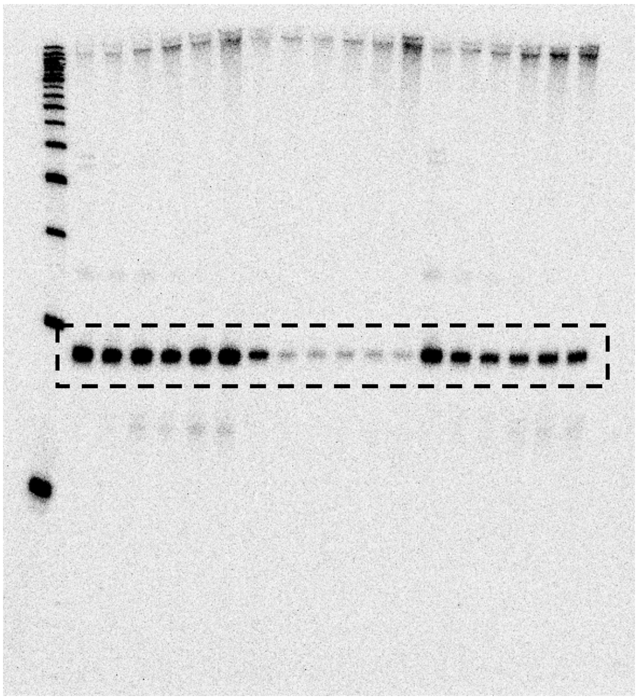

Qrr2

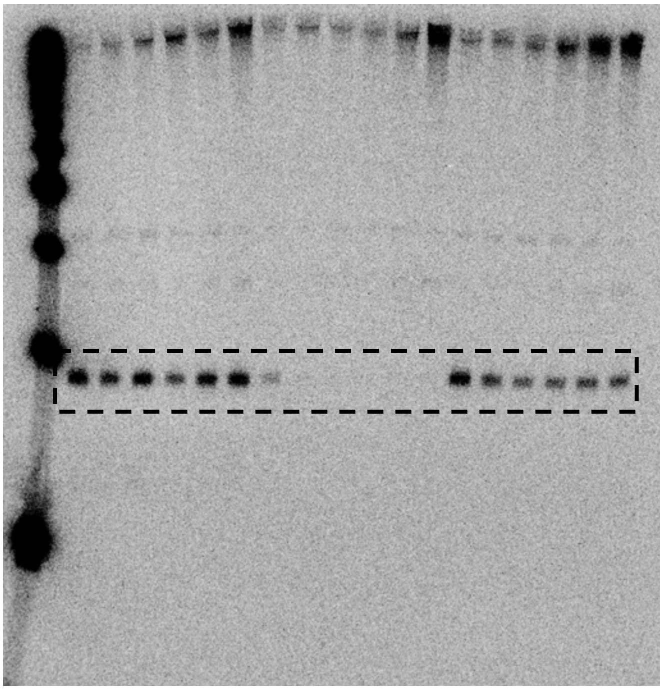

Qrr3

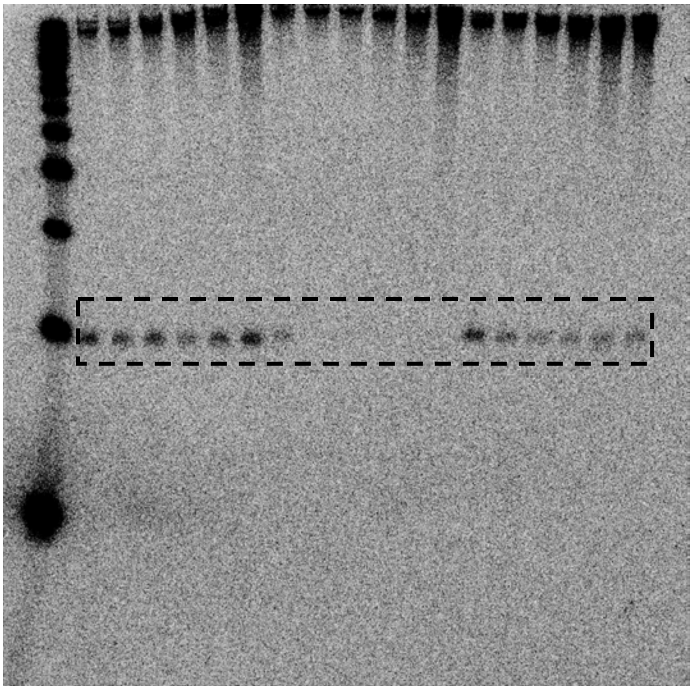

Source data for Fig. 3A

Replicate I

Qrr4

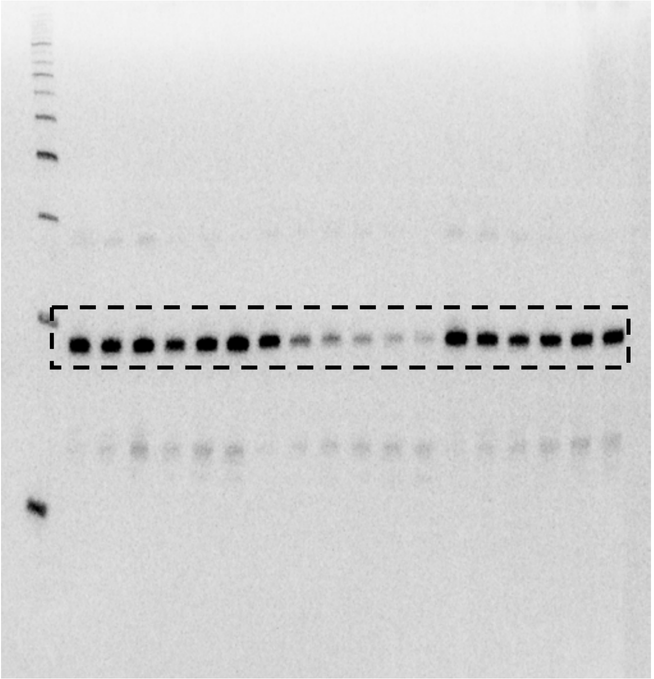

5S rRNA

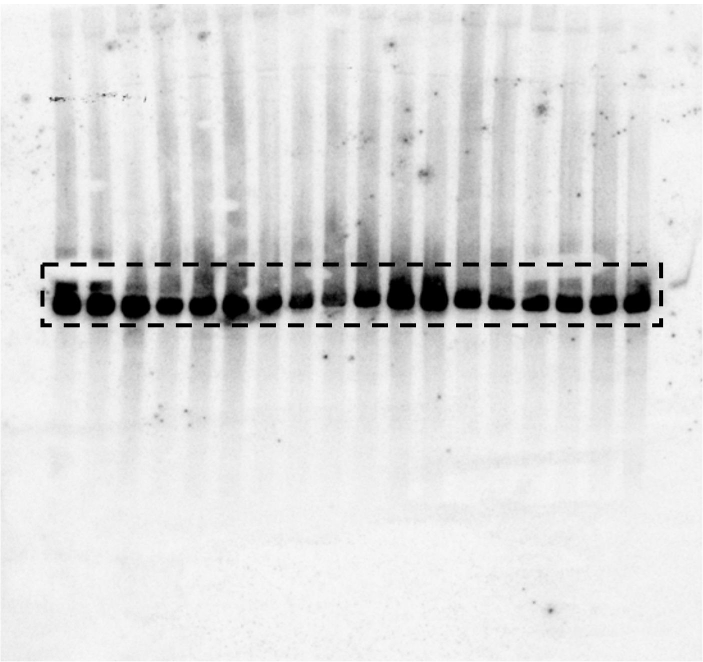

Source data for Fig. 3A

Replicate II

QrrX

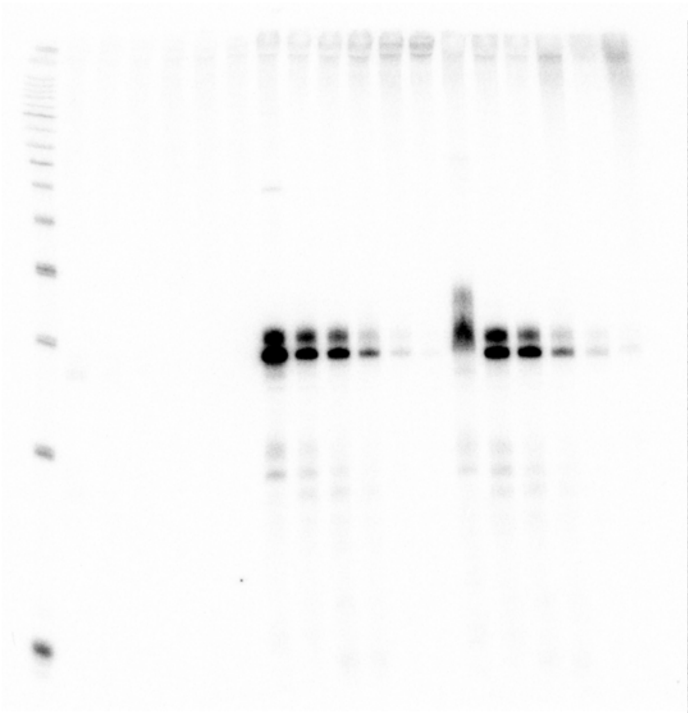

Qrr1

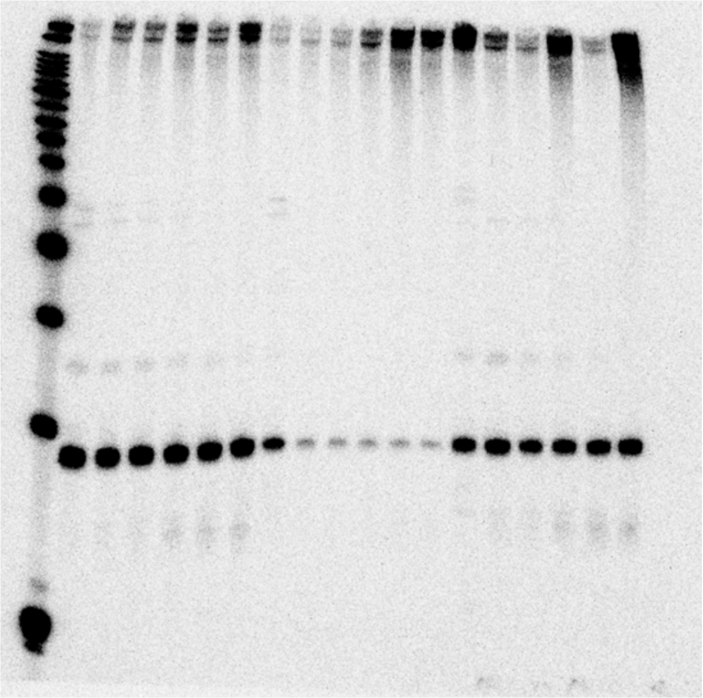

Qrr2

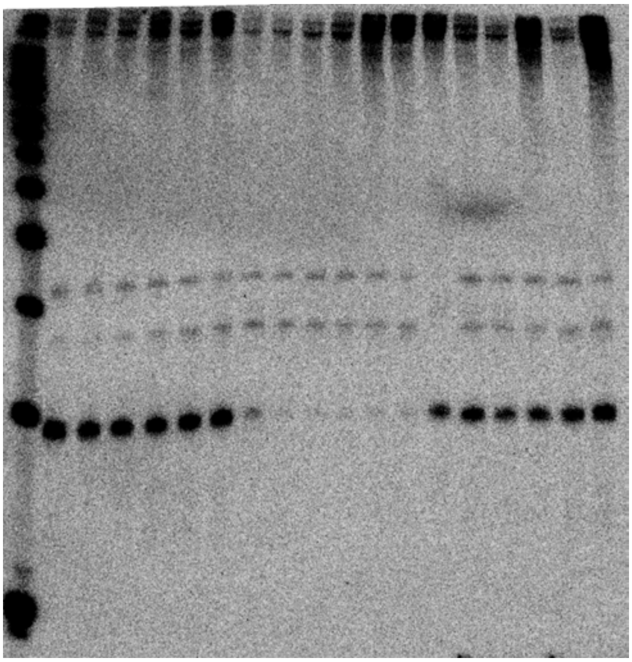

Qrr3

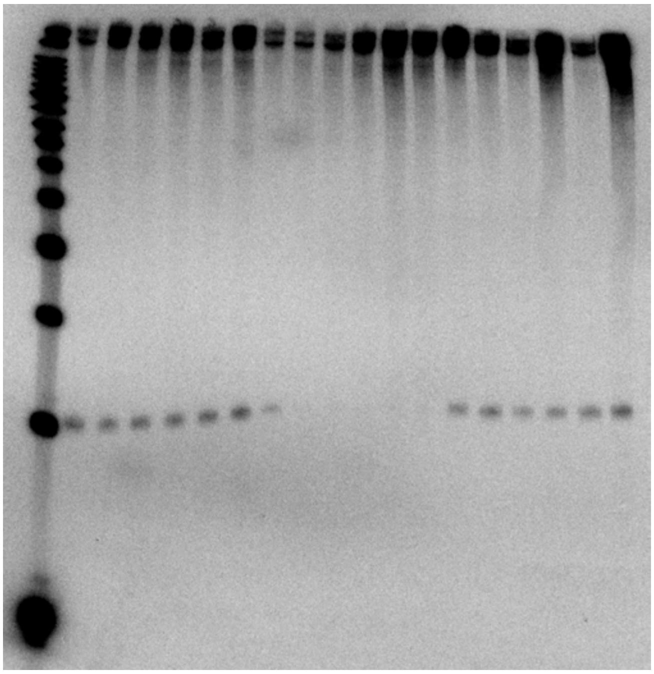

Source data for Fig. 3A

Replicate II

Qrr4

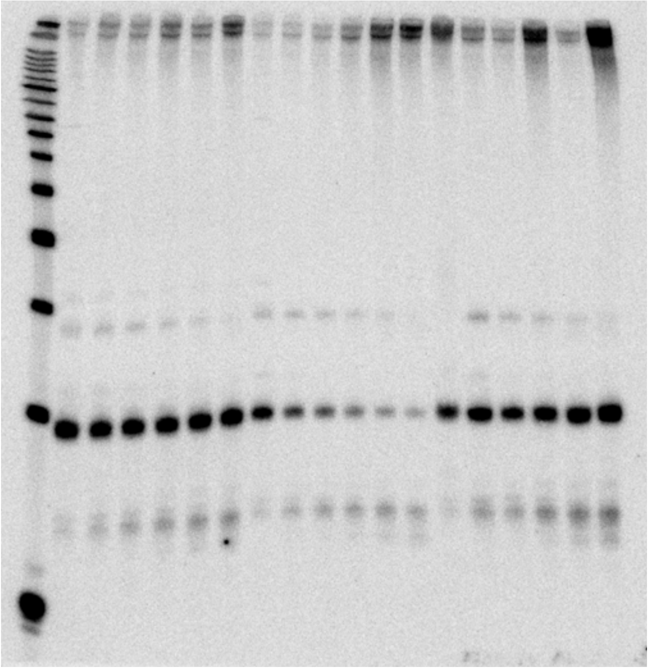

5S rRNA

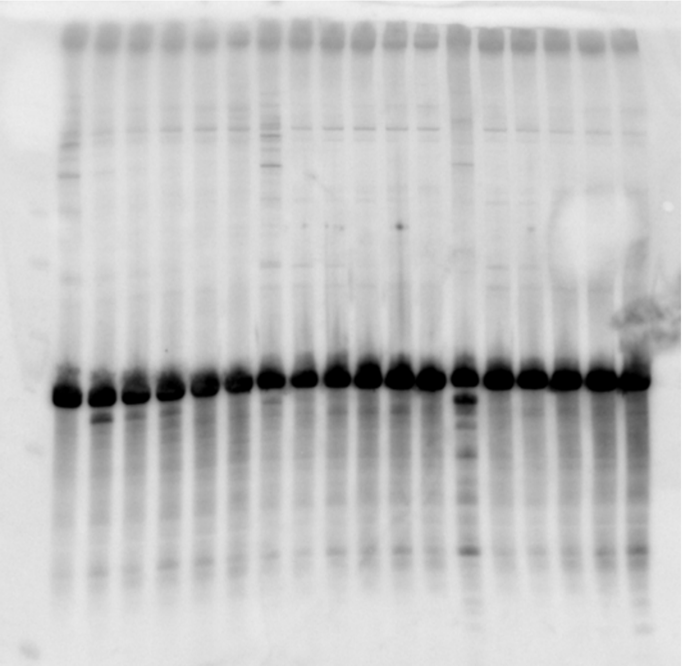

Source data for Fig. 3A

Replicate III

QrrX

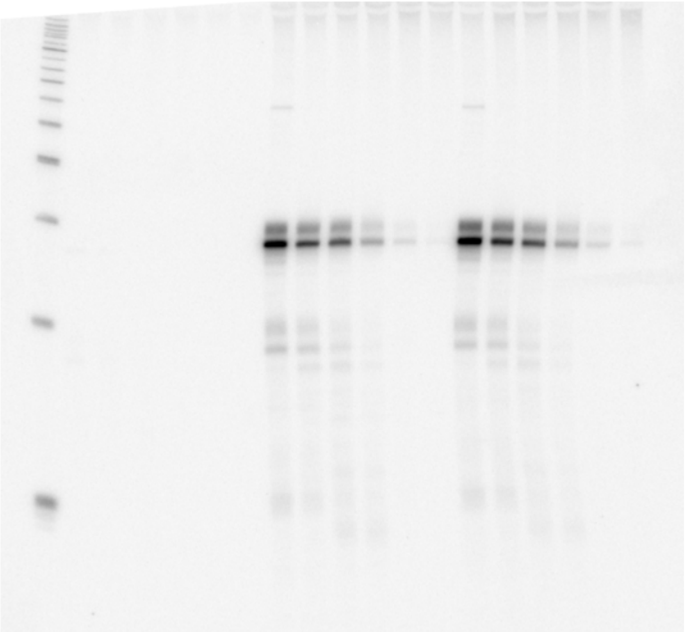

Qrr1

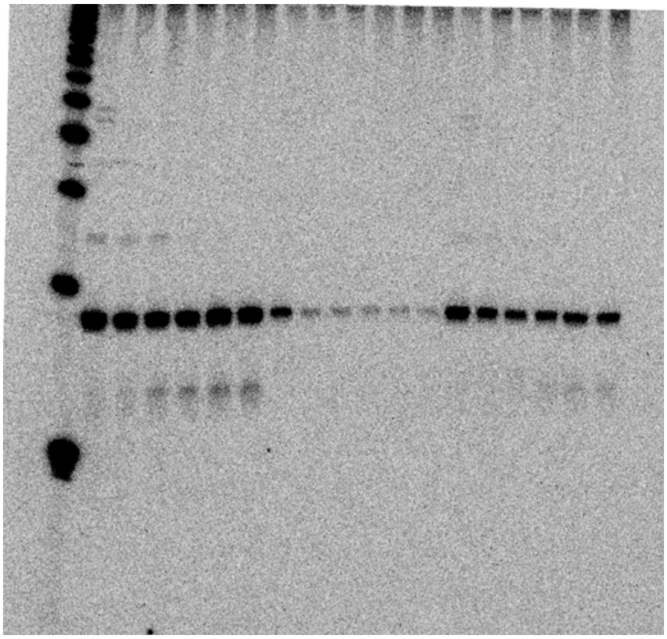

Qrr2

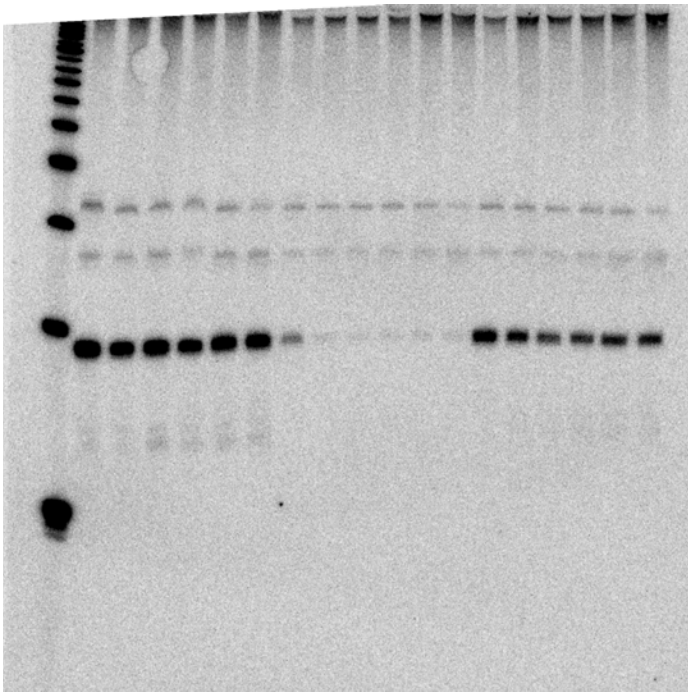

Qrr3

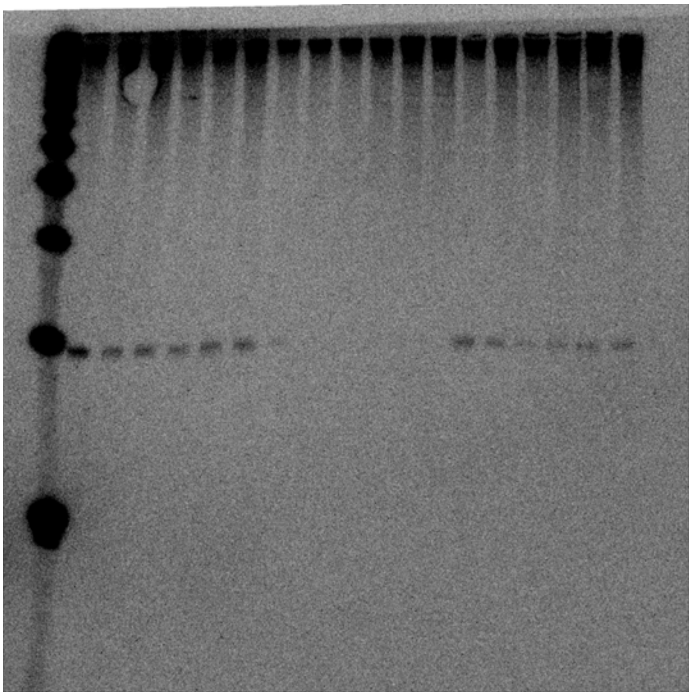

Source data for Fig. 3A

Replicate III

Qrr4

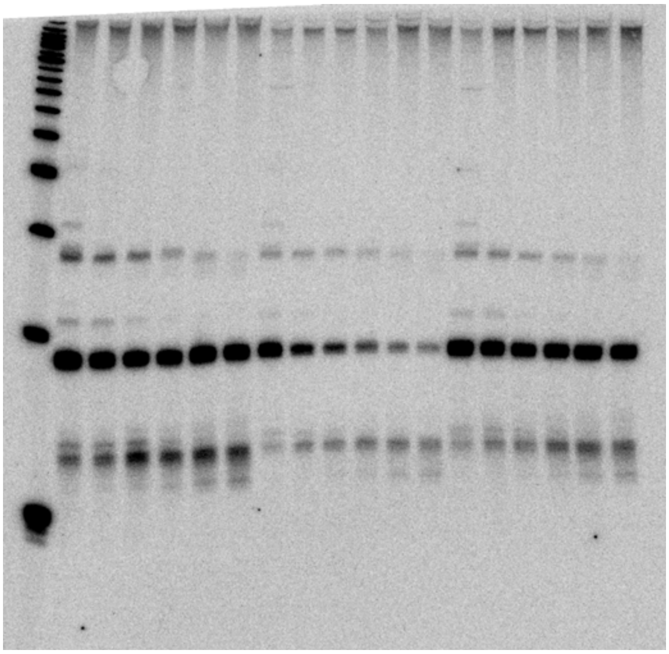

5S rRNA

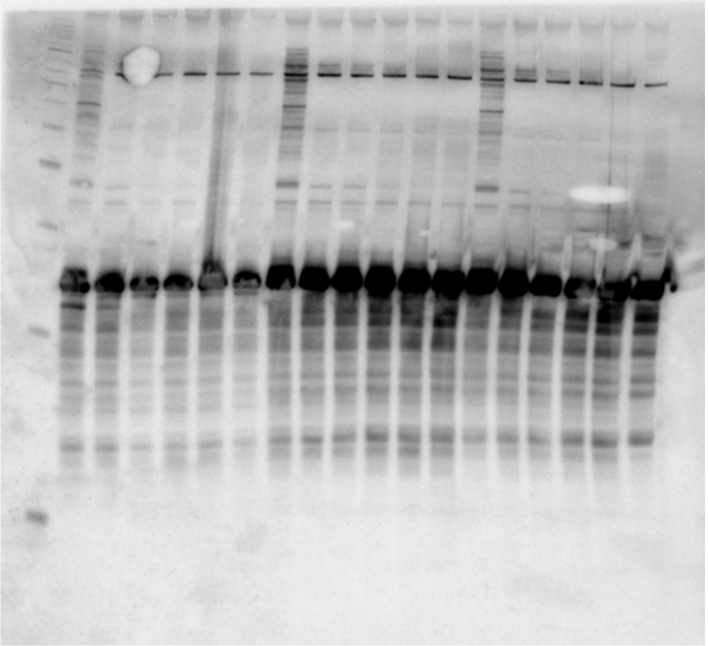

Source data for Fig. 3C

Replicate I

QrrX

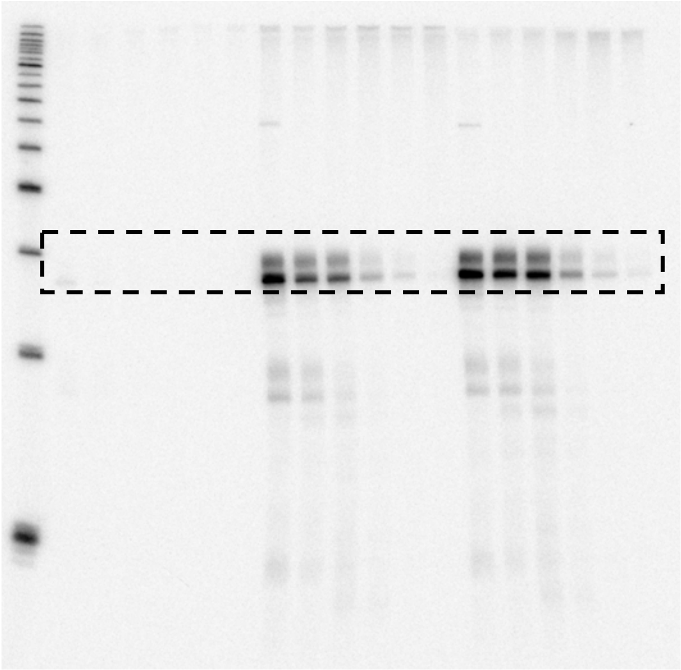

Qrr1

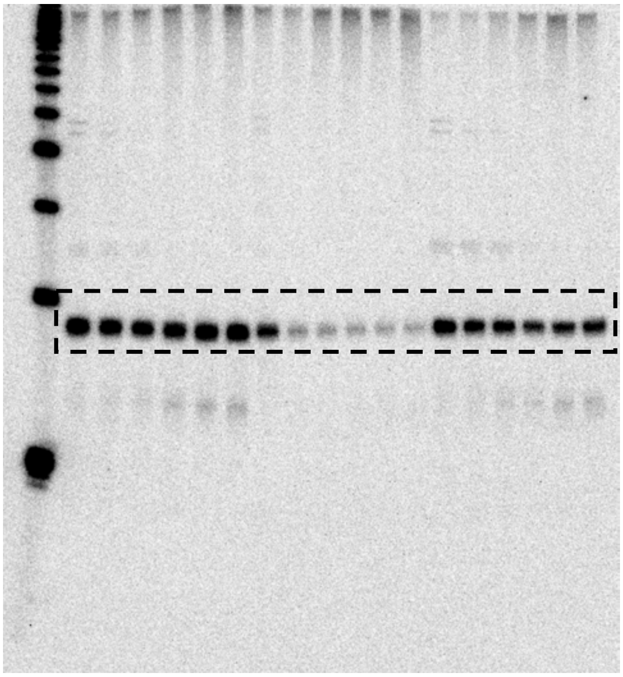

Qrr4

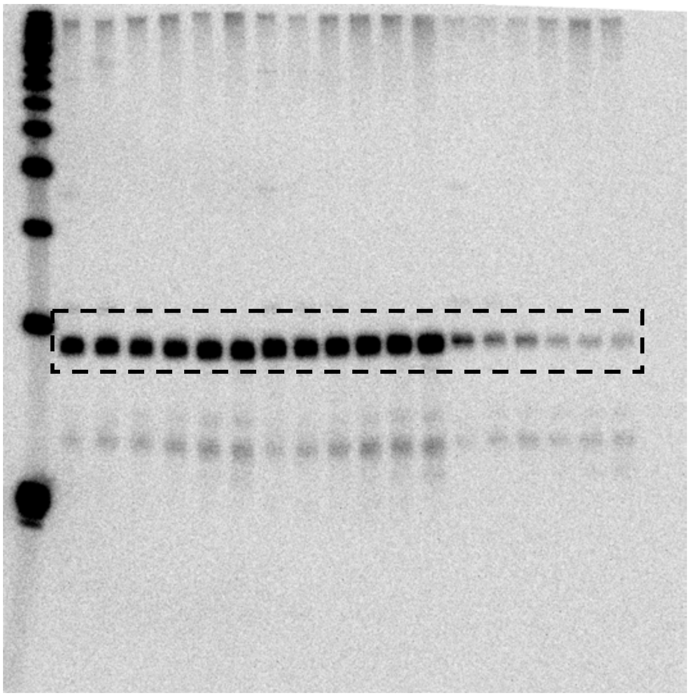

5S rRNA

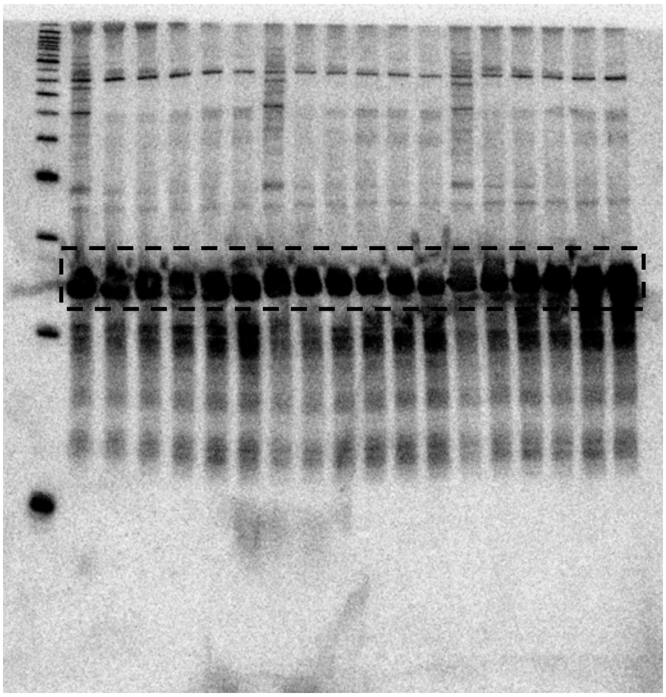

Source data for Fig. 3C

Replicate II

QrrX

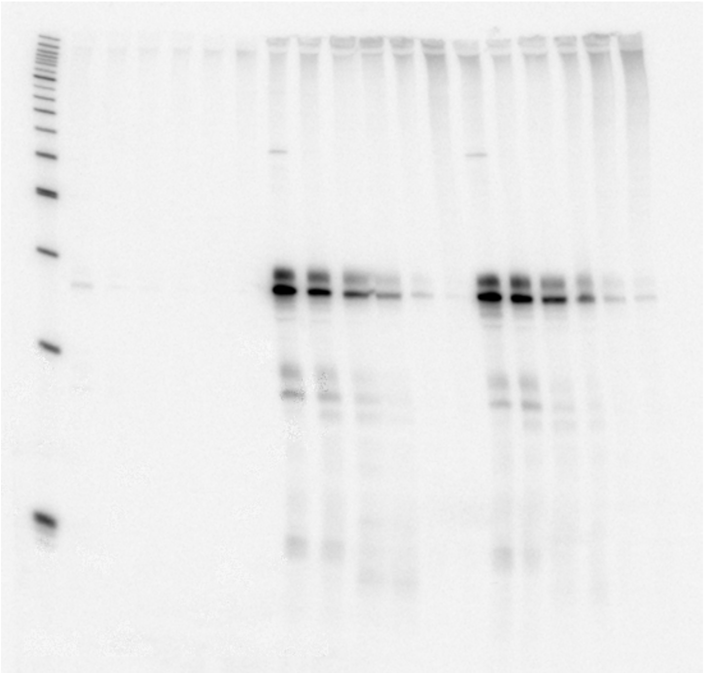

Qrr1

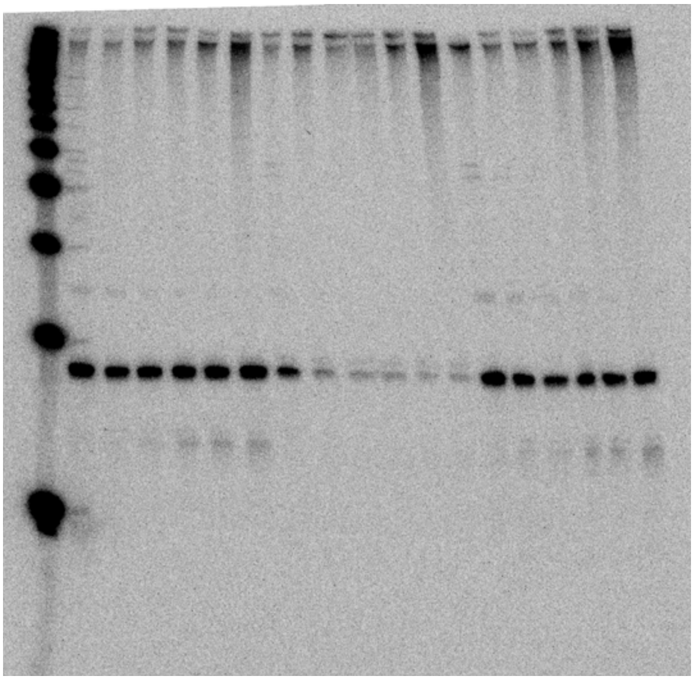

Qrr4

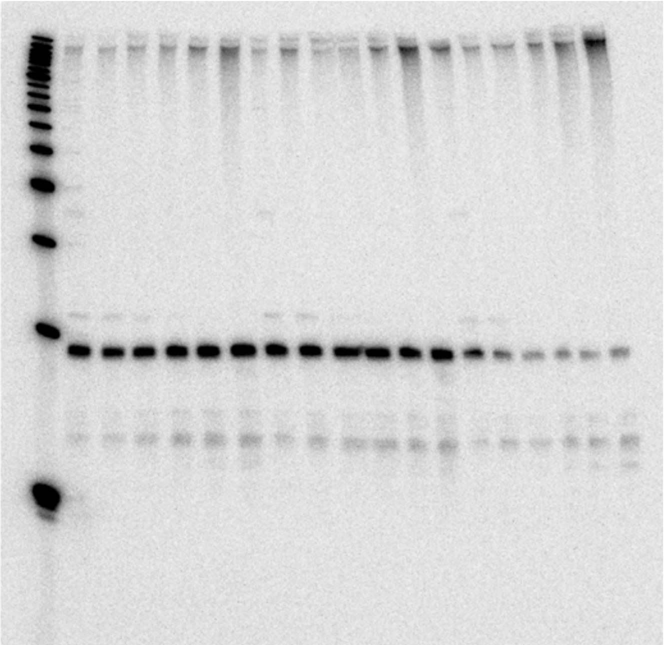

5S rRNA

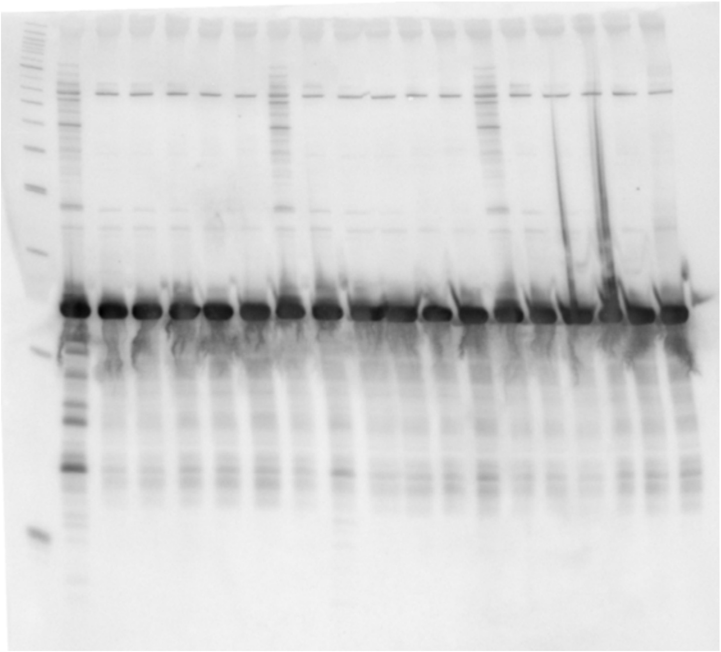

Source data for Fig. 3C

Replicate III

QrrX

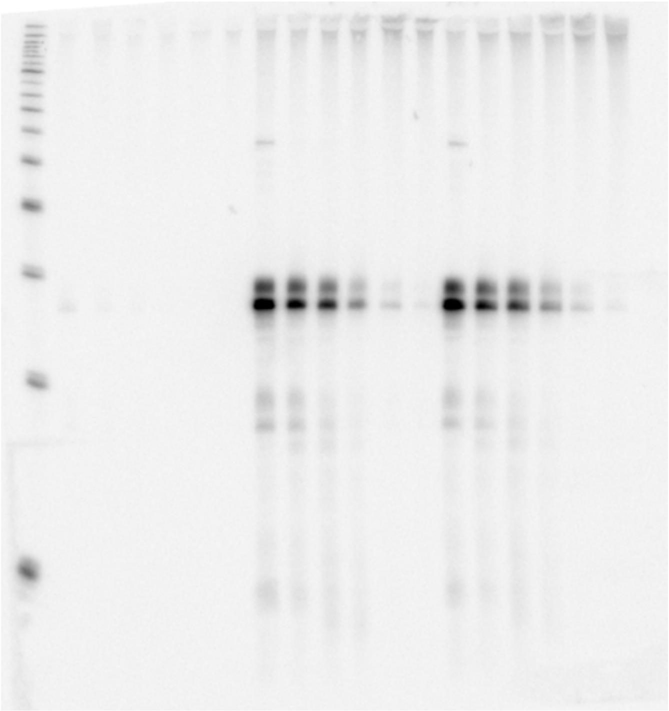

Qrr1

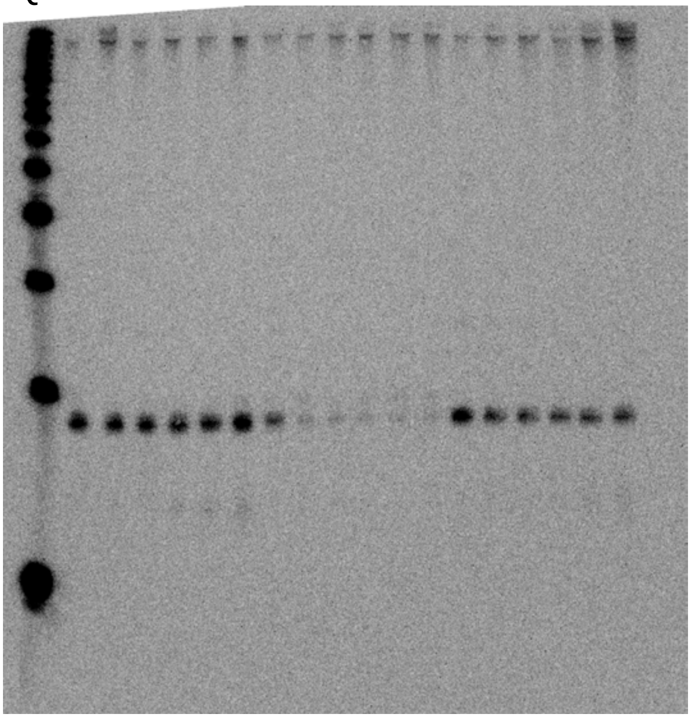

Qrr4

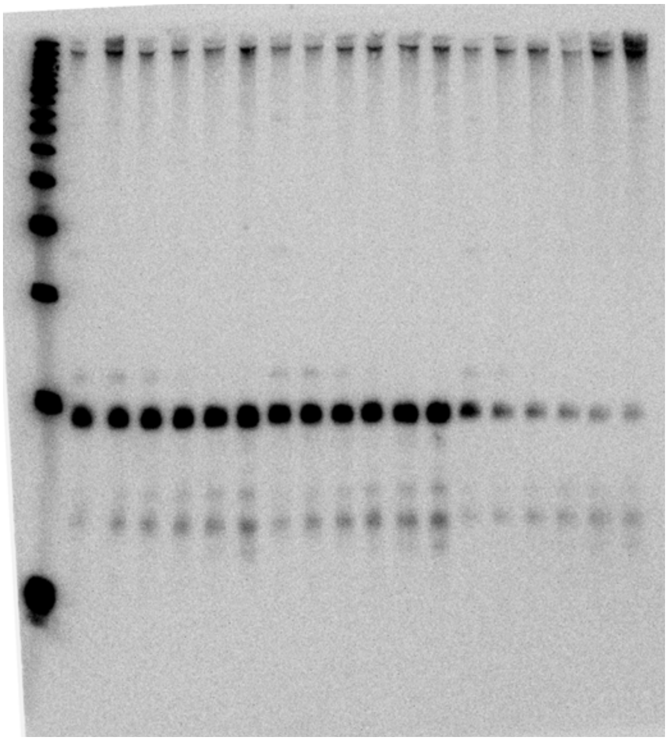

5S rRNA

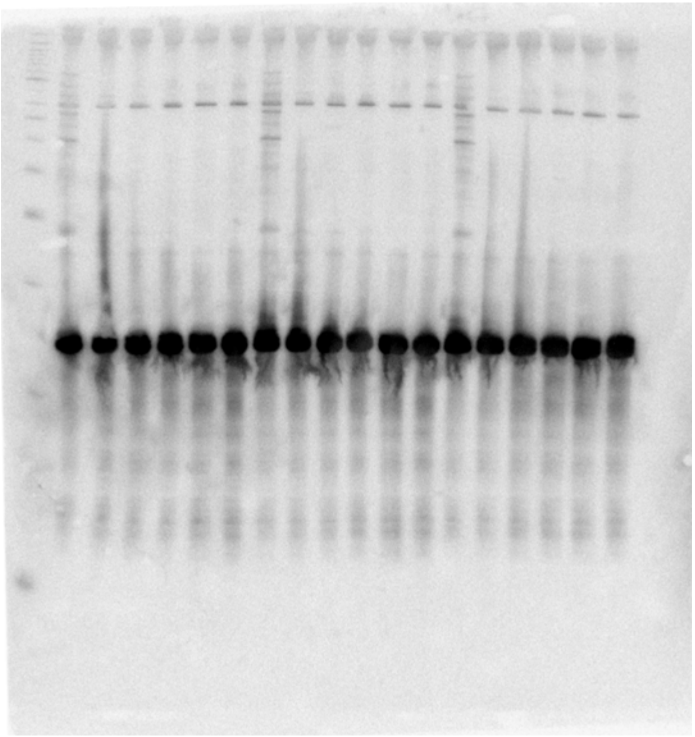

Source data for Fig. 3D

Replicate I

QrrX

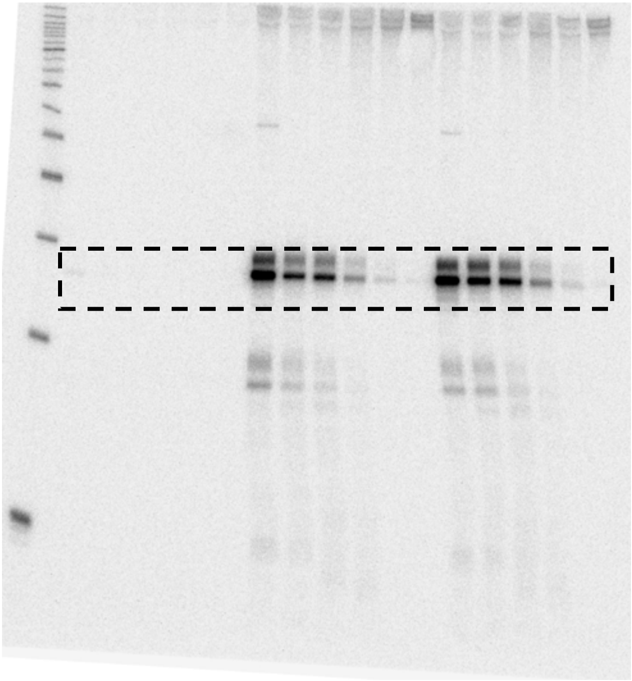

Qrr1

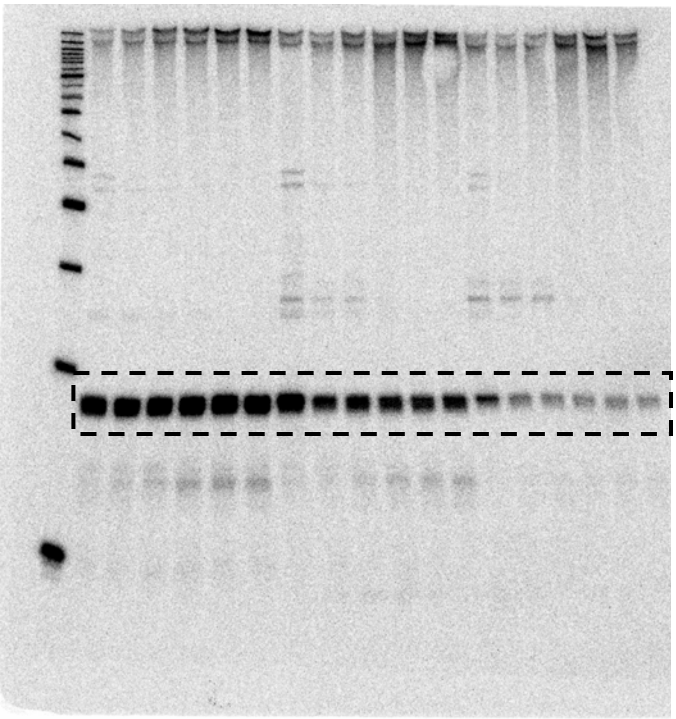

Qrr4

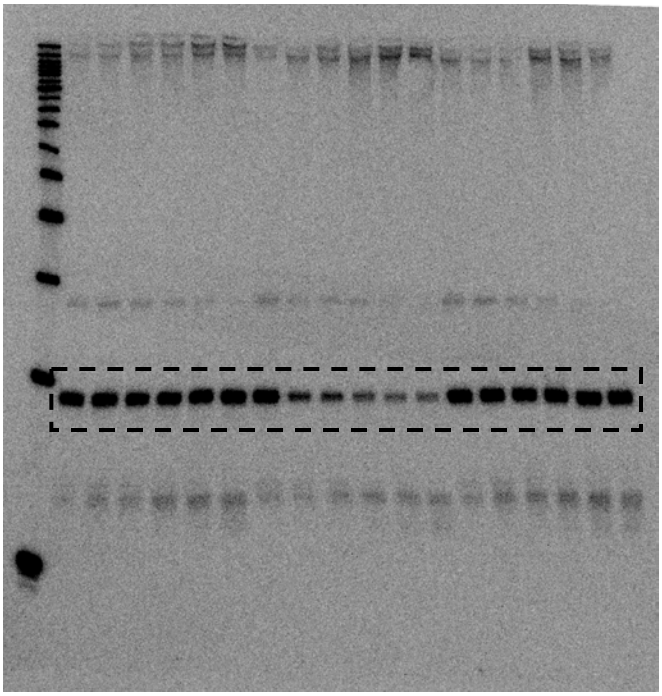

5S rRNA

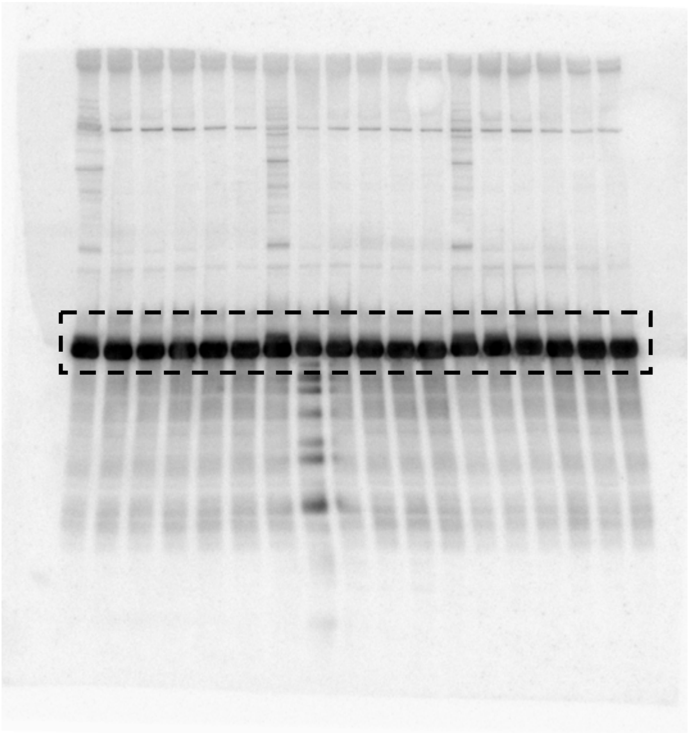

Source data for Fig. 3D

Replicate II

QrrX

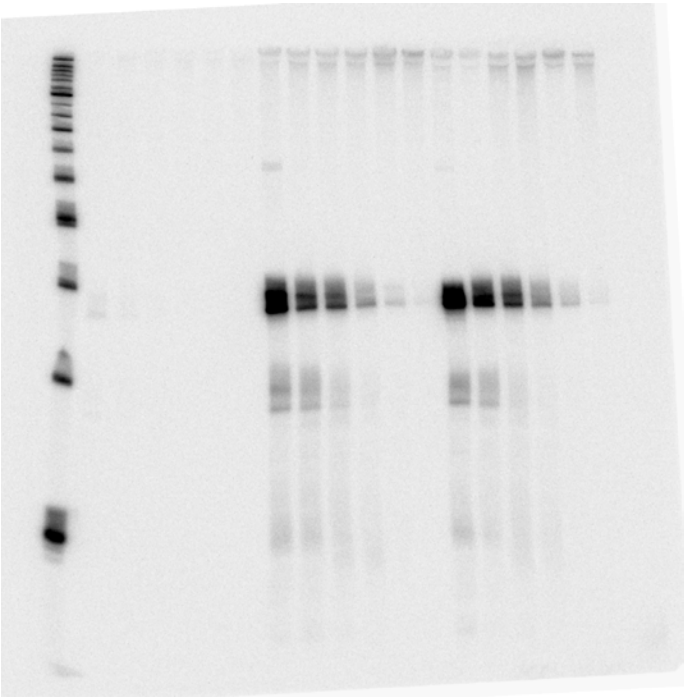

Qrr1

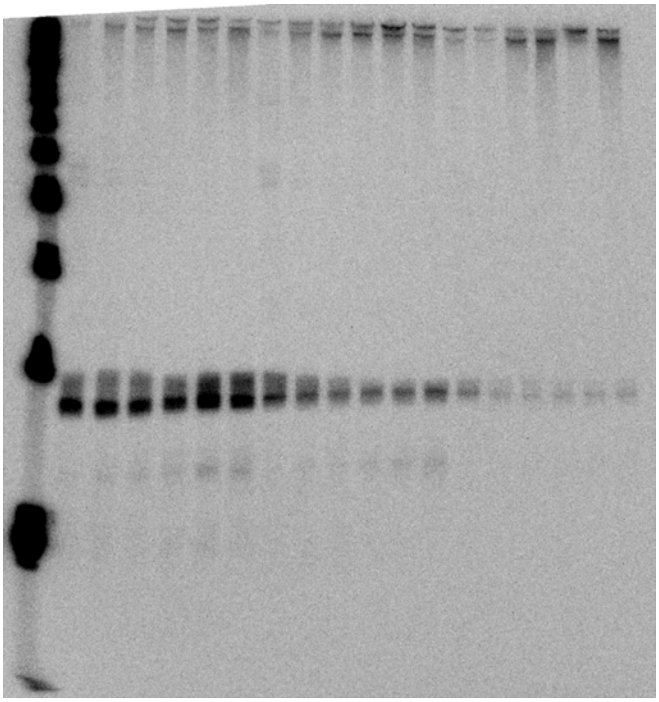

Qrr4

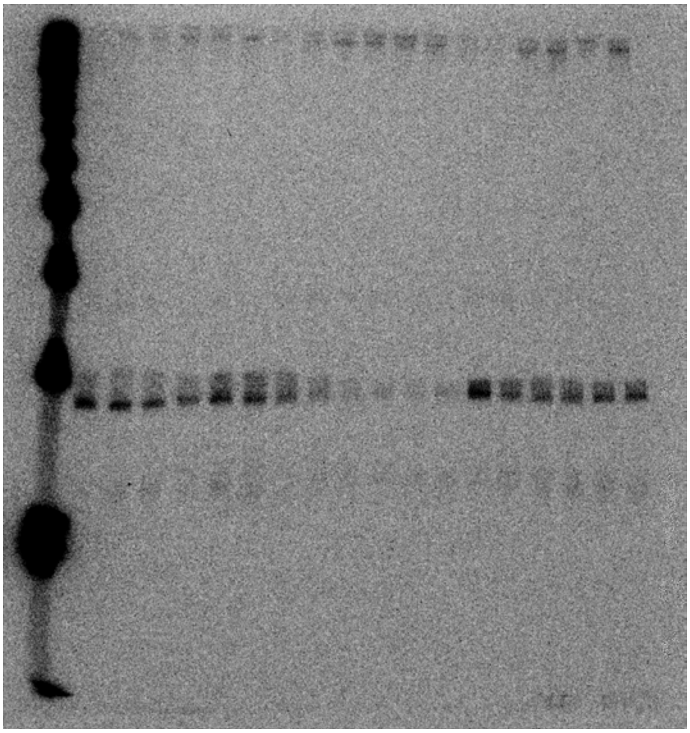

5S rRNA

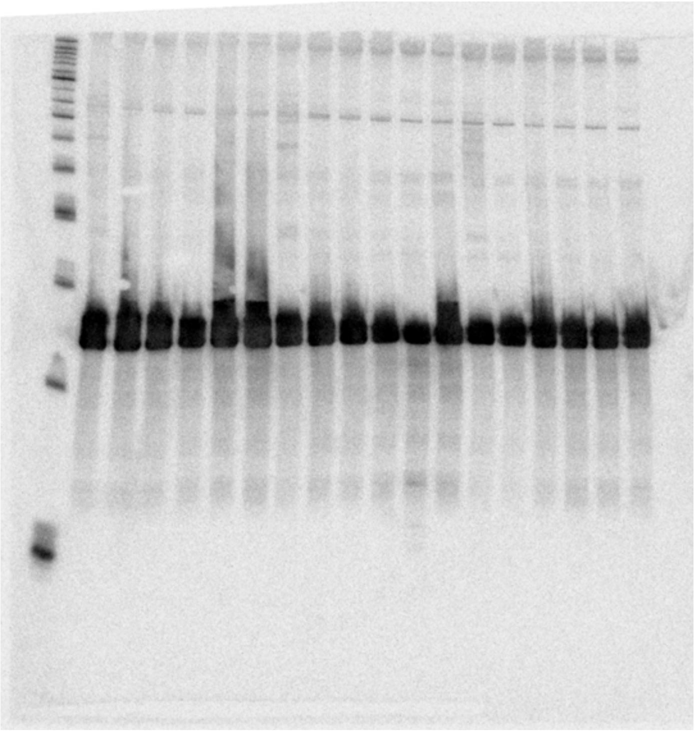

Source data for Fig. 3D

Replicate III

QrrX

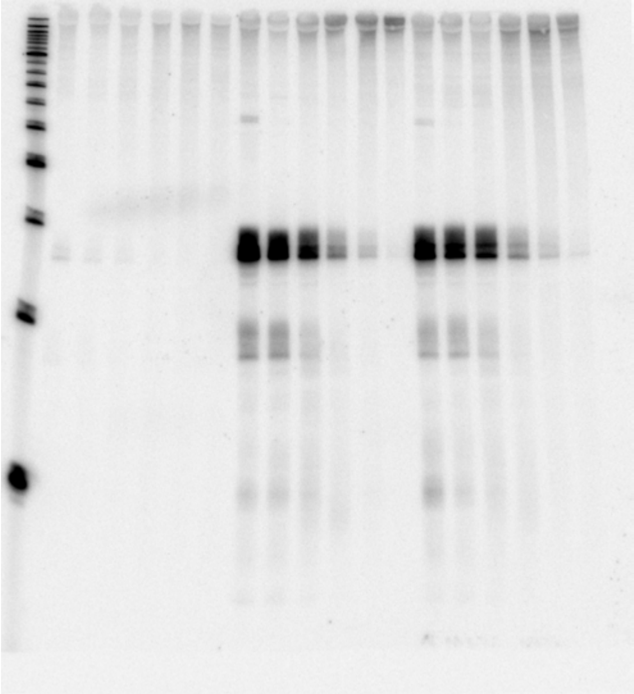

Qrr1

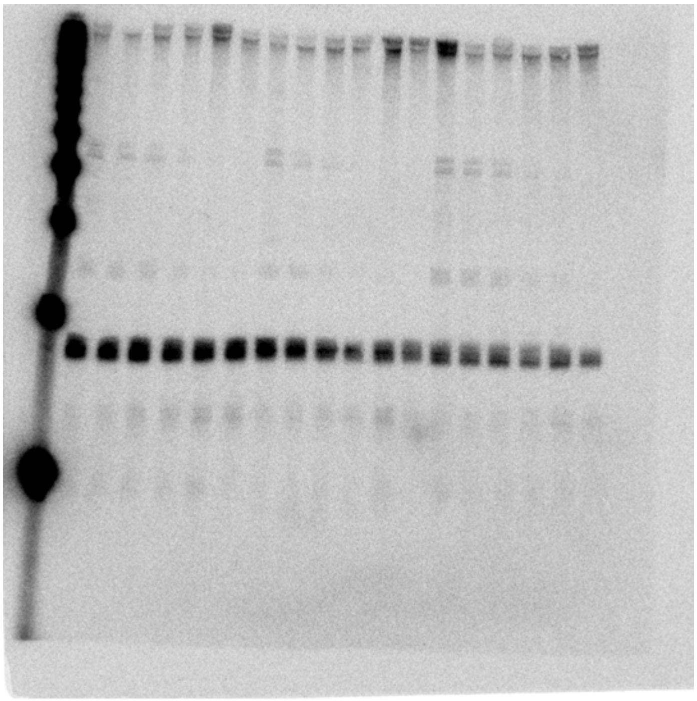

Qrr4

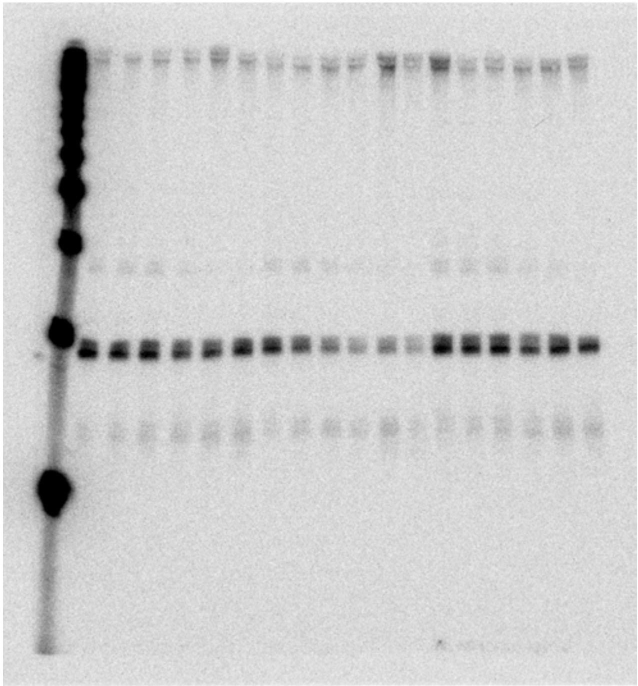

5S rRNA

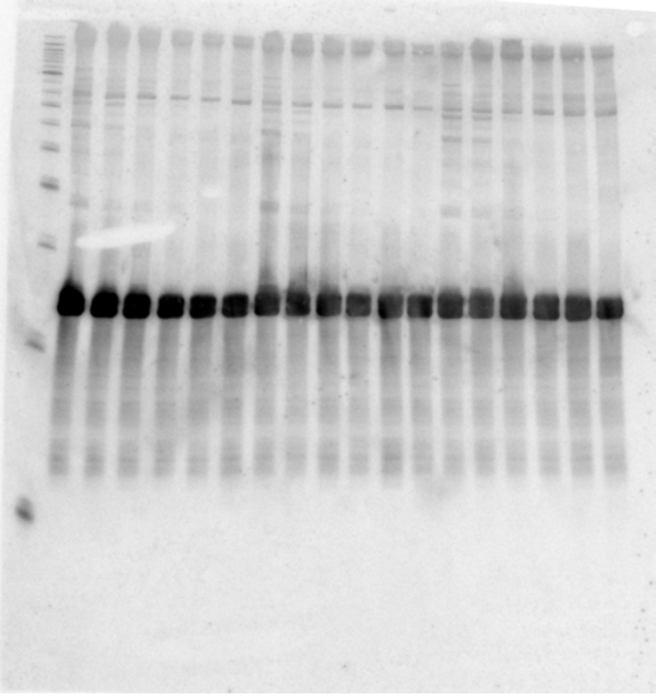

Source data for Fig. 4A

Replicate I

QrrX

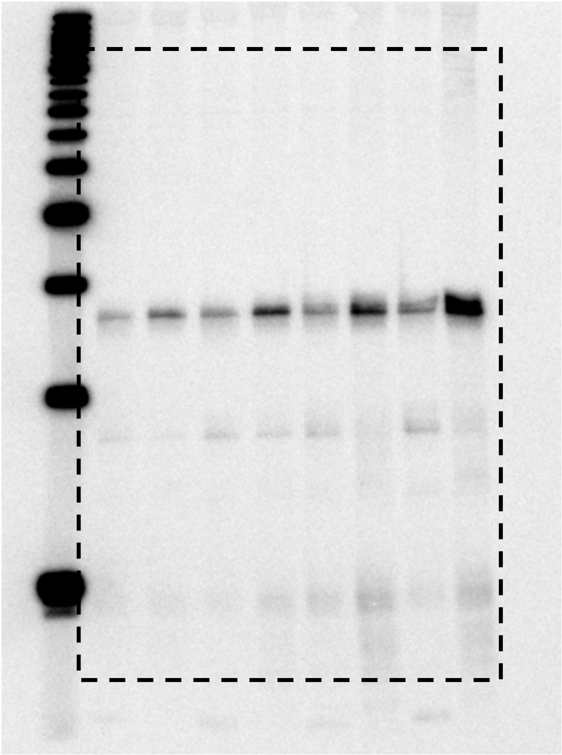

Qrr1

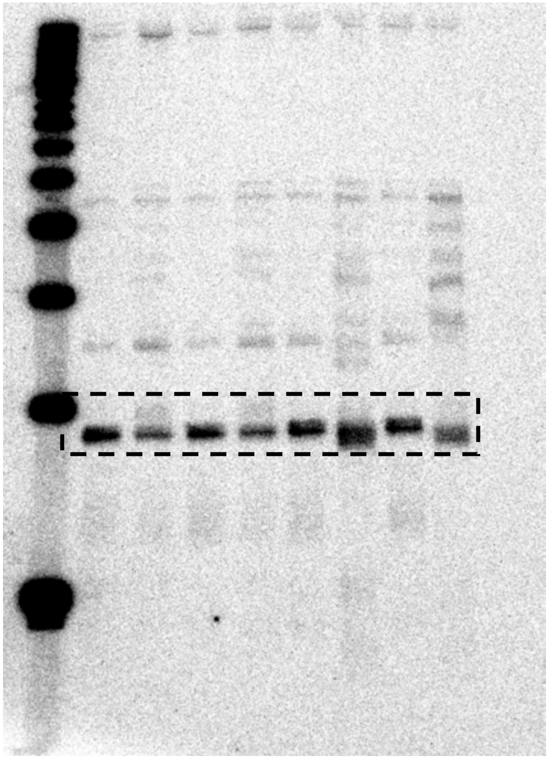

Qrr4

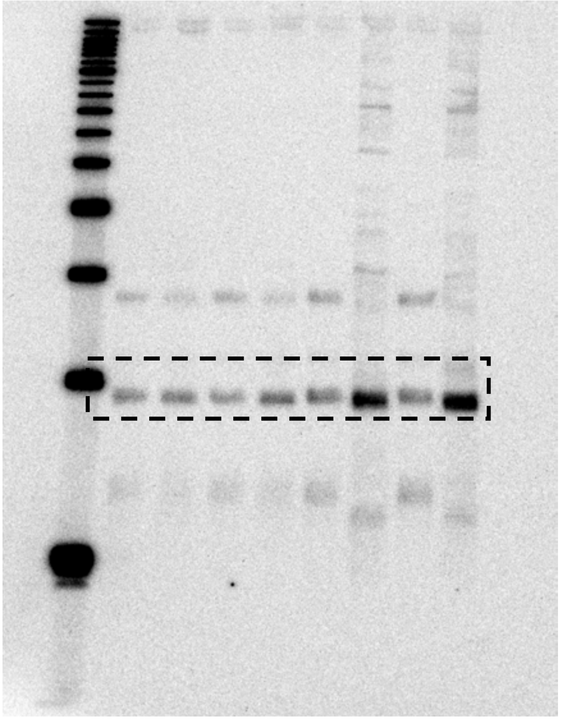

5S rRNA

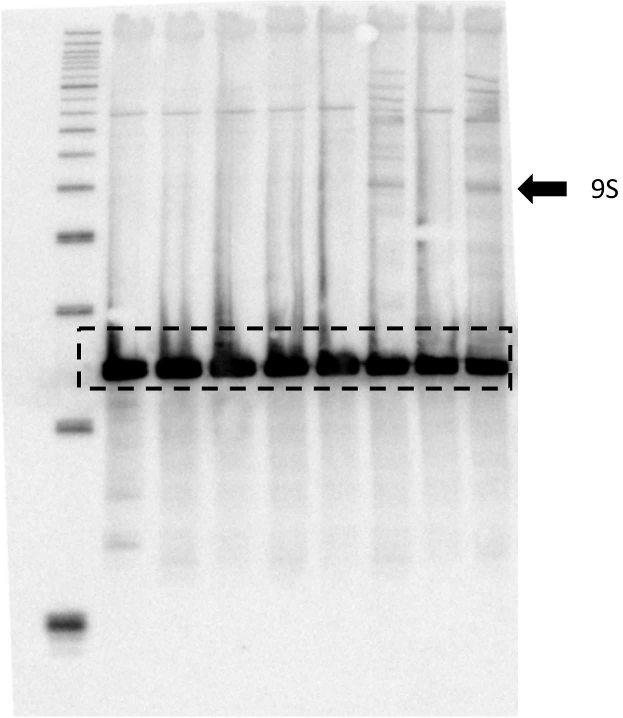

Source data for Fig. 4A

Replicate II

QrrX

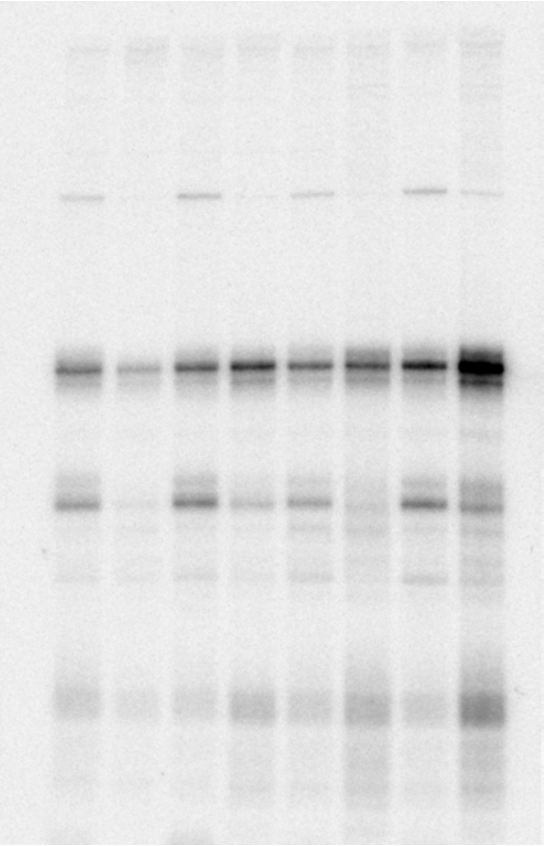

Qrr1

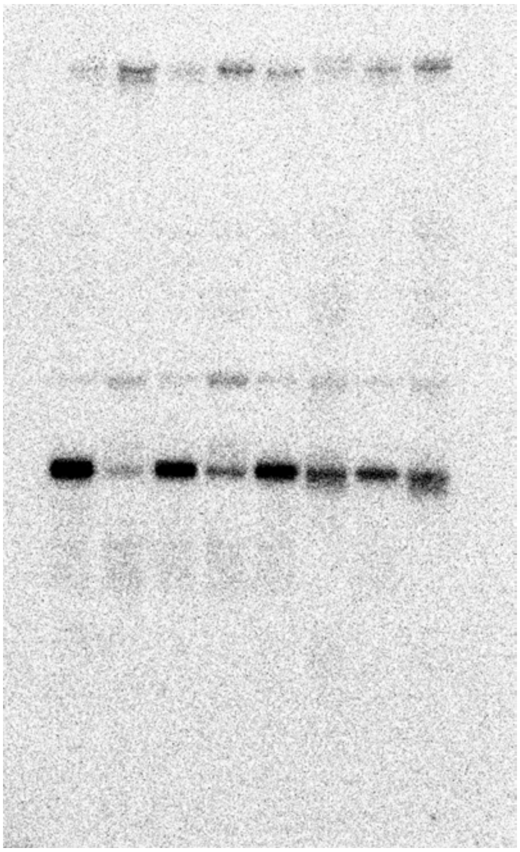

Qrr4

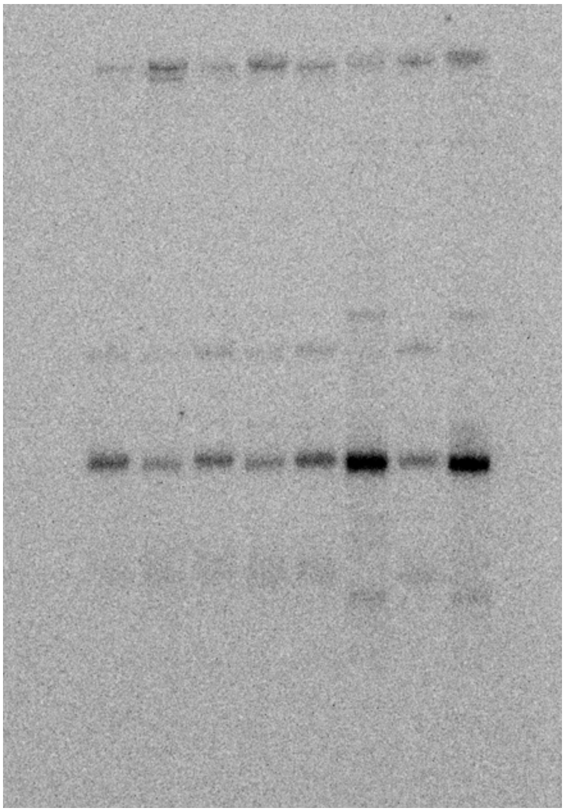

5S rRNA

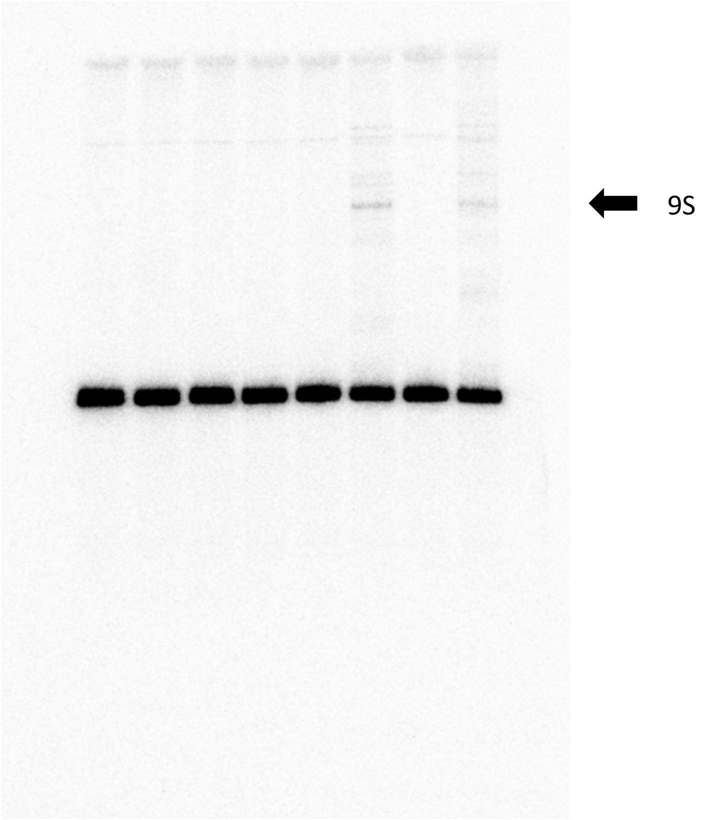

Source data for Fig. 4A

Replicate III

QrrX

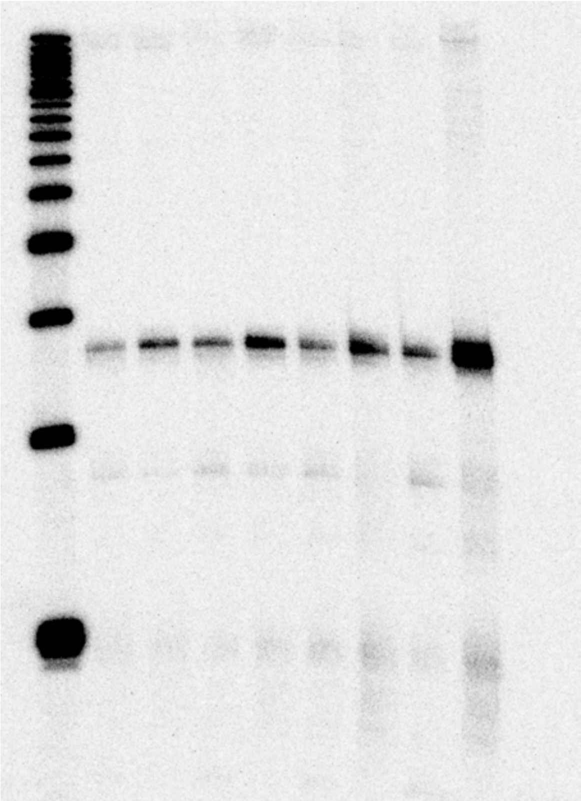

Qrr1

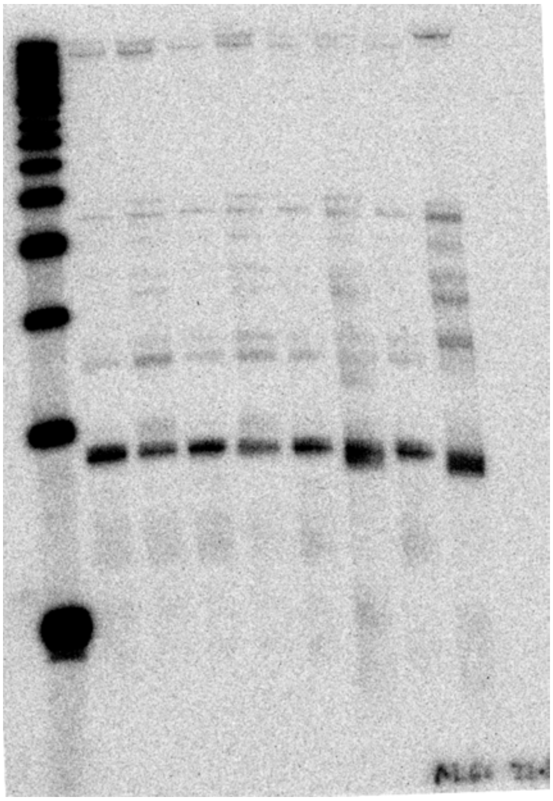

Qrr4

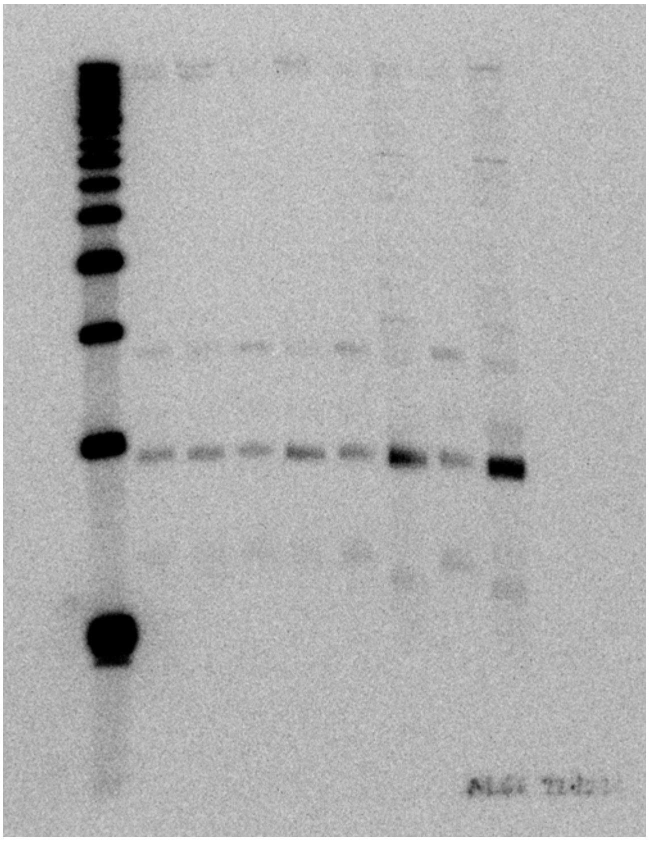

5S rRNA

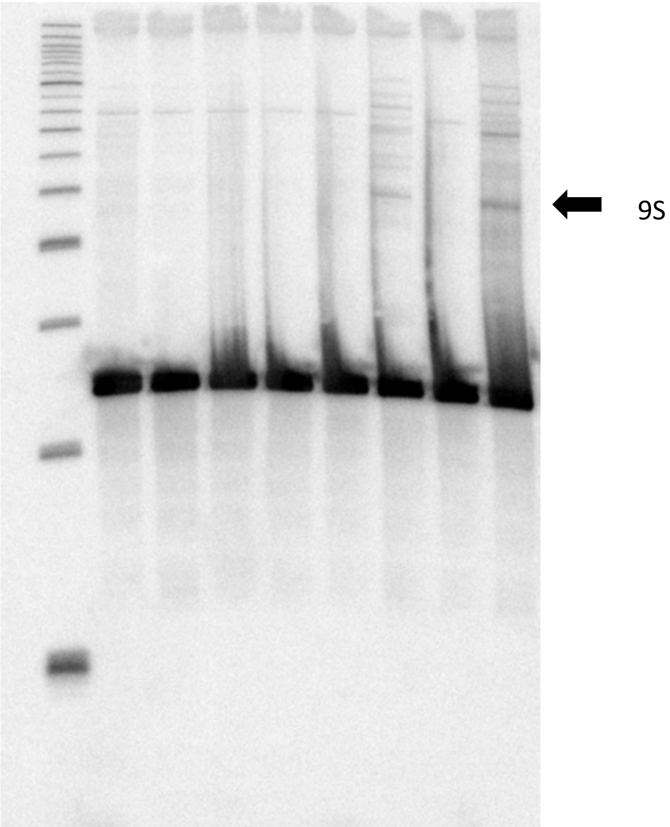

Source data for Fig. 4B

Replicate I

QrrX

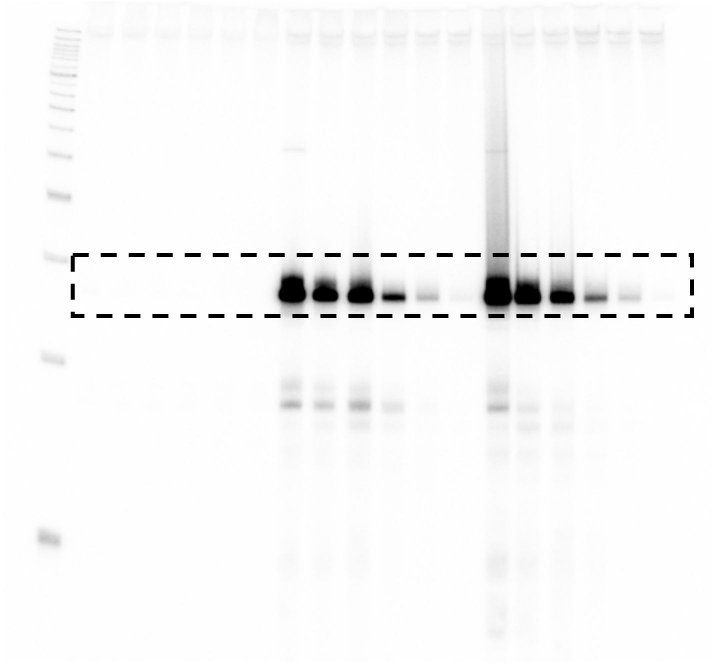

Qrr4

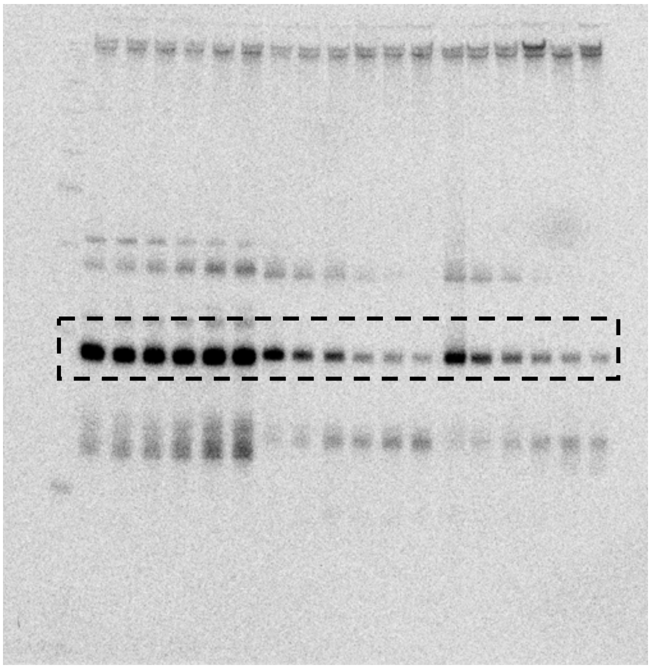

5S rRNA

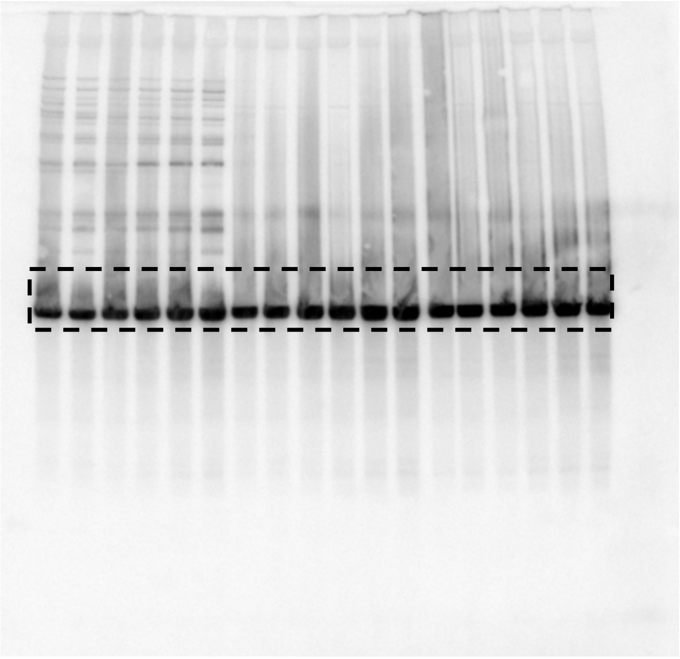

Source data for Fig. 4B

Replicate II

QrrX

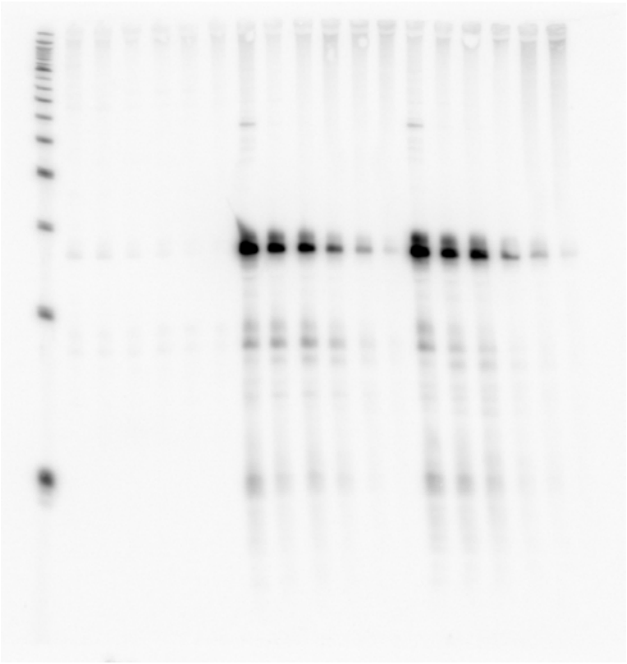

Qrr4

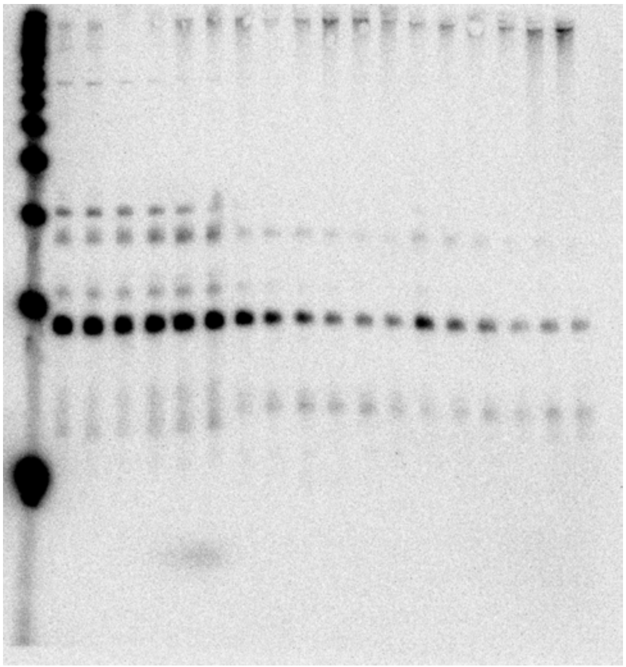

5S rRNA

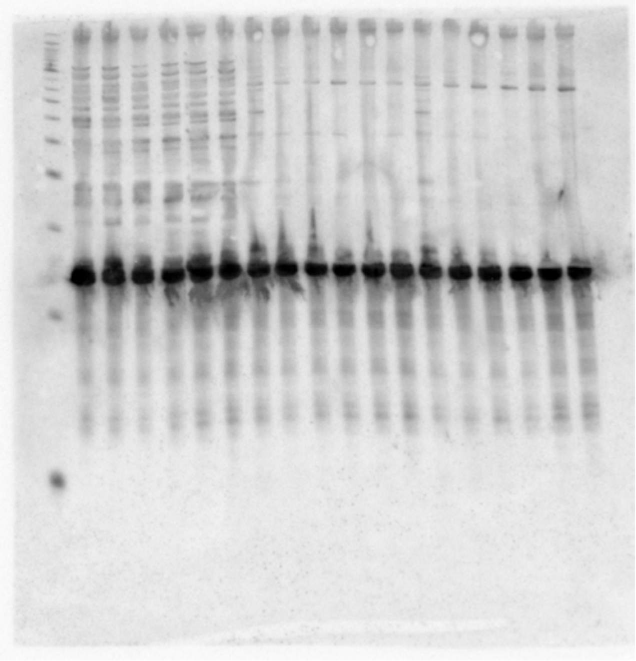

Source data for Fig. 4B

Replicate III

QrrX

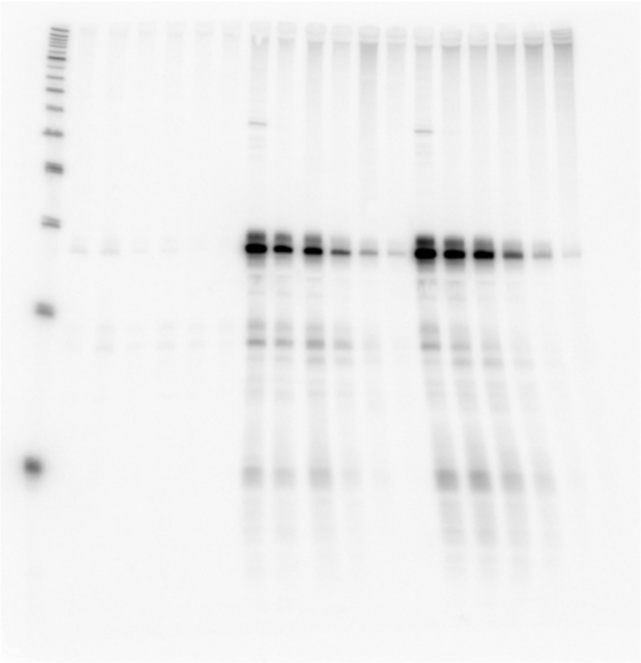

Qrr4

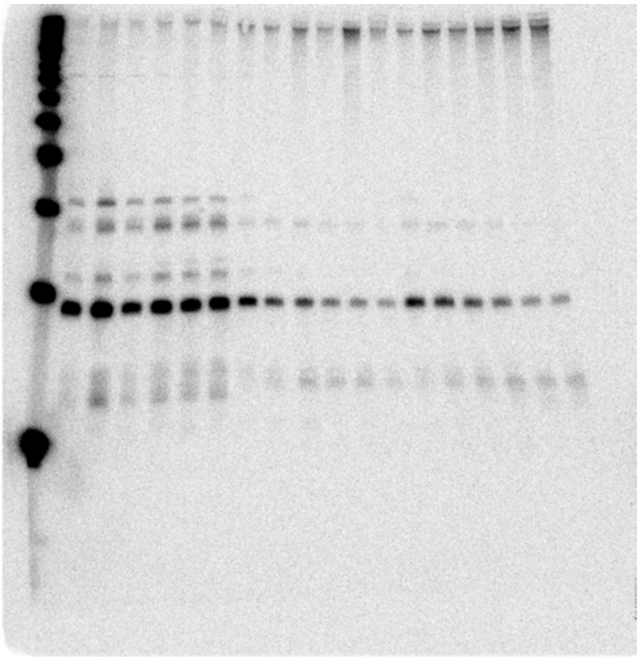

5S rRNA

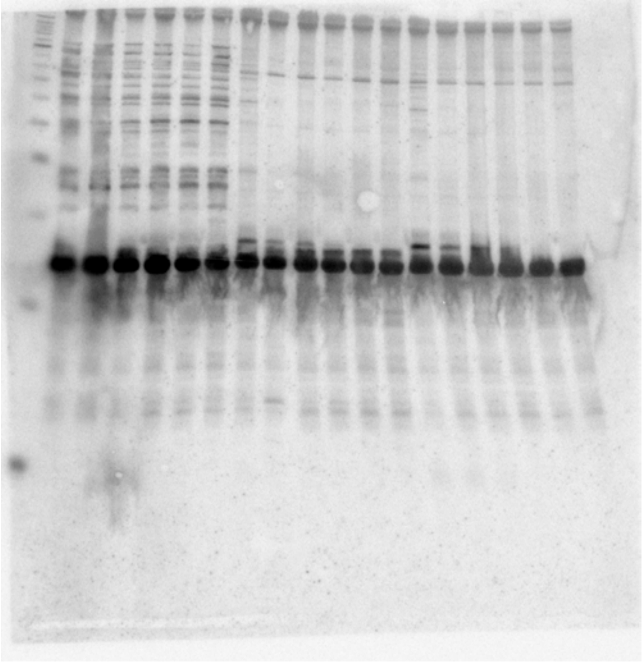

Source data for Fig. 4C

Replicate I

QrrX

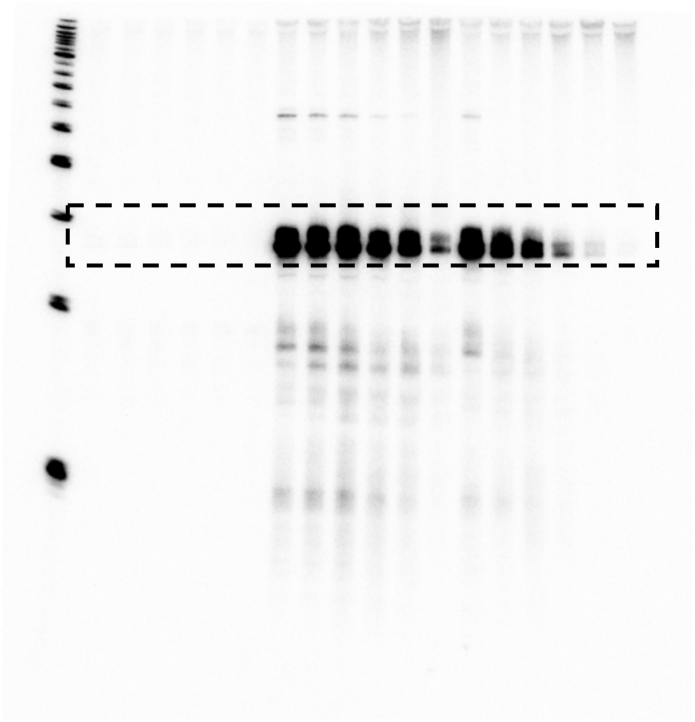

Qrr4

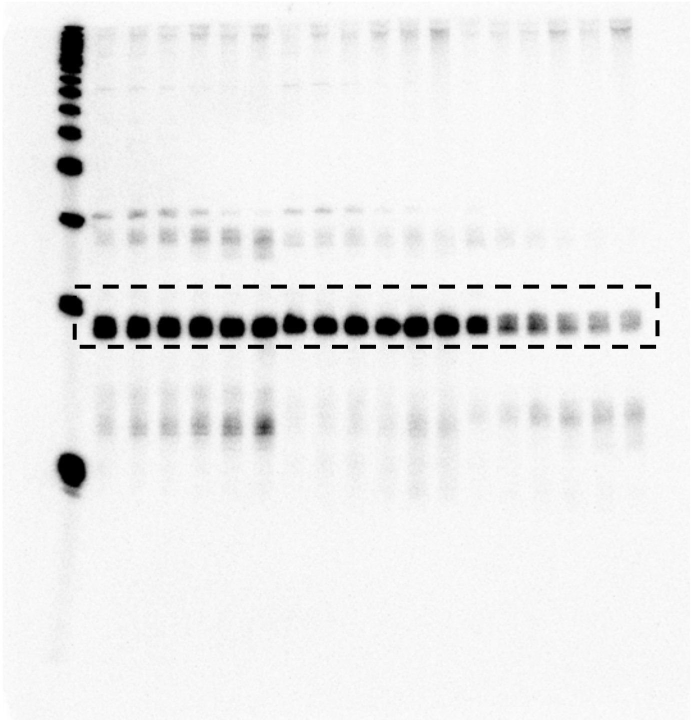

5S rRNA

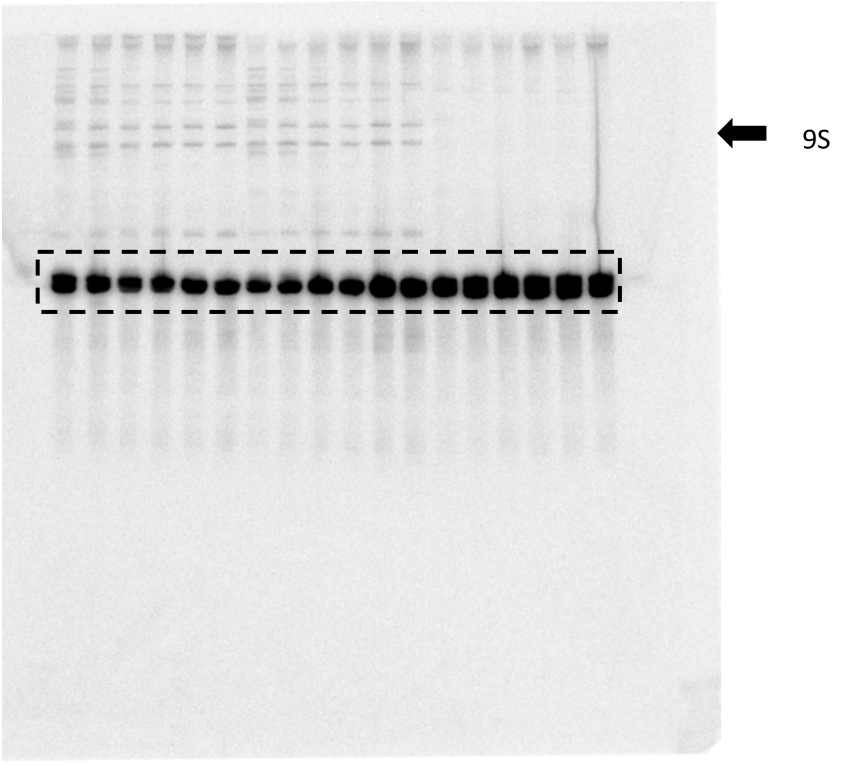

Source data for Fig. 4C

Replicate II

QrrX

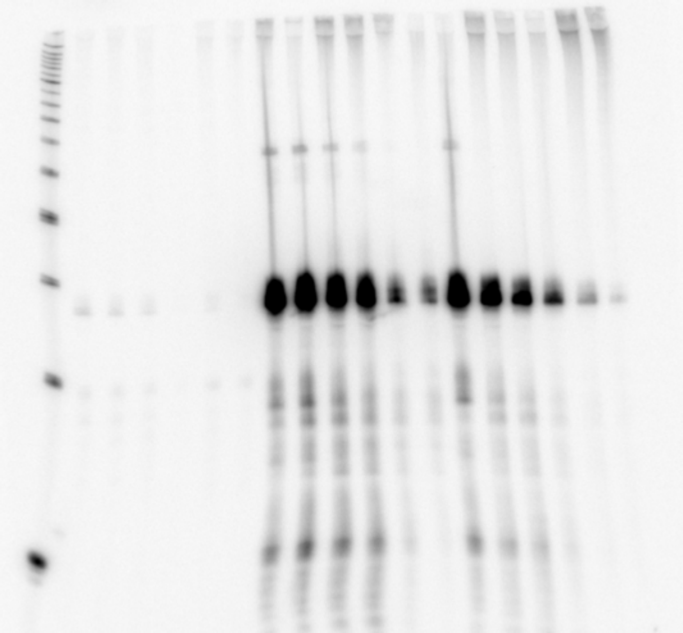

Qrr4

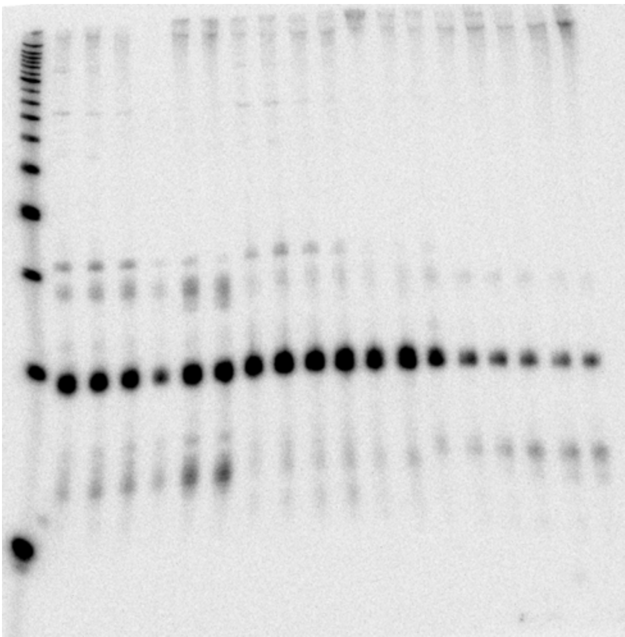

5S rRNA

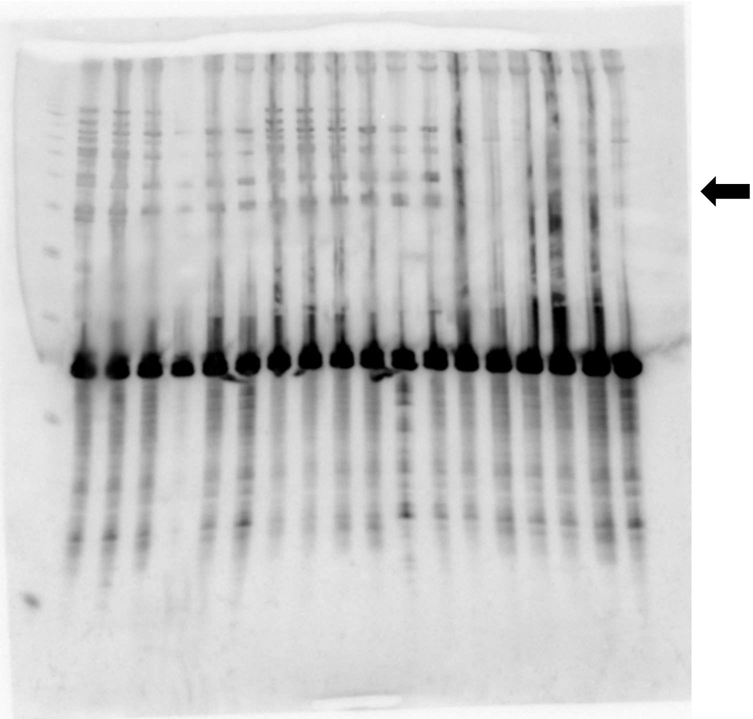

9S

Source data for Fig. 4C

Replicate III

QrrX

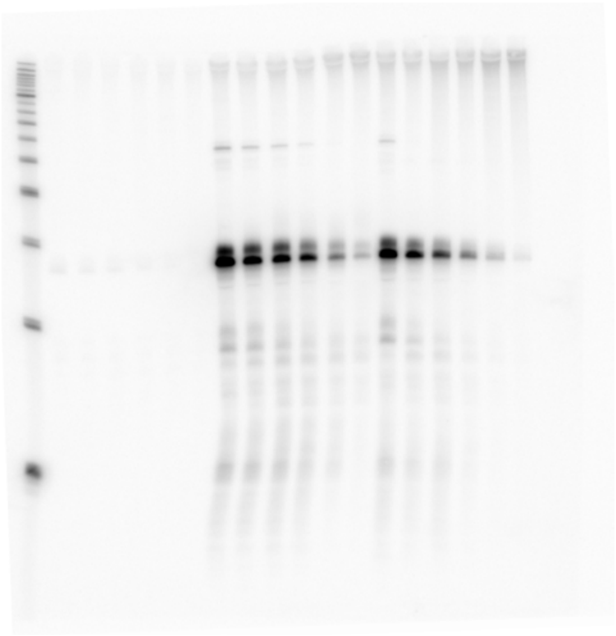

Qrr4

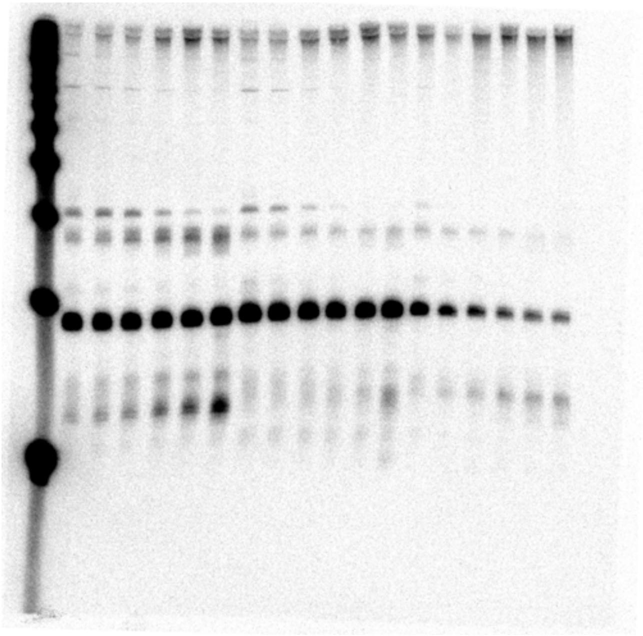

5S rRNA

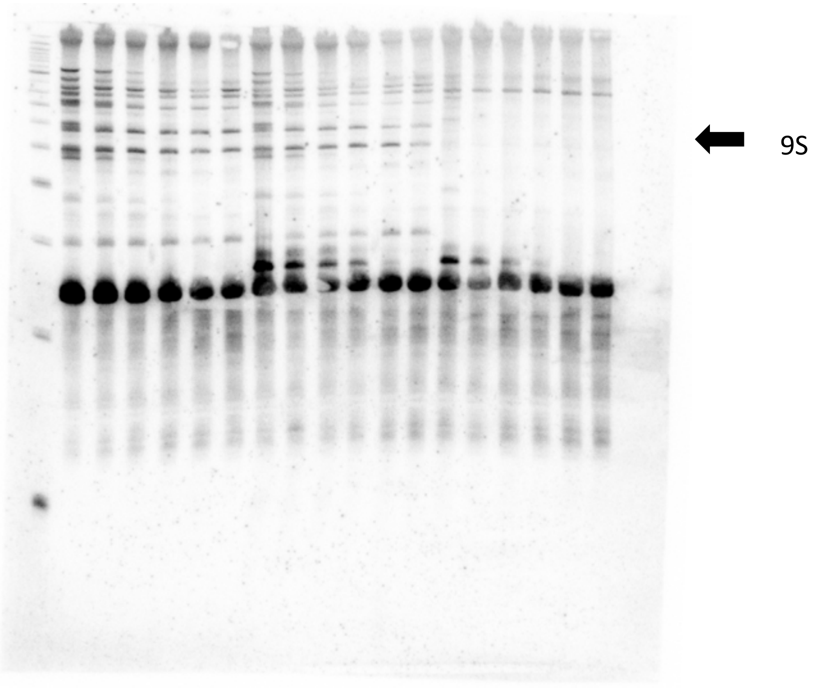

Source data for Fig. 5B

Replicate I

QrrX

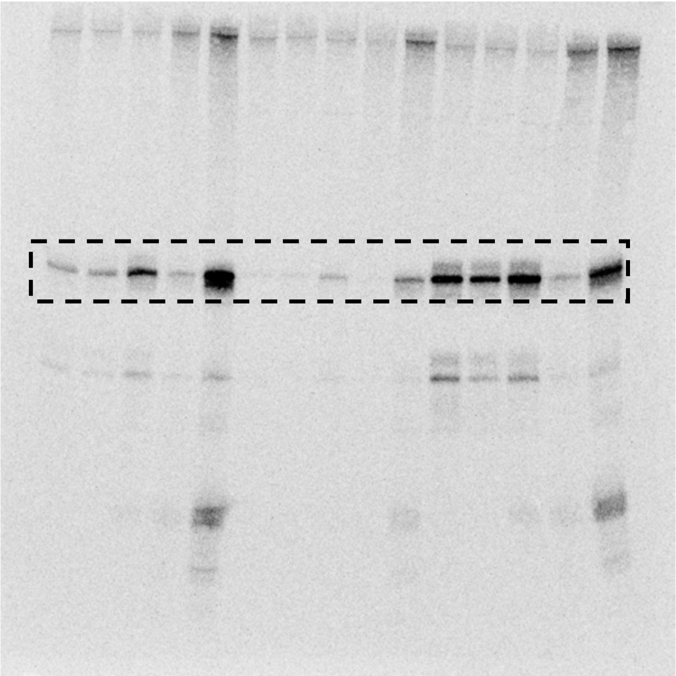

Qrr1

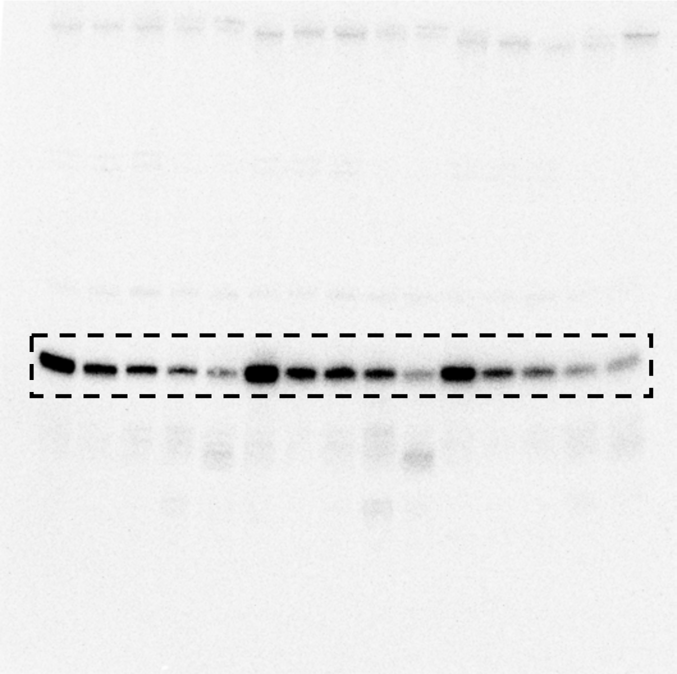

Qrr2

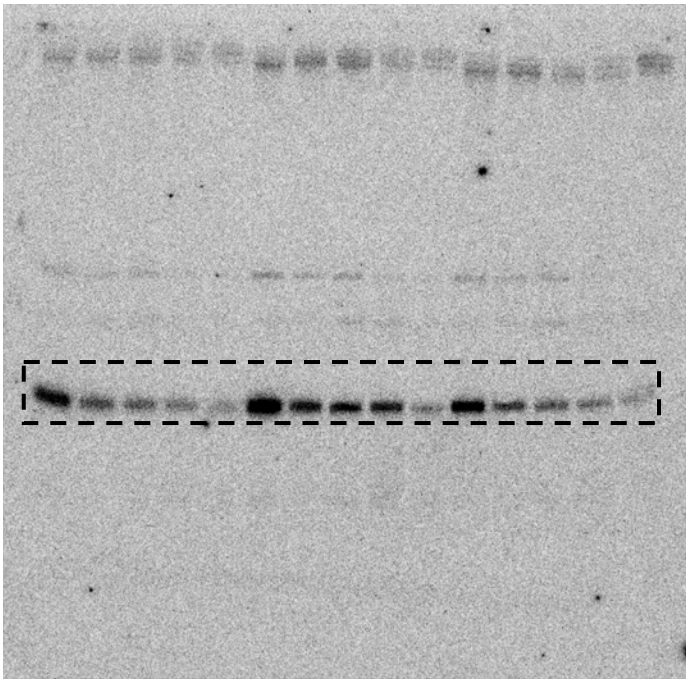

Qrr3

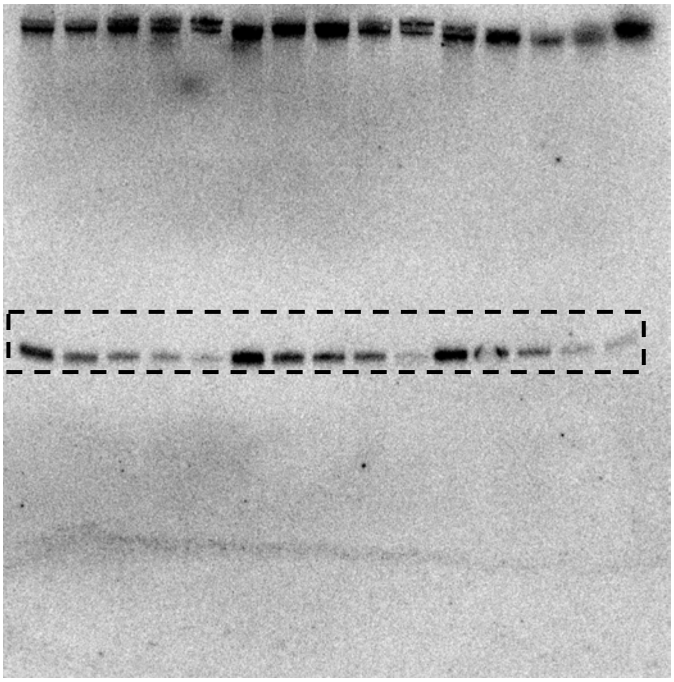

Source data for Fig. 5B

Replicate I

Qrr4

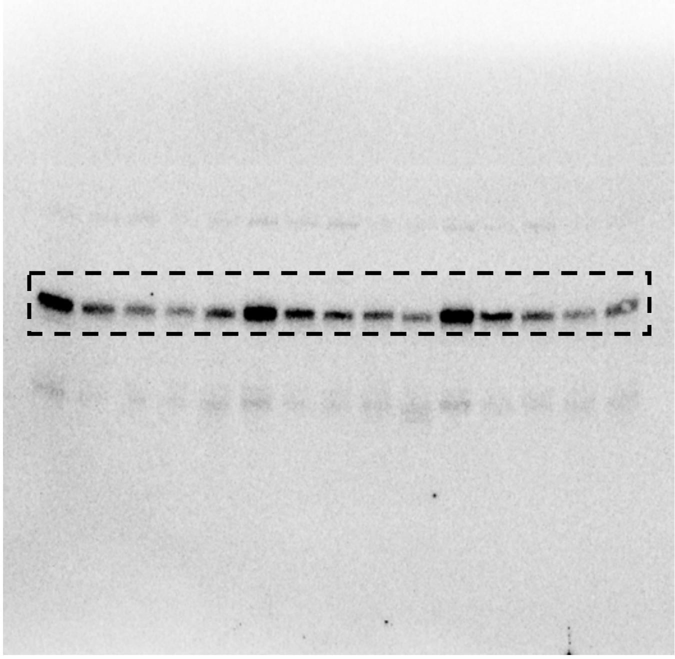

5S rRNA

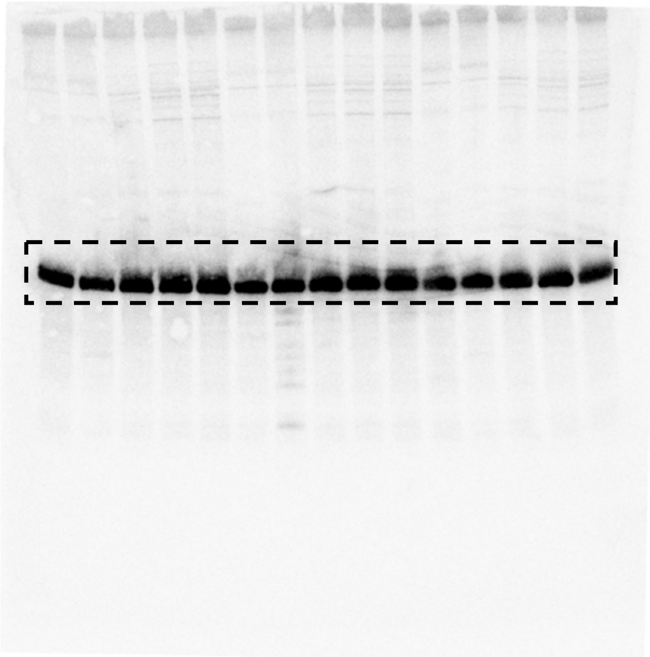

Source data for Fig. 5B

Replicate II

QrrX

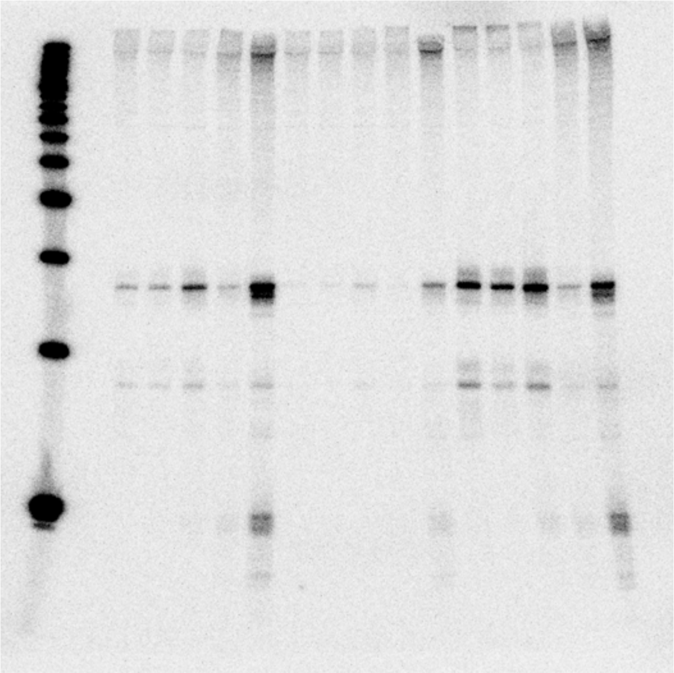

Qrr1

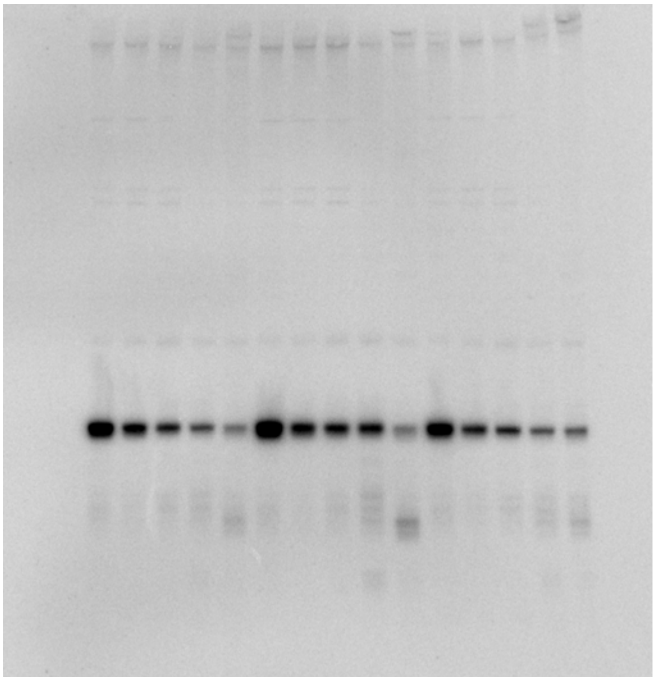

Qrr2

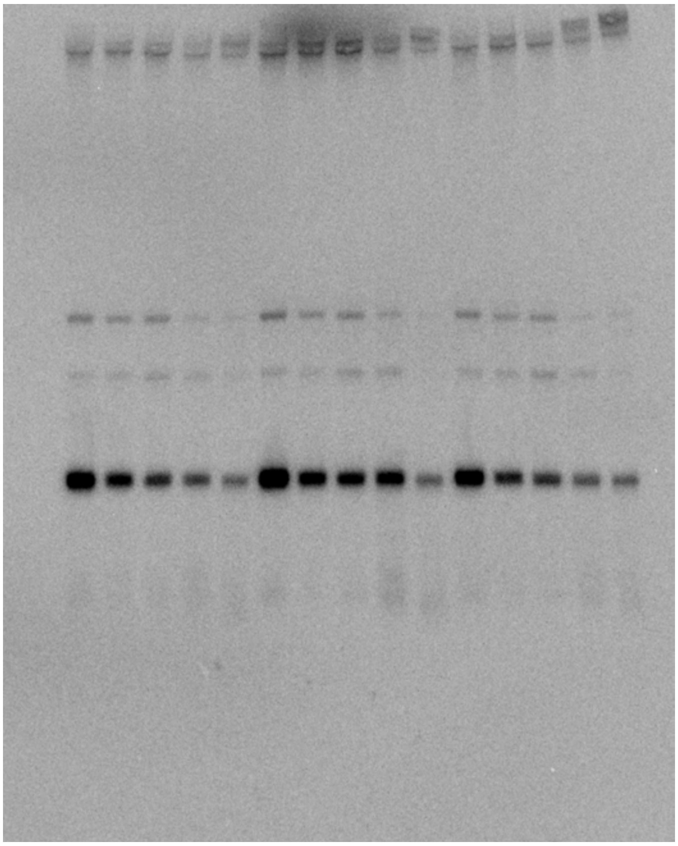

Qrr3

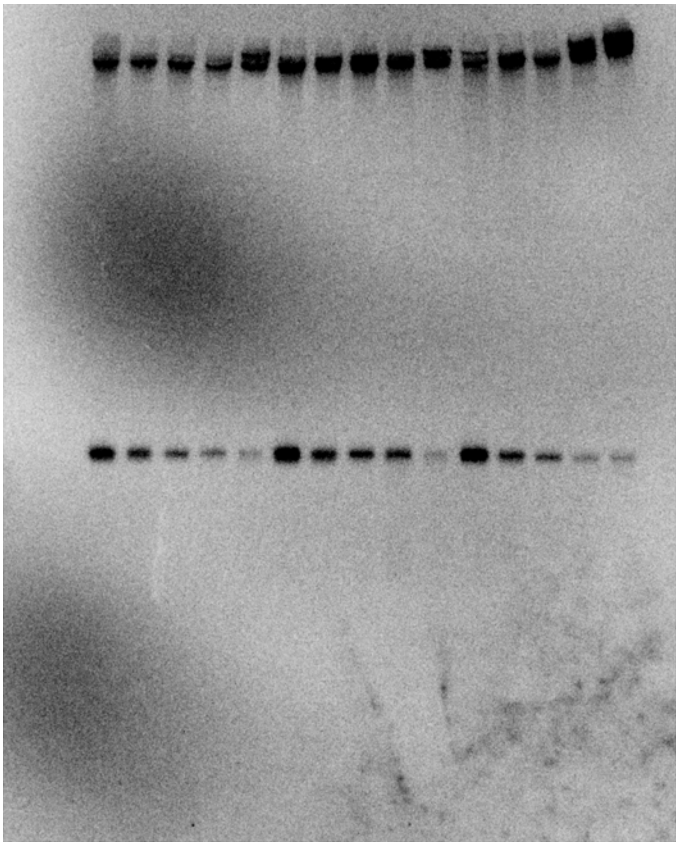

Source data for Fig. 5B

Replicate II

Qrr4

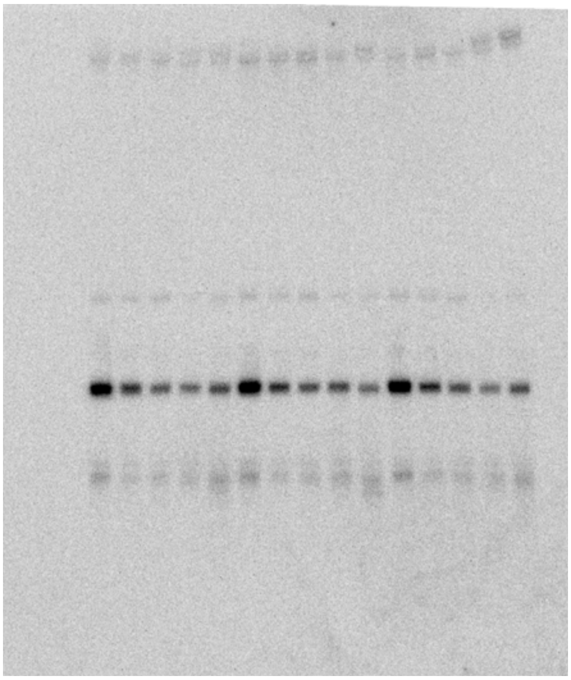

5S rRNA

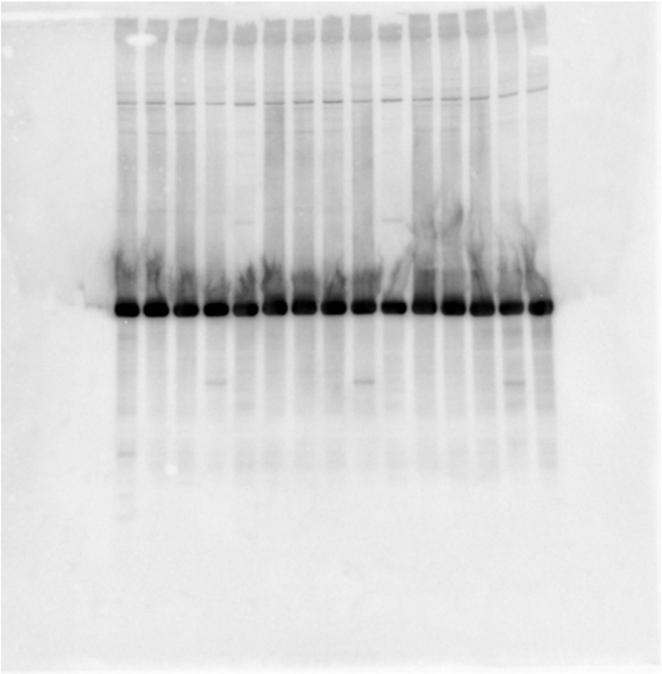

Source data for Fig. 5B

Replicate III

QrrX

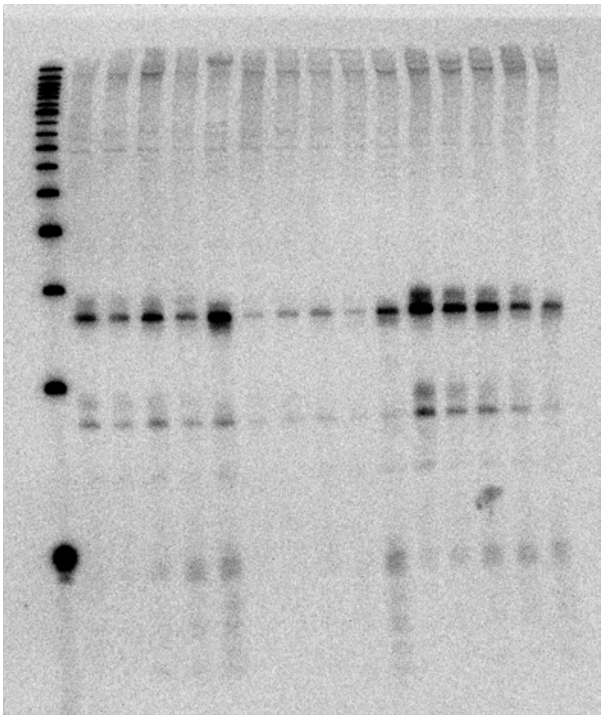

Qrr1

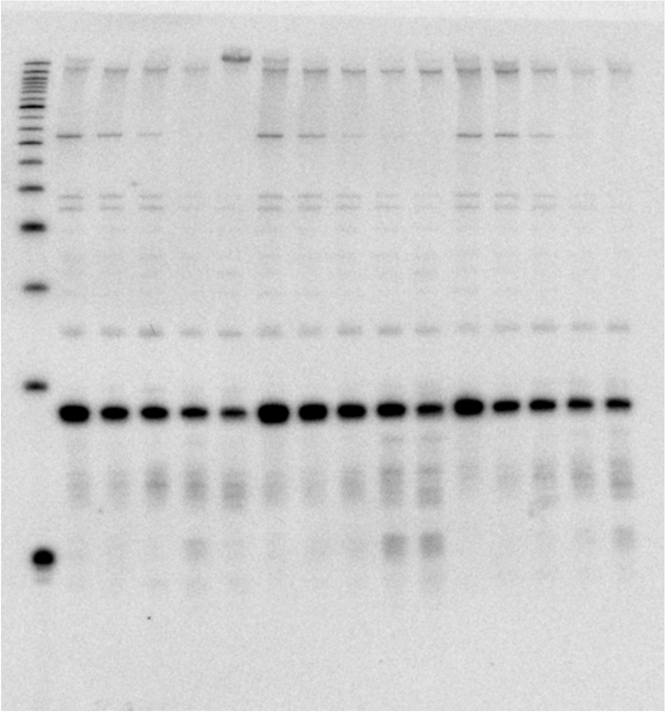

Qrr2

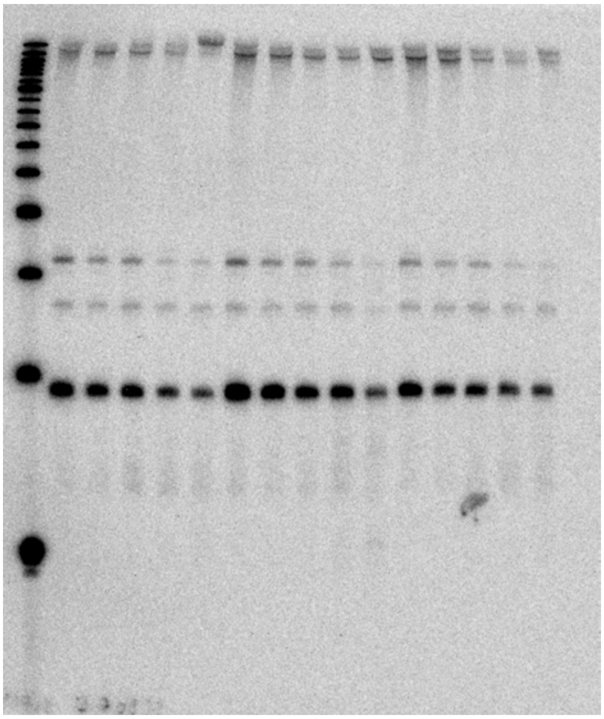

Qrr3

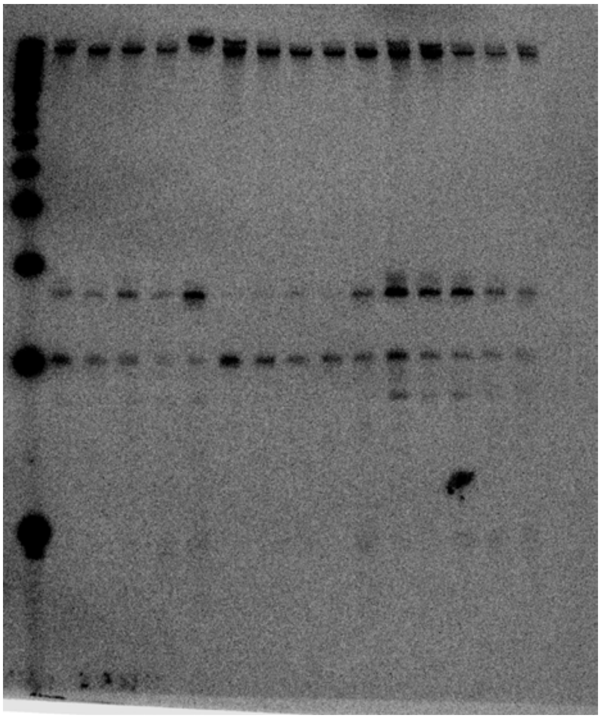

Source data for Fig. 5B

Replicate III

Qrr4

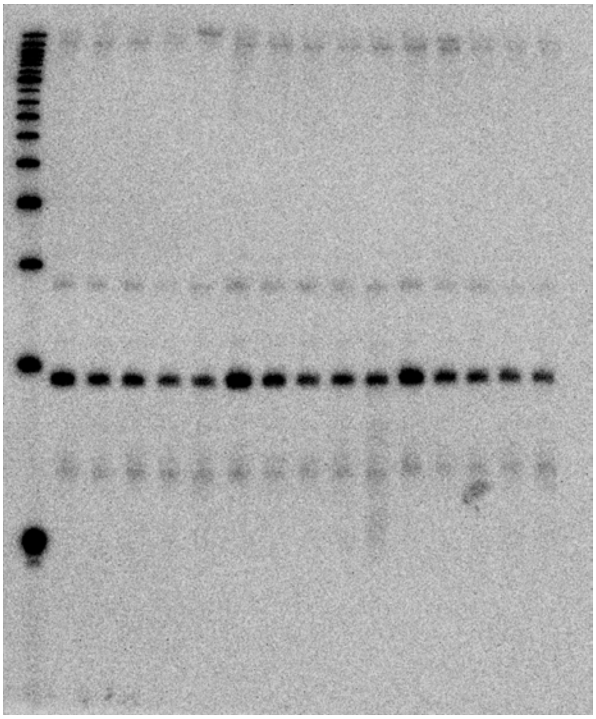

5S rRNA

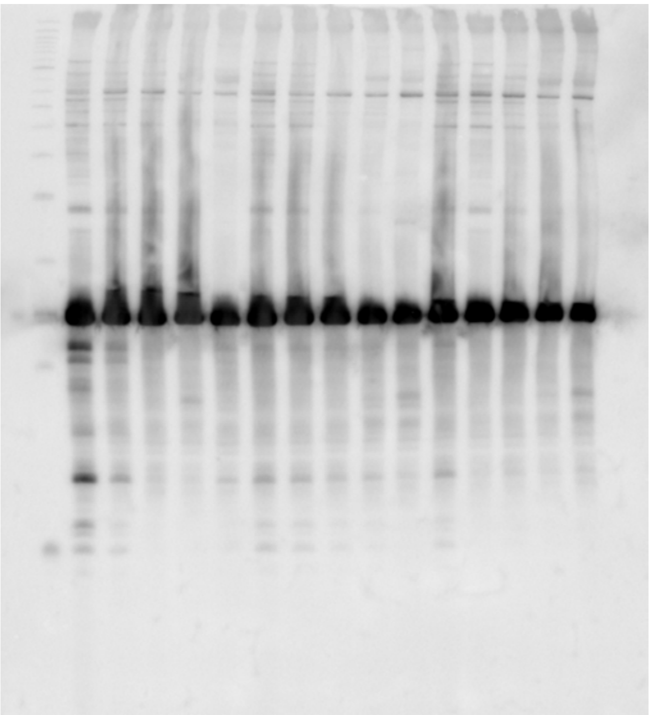

Source data for Fig. 6A

Replicate I – Western Blot

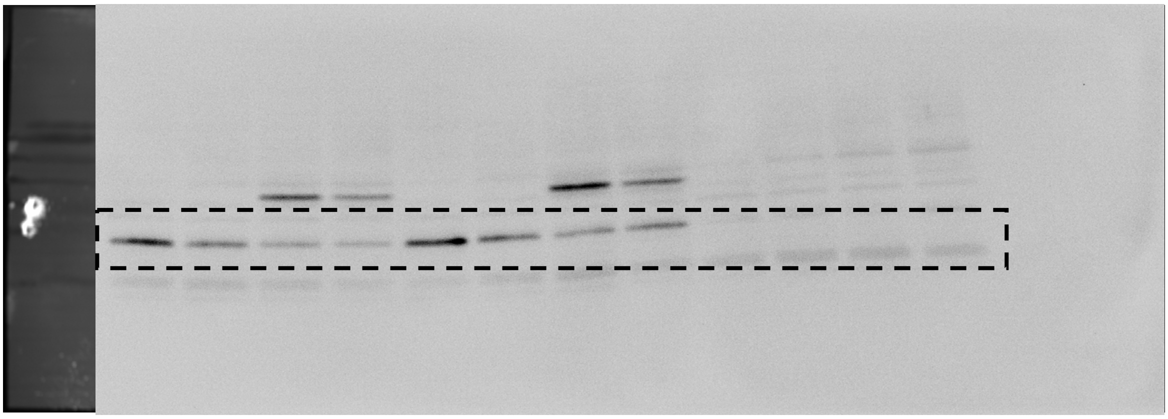

anti-FLAG

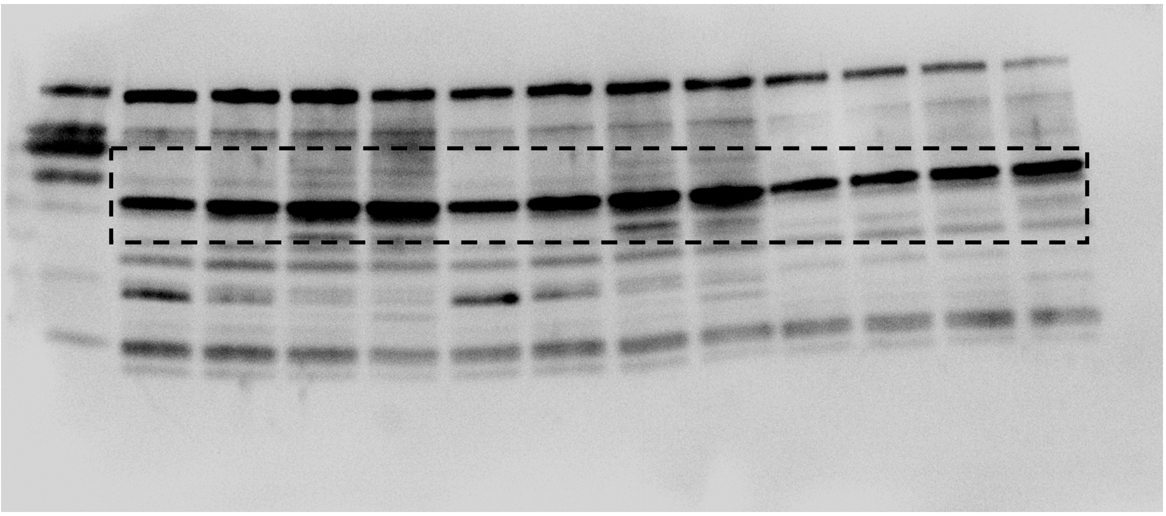

anti-RNAP

Source data for Fig. 6A

Replicate I – Northern Blot

QrrX

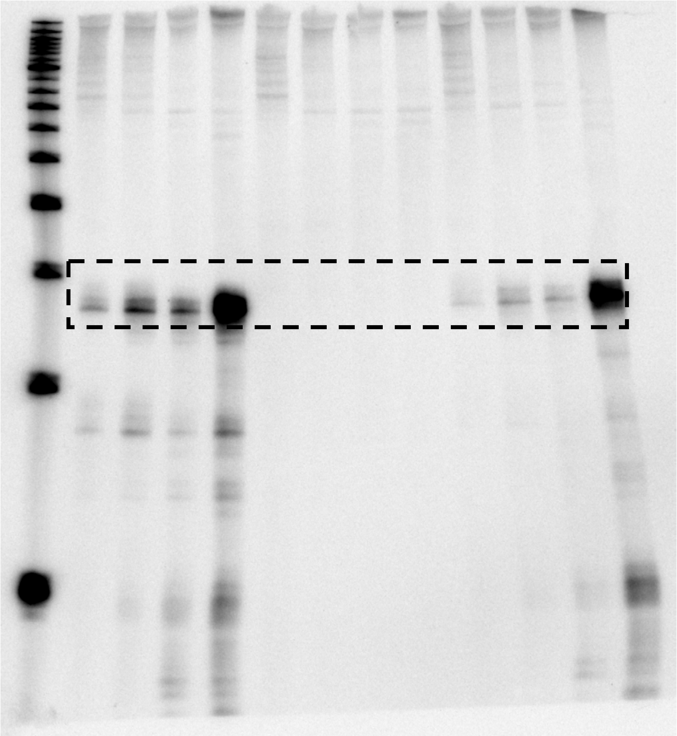

Qrr4

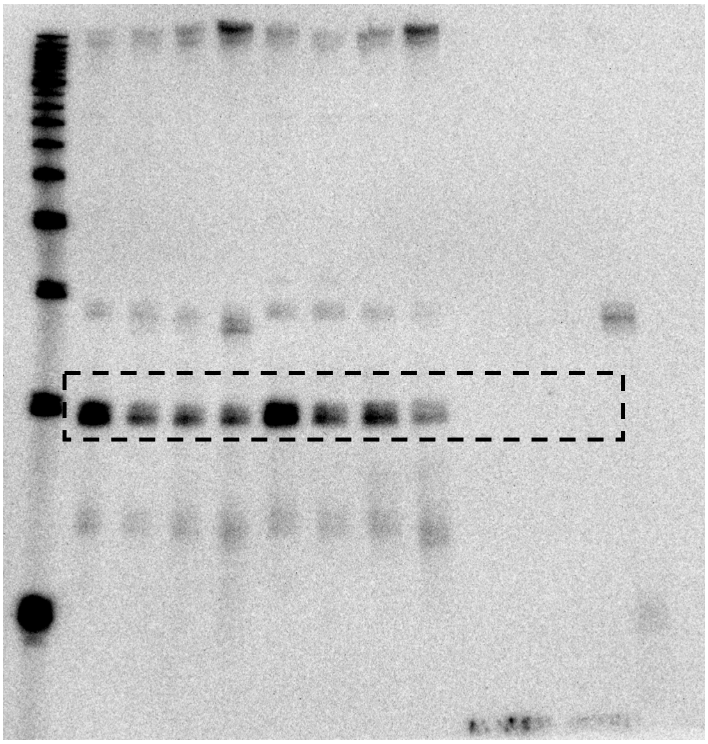

5S

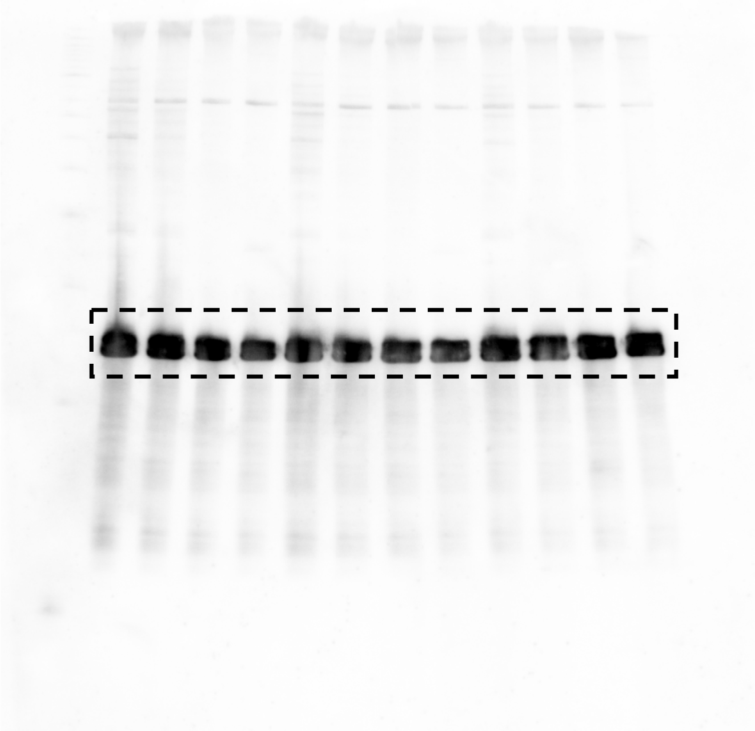

Source data for Fig. 6A

Replicate II – Western Blot

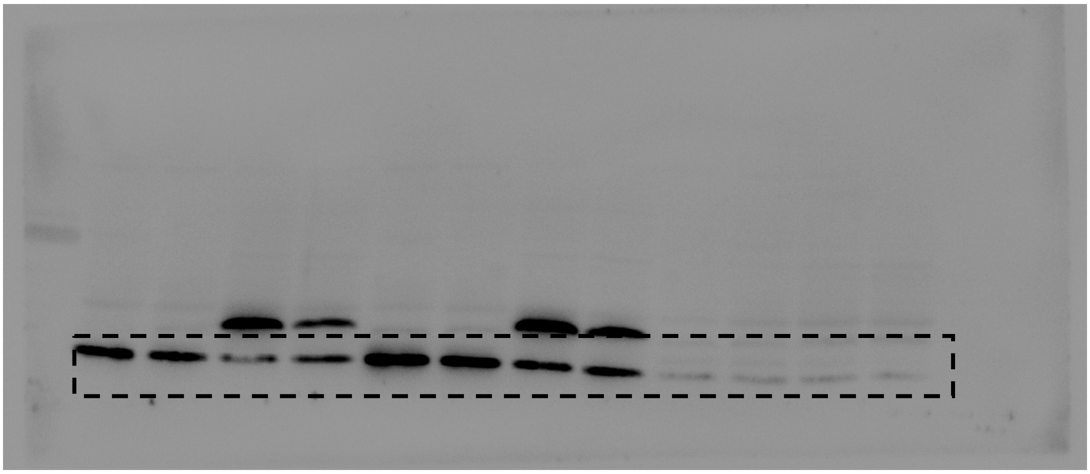

anti-FLAG

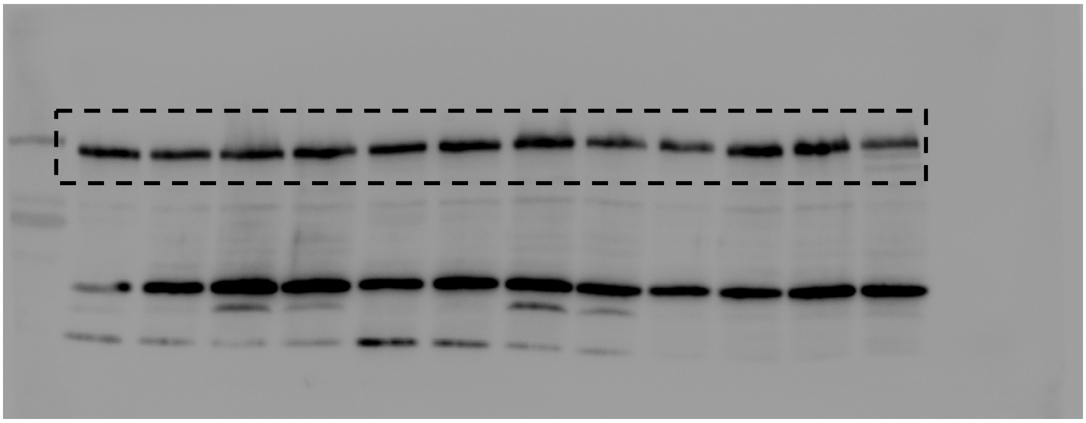

anti-RNAP

Source data for Fig. 6A

Replicate II – Northern Blot

QrrX

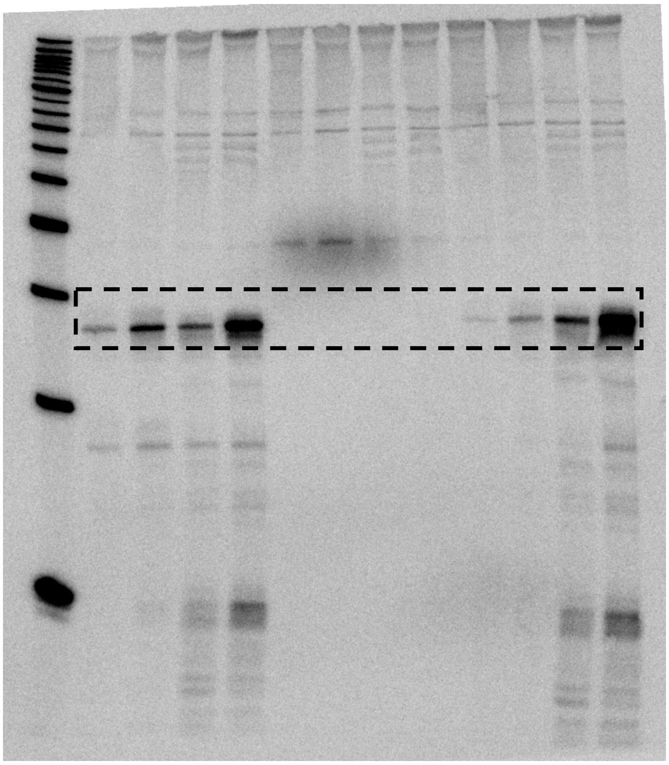

Qrr4

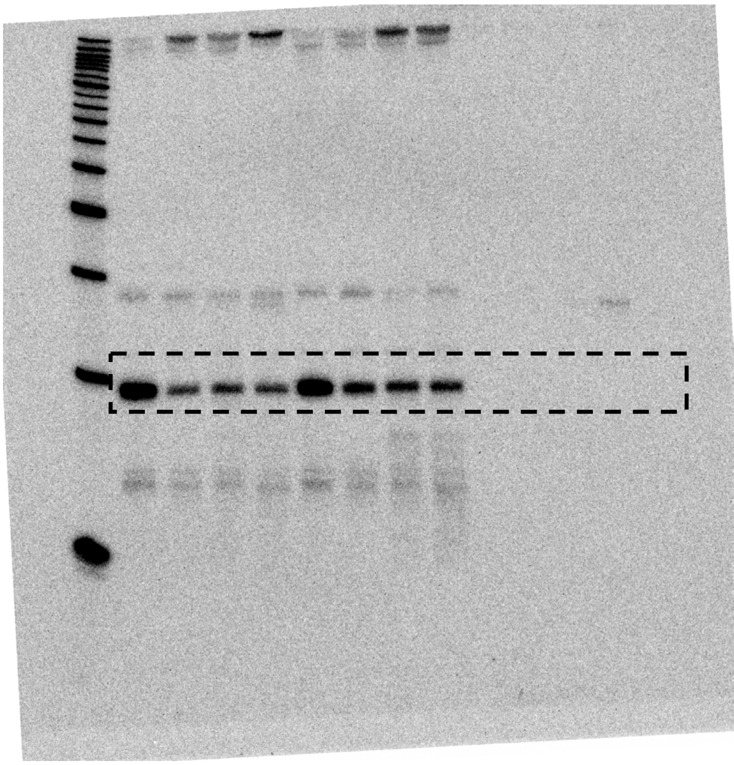

5S

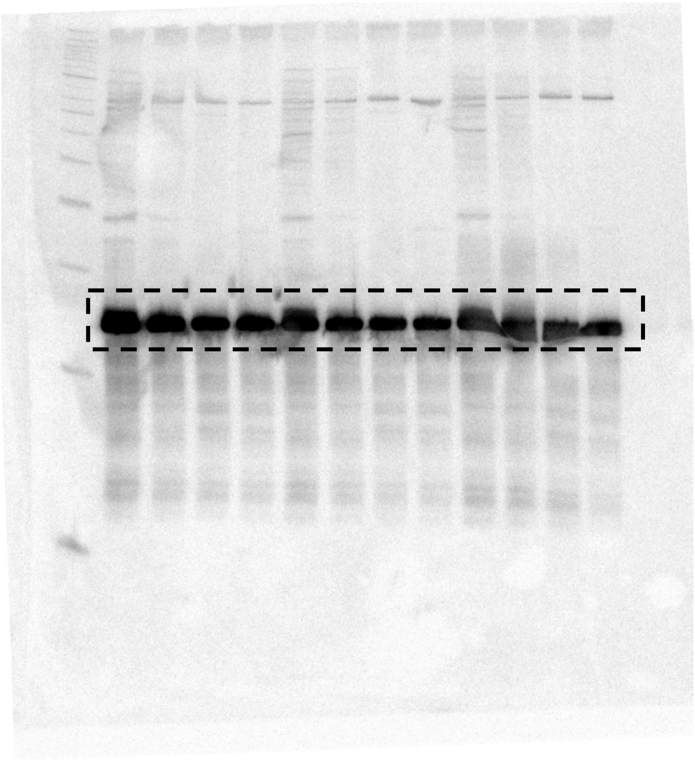

Source data for Fig. 6A

Replicate III – Western Blot

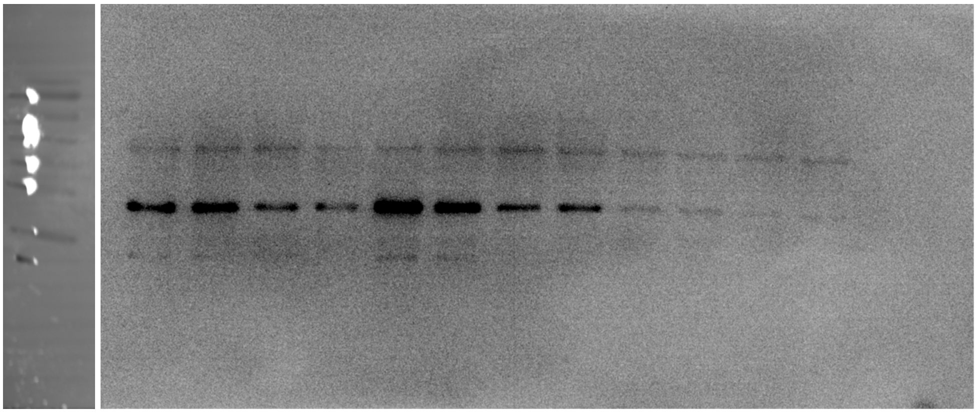

anti-FLAG

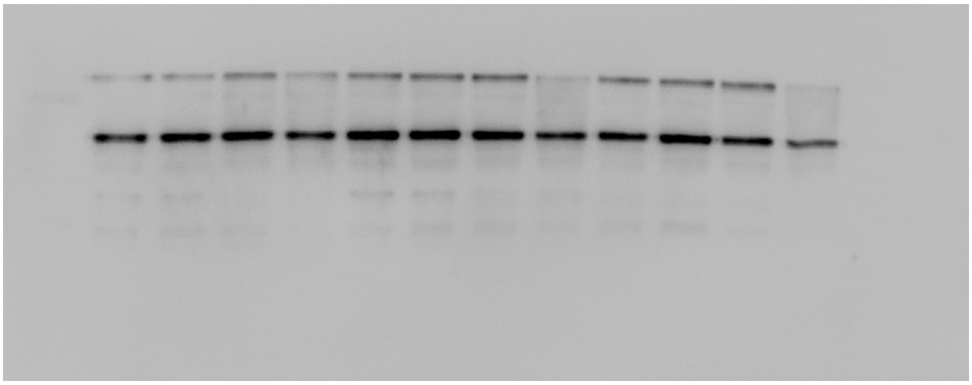

anti-RNAP

Source data for Fig. 6A

Replicate III – Northern Blot

QrrX

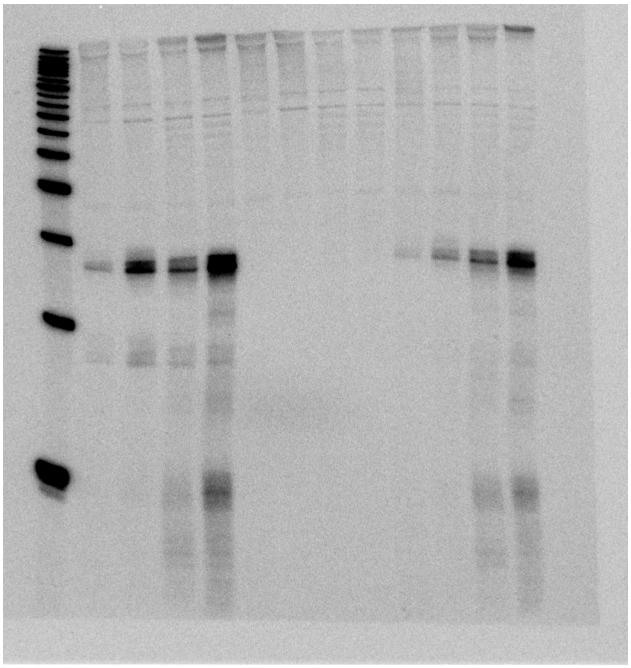

Qrr4

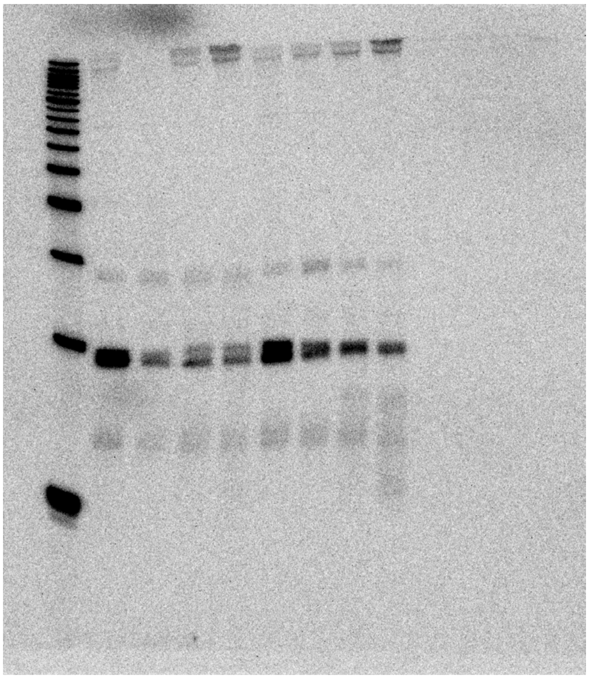

5S

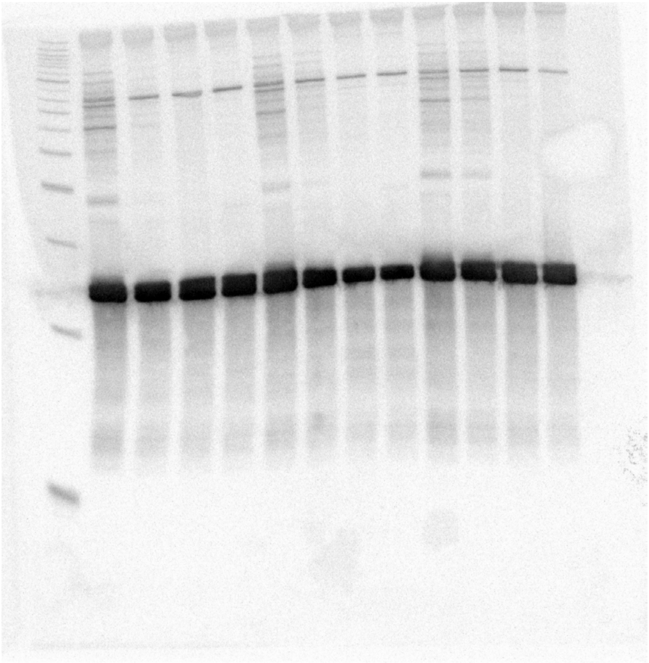

Source data for Fig. 6B

Replicate I – Western Blot

anti-FLAG

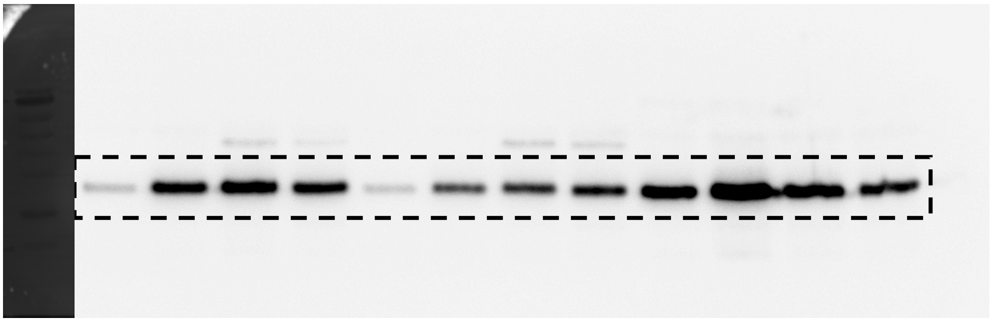

anti-RNAP

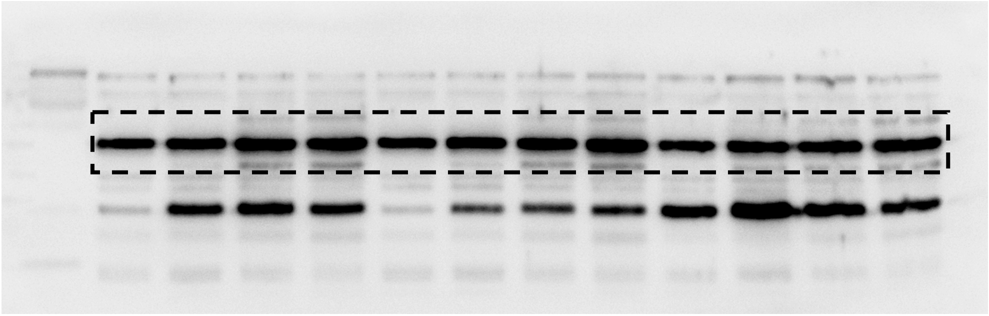

Source data for Fig. 6B

Replicate I – Northern Blot

QrrX

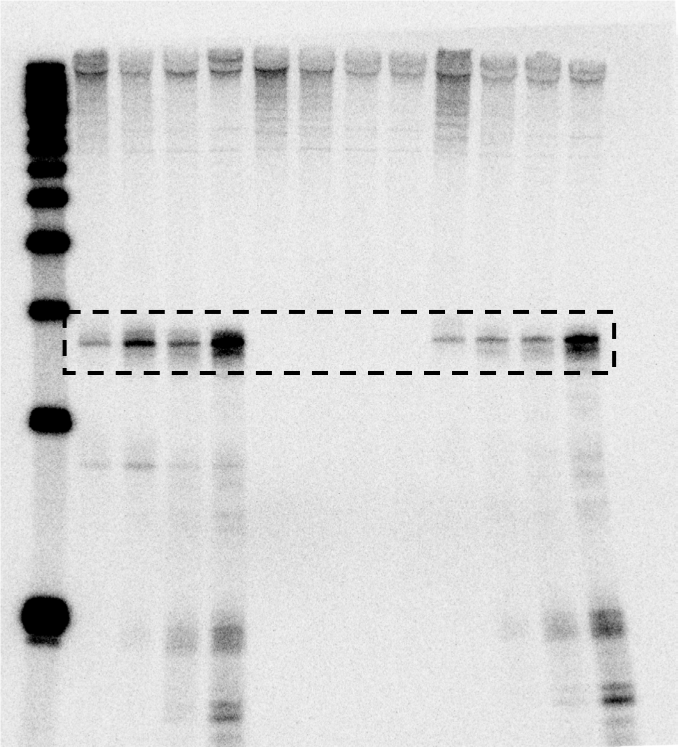

Qrr4

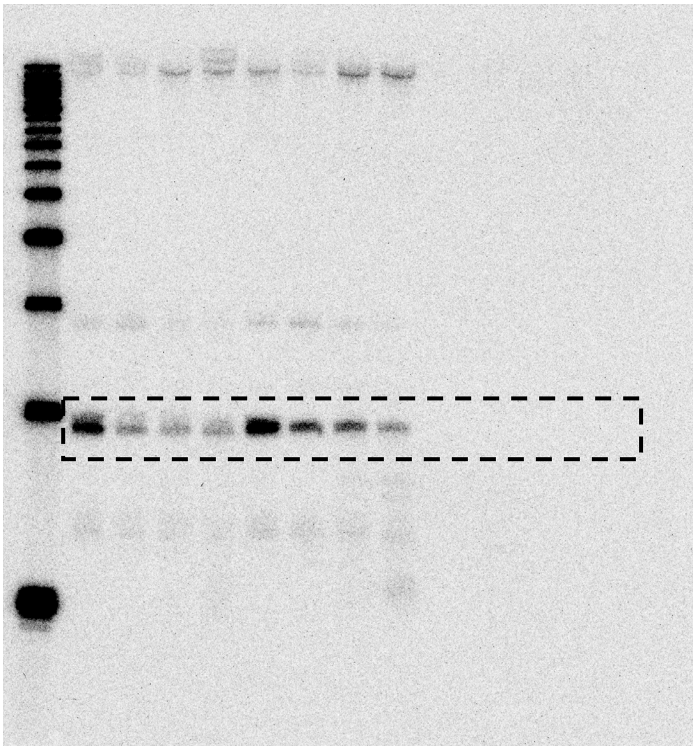

5S

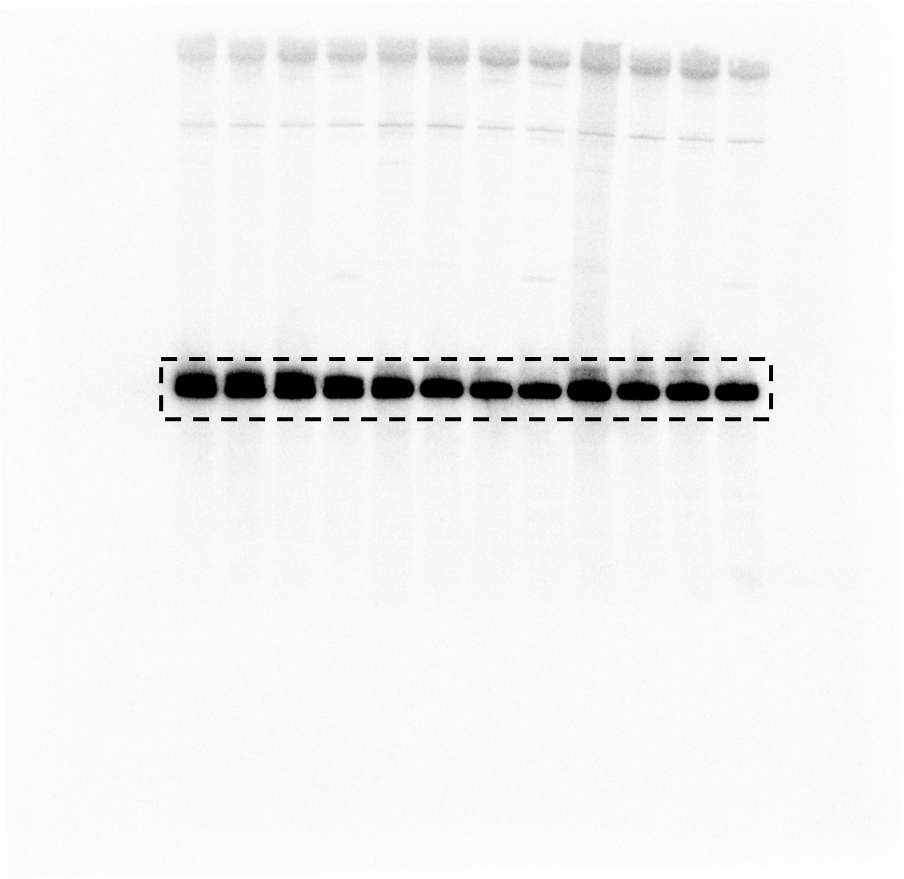

Source data for Fig. 6B

Replicate II – Western Blot

anti-FLAG

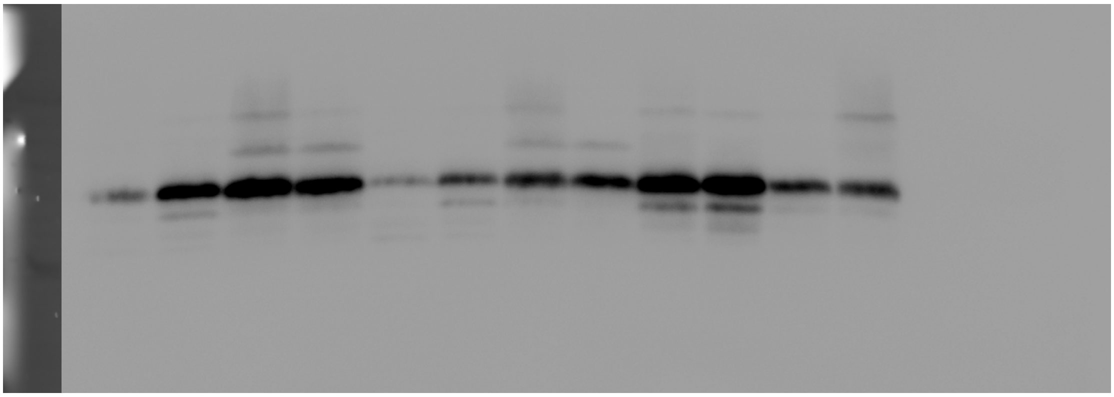

anti-RNAP

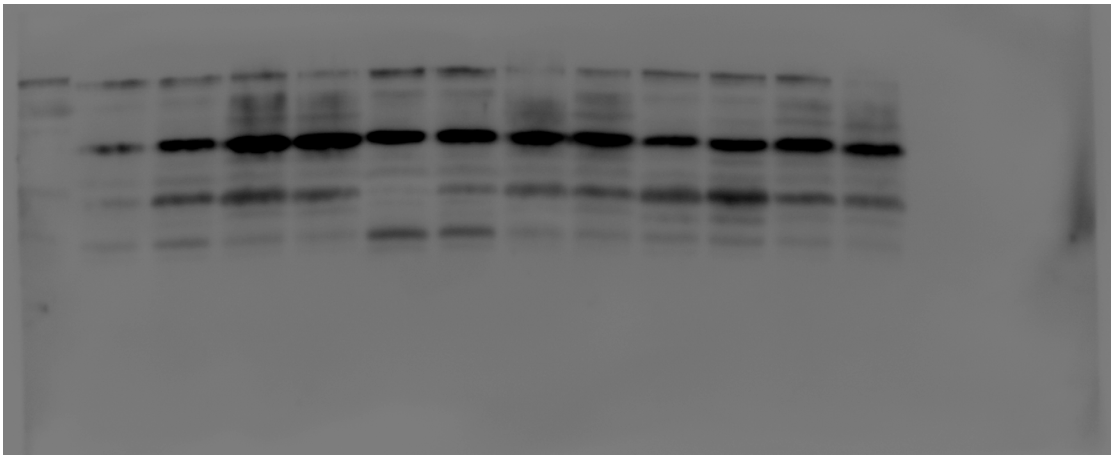

Source data for Fig. 6B

Replicate II – Northern Blot

QrrX

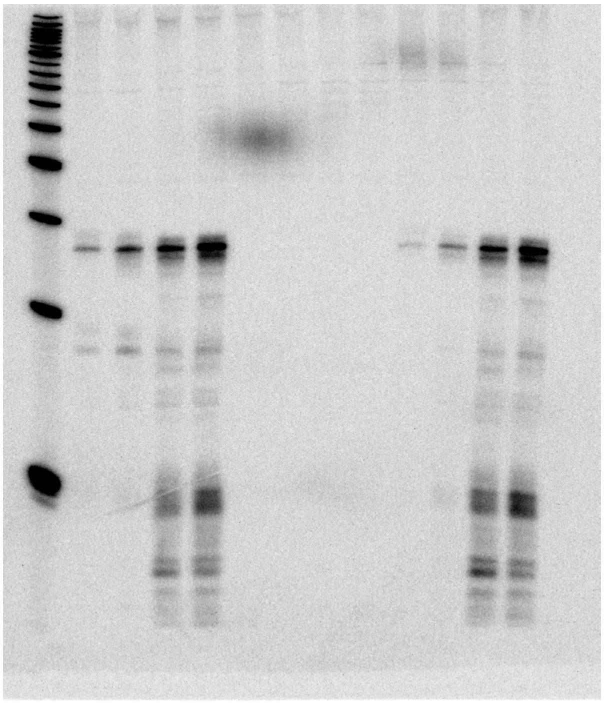

Qrr4

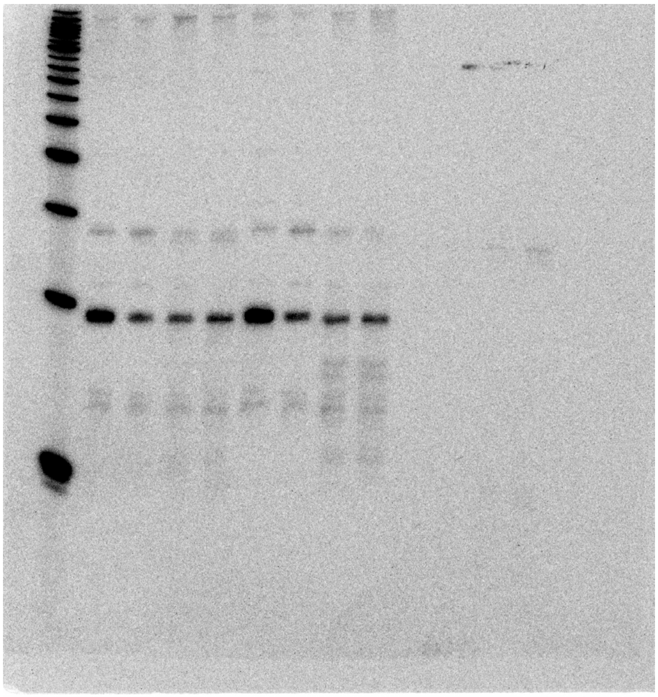

5S

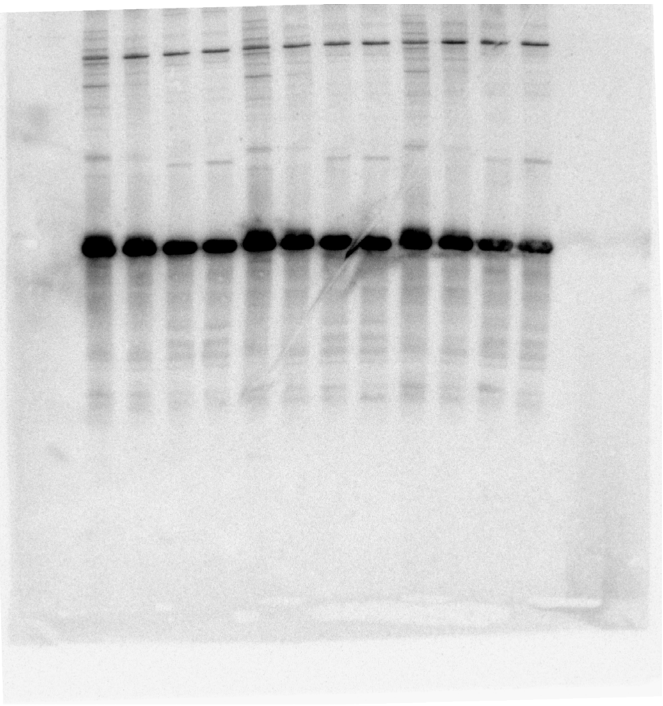

Source data for Fig. 6B

Replicate III – Western Blot

anti-FLAG

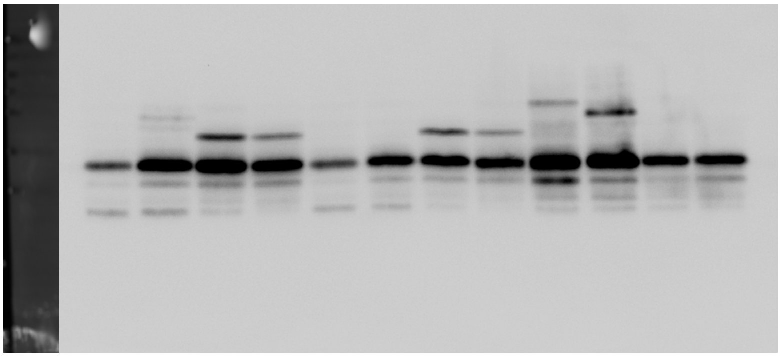

anti-RNAP

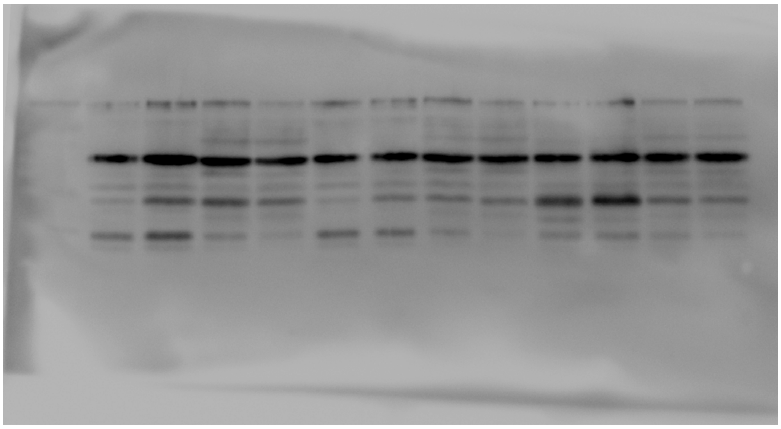

Source data for Fig. 6B

Replicate III – Northern Blot

QrrX

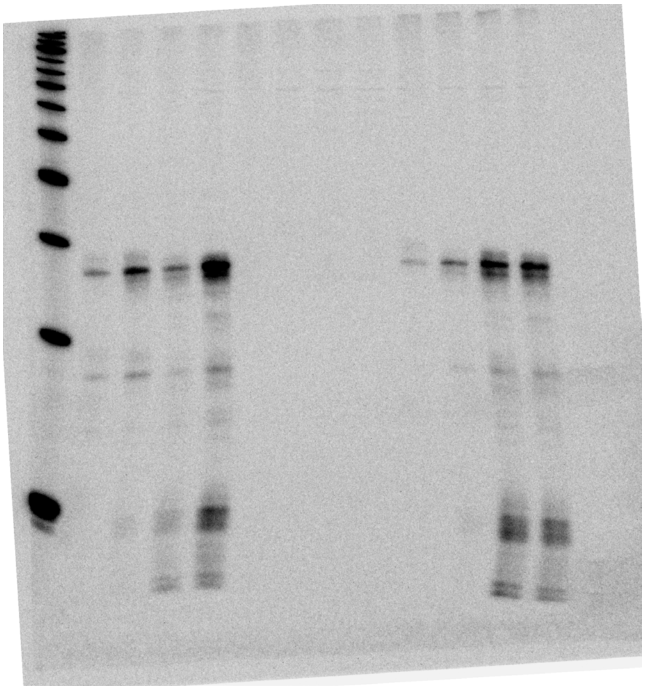

Qrr4

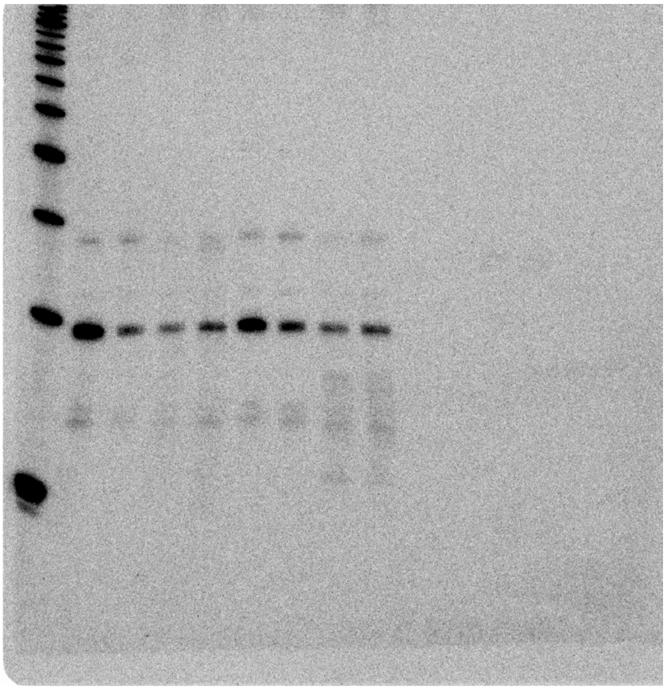

5S

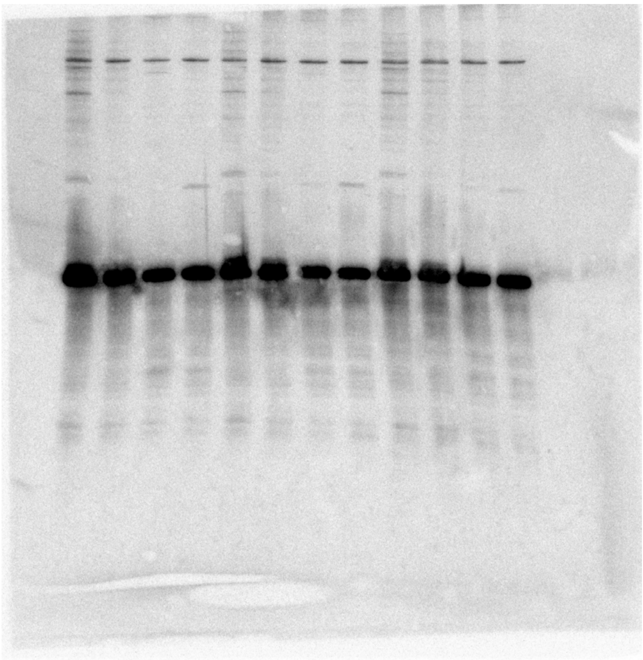

Source data for Fig. 6C

AphA

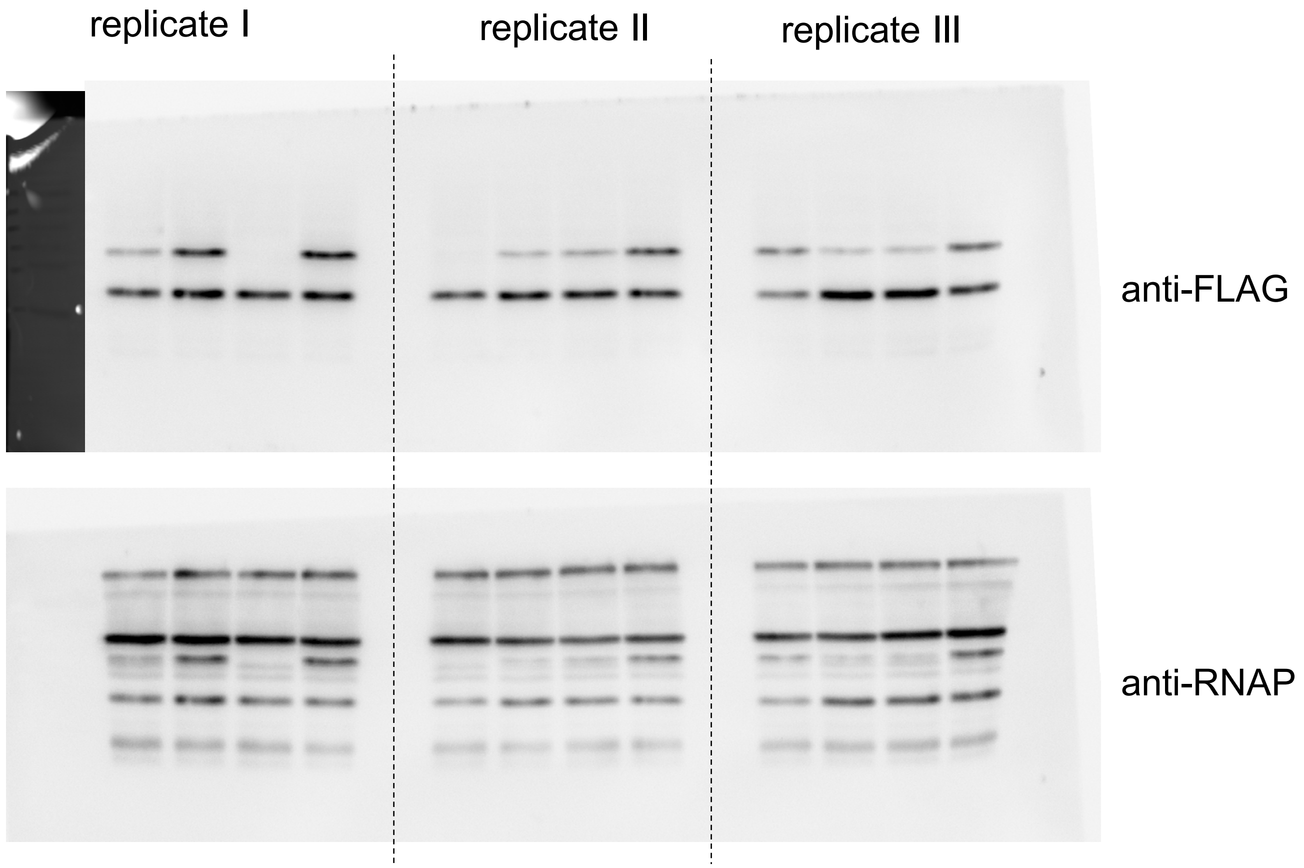

Source data for Fig. 6C

HapR

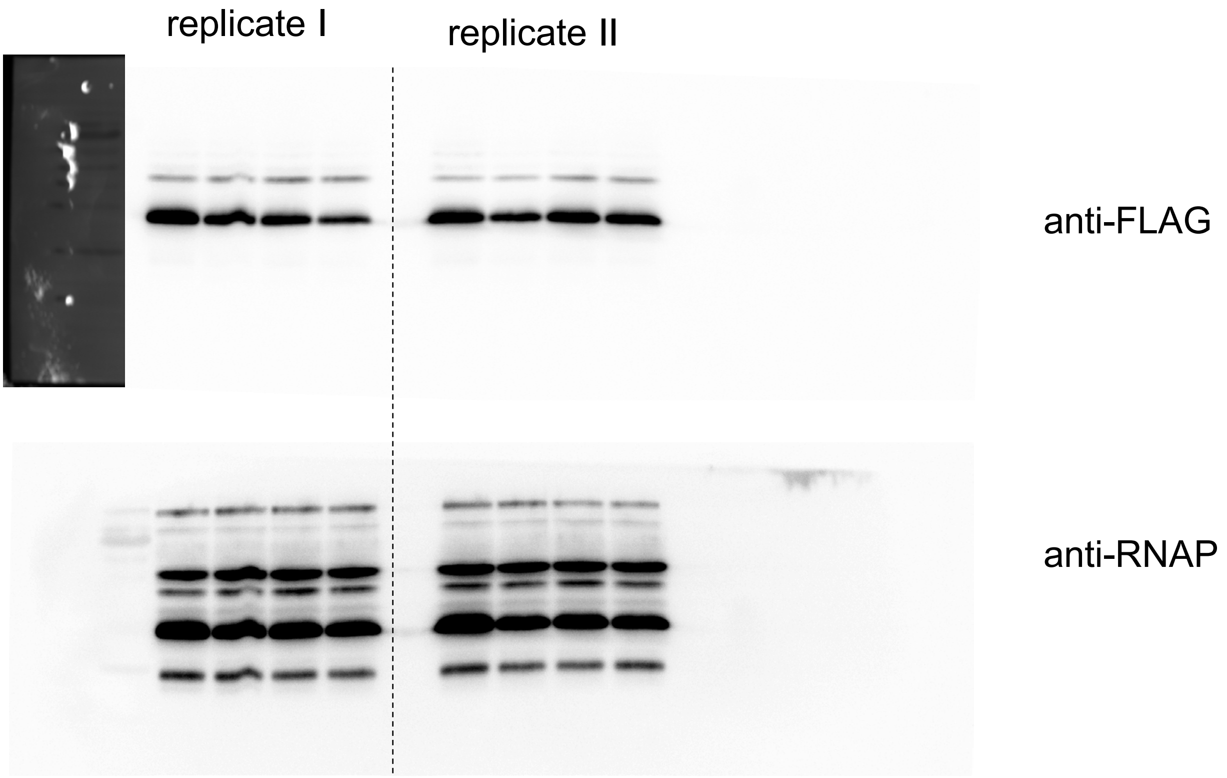

replicate III

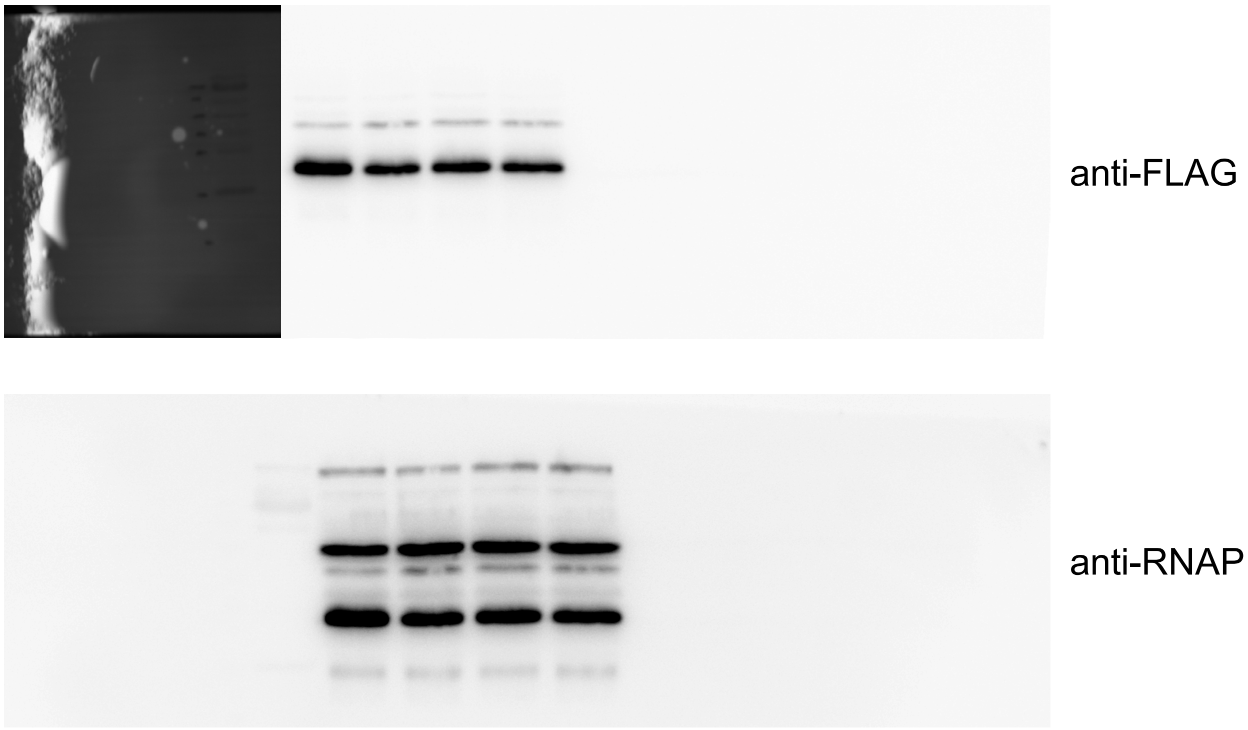

Source data for Fig. S4A
Replicate I

QrrX

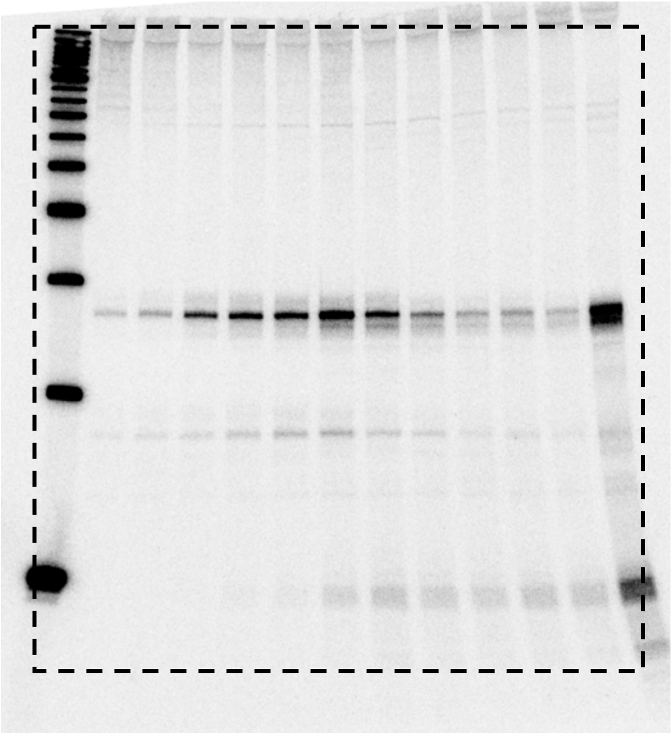

Qrr1

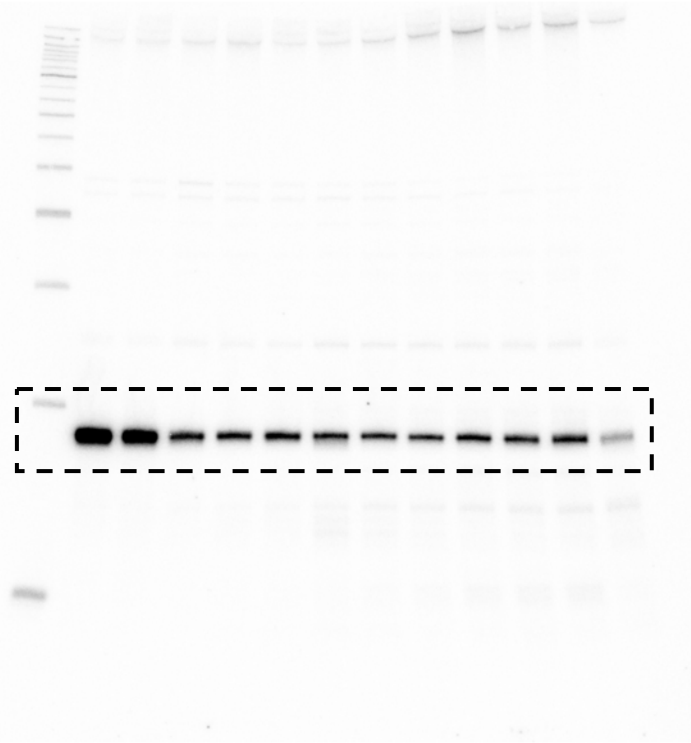

Qrr2

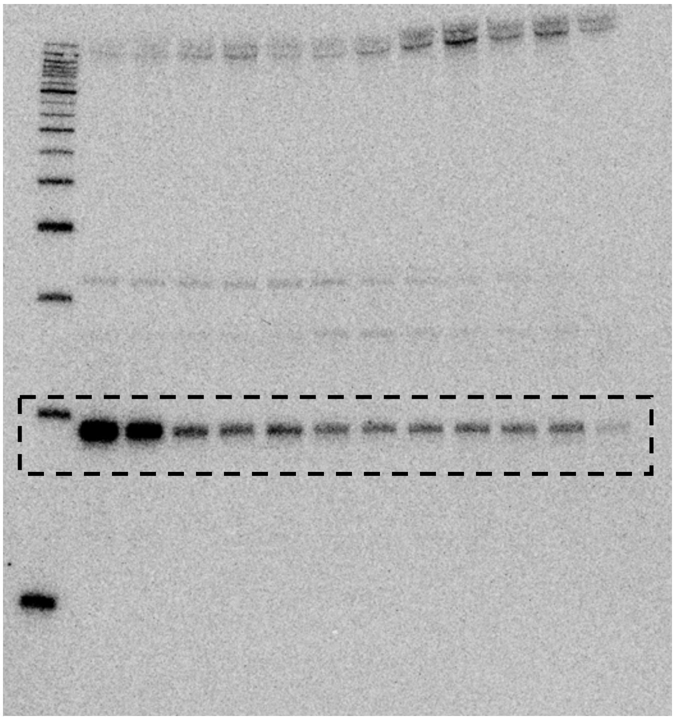

Qrr3

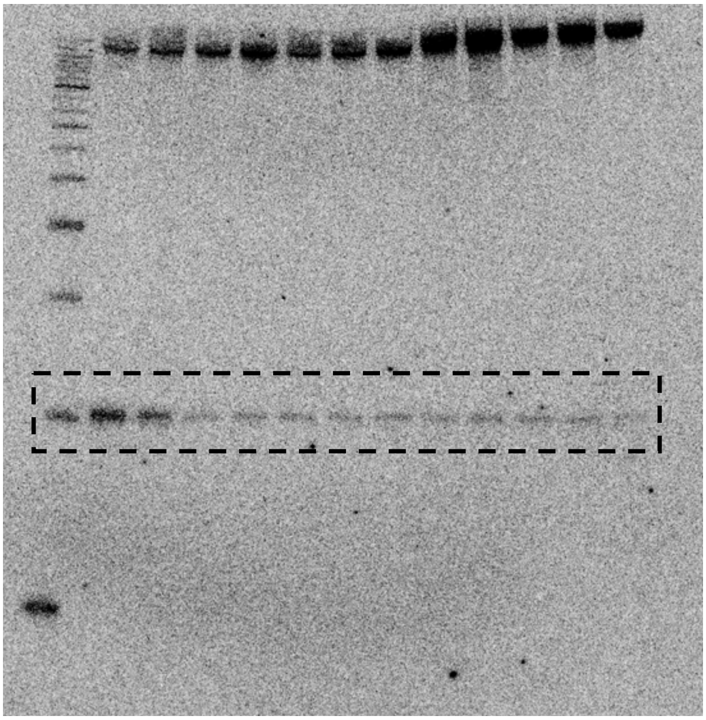

Source data for Fig. S4A

Replicate I

Qrr4

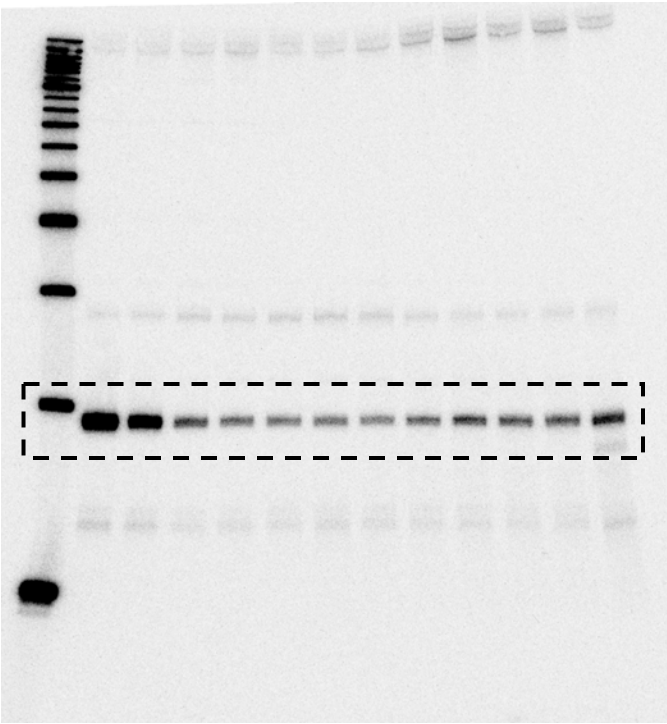

5S rRNA

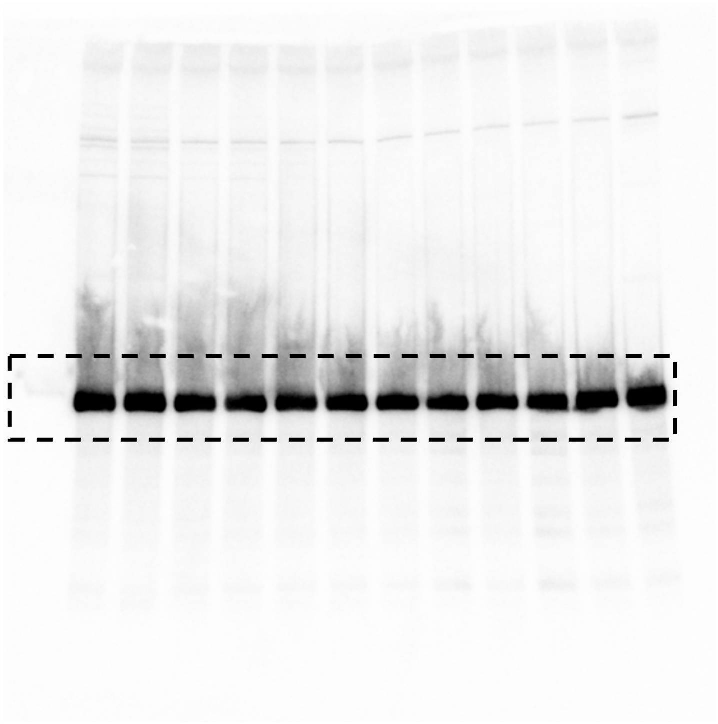

Source data for Fig. S4A

Replicate II

QrrX

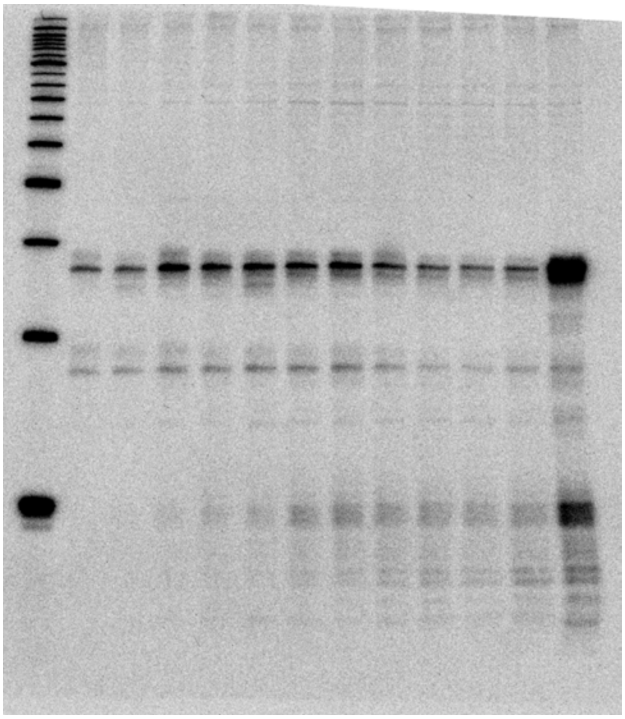

Qrr1

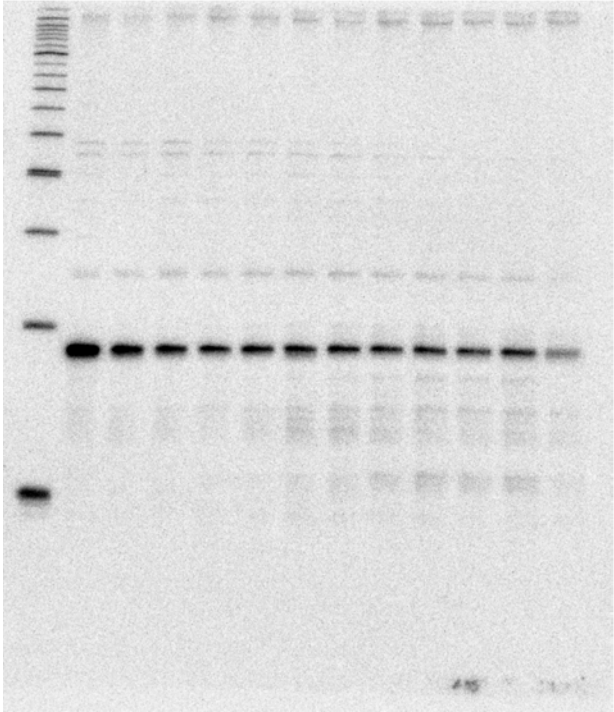

Qrr2

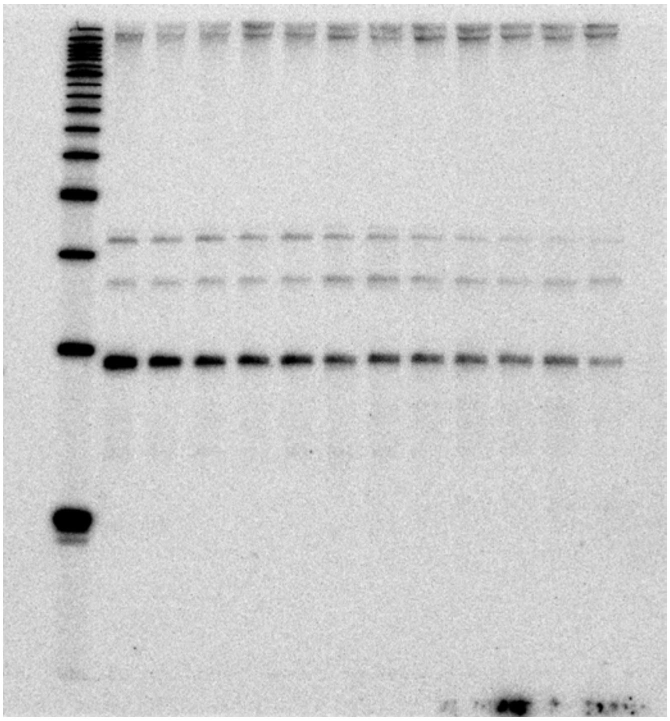

Qrr3

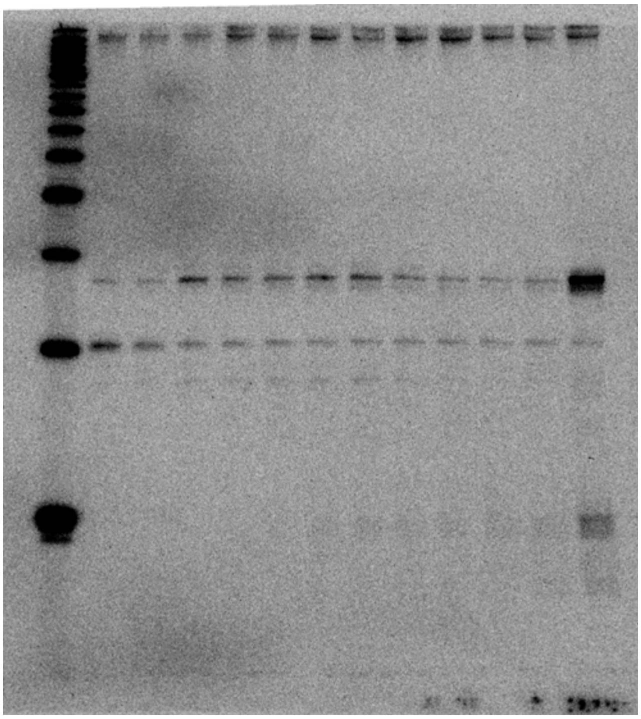

Source data for Fig. S4A
Replicate II

Qrr4

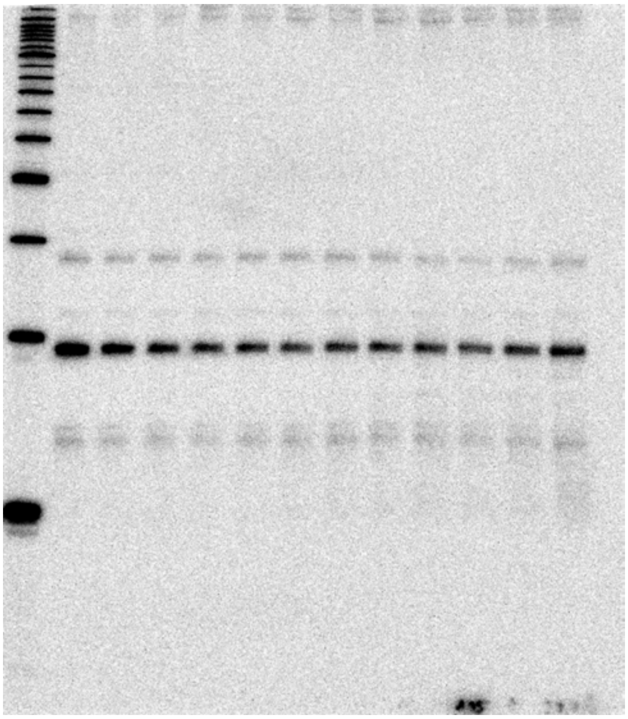

5S rRNA

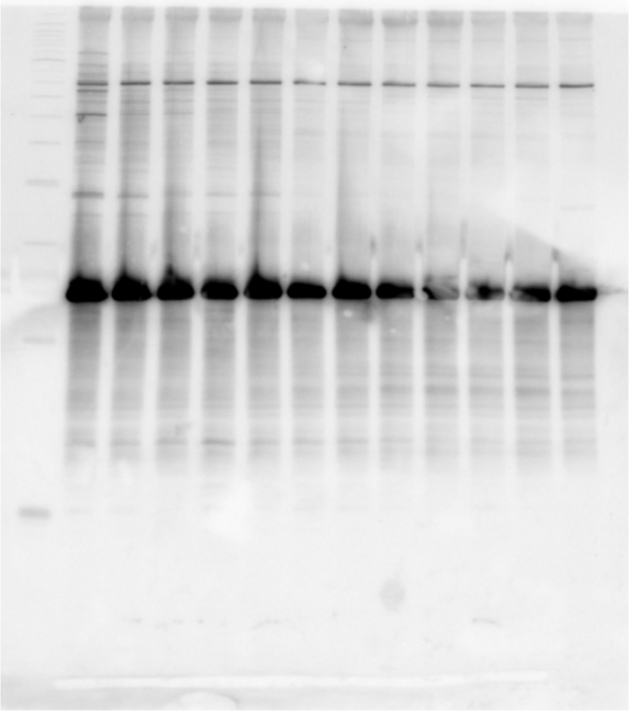

Source data for Fig. S4A

Replicate III

QrrX

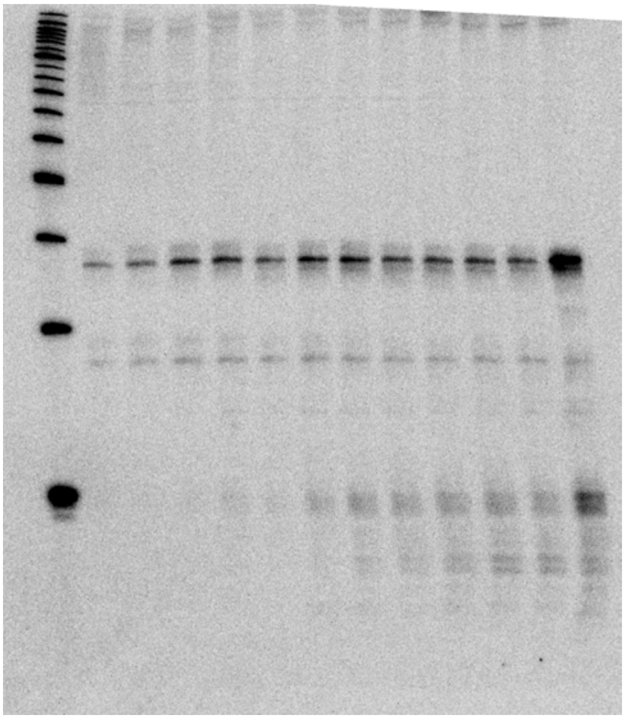

Qrr1

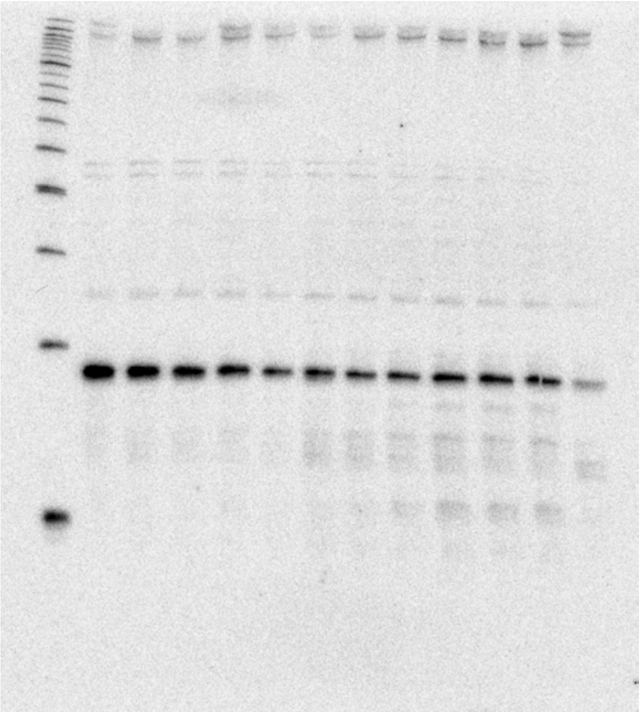

Qrr2

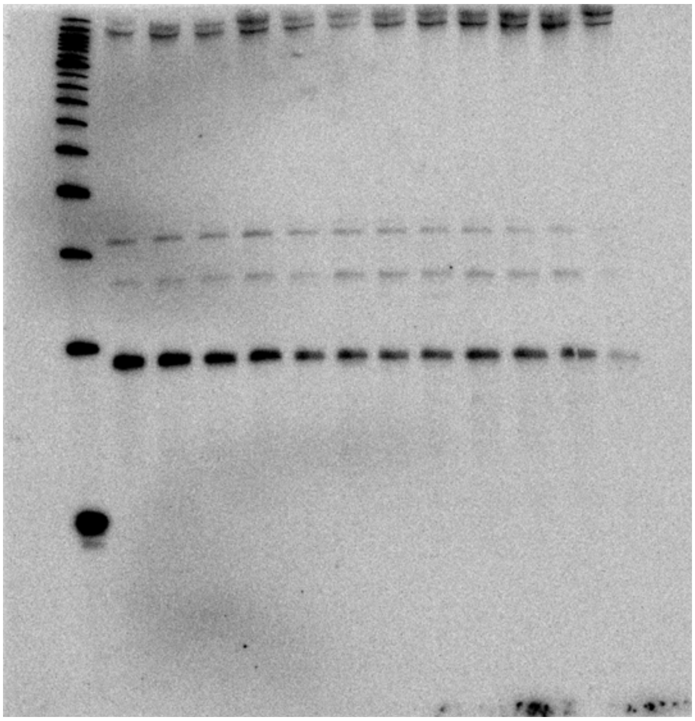

Qrr3

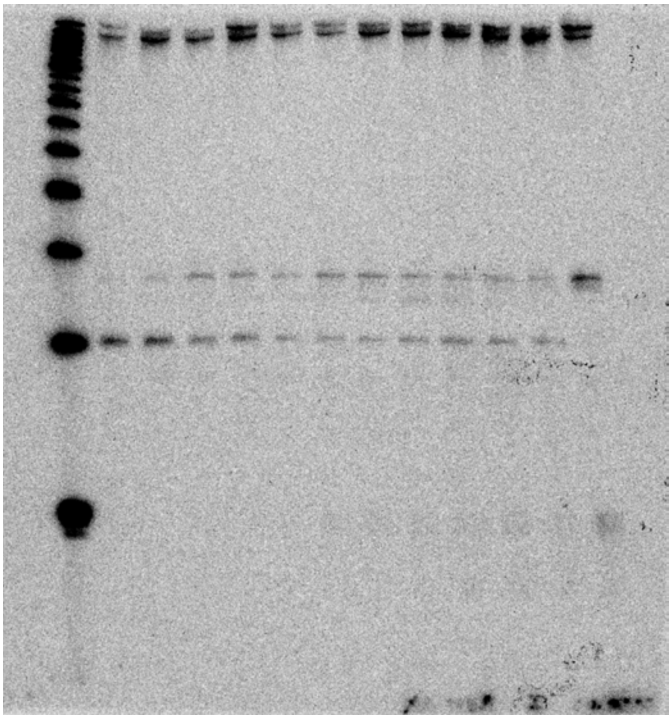

Source data for Fig. S4A

Replicate III

Qrr4

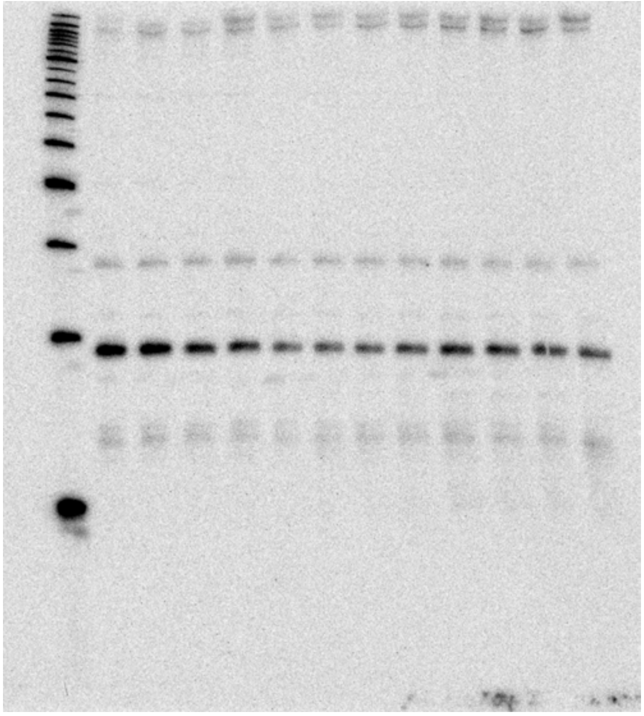

5S rRNA

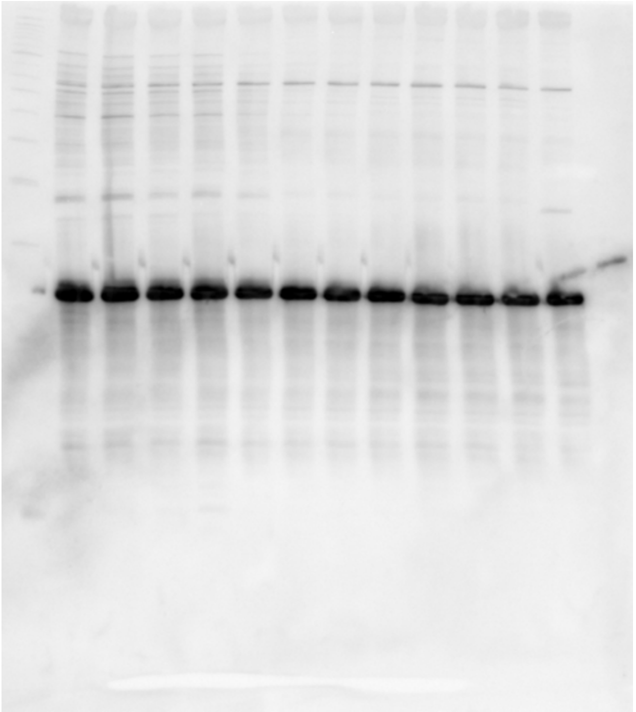

Source data for Fig. S4B

Replicate I

QrrX

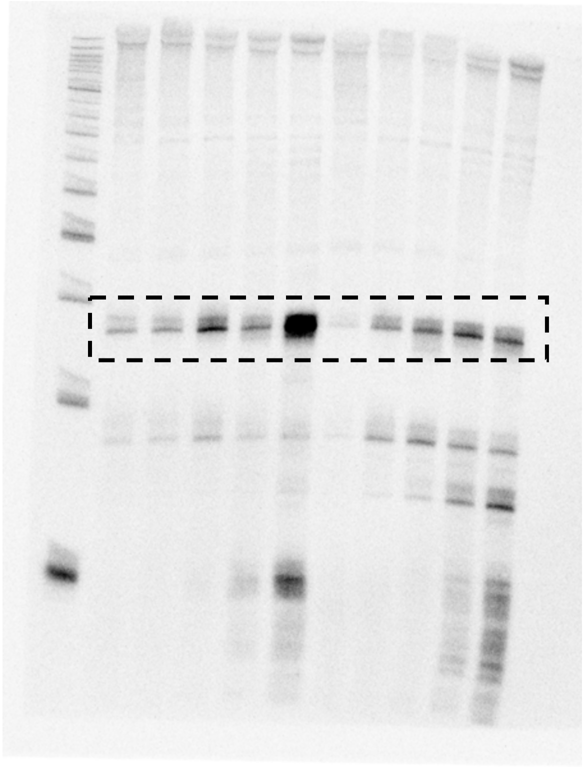

Qrr1

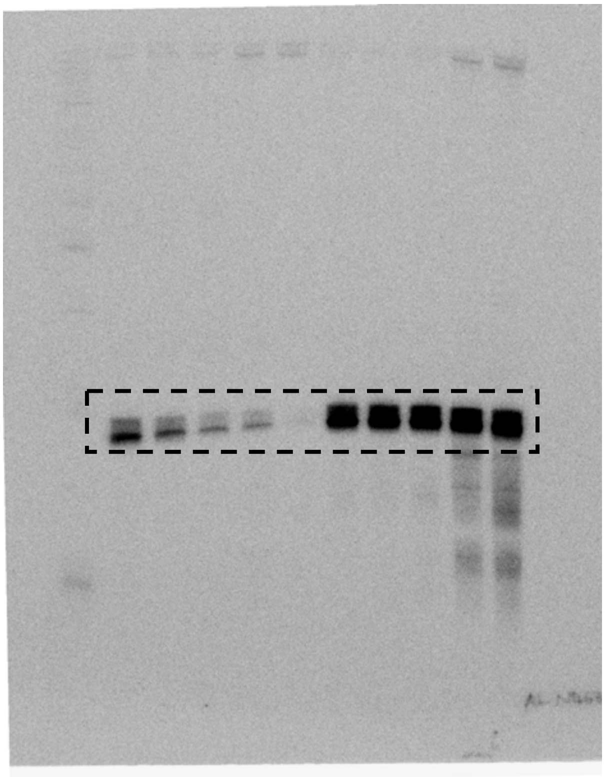

Qrr2

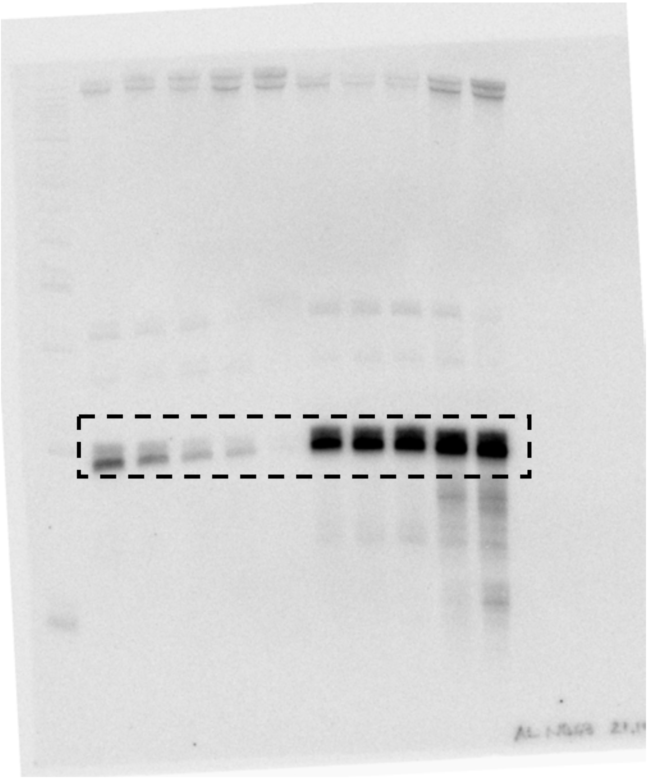

Qrr3

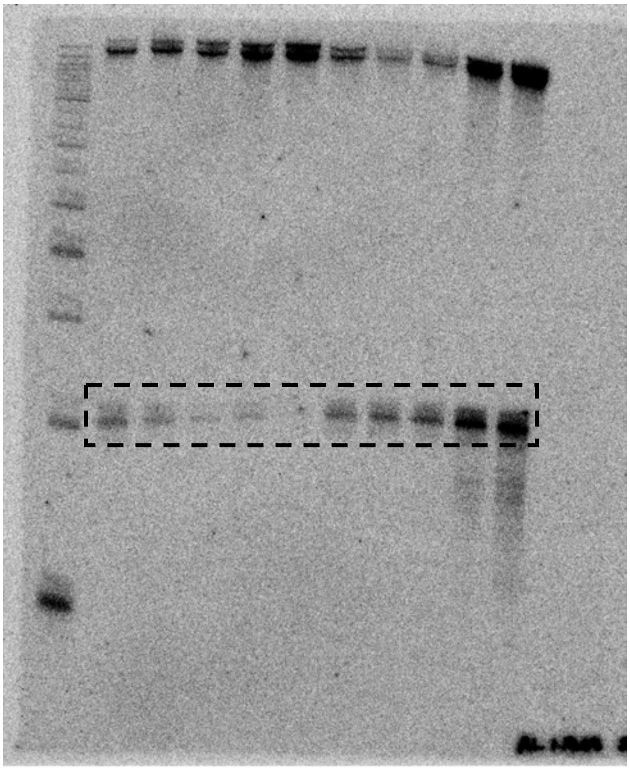

Source data for Fig. S4B

Replicate I

Qrr4

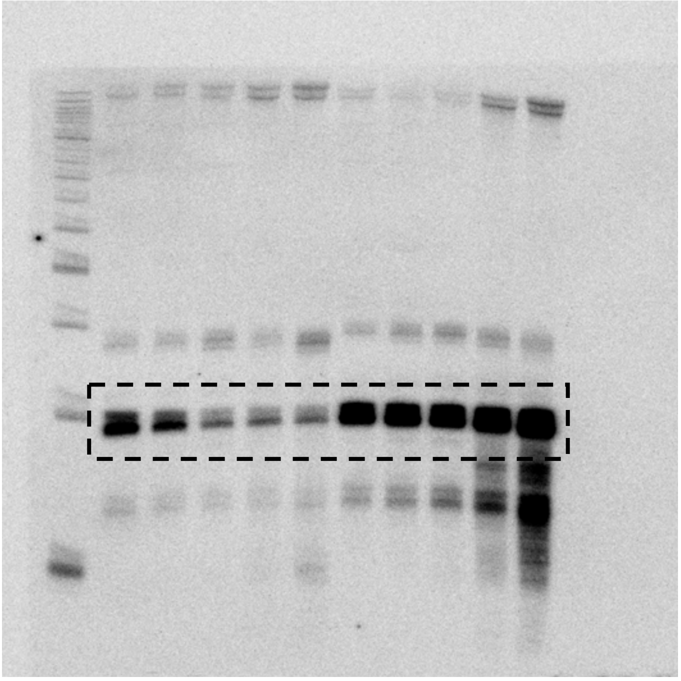

5S rRNA

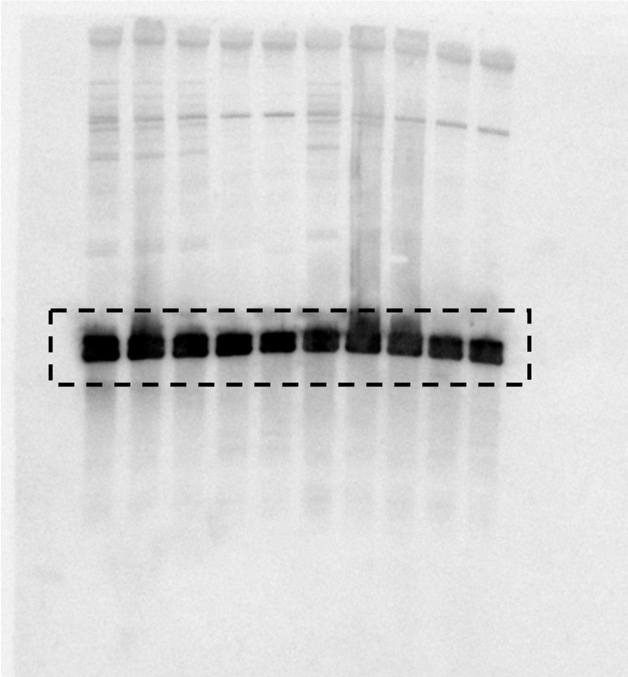

Source data for Fig. S4B

Replicate II

QrrX

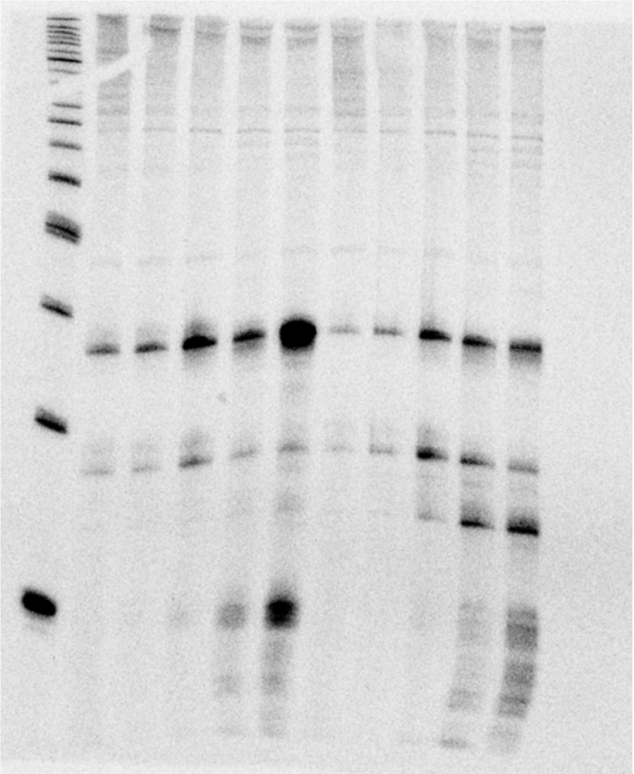

Qrr1

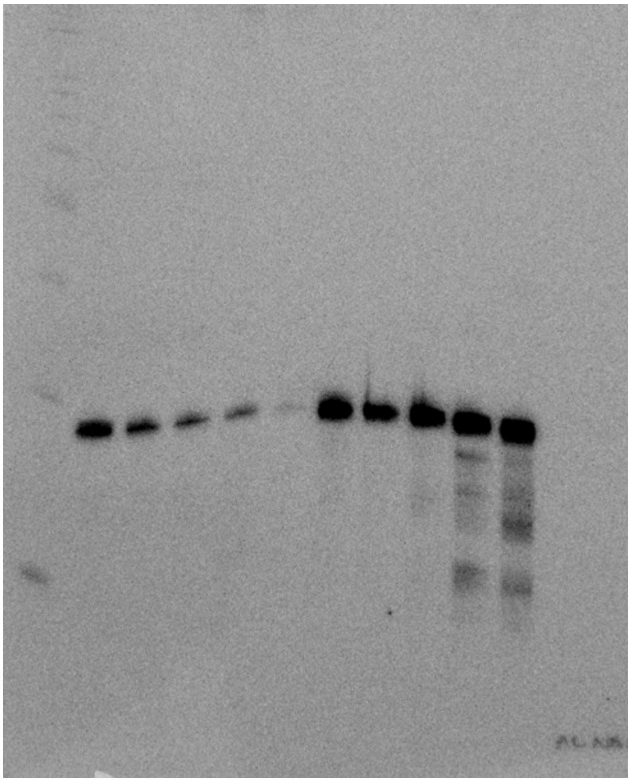

Qrr2

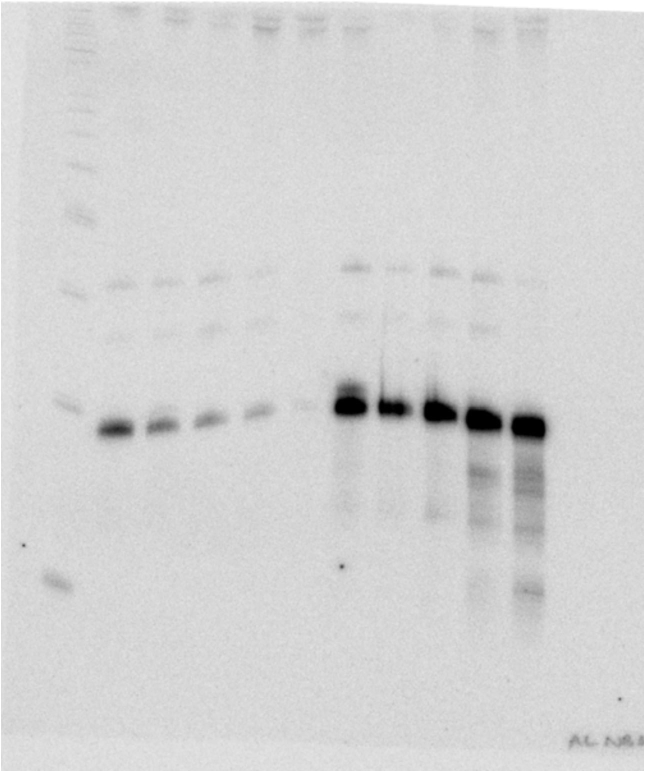

Qrr3

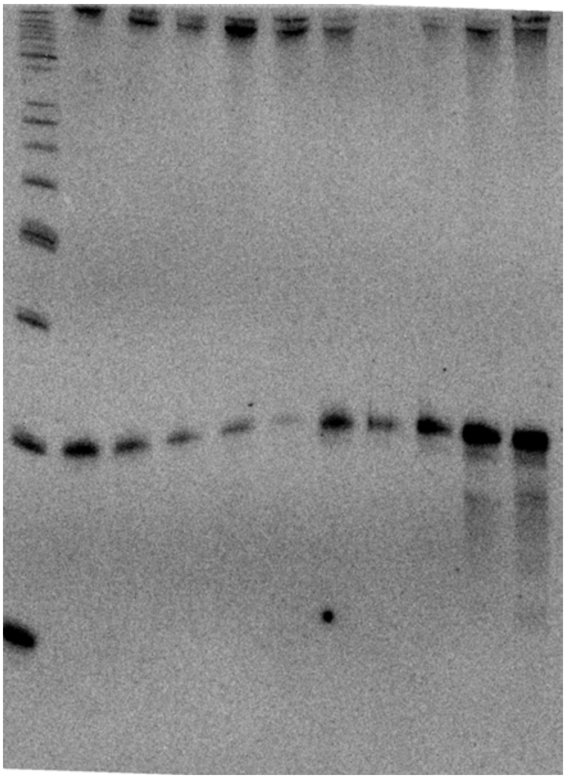

Source data for Fig. S4B

Replicate II

Qrr4

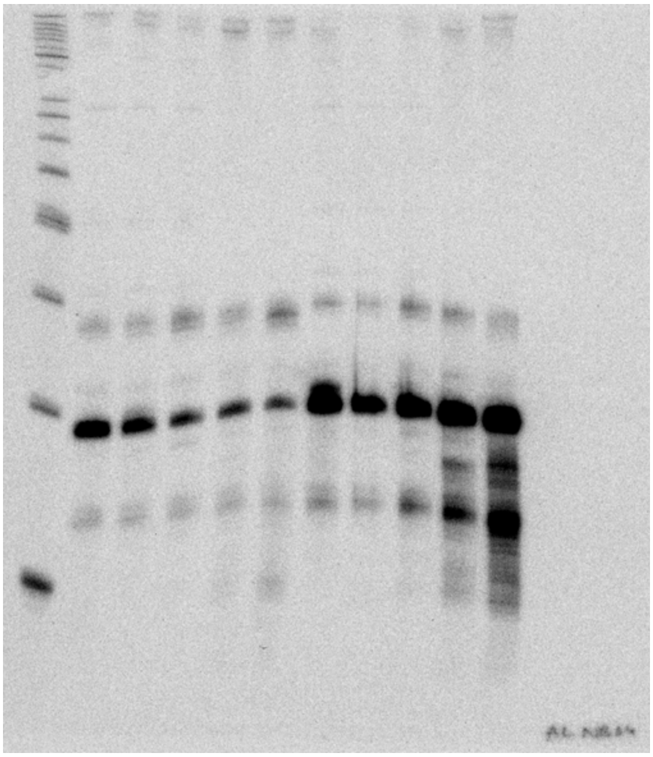

5S rRNA

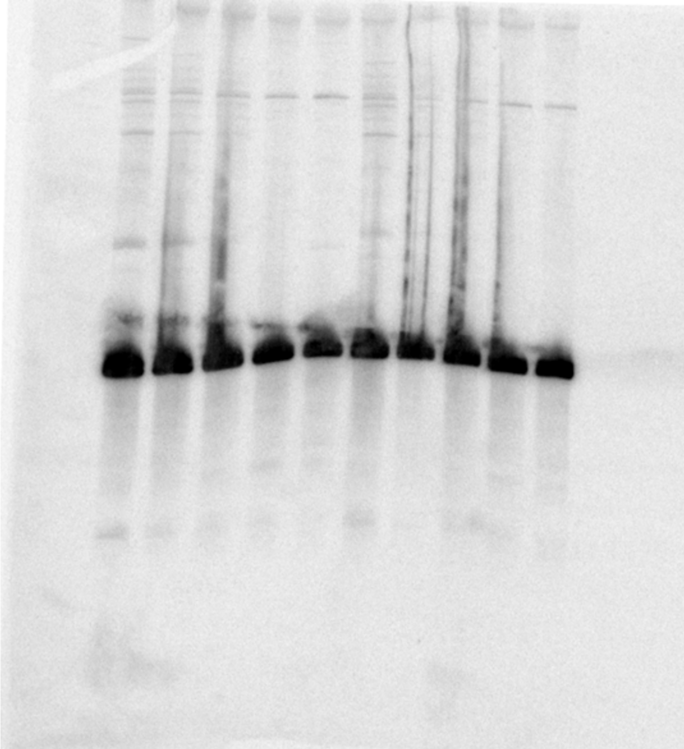

Source data for Fig. S4C

Replicate I

QrrX

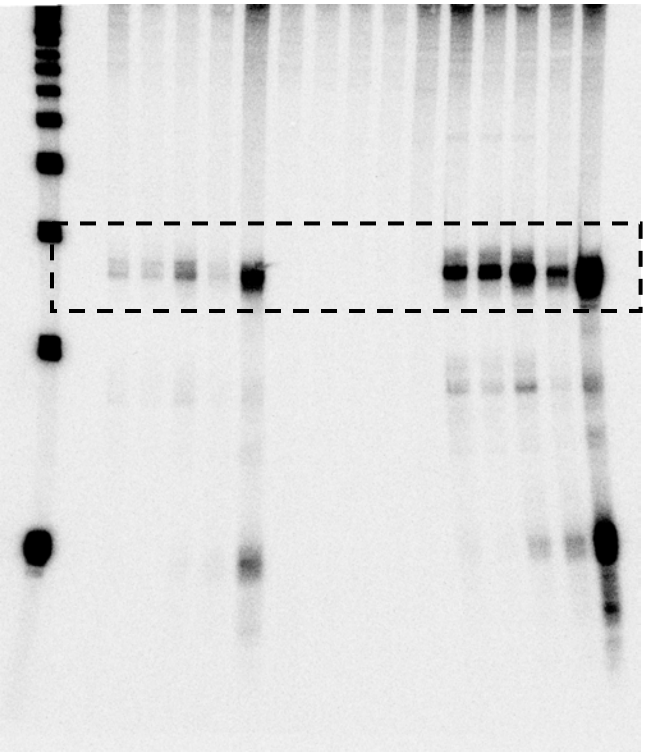

Qrr1

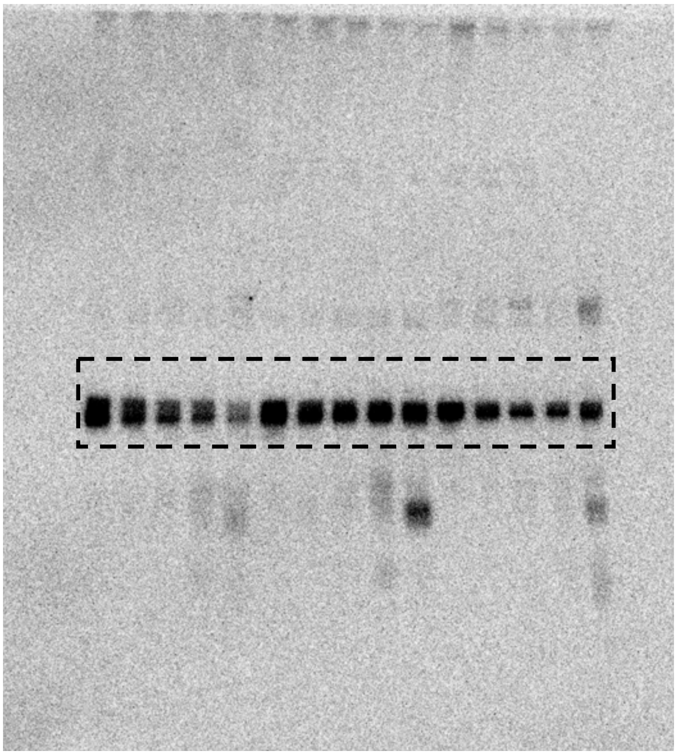

Qrr2

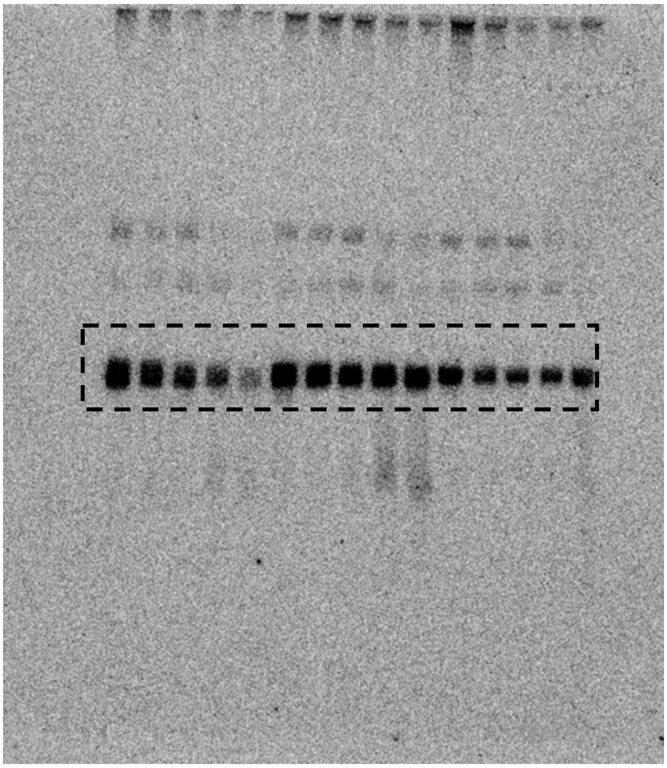

Qrr3

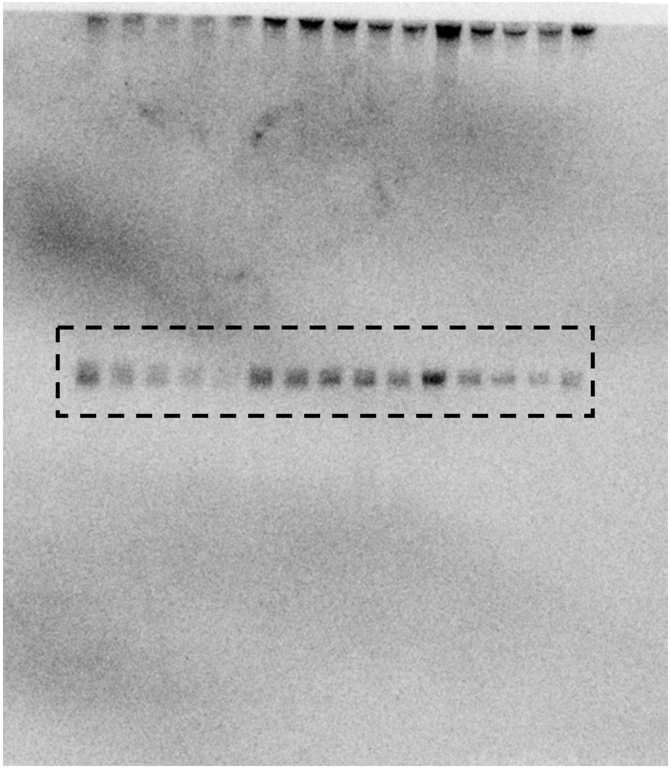

Source data for Fig. S4C

Replicate I

Qrr4

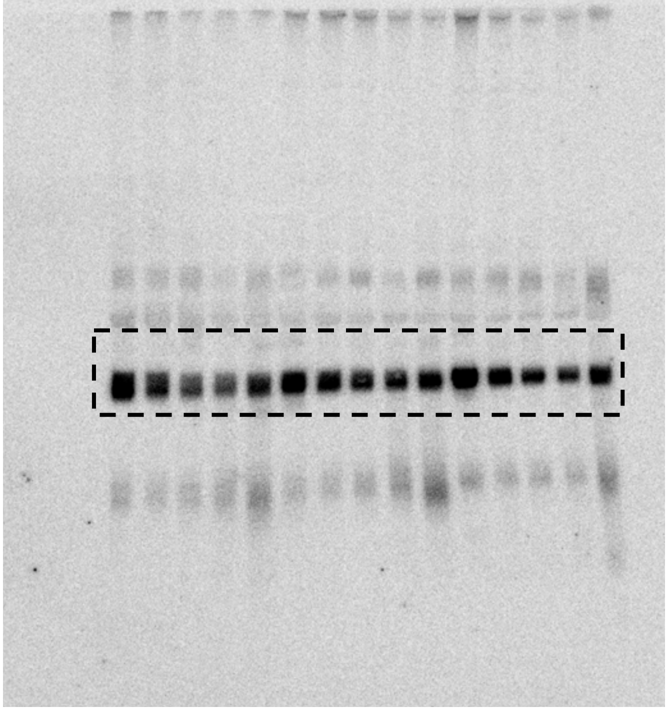

5S rRNA

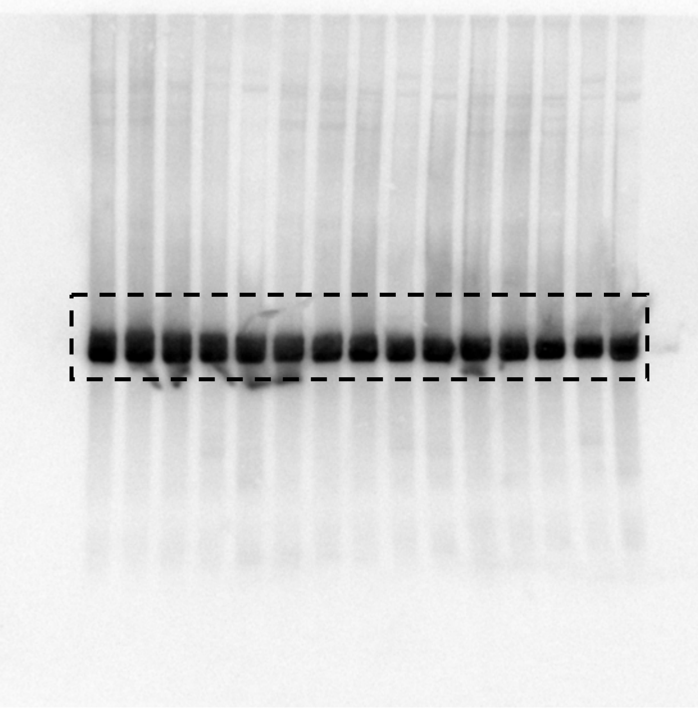

Source data for Fig. S4C

Replicate II

QrrX

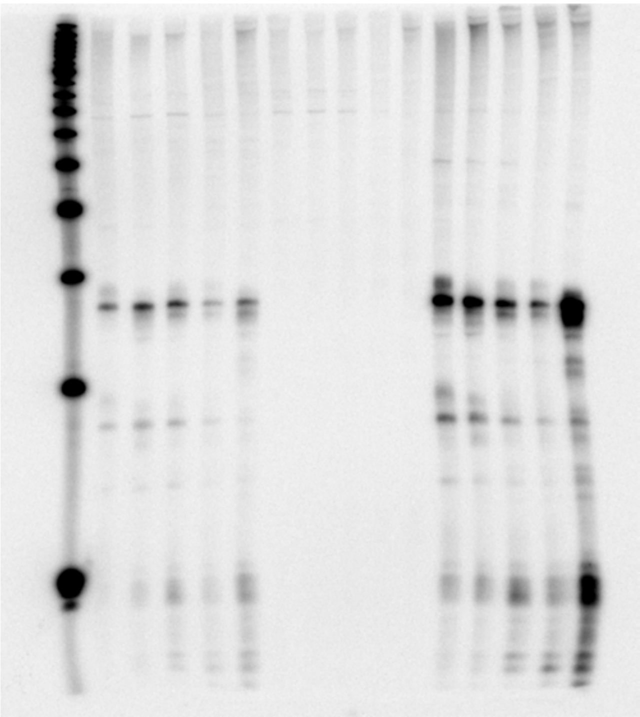

Qrr1

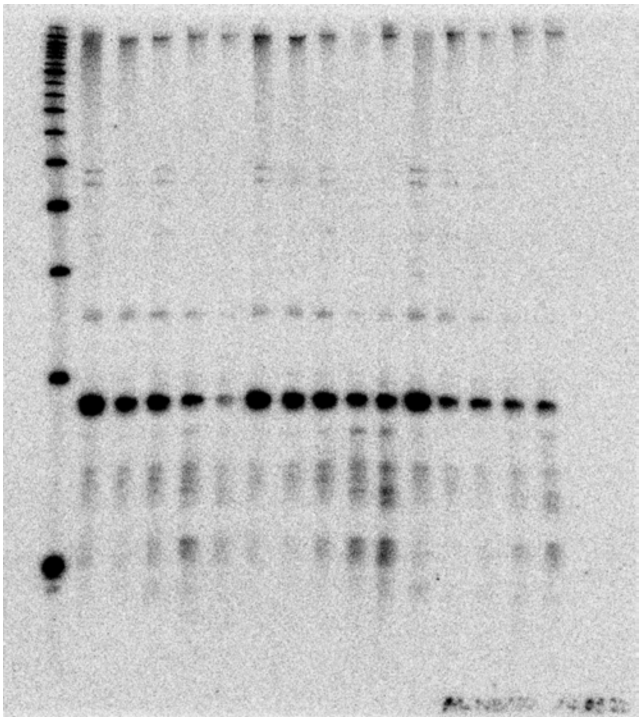

Qrr2

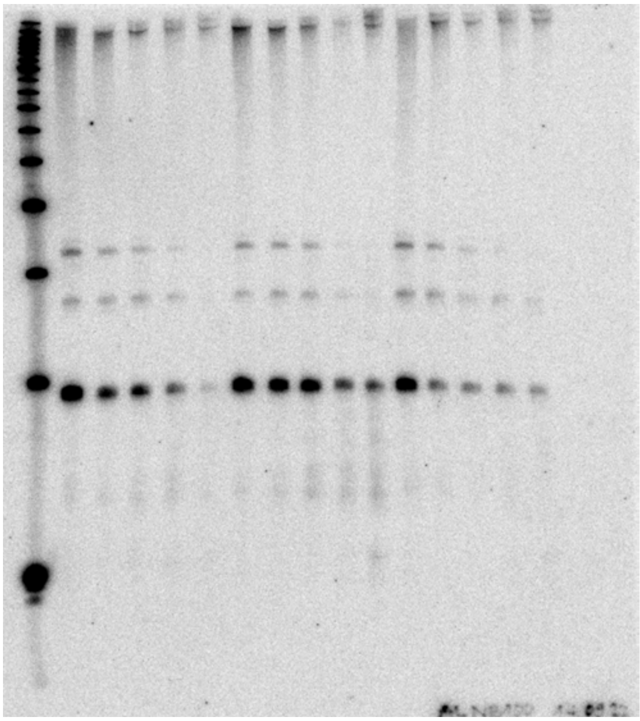

Qrr3

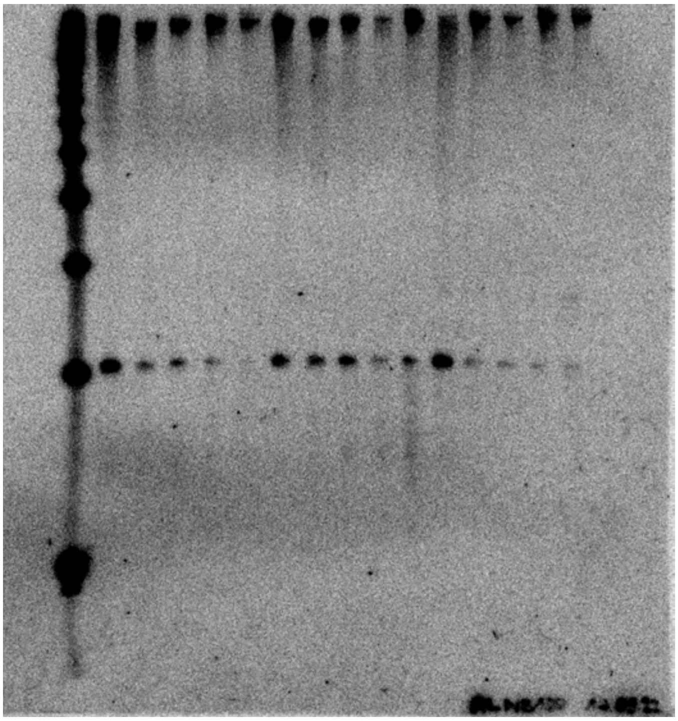

Source data for Fig. S4C

Replicate II

Qrr4

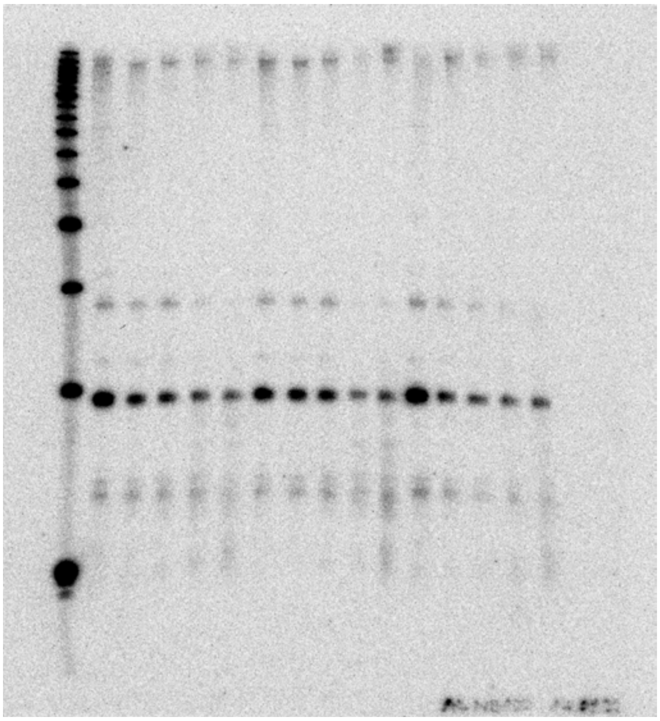

5S rRNA

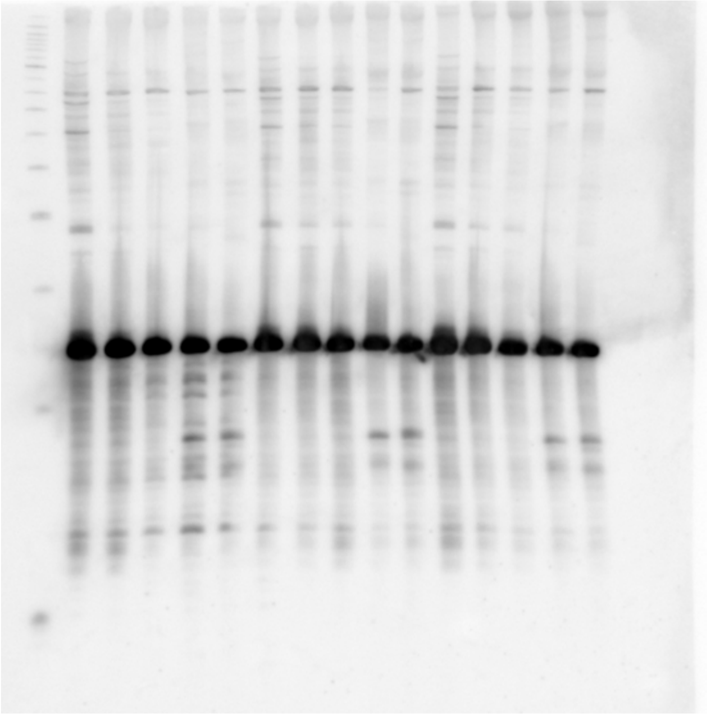

Source data for Fig. S4C

Replicate III

QrrX

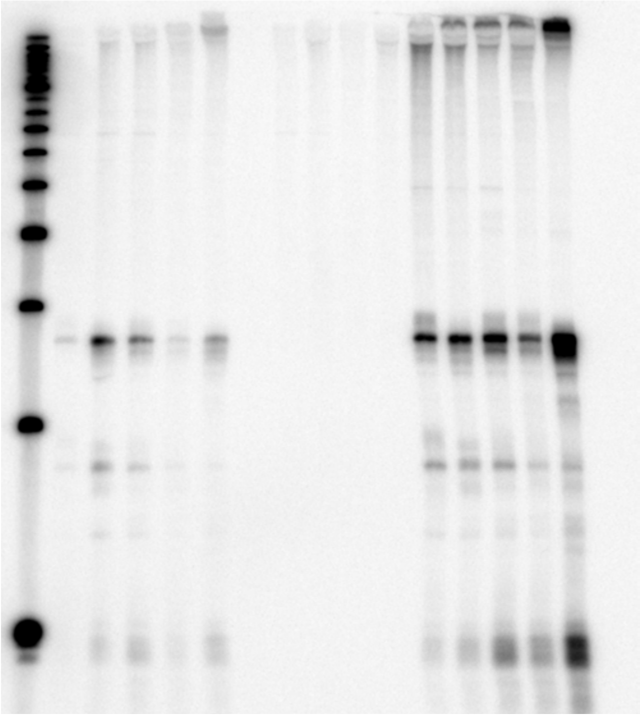

Qrr1

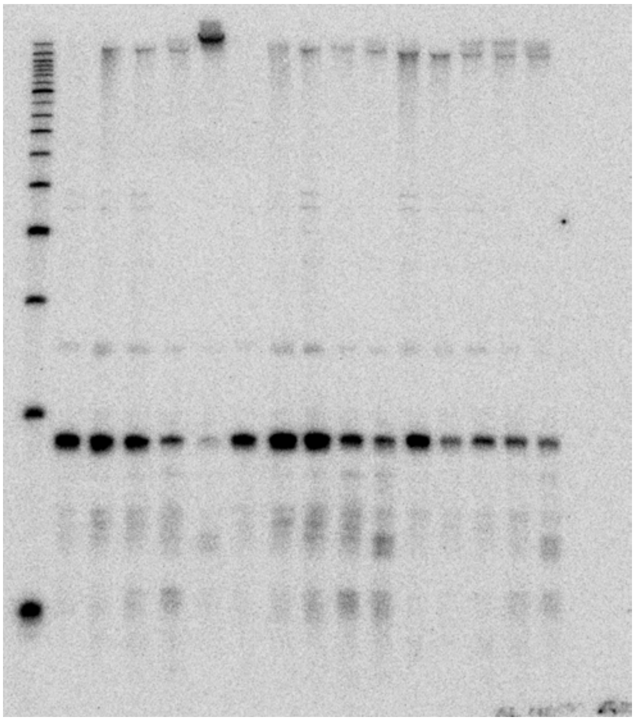

Qrr2

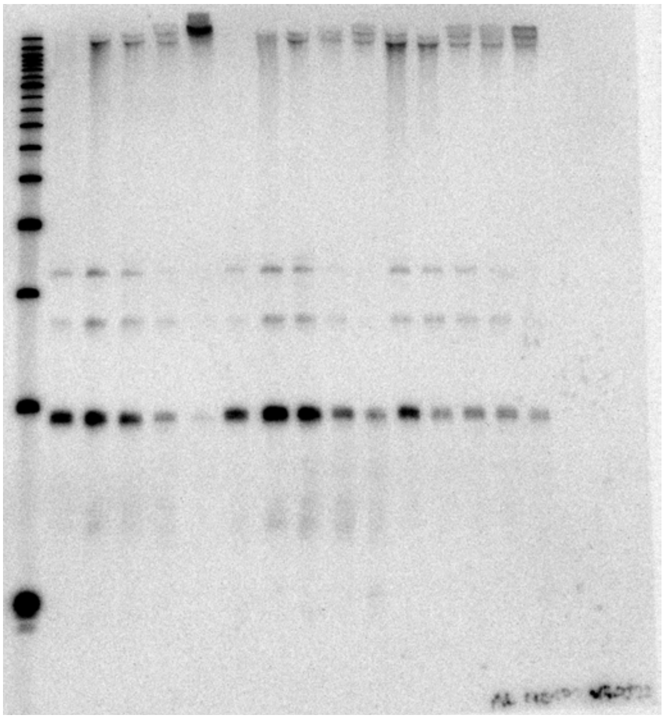

Qrr3

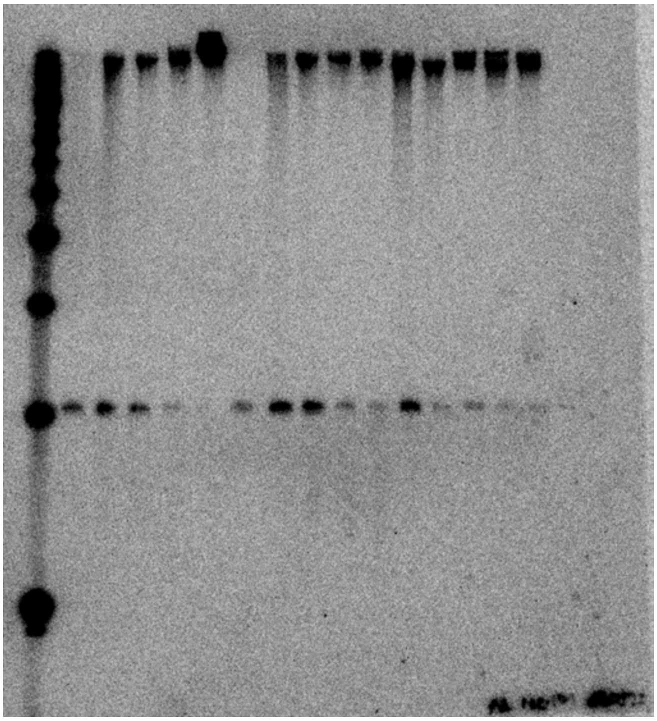

Source data for Fig. S4C

Replicate III

Qrr4

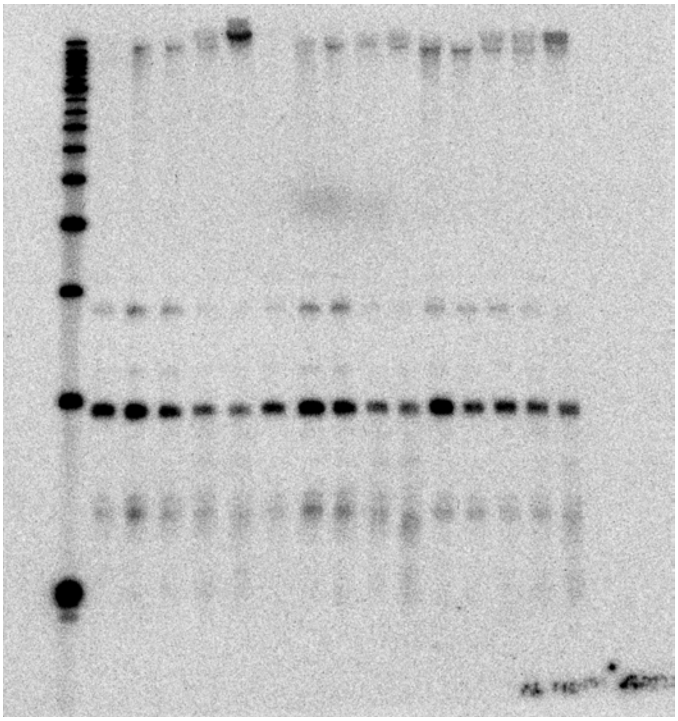

5S rRNA

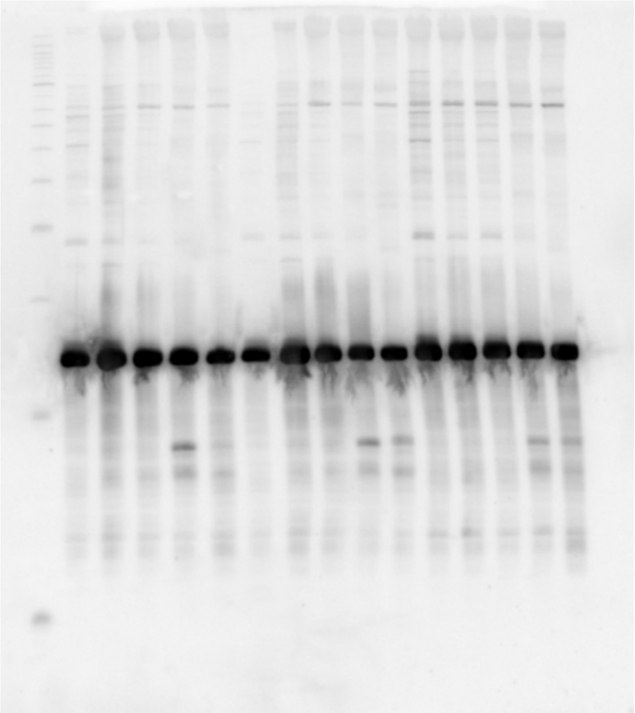

Source data for Fig. S5B

Replicate I

Qrr1

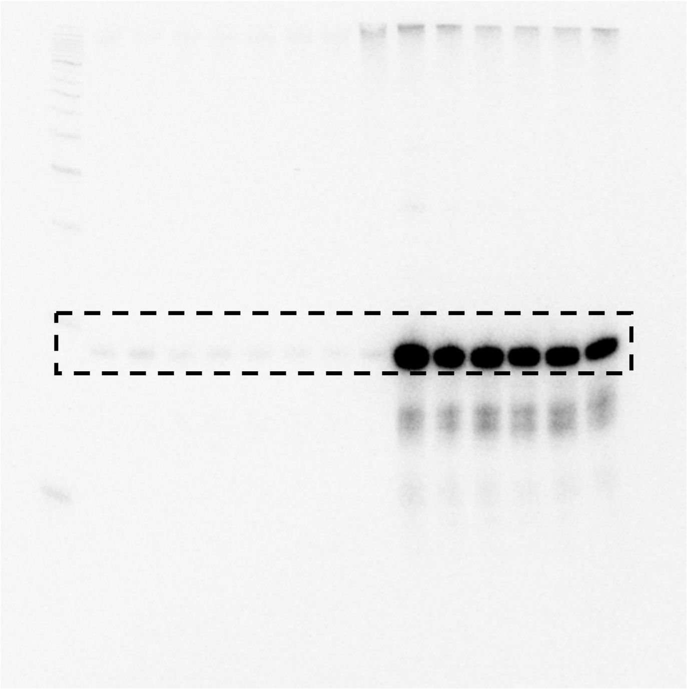

QrrX

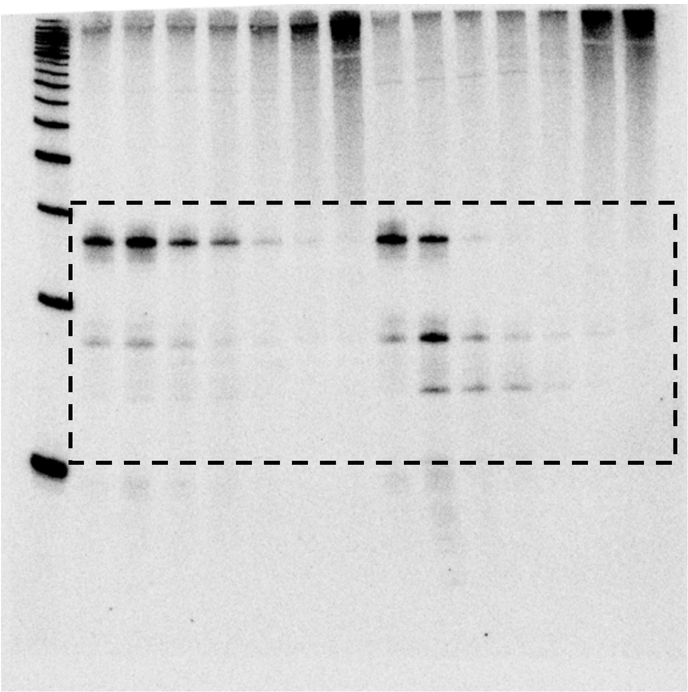

5S rRNA

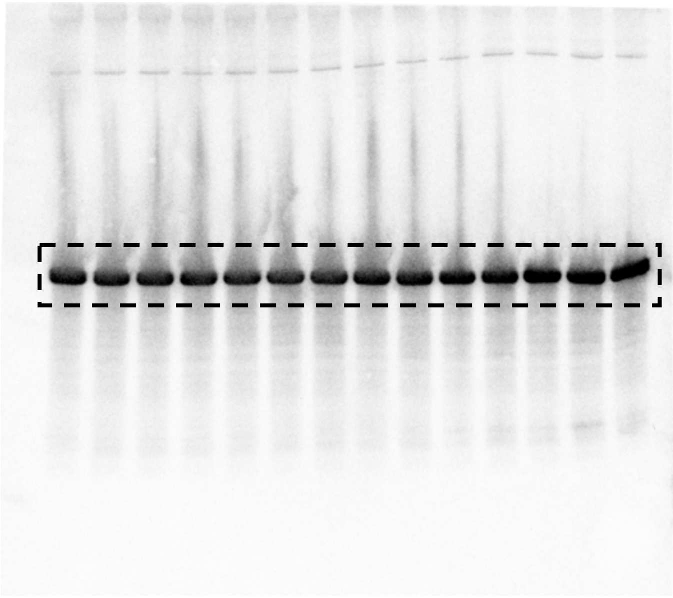

Source data for Fig. S5B

Replicate II

Qrr1

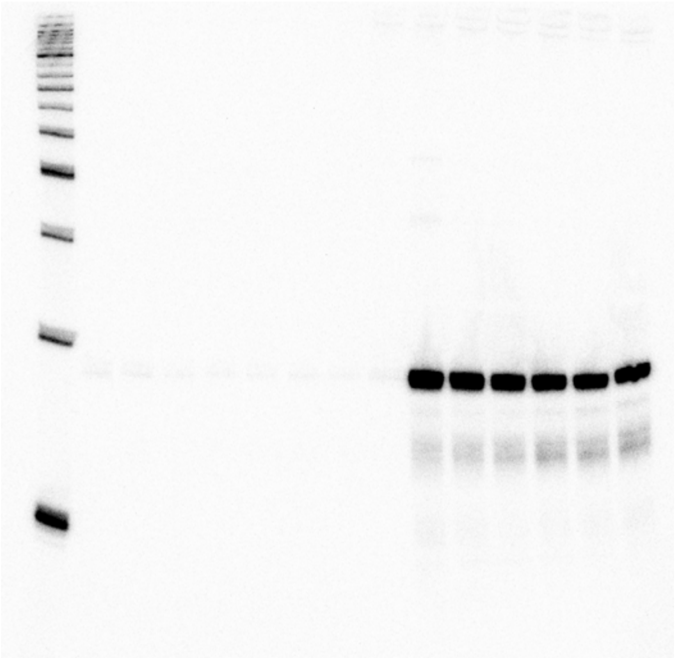

QrrX

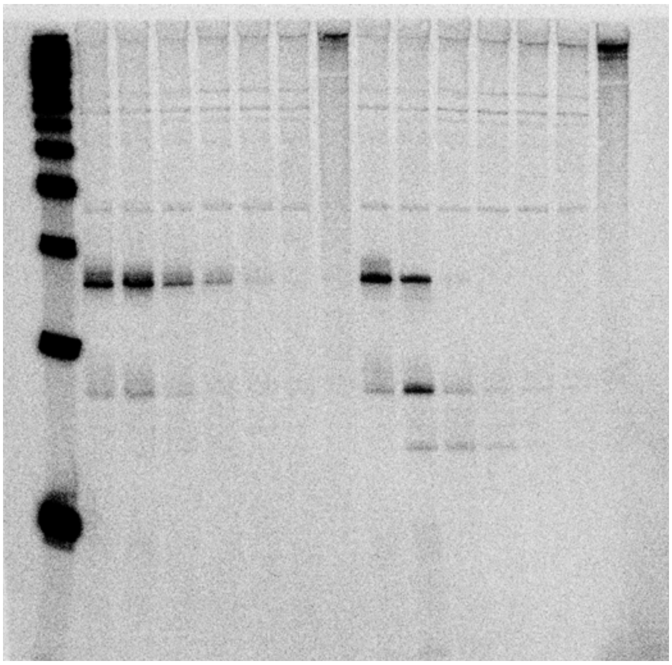

5S rRNA

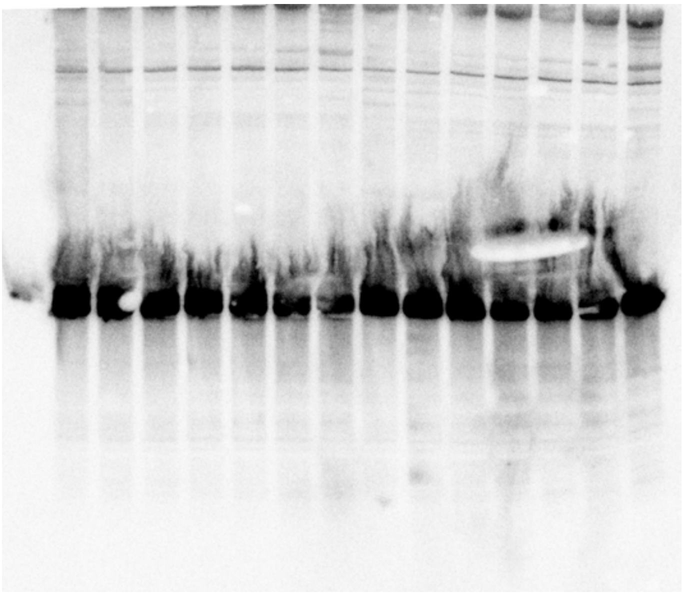

Source data for Fig. S5C

Replicate I

Qrr2

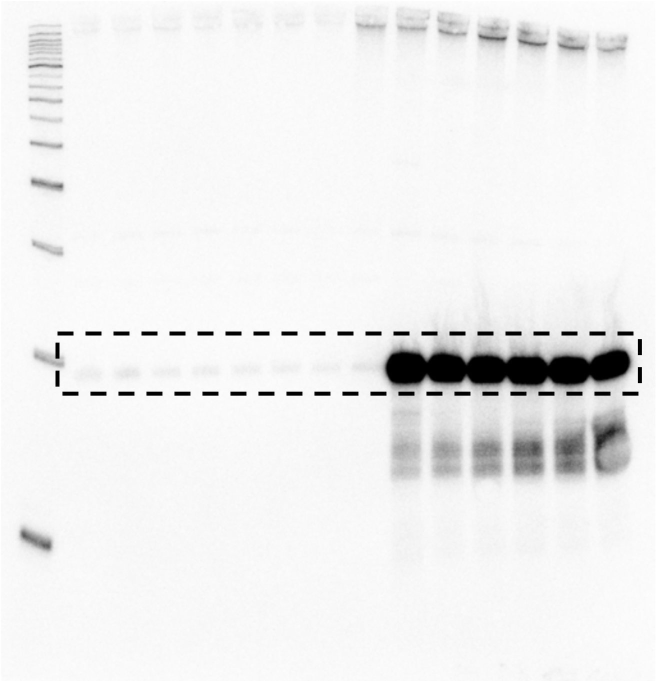

QrrX

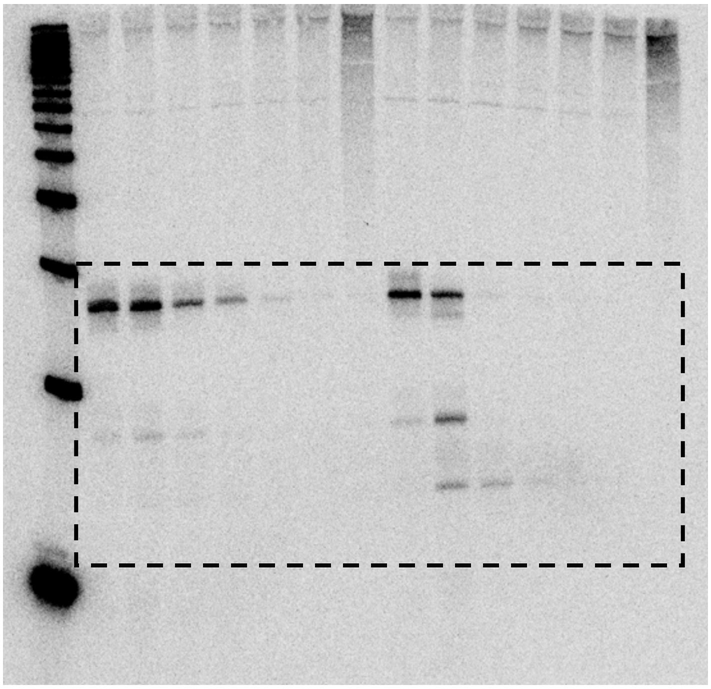

5S rRNA

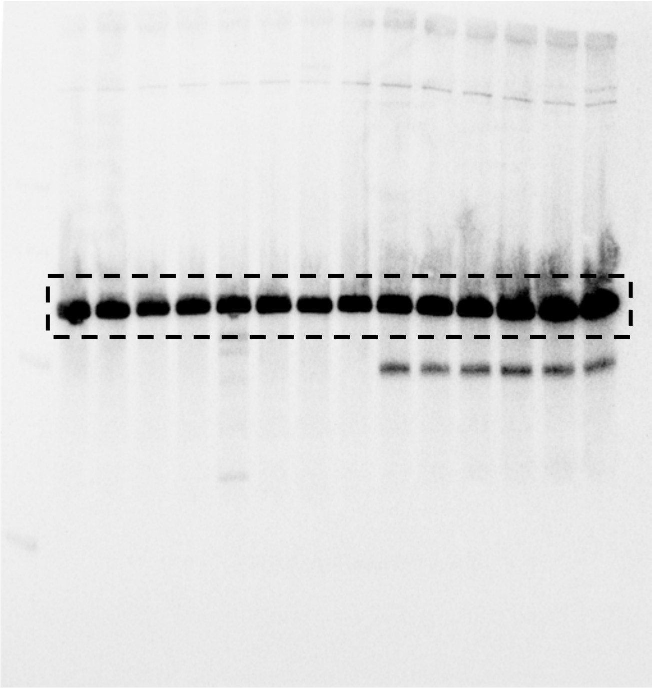

Source data for Fig. S5C

Replicate II

Qrr2

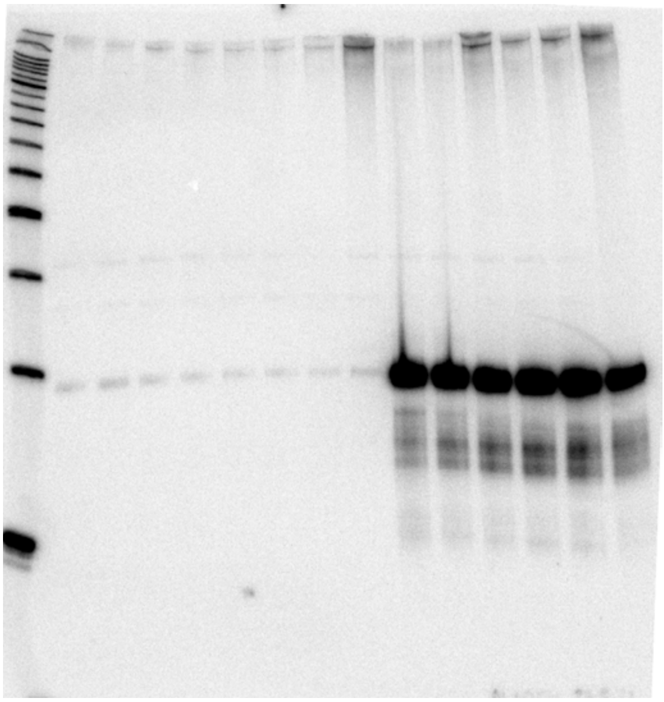

QrrX

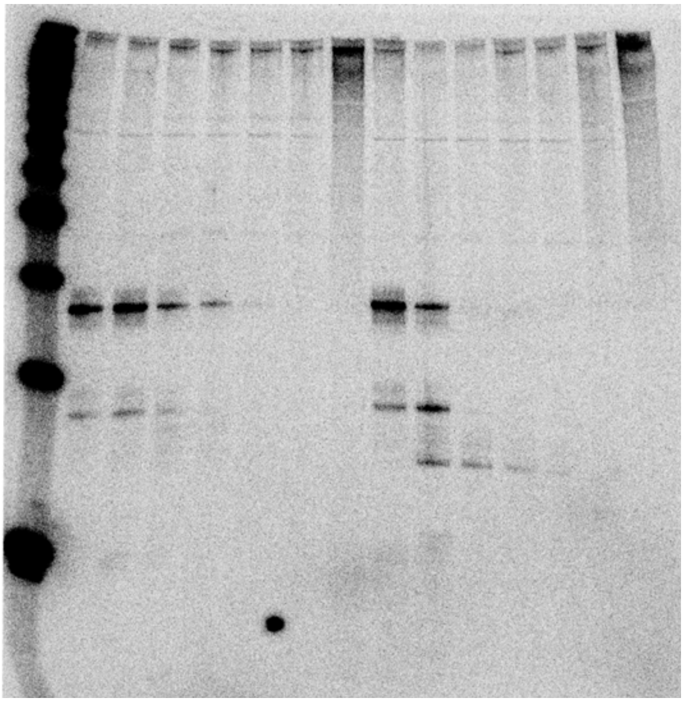

5S rRNA

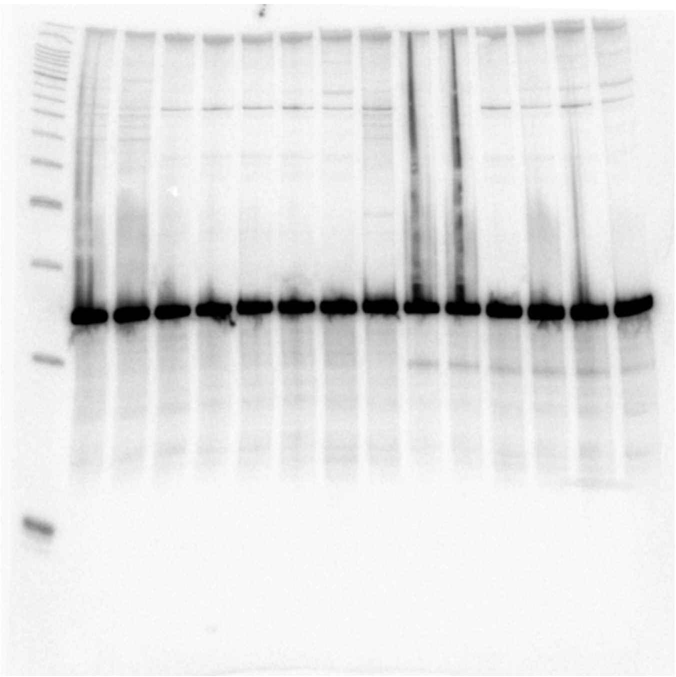

Source data for Fig. S5D

Replicate I

Qrr3

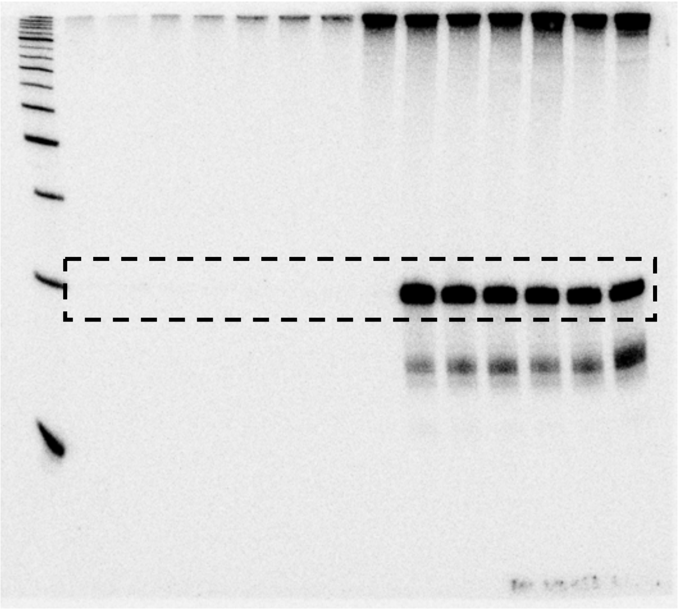

QrrX

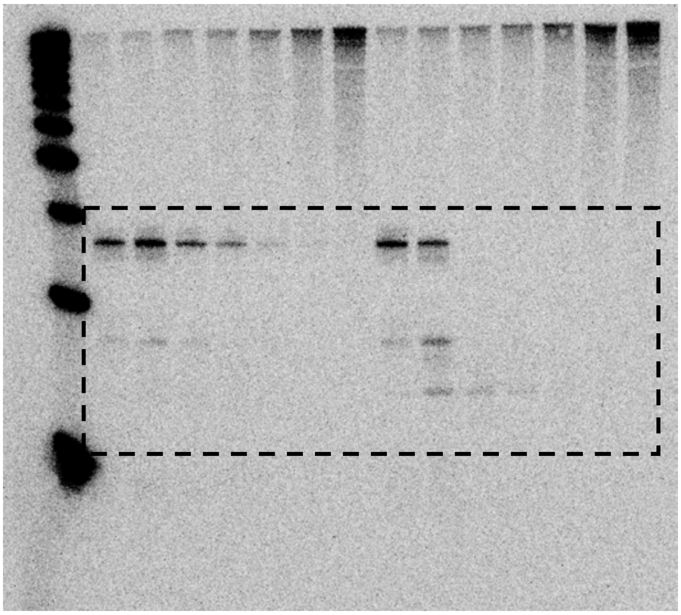

5S rRNA

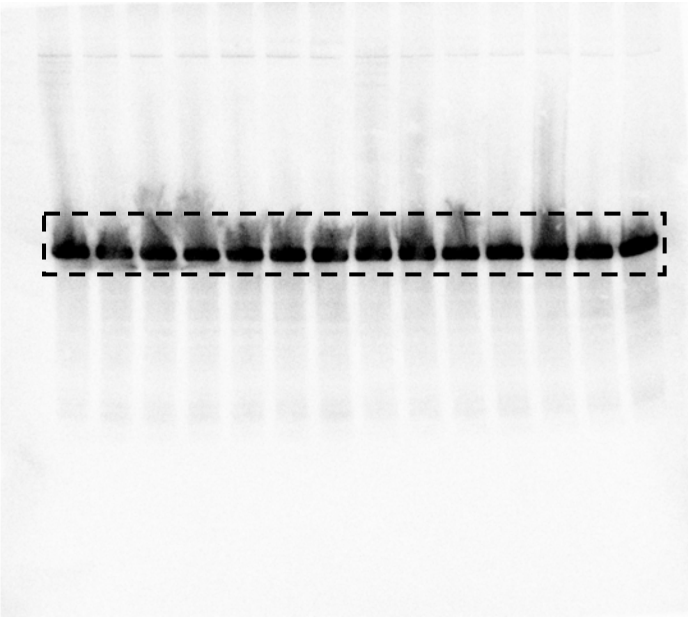

Source data for Fig. S5D

Replicate II

Qrr3

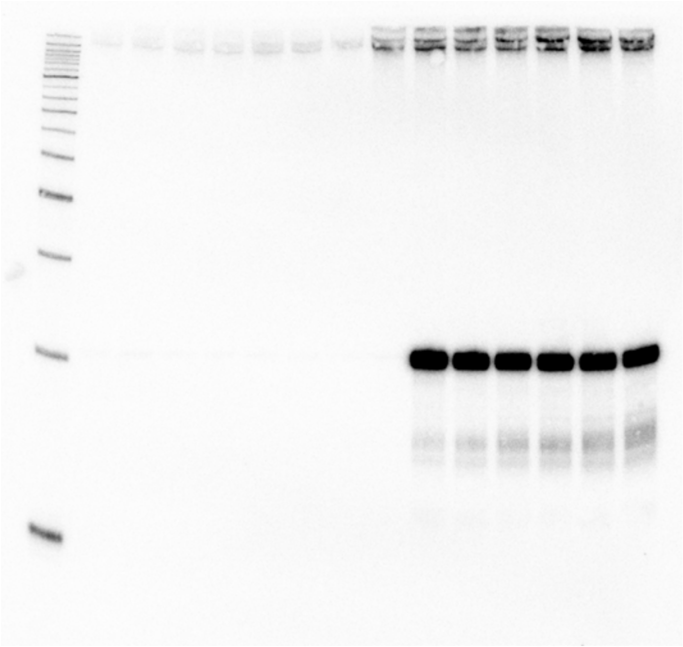

QrrX

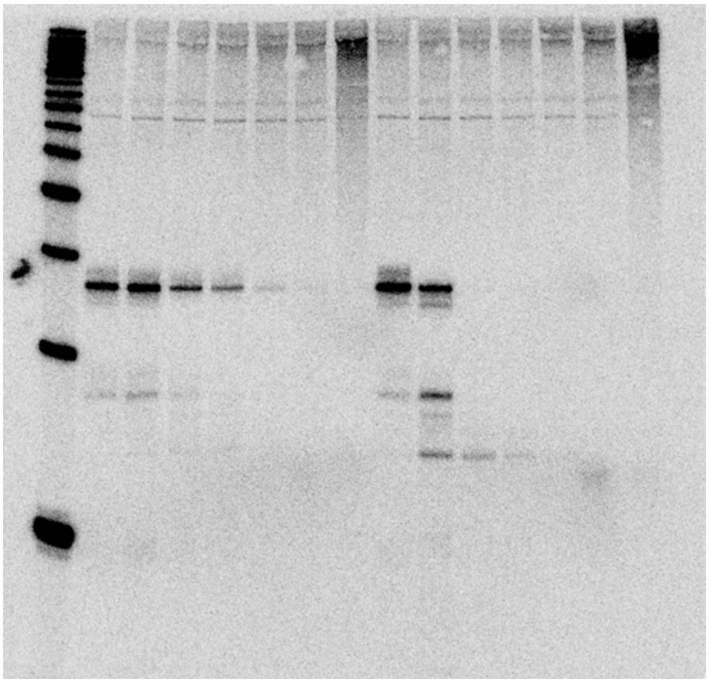

5S rRNA

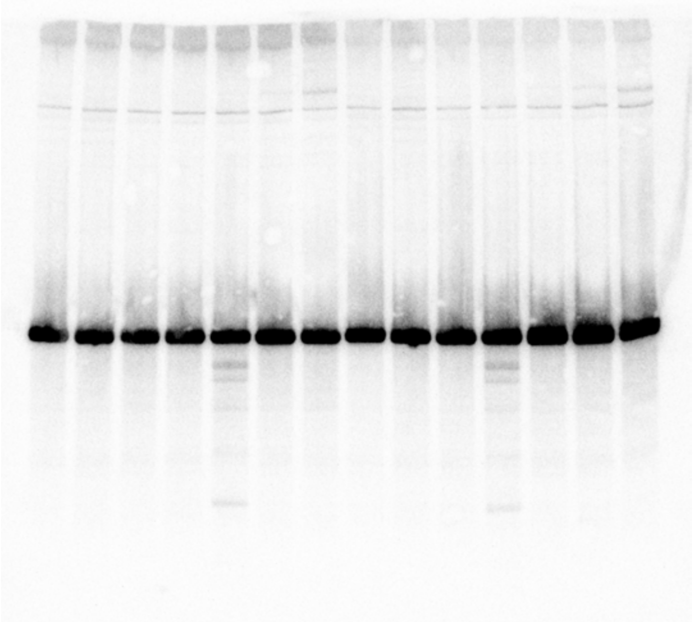

Source data for Fig. S5E

Replicate I

Qrr4

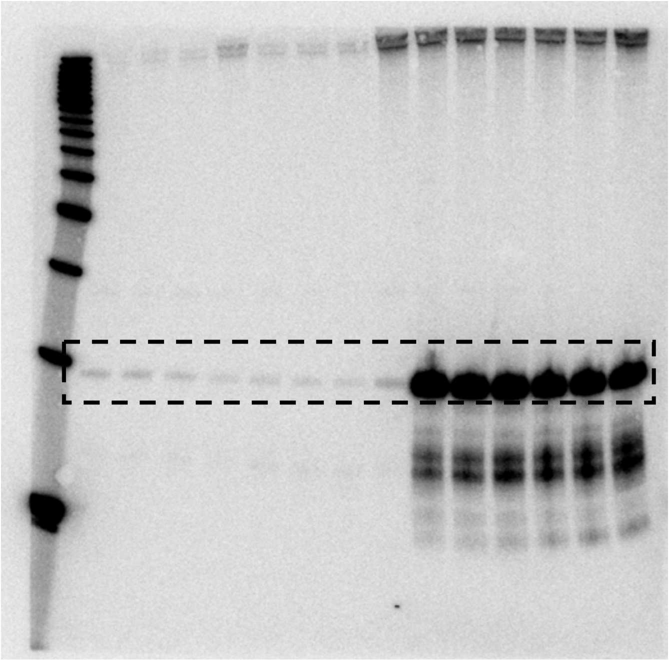

QrrX

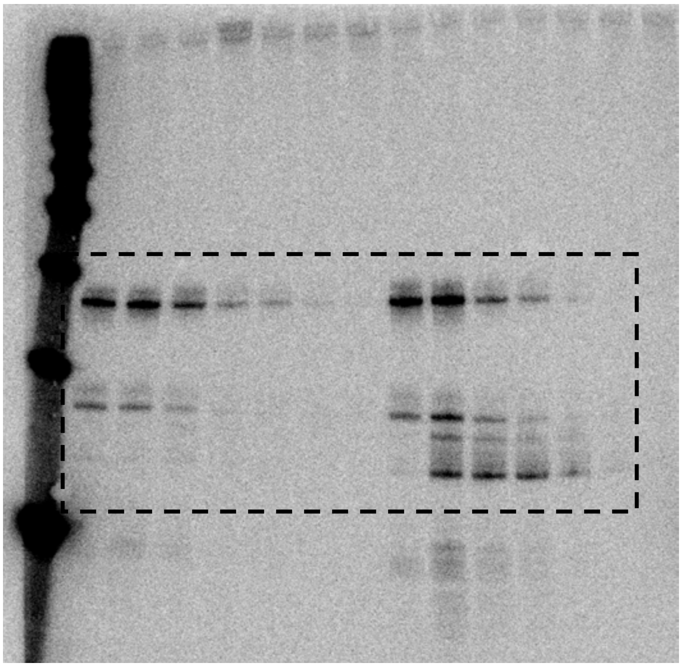

5S rRNA

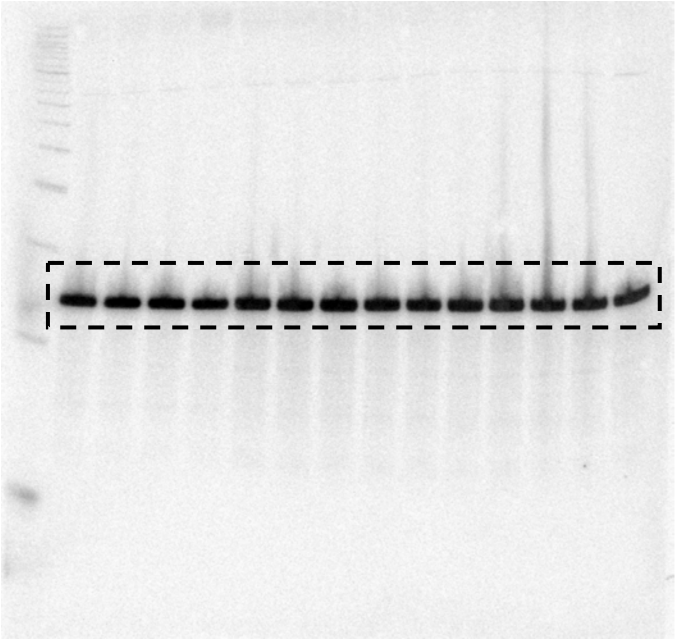

Source data for Fig. S5E

Replicate II

Qrr4

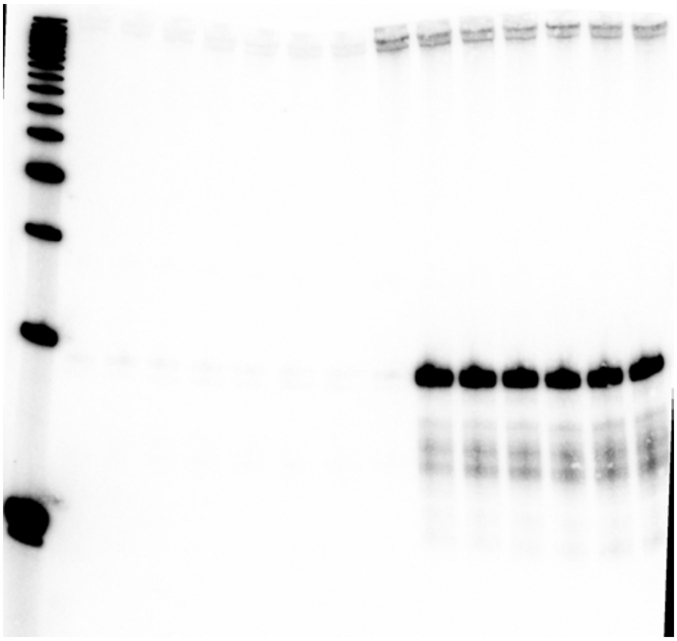

QrrX

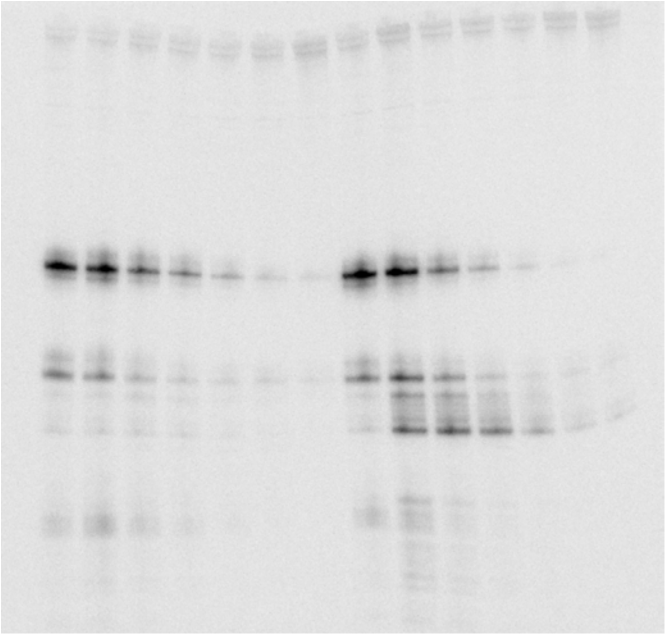

5S rRNA

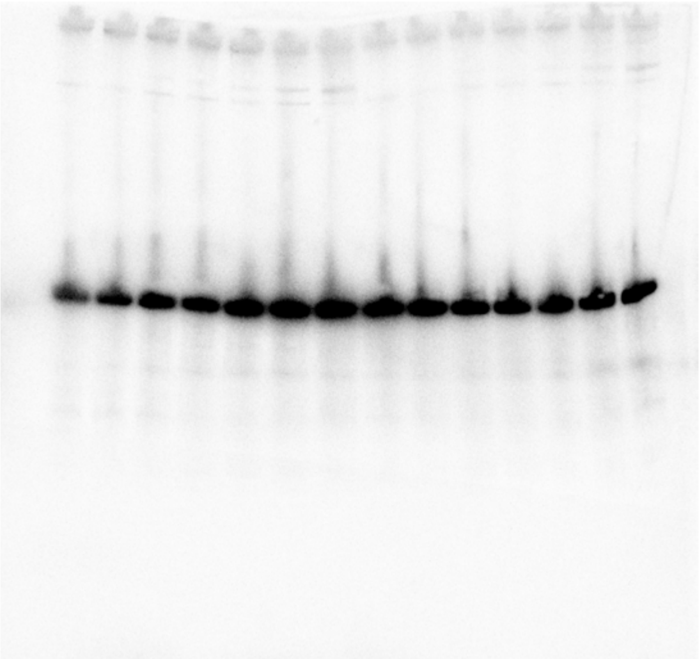

Source data for Fig. S5F

Replicate I

Qrr4

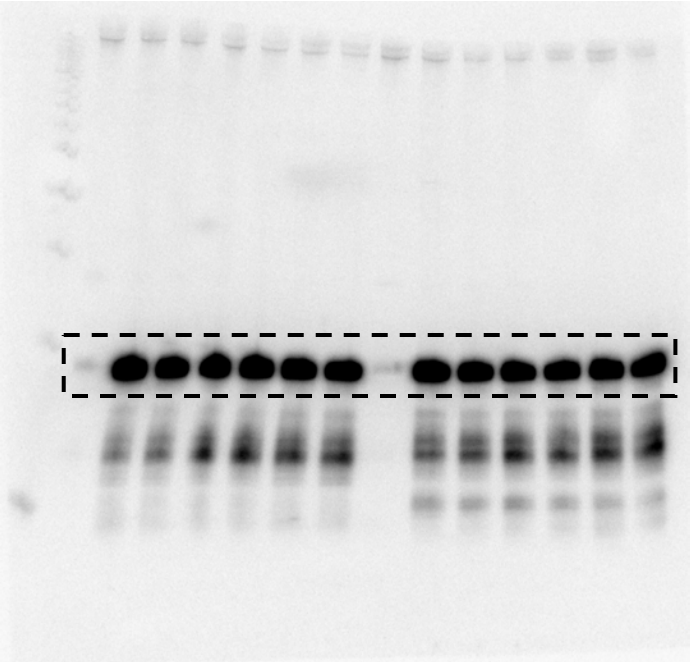

QrrX

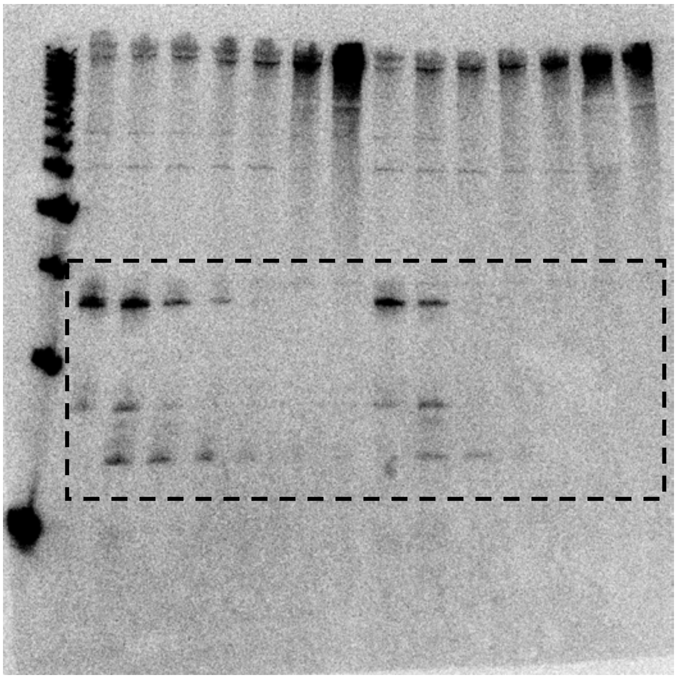

5S rRNA

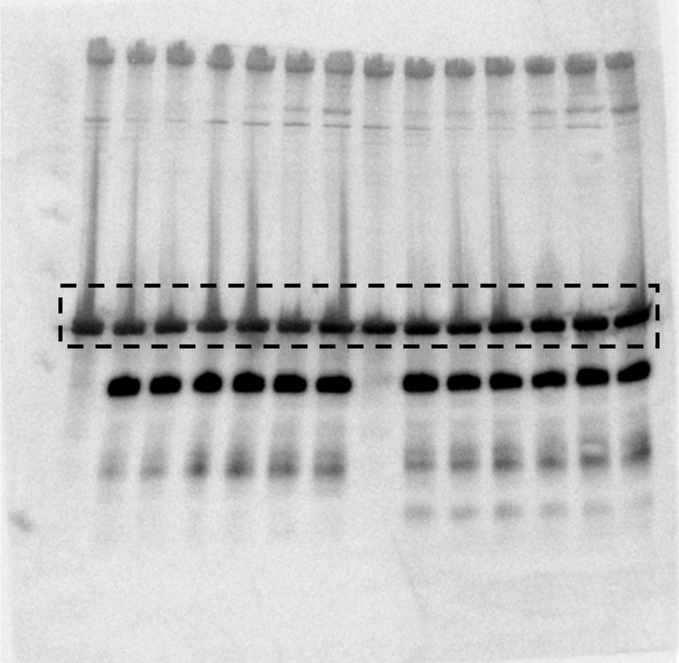

Source data for Fig. S5F

Replicate II

Qrr4

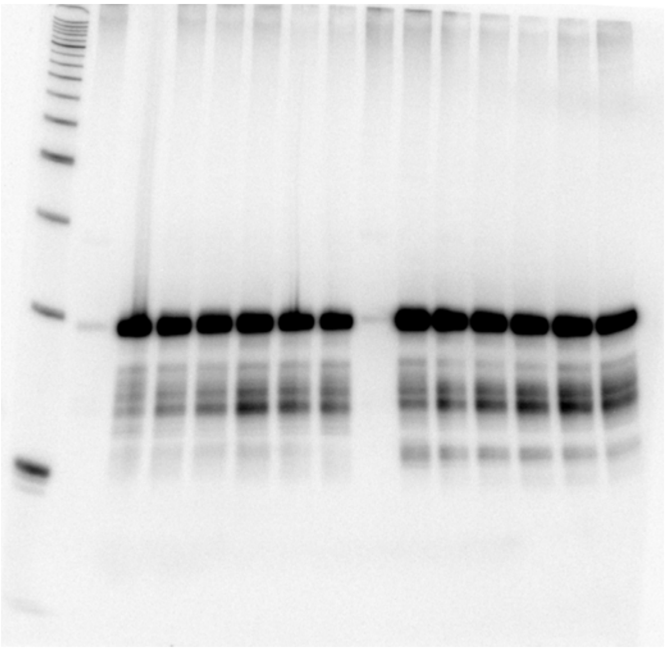

QrrX

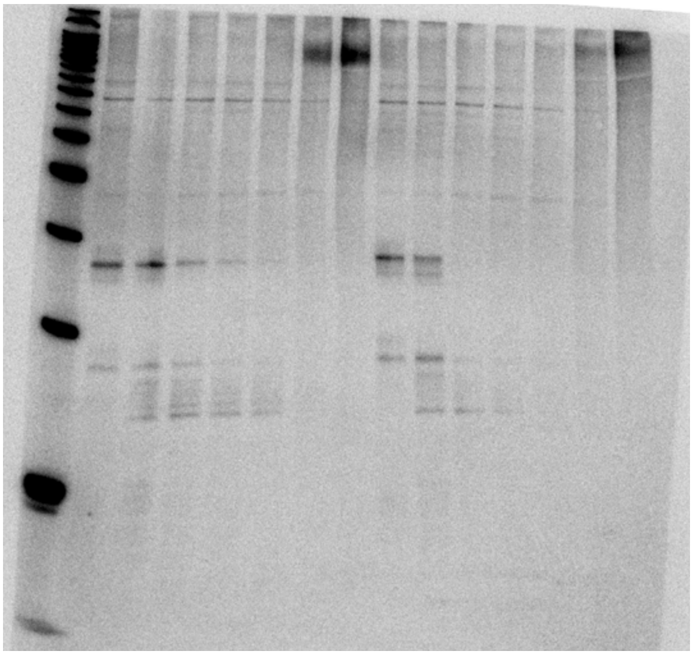

5S rRNA

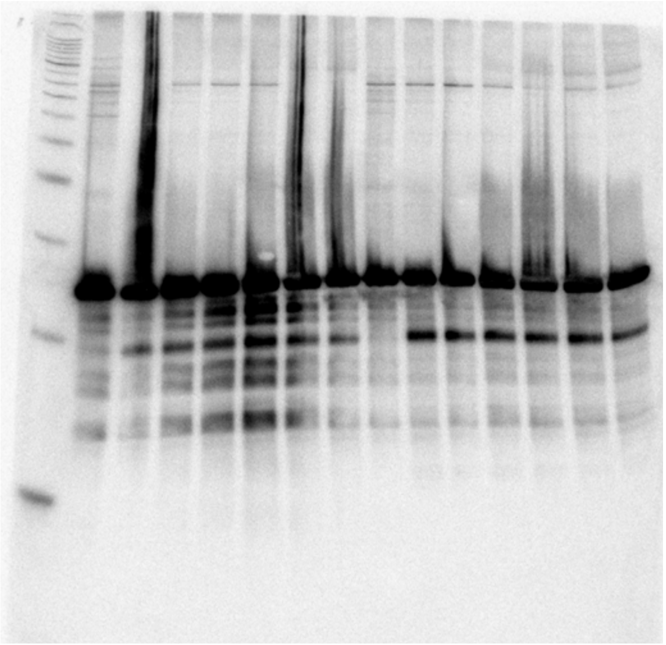

Source data for Fig. S5F

Replicate III

Qrr4

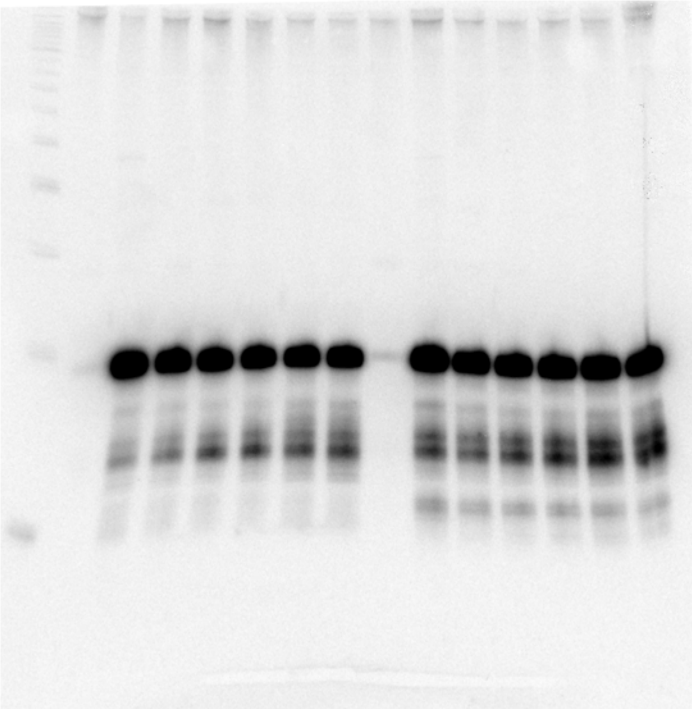

QrrX

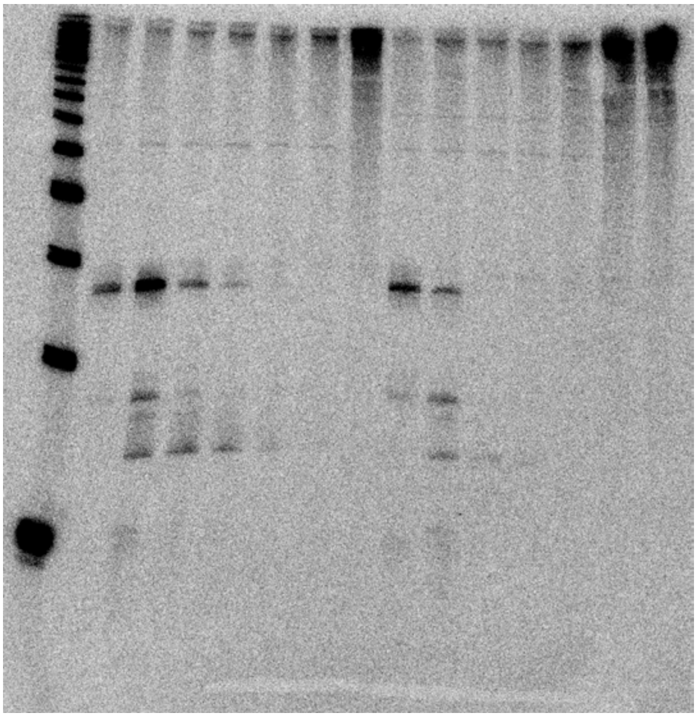

5S rRNA

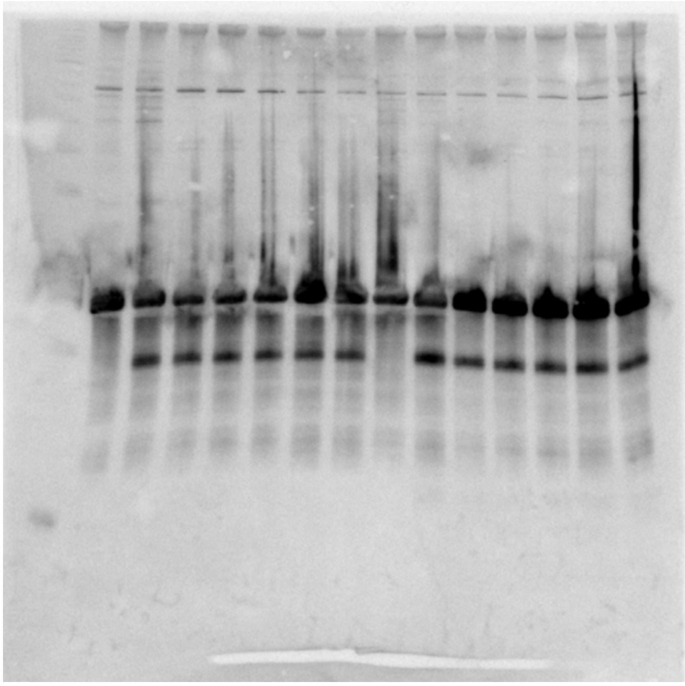

Source data for Fig. S6

Replicate I

QrrX

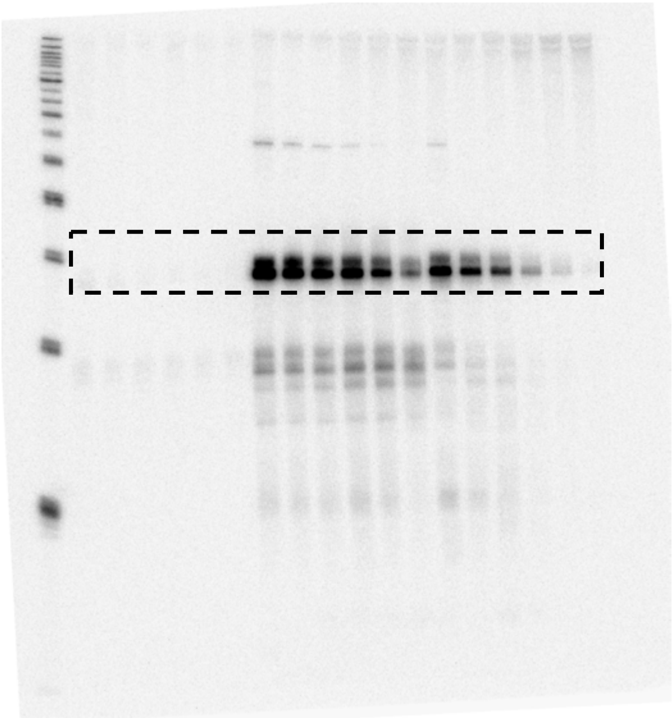

Qrr4

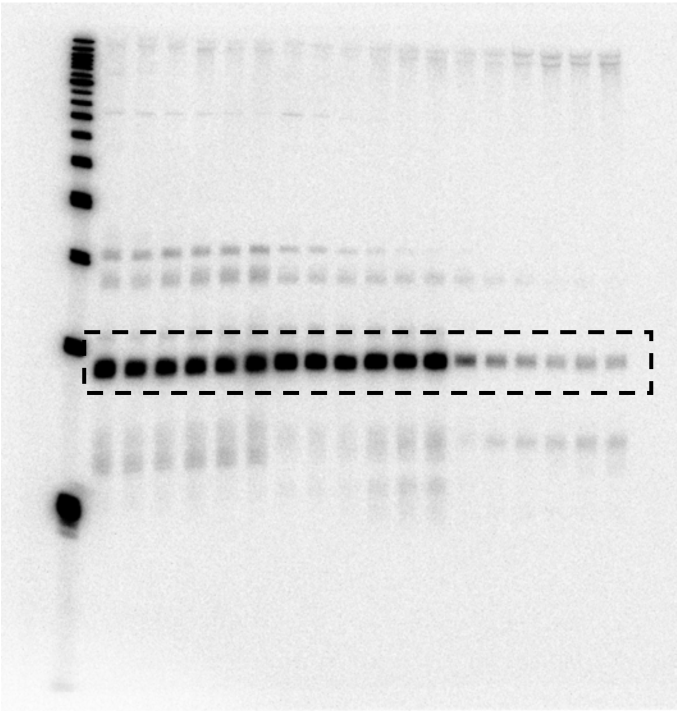

5S rRNA

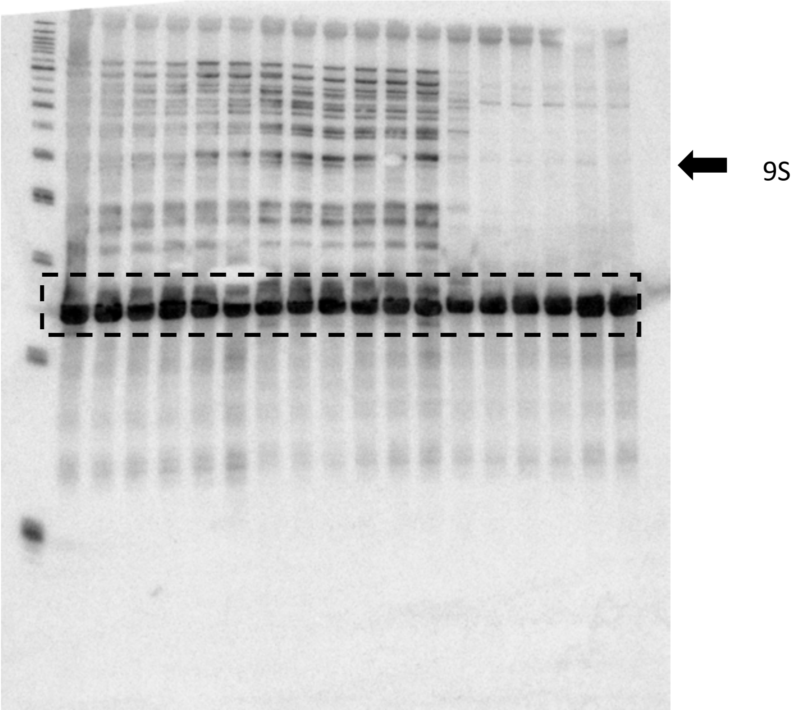

Source data for Fig. S6

Replicate II

QrrX

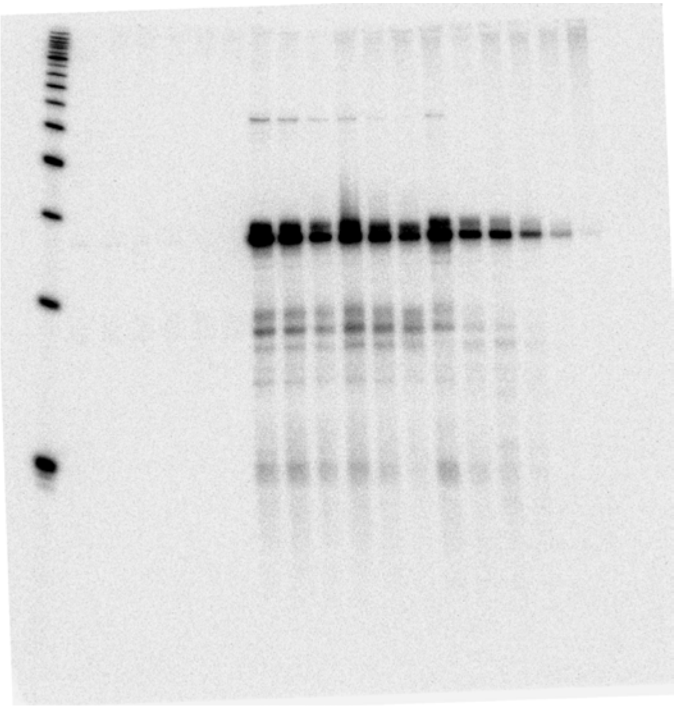

Qrr4

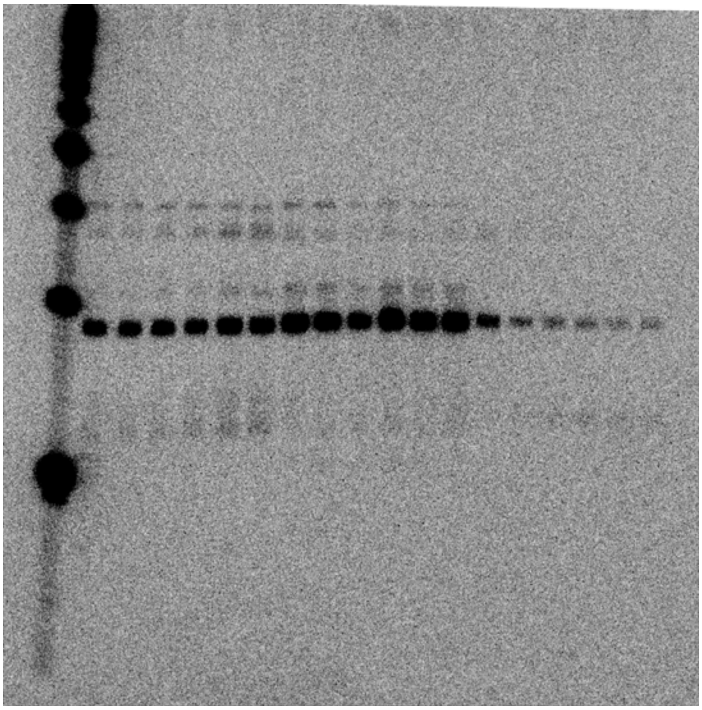

5S rRNA

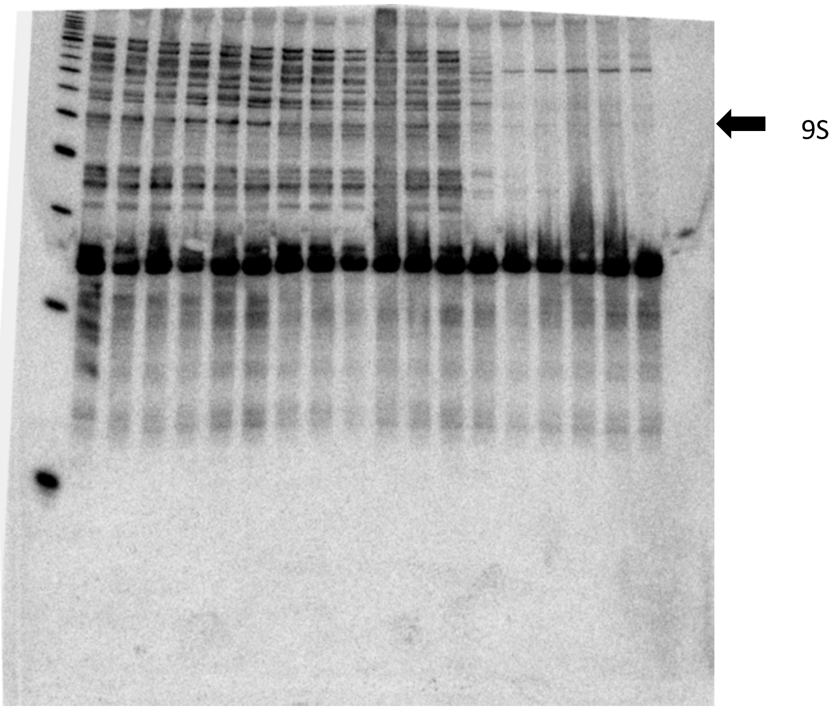

Source data for Fig. S6

Replicate III

QrrX

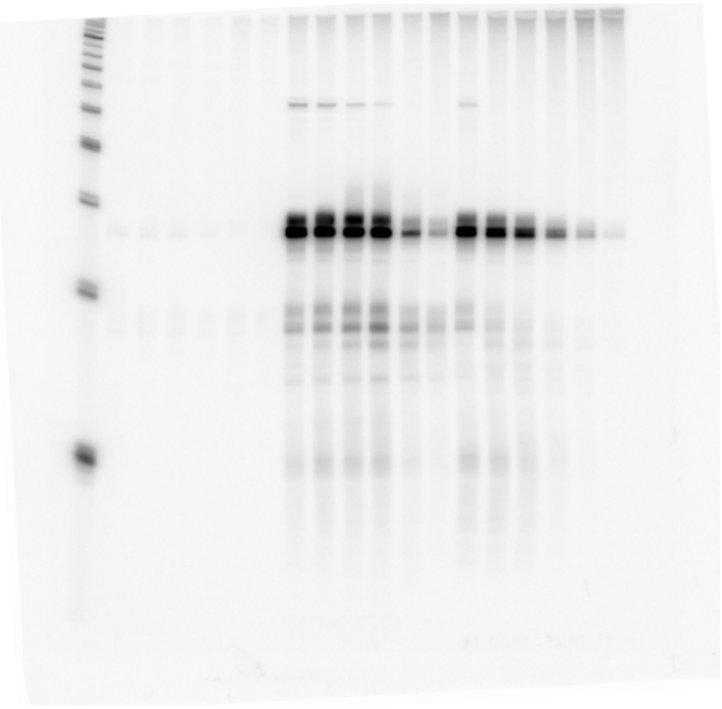

Qrr4

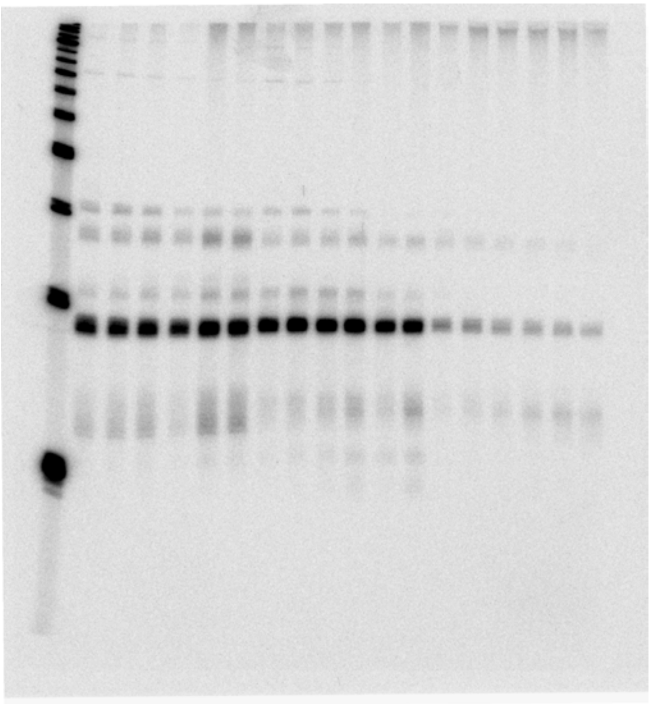

5S rRNA

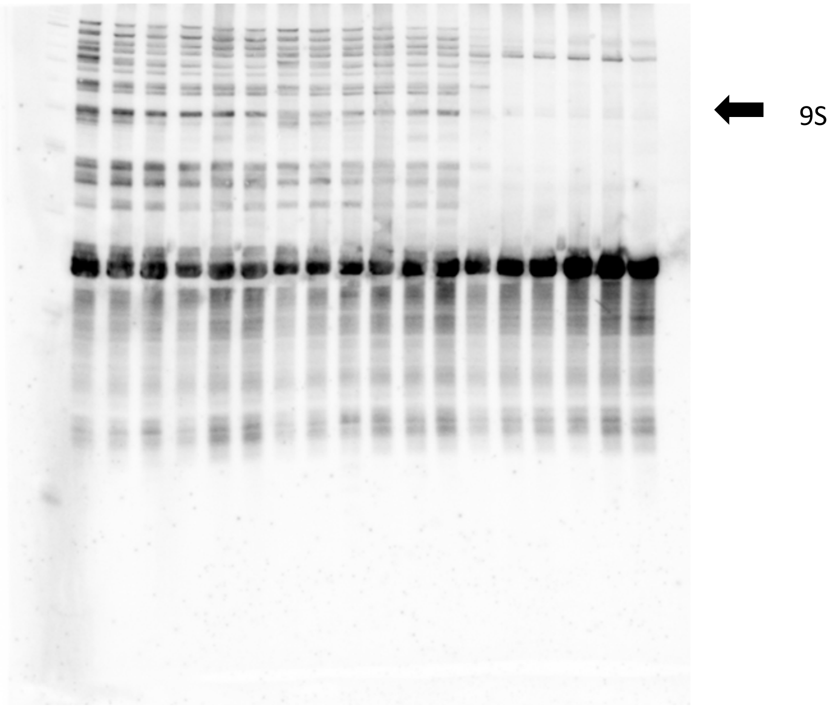

Source data for Fig. S7E

Replicate I

QrrX

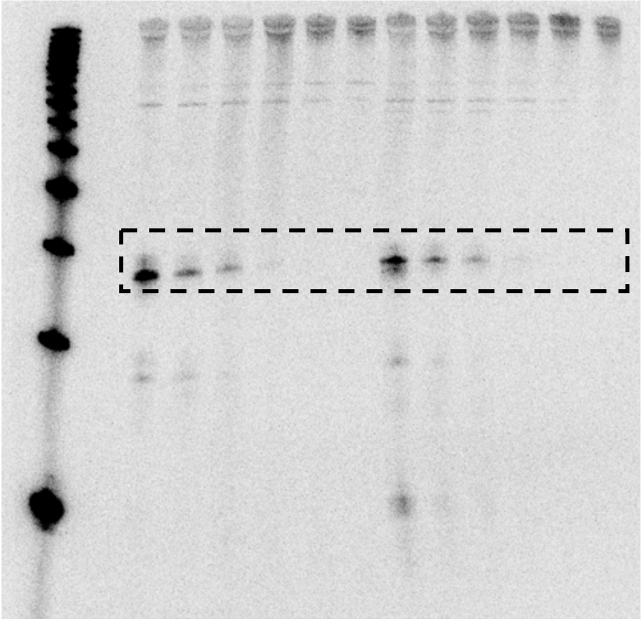

5S rRNA

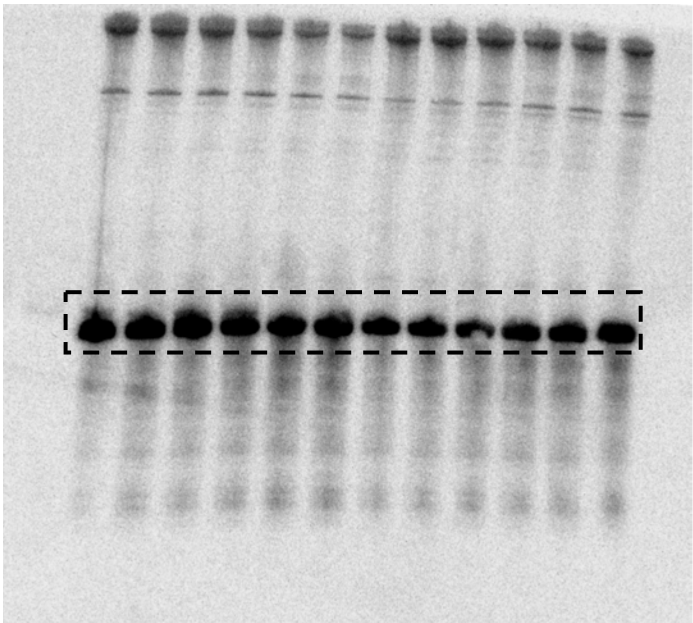

Replicate II

QrrX

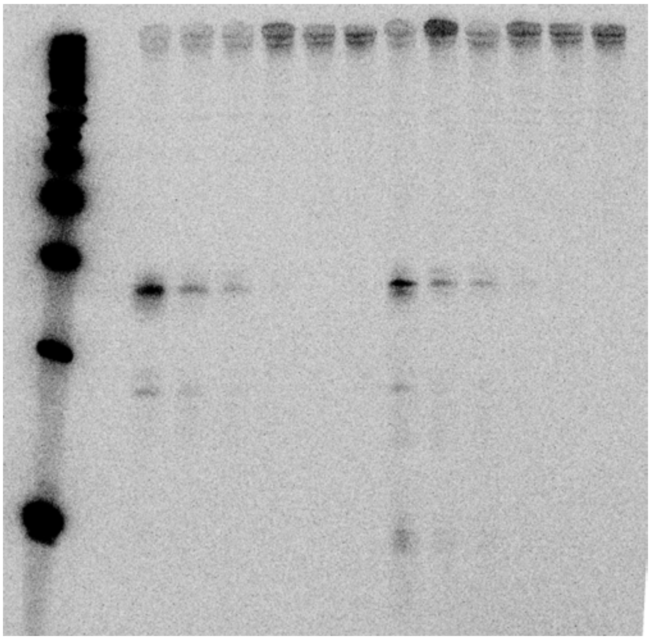

5S rRNA

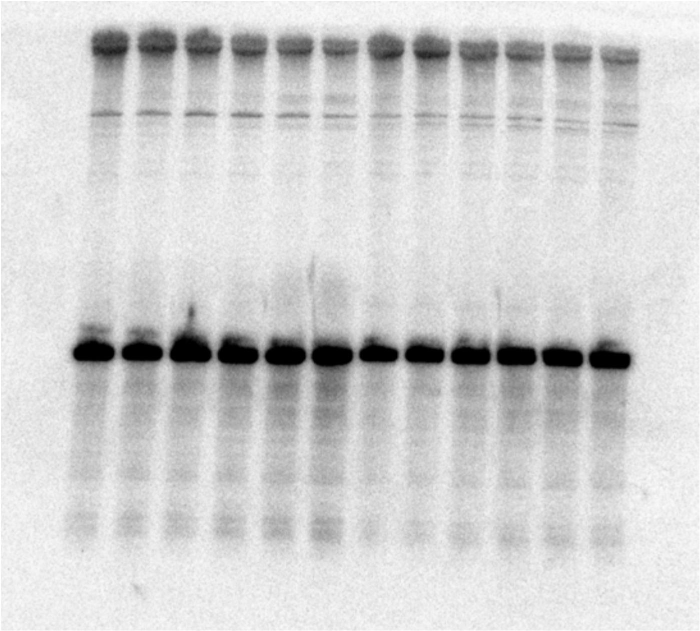

Source data for Fig. S7E

Replicate III

QrrX

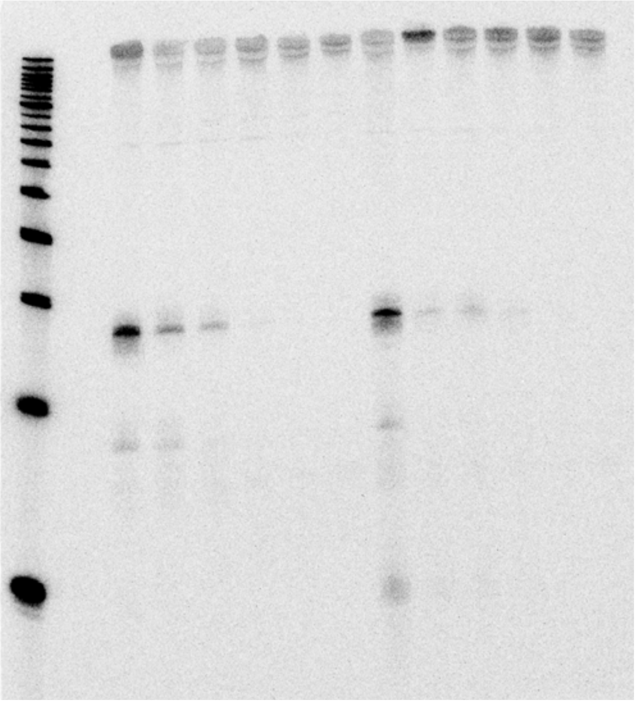

5S rRNA

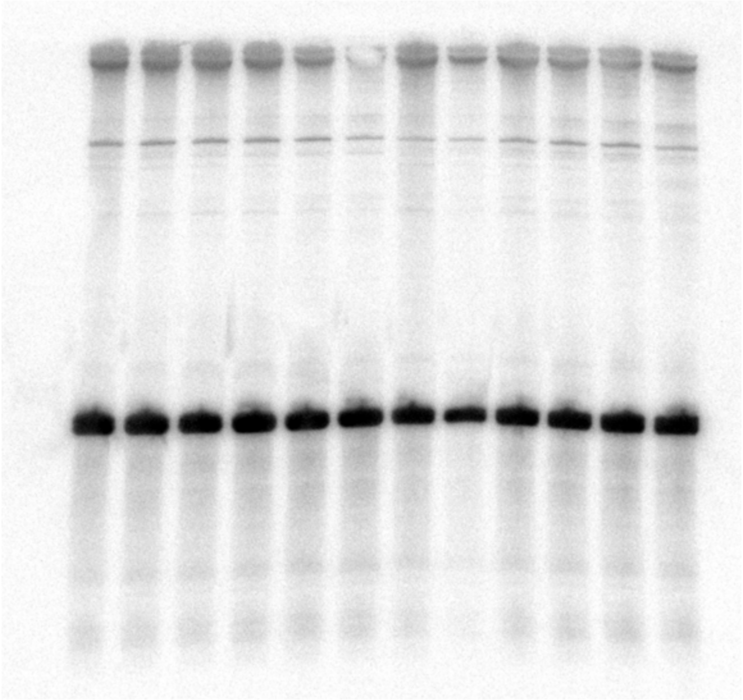

Source data for Fig. S7F

Replicate I

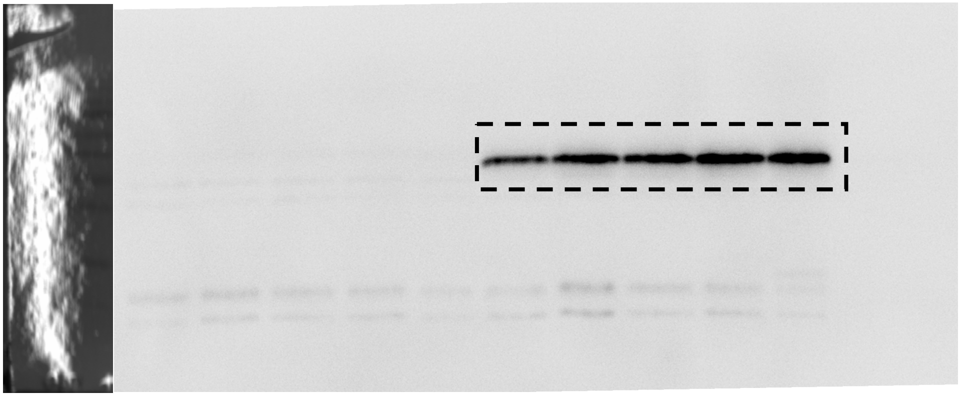

anti-FLAG

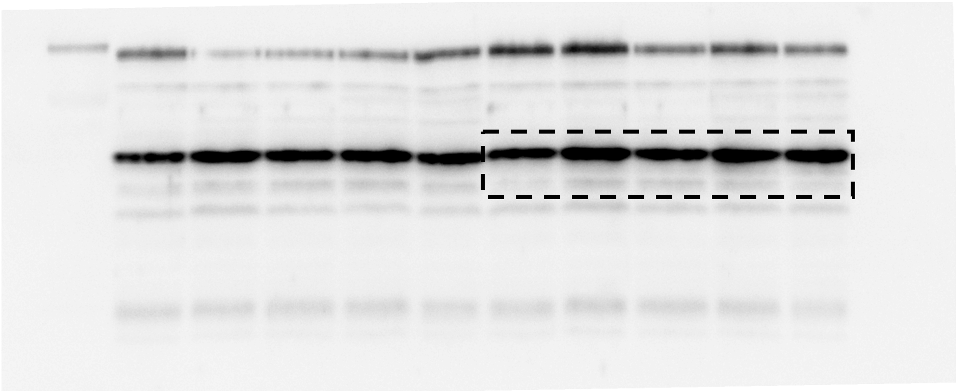

anti-RNAP

Replicate II

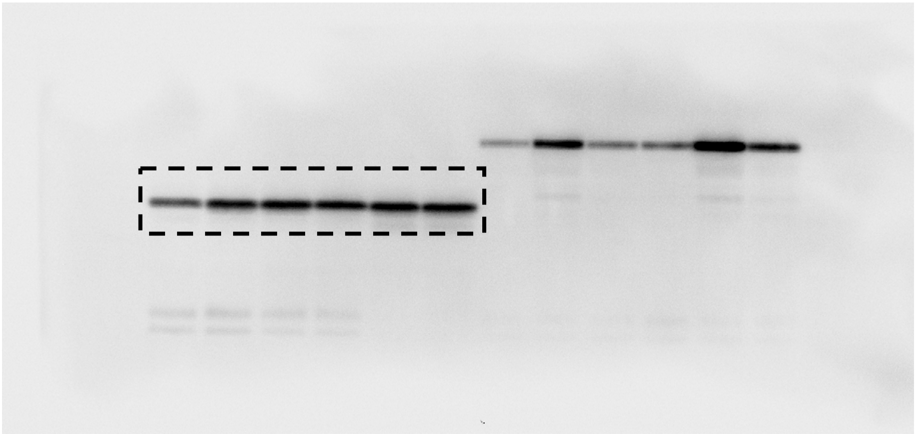

anti-FLAG

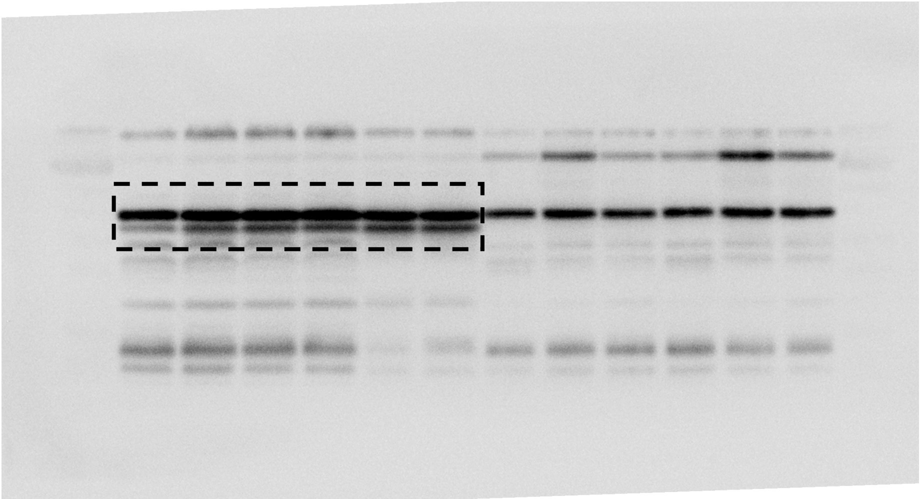

anti-RNAP

Source data for Fig. S7F

Replicate III

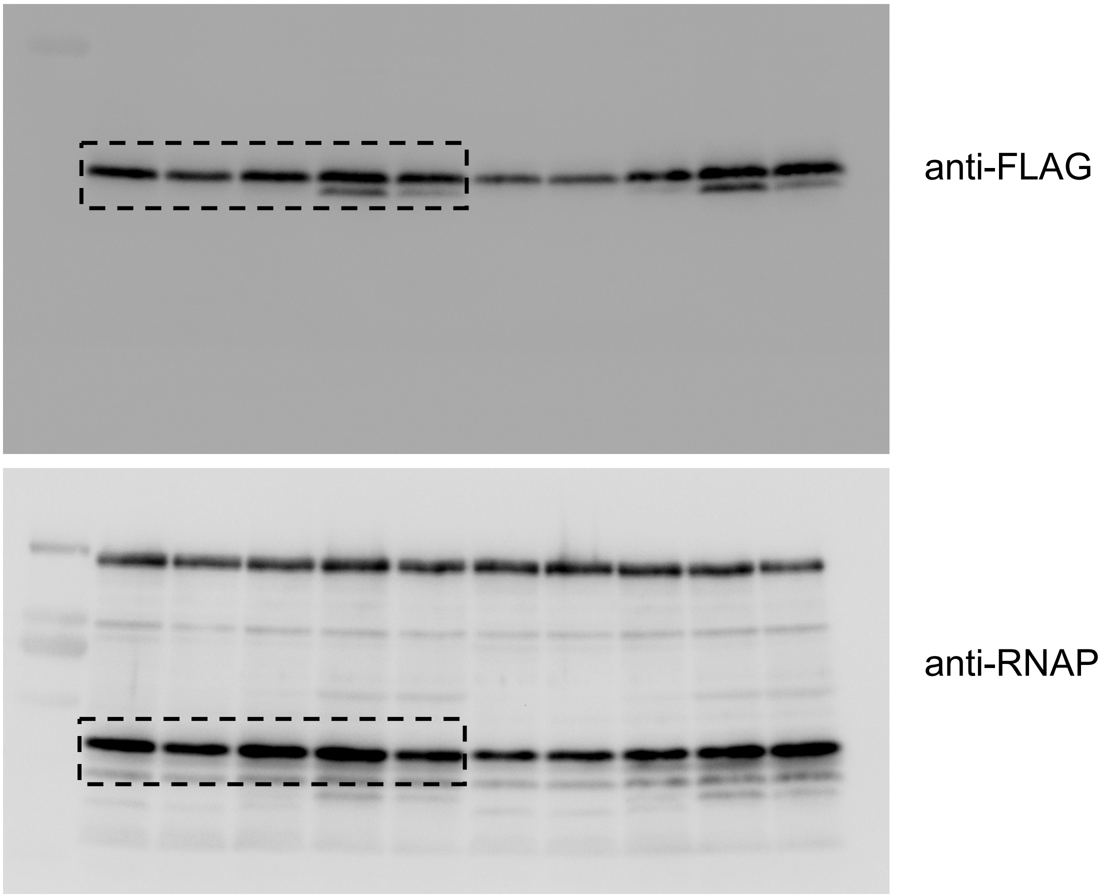

Supplement: Supplementary file 6 — Source Data [file 41467_2022_35261_MOESM6_ESM.zip › Source Data/Source Data.pdf]
